# Diurnal Variations of Human Circulating Cell-Free Micro-RNA

**DOI:** 10.1371/journal.pone.0160577

**Published:** 2016-08-05

**Authors:** Niels H. H. Heegaard, Anting Liu Carlsen, Berit Lilje, Kim Lee Ng, Mette E. Rønne, Henrik L. Jørgensen, Henriette Sennels, Jan Fahrenkrug

**Affiliations:** 1 Department of Autoimmunology and Biomarkers, Statens Serum Institut, Copenhagen, Denmark; 2 Department of Clinical Biochemistry and Pharmacology, Odense University Hospital, Odense, Denmark; 3 Department of Congenital Disorders, Statens Serum Institut, Copenhagen, Denmark; 4 Department of Microbiology and Infection Control, Statens Serum Institut, Copenhagen, Denmark; 5 Department of Clinical Biochemistry, Faculty of Health Science, Bispebjerg Hospital, University of Copenhagen, Denmark; 6 Department of Clinical Biochemistry, Faculty of Health Science, Rigshospitalet - Glostrup, University of Copenhagen, Copenhagen, Denmark; University of Texas Southwestern Medical Center, UNITED STATES

## Abstract

A 24-hour light and dark cycle-dependent rhythmicity pervades physiological processes in virtually all living organisms including humans. These regular oscillations are caused by external cues to endogenous, independent biological time-keeping systems (clocks). The rhythm is reflected by gene expression that varies in a circadian and specific fashion in different organs and tissues and is regulated largely by dynamic epigenetic and post-transcriptional mechanisms. This leads to well-documented oscillations of specific electrolytes, hormones, metabolites, and plasma proteins in blood samples. An emerging, important class of gene regulators is short single-stranded RNA (micro-RNA, miRNA) that interferes post-transcriptionally with gene expression and thus may play a role in the circadian variation of gene expression. MiRNAs are promising biomarkers by virtue of their disease-specific tissue expression and because of their presence as stable entities in the circulation. However, no studies have addressed the putative circadian rhythmicity of circulating, cell-free miRNAs. This question is important both for using miRNAs as biological markers and for clues to miRNA function in the regulation of circadian gene expression. Here, we investigate 92 miRNAs in plasma samples from 24 young male, healthy volunteers repeatedly sampled 9 times during a 24-hour stay in a regulated environment. We demonstrate that a third (26/79) of the measurable plasma miRNAs (using RT-qPCR on a microfluidic system) exhibit a rhythmic behavior and are distributed in two main phase patterns. Some of these miRNAs weakly target known clock genes and many have strong targets in intracellular MAPK signaling pathways. These novel findings highlight the importance of considering bio-oscillations in miRNA biomarker studies and suggest the further study of a set of specific circulating miRNAs in the regulation and functioning of biological clocks.

## Introduction

The levels of many biological molecules vary according to a biological rhythm, *i*.*e*., a cyclic change that roughly follows a 24-hour period. The periodicity is endogenously generated (circadian rhythms) but typically instructed (probably through dynamic epigenetic changes [1]) by the day/night light cycle (the diurnal-nocturnal cycle). Apart from being of considerable basic interest in physiology and disease, awareness of such biomolecular oscillations is of obvious importance for measurements in laboratory medicine and for studies developing *e*.*g*. diagnostic biomarkers. In humans, the principal circadian oscillator is found in the suprachiasmatic nucleus (SCN) of the hypothalamus. Several peripheral local clocks supplement it and together they coordinate the physiological cycles [2;3]. Most circadian gene expression is organ specific and in the mouse genome up to half of all genes oscillate according to a circadian rhythm somewhere in the organism [4]. In the pineal gland which is controlled by the SCN almost 60% of 4,459 genes exhibit significant day/night expression changes [5]. Proper functioning of the clock system requires an intricate interplay between transcriptional, post-transcriptional, and post-translational mechanisms [6]. Recently, several studies have shown that circadian oscillations of protein expression (involving phases, periods, and amplitudes) to a much larger extent than previously envisioned are controlled post-transcriptionally [7;8]. As an example, in the murine hepatic proteome about 20–50% of the transcripts representing 5–10% of total protein were not cyclic despite cyclic protein levels [9–11]. Also, next-generation sequencing studies indicate that 80% of clock-controlled genes are not rhythmically transcribed [12]. Post-transcriptional circadian regulation could, among other factors, be due to micro-RNA (miRNA) [13–16], and in one example involving miRNAs interacting with clock genes *Bmal1* and *Clock* in mice, miR-219 and miR-132 were demonstrated to be clock gene and light-regulated, respectively [17]. Also, circulating miRNAs in mice interacting with *Bmal1* were shown to be rhythmically fluctuating suggesting that they act as extracellular signals regulating peripheral circadian oscillators [18]. In addition, other noncoding RNAs are rhythmic in specific tissues [19], and because a single miRNA has many potential mRNA targets, one fluctuating miRNA may orchestrate fluctuations of many different proteins simultaneously. However, while several miRNAs and indeed the pre-miRNA processing machinery (Dicer RNase) are rhythmic in specific tissues such as the SCN, the eyes, and liver [8;20] and may have circadian functions [21], it is not known if any of the circulating miRNA in human blood exhibits diurnal variation. It is also not known if any oscillating circulating, cell-free miRNA would function as a regulator of peripheral circadian oscillators and/or simply is a reflection of changing miRNA levels at the sites of central and peripheral oscillators. Yet another possibility is that some miRNA oscillations may occur because the miRNAs are transcribed together with oscillating protein-coding RNA. In any event, no studies of the physiological rhythmicity of extracellular miRNAs in the human circulation have been presented. This is important also because the circulating pool of miRNA is under scrutiny as a convenient reservoir of information on disease and functions in numerous conditions and diseases. Here, we study a panel of 92 miRNAs in plasma from healthy individuals. The miRNA panel includes miRNAs that are expressed in the pineal gland or have been reported as rhythmically expressed or as implicated in regulating oscillatory regulation of pineal gland/retinal gene expression in animal studies [6;8;17;22–27] or have been predicted from literature and bioinformatics screening and shown in experiments with cellular miRNA to fluctuate [28;29]. Also, we include miRNAs that are known to be detectable in plasma or are abundant in the central nervous system [30;31]. This panel of miRNAs is here quantitatively determined in plasma samples obtained from a group of 24 healthy young men at regular 3-hr intervals in a controlled environment during 24 hours. Of the 79 miRNA that could be reliably measured we find n = 26 that are significantly rhythmic and which represent two main phase patterns. This is the first report of diurnal variations of circulating, extracellular human miRNAs. The findings add another important intra-individual variable to consider in the design and interpretation of biomarker development studies and suggest that investigations of functional roles of these miRNAs in the regulation of biological clocks are warranted.

## Materials and Methods

### Study design

In the present study, samples from the Bispebjerg study of diurnal variations [32] were used to analyze variations of circulating cell-free micro RNA. In brief, 24 healthy Caucasian male volunteers (mean age 26 years) stayed for 24 hours at the hospital ward with 15 hours of wakefulness in ordinary day/room light (mean light intensity 219 lux) and 9 hours of sleep from 23:00 h to 08:00 h in the dark. Every third hour (nine time points in total), each participant had blood samples taken; sampling during the night was guided by red light (19 lux). During the awake period, the participants were allowed to carry out low intensity activities and standardized normal calorie meals were served at 09:30 h, 13:00 h and 19:00 h. The volunteers’ regular routine of diurnal activity and nocturnal sleep were validated by the presence of a normal 24-hour melatonin pattern [32]. All participants signed an informed written consent before inclusion and the study was conducted according to the Helsinki declaration of 1975, as revised in 1983. The Regional Scientific Ethical Committee of the Capital Region of Denmark (protocol number H-B-2008-011) and the Danish Data Protection Agency (journal number 2008-41-1821) approved the study.

### Analysis of circulating microRNA

A total of 216 plasma samples representing the 9 time points from the 24 study subjects were included. Blood samples for analysis of miRNA were drawn in K_3_EDTA (ethylene diamine tetraacetic acid) plasma tubes (Greiner Bio-one, Frickenhausen, Germany). Immediately after sampling plasma was isolated by centrifugation at 1,500 *g* at 20°C for 10 minutes and stored at −80°C until analysis.

#### RNA purification

A total RNA purification kit (Norgen Biotek Corp., Ontario, Canada) was used to purify RNA from 100 μL plasma according to the instructions of the manufacturer with small modifications: 10 mM dithiothreitol (DTT) (Sigma-Aldrich Co. LLC, Germany) and 1.7 pM synthetic *C*. *elegans* miR-54 and -238 (Tag Copenhagen A/S, Denmark) were added into lysis buffer. One uL of RNAse inhibitor (20 U/μL) (Applied Biosystems (ABI), Foster city, CA, USA) was added to every elution tube before elution of RNA. Purified RNA samples were kept at -20°C until used.

#### Reverse transcription

Reverse transcription (RT) was performed by the TaqMan microRNA Reverse Transcription Kit (Applied Biosystems (ABI), Foster city, CA, USA) according to the instructions of the manufacturer with modifications: The RT-primer-mix consisted of equal volumes of each of 48 different 5x RT miR-specific stem-loop primers (Applied Biosystems (ABI), Foster city, CA, USA). Each reverse transcription reaction volume was 10 μL using 1 μL Multiscribe reverse transcriptase, 3 μl RT-primer-mix, 1 μL 10 X buffer, 0.2 μL 100 mM dNTPs, 0.15 μL RNase inhibitor and 4.65 μL RNA purified from plasma. Reverse transcription was performed on an ABI 2720 Thermal Cycler (Applied Biosystems, Foster City, CA, USA) using a standard protocol (16°C, 30 min; 42°C, 30 min; 85°C 5 min; hold at 4°C). Reverse transcribed samples (cDNA) were kept at -20°C until used. The same procedure was used for a second set of miRNAs (S1 Table).

#### Preamplification

For each of the 2 sets of analyses that were performed (*cf*. S1 Table) specific target amplification of the cDNA was accomplished using the TaqMan PreAmp master mix (Applied Biosystems (ABI), Foster city, CA, USA) with a mix of the TaqMan MicroRNA Assays (ABI) consisting of equal volumes of the 48 different 20x assays diluted with 1x TE buffer to a final concentration of 0.2x. Preamplification mixtures (10 μL) contained cDNA (diluted 1:3 with H_2_O) 2.5 μL, mixed with 5 μL 2x TaqMan PreAmp master mix and 2.5 μL of the 0.2x TaqMan miR-assay mix. Preamplification was performed on an ABI 2720 Thermal Cycler (Applied Biosystems, Foster City, CA, USA) with the following program steps: 95°C for 10 min, 16 cycles of 95°C, 15 s and 60°C, 4 min, then hold at 4°C. Preamplified samples were kept at -20°C until used.

#### Quantitative PCR

Preamplified samples (diluted 1:5 with H_2_O) and TaqMan 20x assays miRNAs were applied to primed 96.96 dynamic array chips using loading and assay reagents according to the manufacturer (Fluidigm Corp., USA). All miR-assays were performed in duplicate. The samples of this study were analyzed on three 96x96 chips (Fluidigm Corp., USA) with two different 48-miRNA assay panels (for a total of 6 chips). After loading the reaction chambers using the integrated fluid circuit (IFC) HX controller from Fluidigm, the real-time PCR including image capture after each cycle was performed in a BioMark real-time PCR system (Fluidigm Corp., USA) using single probe (FAM-MGB, reference: ROX) settings and GE 96x96 standard v1 protocol with 40 cycles. Data processing took place using the Fluidigm real-time PCR analysis software (v. 4.1.2).

#### Data handling

The “auto detectors” setting was chosen for data from all of the chip runs. Data with average Cq values above 35 were excluded from data sets. Each remaining average Cq value was technically normalized with the average Cq of the spike-in synthetic miRNAs (cel-miR-54 and cel-miR-238) for that particular sample yielding the -ΔCq values (= average Cq of cel-miR-54 and cel-miR-238 − average Cq of the hsa-miR). This technical normalization is included to level out variations during the purification steps. These -ΔCq values were further normalized with the row-specific average ΔCq value of miRNAs that were detected in all samples to correct for variations in total input RNA. Thus, we used the average -ΔCq of 40 miRNAs in the first 48-miRNA panel and of 20 miRNAs in the second 48-miRNA panel (S1 Table) for each row to subtract from all miRNA-ΔCq values in each sample (row). These technically and row mean-normalized expression values were then used for the rhythmicity analysis. The normalized miRNA data are shown in S2 Table. Additionally, for the first miRNA panel (where miR-16 was included), we also normalized the data with row-specific miR-16 values instead of using row mean-normalization (data included in S3 Table).

### Rhythmicity analysis

As described earlier [32], under the assumption of 24-hour periods, the time-related miRNA expression data for the complete set of 216 results for each miRNA were fitted into a combined cosine and sine function and analyzed for possible diurnal rhythms. The methods for cosinor-rhythmometry for groups including the *p*-value calculation after linearization correspond to previously published methods [33]. The 24-hour rhythms were characterized by the rhythm parameters: mesor (rhythm-adjusted average about which oscillation occurs), amplitude (the difference between the peak (zenith) and lowest (nadir) value of the fitted cosinor curve) and time of peak and nadir [33]. Whether a parameter exhibited a clear diurnal rhythm was evaluated from the *p*-value and the shape of the cosine curve. The time-dependent distribution of the individual observations of the miRNAs presenting diurnal rhythms was graphically summarized as the best fitting cosine curve against time. At each time point, mean values and standard errors of the mean are shown. The data analyses were performed using SAS version 9.3 (SAS Institute Inc, Cary, NC, USA). A *p*-value < 0.05 was considered significant. All data, including graphs of all detected miRNAs (S1 Fig) and the rhythmicity analysis data (S1 and S2 Files) are included as Supporting Information.

### MiRNA target analysis

Analysis of individual miRNA targets was based on miRTarBase 6.0 (http://mirtarbase.mbc.nctu.edu.tw/). The miRNAs were also investigated for targets in known central clock genes (*CRY1/2*, *PER1/2/3*) and in the MAPK pathway. For MAPK, all the genes in this pathway were identified through the KEGG homepage (in total 225 genes) and compared to all stringently validated genes (607 unique genes) regulated by the miRNAs found in the study (validated by 2 or more of the following methods: reporter assay, Western blot, or qPCR). Pathway representation of miRNA targets was accomplished using the KEGG pathway database (http://www.genome.jp/kegg-bin/show_pathway?org_name=hsa&mapno=04010&mapscale=&show_description=hide), target genes were marked in red, using the “User data mapping” function.

The miRNA targets were mapped to KEGG and Reactome pathways using the program gProfileR (v. 0.5.3) through R (v. 3.1.3). Organism was set to “hsapiens” and significant to “TRUE”, otherwise default settings were used. The *p*-values obtained from gProfileR were used as values for heatmaps and all miRNA-pathway combinations that were not significant were assigned the value 0.05 for visualization. Heatmaps were made using the gplots package (vs. 2.17.0), trace was disabled, otherwise default settings were used.

## Results and Discussion

### Diurnal fluctuations of plasma miRNAs in healthy volunteers

A normal 24-hour rhythm of the participants was confirmed by analyzing for melatonin that exhibited a clear rhythm (*p* < 0.0001) with an amplitude (amp) of 19.84 pg/ml and a nightly peak level at 03:34 h in the study samples as previously reported [32].

We found 79 of the assayed 92 miRNAs to be reliably detected. Excluded miRNAs either gave no or weak (Cq > 35) signal or only signals in a few (less than half) of the samples. To date there are no universally accepted circulating miRNAs that can be used as normalizing housekeeping genes and the concentration of the purified miRNA is too low to be determined UV spectrophotometry. Data were therefore normalized in two steps. First, normalization for technical variation was accomplished by using the values of spike-in synthetic non-mammalian miRNA. Second, normalization for variations in input total miRNA was accomplished by row mean-normalization [34] using the mean Cq value in each row (sample) of miRNAs expressed in all samples. Specifically, the row mean of 40 miRNAs in the first panel-experiment and of 20 miRNAs in the second panel-experiment were used for normalization.

**Fig 1:**
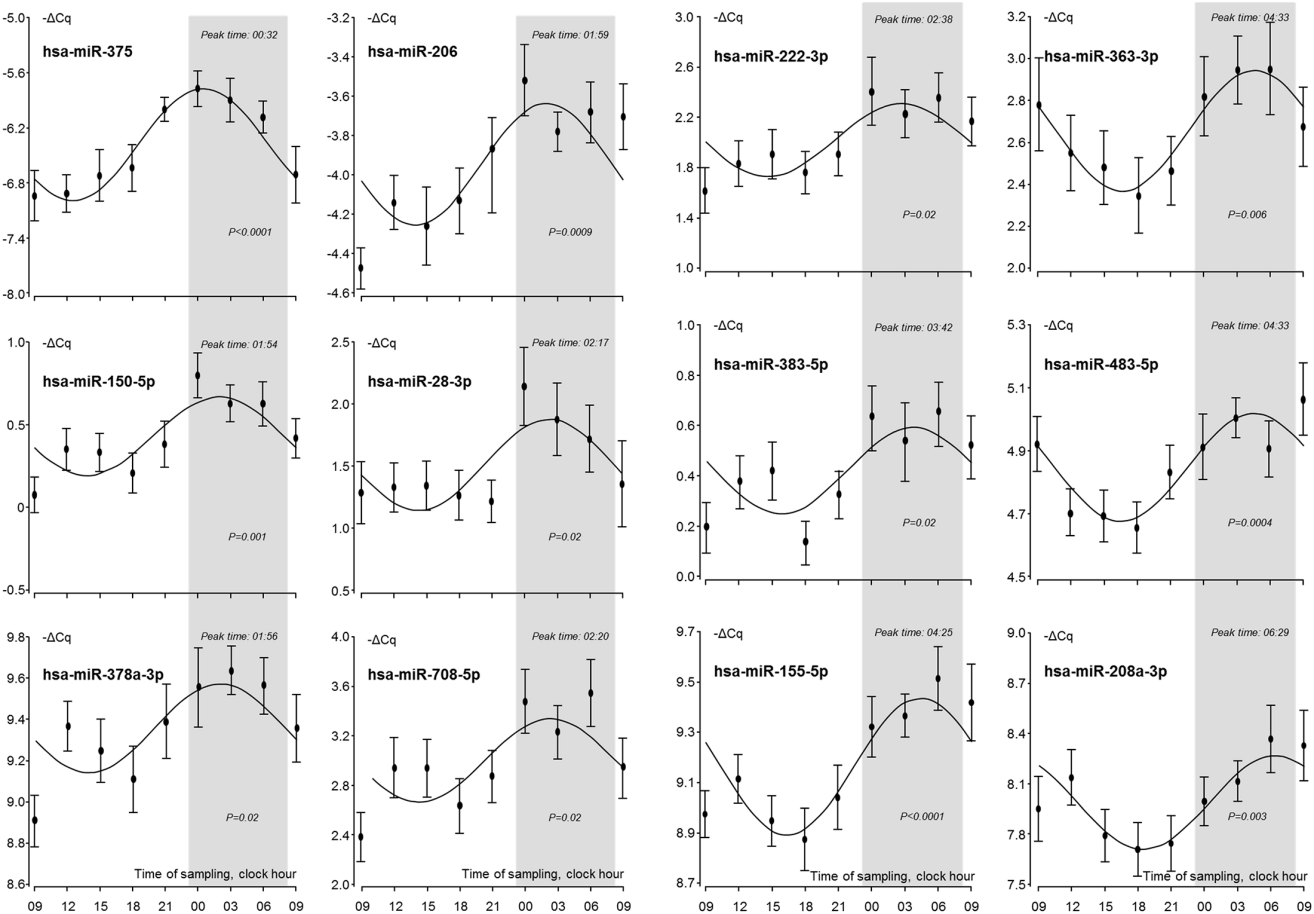
Nocturnal miRNAs. Sleep periods are indicated in grey. Individual points represent the mean and SEM of the values of the 24 test individuals.

**Fig 2:**
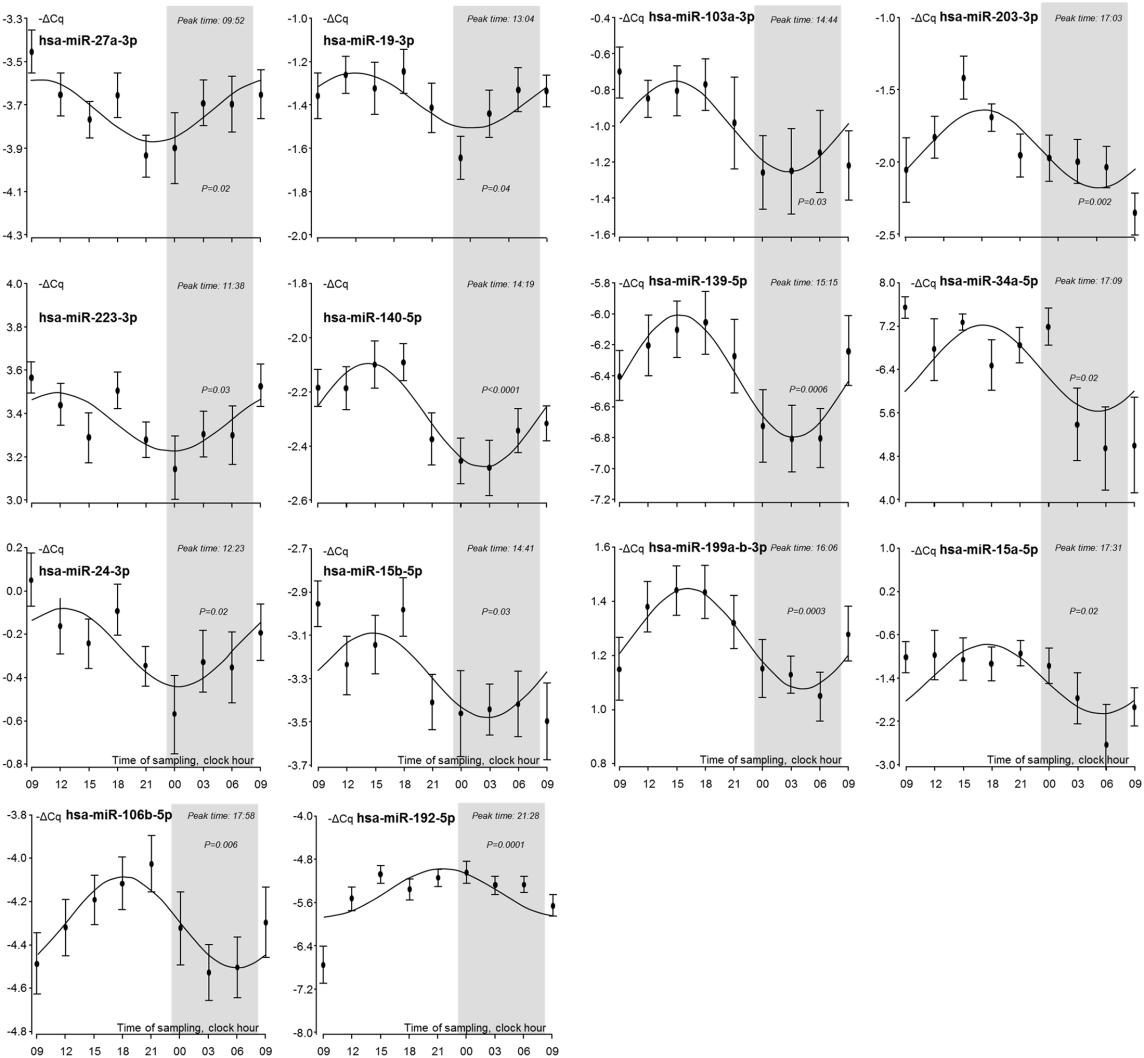
Diurnal miRNAs. Sleep periods are indicated in grey. Individual points represent the mean and SEM of the values of the 24 test individuals.

Of the 79 detected miRNAs 26 were significantly (*p* < 0.05) rhythmic (Figs 1 and 2; in S1 Fig is included all the graphs of the 79 detected miRNAs). The time-of-day dependent distribution of the individual observations of the miRNAs presenting diurnal rhythms is listed in Table 1. In the graphs the sleep periods are indicated in grey and miRNAs peaking at night (n = 12, Fig 1) and during day (n = 14, Fig 2) are shown in the respective figures.

**Table 1:** Rhythmic, circulating miRNAs (*p* < 0.05) ranked according to their peak time (Max time).

miRNA	Mesor	Amplitude	Max time	Min time	P-value
hsa-miR-375	-6.4	1.2	00:32	12:32	*<0*.*0001*
hsa-miR-150-5p	0.4	0.5	01:54	13:54	*0*.*001*
hsa-miR-378a-3p	9.4	0.4	01:56	13:56	*0*.*02*
hsa-miR-206	-4.0	0.6	01:59	13:59	*0*.*0009*
hsa-miR-28-3p	1.5	0.7	02:17	14:17	*0*.*02*
hsa-miR-708-5p	3.0	0.7	02:20	14:20	*0*.*02*
hsa-miR-222-3p	2.0	0.6	02:38	14:38	*0*.*02*
hsa-miR-383-5p	0.4	0.3	03:42	13:42	*0*.*02*
hsa-miR-155-5p	9.2	0.5	04:25	16:25	*<0*.*0001*
hsa-miR-363-3p	2.7	0.6	04:33	16:33	*0*.*006*
hsa-miR-483-5p	4.8	0.3	04:33	16:33	*0*.*0004*
hsa-miR-208a-3p	8.0	0.6	06:29	18:29	*0*.*003*
hsa-miR-27a-3p	-3.7	0.3	09:52	21:52	*0*.*02*
hsa-miR-223-3p	3.4	0.3	11:38	23:38	*0*.*02*
hsa-miR-24-3p	-0.3	0.4	12:23	00:23	*0*.*02*
hsa-miR-19-3p	-1.4	0.3	13:04	01:04	*0*.*04*
hsa-miR-140-5p	-2.3	0.4	14:19	02:19	*<0*.*0001*
hsa-miR-15b-5p	-3.3	0.4	14:41	02:41	*0*.*03*
hsa-miR-103a-3p	-1.0	0.5	14:44	02:44	*0*.*03*
hsa-miR-139-5p	-6.4	0.8	15:15	03:15	*0*.*0006*
hsa-miR-199a/b-3p	1.3	0.4	16:06	04:06	*0*.*0003*
hsa-miR-203-3p	-1.9	0.5	17:03	05:03	*0*.*002*
hsa-miR-34a-5p	6.4	1.6	17:09	05:09	*0*.*02*
hsa-miR-15a-5p	-1.4	1.3	17:31	05:31	*0*.*02*
hsa-miR-106b-5p	-4.3	0.4	17:58	05:58	*0*.*006*
hsa-miR-192-5p	-5.4	0.9	21:28	09:28	*0*.*0001*

As an alternative to row mean-normalization it might be possible to use single miRNAs as endogenous controls in the same way as small nucleolar RNAs are used in tissue and cell-miRNA profiling. In extensive method comparisons between serum and plasma, miR-16 emerged as one possible candidate for such an abundant, endogenous circulating miRNA normalizer [35]. Other studies, however, have not found miR-16 to be appropriate for this purpose. One reason is that it is associated with at least 10 non-neoplastic diseases [36] and is differently reported as a stable and a disease-associated circulating miRNA even in studies of the same disease [37]. Also, no studies have addressed the rhythmicity of miR-16 itself in the circulation. Here, we find it quite stable (S2 Fig) during the 24-hour period. The two normalization methods (cf. side-by-side comparison in S3 Fig) yield overall quite similar time-curves, but miR-16 normalization leads to less significant miRNAs found in the rhythmicity analysis, probably due to the more pronounced variation of the ΔCq-values at each time point. Thus, when miR-16 was used for normalization instead of row mean-normalization of the first miRNA panel data, a total of 9 miRNAs, all part of the miRNAs emerging as significantly rhythmic after row mean-normalization, were confirmed. However, 9 others were not found (S1 Table) and in view of this and of the lack of consensus regarding single-miRNA normalizers we decided to use the row mean-normalization method throughout.

We compared our findings with mammalian miRNAs bioinformatically predicted to target clock genes, their isoforms, and genes associated with a pattern of circadian protein expression despite constitutively expressed mRNA [28]. In the present study we included 17 of the 38 miRNAs equally predicted by three different miRNA-target prediction programs and with validated rhythmic target genes. Of these 17 miRNAs, 5 (miRs-9, -29b, -96, -219, and -346) were not reliably detected and 10 miRNAs did not exhibit diurnal oscillation while 2 (miRs-24 and -27a) did show a significant diurnal variation. Even though we do not find a significant diurnal variation of miR-27b (*p* = 0.08) our data show that the closely related miR-27a (one nucleotide difference) does exhibit rhythmicity (*p* = 0.02). In leukocytes miR-27b was previously shown to display rhythmicity in samples from two out of five normal subjects [28]. In another study of fluctuations of leukocyte miRNAs [29] miR-16 and miR-181a were shown to fluctuate in those 4 out of 6 subjects where the expression was highest at daytime. In our data, representing the mean of 24 healthy volunteers, we do not find a concurrent rhythmic variation of these two miRNAs. It is highly warranted in future studies to address the rhythmicity of the miRNA precursors (pri-miRs) in specific cell types, *e*.*g*. leukocytes to address if miRNA rhythmicity is directly transcriptionally regulated.

The human homologues of mouse miRNAs -219a and -132 which exhibit rhythmic expression in mouse brain tissue and act as clock-transcription factor and light-regulated miRNAs, respectively [17] were included in our study but miR-219a was found not to be detectable in plasma, and miR-132 had a stable, non-cyclic expression. Also, while we were able to detect miR-182 in plasma, it was not oscillating. Its pre-form has been reported to be modulating the circadian clock and was abnormally processed in a study of depressive patients with sleep disorders [24]. Regulation by other environmental factors such as *e*.*g*. food intake is possible, but fasting was previously found not to affect the levels of 162 circulating human miRNAs [38]. This study identified miR-99a-5p and miR-139-5p as novel endogenous controls for serum miRNA because of stable values across individuals. However, our data show that while the levels of miR-99a-5p are not rhythmic they are quite variable (with a difference of 21% between the highest and lowest mean values) during the 24-hour period. For miR-139-5p, the data in fact show clear rhythmicity (*p* = 0.0006). None of these two miRNAs therefore appears to be optimal as endogenous normalizers for plasma miRNAs.

### Target analysis of rhythmic plasma miRNAs

The presence of specific, circulating, cell-free miRNAs that display diurnal rhythmicity in plasma from healthy humans is a novel finding. The mechanisms behind the fluctuations are unknown. Thus, miRNA-rhythmicity may be due to oscillating transcription, fluctuating release from cells, fluctuating half-lives, and any combinations of these factors. The putative functional roles of circulating miRNA are unclear even though a number of studies show that protein expression may be affected by cell-free miRNA taken up by target cells [39;40]. For that reason the potential targets of the specific rhythmic miRNAs may be of interest even though the miRNAs identified here are not envisioned to be functional as translational repressors while present in the circulation but only after being taken up by target cells or tissues. With the caveat that this is not experimentally addressed in the present study and that most of the miRNAs in the circulation may in fact reflect the end result of cellular disposal we analyzed for targets in the circadian system and also for common targets using bioinformatics approaches.

In the case of miR-375 –a highly significantly fluctuating miRNA (*p* < 0.0001) there are a number of functionally validated targets of possible relevance including transcription factor *SP1* which is among the top 5 transcription factors associated with circadian clocks in mice [41]. *MECP2* (methyl CpG binding protein), a circadian-cycle dependent epigenetic regulator of gene expression in the brain [42] is also a validated target of miR-375. Another interesting target is *RASD1* which regulates responsiveness of the circadian clock and is rhythmically expressed in the SCN in mouse models [43]. Finally, miR-375 also targets *MYC* which competes with the *Clock-Bmal1* master circadian transcription factor for the same nucleotide sequence motifs in the genome [44]. The miR-483 (*p* = 0.0004) is a pineal miRNA and was shown in rats to target melatonin expression [22].

In mice, miR-155-5p (*p* < 0.0001) targets the *Bmal1* mRNA directly and is an important regulator of the innate immune response that is conditioned across the circadian day in myeloid cells [45]. Human miR-155 is homologous to mouse miR-155 (only one nucleotide substitution) and therefore likely to be functionally similar. This proinflammatory miRNA is also found in lymphocytes and since lymphocyte counts display rhythmicity, *i*.*e*. a decline in the morning and a peak during night [2;46;47], the miR-155 fluctuations observed in plasma may, at least partly, reflect the physiologically variable lymphocyte counts. Both miR-155-5p and miR-206 (*p* = 0.0009) target *CCND1*, a cell cycle marker that is timed by clock proteins in the mouse [48]. Also, miR-155-5p targets the *CEBPB* transcription factor which controls practically all members of the core circadian clock system [49].

miR-208a-3p (*p* = 0.003) has *Sox-6* as one of its validated targets (as does miR-155-5p) and *Sox-6* expression is negatively correlated with *Per1* expression. *Per1* is a key circadian gene in the mouse involved in a negative feed-back loop with the *Clock* transcription factor [41]. Another miR-208a-3p target, *CDKN1A* (or p21), is a cyclin-dependent kinase inhibitor controlling G1 cell cycle progression and is circadian in a number of mouse tissues [41]. It is also a validated target of two other clearly rhythmic miRNAs in the present study, *i*.*e*., miR-363-3p (*p* = 0.006) and miR-106b-5p (*p* = 0.006). Finally, the transcription factor est-1, which is yet another functionally validated miR-208a target, regulates many circadian genes in the SCN and is down-regulated during the day and upregulated during the night in rats [50].

MiR-139-5p (*p* = 0.0006) targets *IGF1R* which together with *PDK1* (a kinase downstream of *IGF1R* (both *IGF1R* and *PDK1* are targeted by miR-375)) has been shown to be induced by light in a study of the coupling of cell proliferation with diurnal/circadian cycles in a human breast cancer model [51]. It also targets *ROCK2* that oscillates under the control of the *RORα* clock gene, and thus regulates differences in myosin light chain phosphorylation leading to rhythmicity in the contractility of the vascular system [52].

miR-106b-5p (*p* = 0.006) targets *CDKN1A* (p21) as mentioned above in addition to *PTEN* which appears to be involved in the maintenance of circadian rhythm in mouse models [53]. In addition, p21 is a target of p53 which is targeted by both miR-375 (*p* = 0.0001) and miR-150-5p (*p* = 0.001). In turn, p21 effects the phosphorylation of Rb1, another validated target of miR-106-5p, and thereby affects cell cycling.

Transcripts of a number of genes involved in epigenetic regulation (acetyltransferases, deacetylases, and DNA methyltransferases (DNMT1, HDAC4, HDAC7, KAT2B, MECP2)) are strongly validated targets of the highly significant rhythmic miRNAs found in the present study. Thus, miR-140-5p (*p* < 0.0001) targets *HDAC4* as well as *HDAC7*, both histone class II deacetylases which regulate transcription and are well established circadian regulators [54]. Another epigenetic clock regulator, *DNMT1*, is also a miR-140-5p target [1]. Finally, a histone acetyltransferase, *KAT2B* which is a circadian transcriptional co-activator of activators of clock genes [55] is targeted by miR-106b-5p.

#### Systems analysis of rhythmic circulating miRNAs and their targets

Analysis of core clock gene mRNA sequences reveal that the *CRY2* mRNA has two weakly interacting miRNAs among the 26 significantly fluctuating miRNAs found in this study, *i*.*e*., miR-106-5p and miR-24-3p while none targets *CRY1*. For the *Per* genes we find that *Per1* is a weakly predicted target for 2 of the fluctuating plasma miRNAs, miR-28-3p and miR-103a-3p, discovered in the present study while *Per2* is predicted to interact weakly with miR-24-3p and miR-363-3p. None of the fluctuating miRNAs are predicted to target *Per3*.

**Fig 3:**
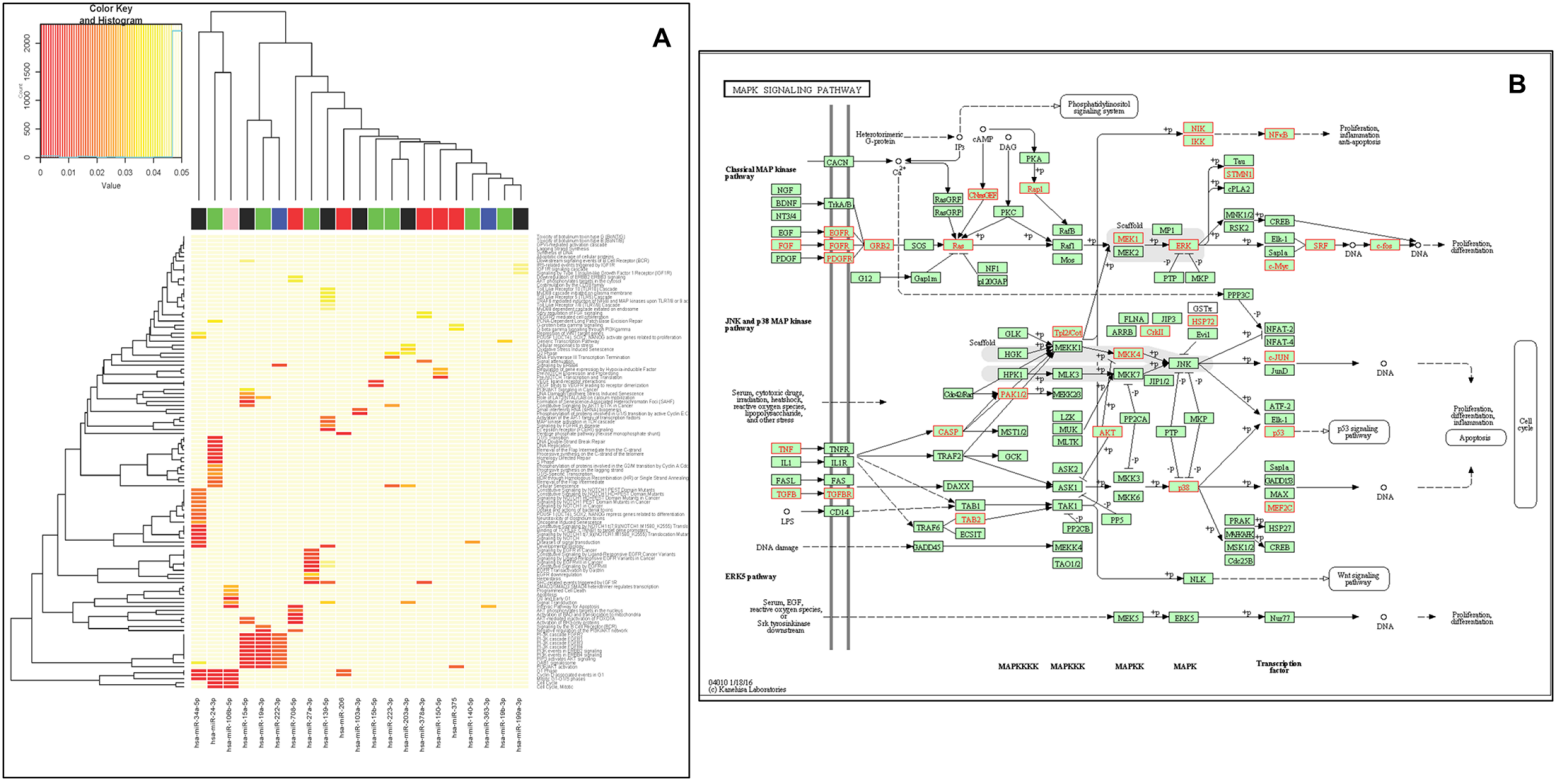
**A, Heatmap showing overrepresented Reactome pathways.** For each miRNA all strong targets (defined by miRTarBase) was used for GO analysis using gProfileR. Only miRNAs where target genes were significantly overrepresented in one or more pathways are shown. Colors indicate *p*-value with red colors being associated with lower *p*-values. Non-significant matches were all assigned the value 0.05. The color bar on top of the plot indicates groups belonging into 5 time-point specific expression groups (group 1: red, group 2: blue, group 3: green, group 4: black, group 5: pink). **B, MAPK pathway targets.** Strongly binding predicted MAPK pathway targets of significant rhythmic miRNAs of the present study are colored red.

When including the target genes of the 29 miRNAs (for miRNAs 19-3p, 199-3p, and 203-3p we used a and b as separate miRNAs) in the pathway analyses, no compelling clustering is visualized except that cell cycling genes and fibroblast and epidermal growth factor receptor-associated intracellular kinase cascades are shared as targets of a number (> 2) of the cyclic miRNAs (Fig 3A). Looking at genes of relevance for intracellular signaling we find that a total of 38 genes (*RAPGEF*, *RAP1B*, *FGF7*, *FGF9*, *EGFR*, *FGFR3*, *PDGFRA*, *PDGFRB*, *GRB2*, *HRAS*, *KRAS*, *MAP2K1*, *MAPK1*, *MAPK3*, *MYC*, *SRF*, *FOS*, *STMN1*, *TNF*, *TGFB1*, *TGFBR1*, *CASP3*, *TAB2*, *PAK2*, *MAP3K8*, *MAP2K4*, *CRKL*, *MAPK14*, *JUN*, *TP53*, *MEF2C*, *AKT1*, *AKT2*, *AKT3*, *HSPA1B*, *MAP3K14*, *CHUK*, *NFKB1*) are both among the strong evidence targets of the cyclic miRNAs as well as situated in the MAPK pathway. These genes are marked with red in Fig 3B.

## Conclusions

The present study identifies circulating miRNAs that undergo clear rhythmic fluctuations in abundance during a 24-hour period in a group of 24 healthy young male individuals. The findings are specific in the sense that only a third (26/79) of the detected miRNAs display rhythmicity and in the sense that two different main phase patterns are observed—one peaking during night and one pattern peaking during day. Despite the challenge of normalizing data of circulating miRNA abundance due to the lack of accepted “housekeeping” circulating miRNAs [34] these observations argue against variations in sampling and RNA purification conditions leading to the observed differences. Thus, this is, to our knowledge, the first time diurnal variations in the levels of human circulating, cell-free miRNAs have been reported. The present study is merely descriptive and does not give a basis for a unifying understanding of the roles of the individual circadian miRNAs especially because of the general target promiscuity of miRNAs and because of the uncertainties regarding the regulation of miRNA synthesis, release to the circulation, and regulation of circulating miRNA removal and degradation. Indeed, a recent report showed that only 7% (4/57) mature miRNAs originating from oscillating primary transcripts were rhythmically expressed [21]. Future work involves cellular and animal models with targeted miRNA disruption and induction. Also, a confirmative analysis over more than one diurnal cycle would be valuable.

In any event, the finding that some miRNAs vary according to a diurnal clock is important information for all studies that include analysis of the levels of circulating miRNAs and the findings also constitute a starting point for further studies of the importance of miRNA oscillations in physiology and disease.

## Supporting Information

S1 Fig Graphs of all detected miRNA ΔCq values (V1) as a function of time (hours after first sample at 09 am). Individual points represent the mean and SEM of the values of the 24 individuals included in the study. In case of significant rhythmicity, the p-values are included on the graphs.
(PPTX)

S2 Fig Representations of miR-16 data. Individual data points, mean and SEM are shown for row mean-normalized, technically normalized, and raw (not normalized) miR-16 data.
(PPTX)

S3 Fig Graphs of miRNAs detected in the first panel. Comparison side-by-side of row mean-normalized with row miR-16 normalized data. Data points (mean and SEM) and curve fitting are shown.
(PPTX)

S1 File Rhytmicity analysis of detected miRNAs by cosinor-rhythmometry using row mean normalized data. The p-values are indicated in red.
(DOCX)

S2 File Rhytmicity analysis of detected miRNAs by cosinor-rhythmometry using miR-16 normalized data. The p-values are indicated in red.
(DOCX)

S1 Table The two miRNA panels employed in this study. Designations (assay name, ID and miRBase name) and sequences are included. Also indicated are excluded miRNAs based on no signal (red background) or too few data (light blue background). For the first panel where data were normalized using both miR-16 and row mean normalization the miRNA in red are those that were significantly rhythmic using the row mean normalized data only while those that are written in green are those that were significantly rhythmic using both normalization methods.
(XLSX)

S2 Table Data file panel 1 and 2. Row mean normalized data for both miRNA panels.
(XLSX)

S3 Table Data file, panel 1. miR-16 normalized data.
(XLSX)

## References

[1] Azzi A, Dallmann R, Casserly A, Rehrauer H, Patrignani A, Maier B, et al: Circadian behavior is light-reprogrammed by plastic DNA methylation. Nat Neurosci 2014;17:377–382. 10.1038/nn.3651 24531307

[2] Hastings MH, Reddy AB, Maywood ES: A clockwork web: circadian timing in brain and periphery, in health and disease. Nat Rev Neurosci 2003;4:649–661. 12894240 10.1038/nrn1177

[3] Lowrey PL, Takahashi JS: Mammalian circadian biology: elucidating genome-wide levels of temporal organization. Annu Rev Genomics Hum Genet 2004;5:407–41.:407–441. 15485355 10.1146/annurev.genom.5.061903.175925 PMC3770722

[4] Zhang R, Lahens NF, Ballance HI, Hughes ME, Hogenesch JB: A circadian gene expression atlas in mammals: implications for biology and medicine. Proc Natl Acad Sci U S A 2014;111:16219–16224. 10.1073/pnas.1408886111 25349387 PMC4234565

[5] Bailey MJ, Coon SL, Carter DA, Humphries A, Kim JS, Shi Q, et al: Night/day changes in pineal expression of >600 genes: central role of adrenergic/cAMP signaling. J Biol Chem 2009;284:7606–7622. 10.1074/jbc.M808394200 19103603 PMC2658055

[6] Kojima S, Shingle DL, Green CB: Post-transcriptional control of circadian rhythms. J Cell Sci 2011;124:311–320. 10.1242/jcs.065771 21242310 PMC3021995

[7] Robles MS, Cox J, Mann M: In-vivo quantitative proteomics reveals a key contribution of post-transcriptional mechanisms to the circadian regulation of liver metabolism. PLoS Genet 2014;10:e1004047 10.1371/journal.pgen.1004047 24391516 PMC3879213

[8] Kojima S, Green CB: Circadian genomics reveal a role for post-transcriptional regulation in mammals. Biochemistry 2015;54:124–133. 10.1021/bi500707c 25303020 PMC4302021

[9] Reddy AB, Karp NA, Maywood ES, Sage EA, Deery M, O'Neill JS, et al: Circadian orchestration of the hepatic proteome. Curr Biol 2006;16:1107–1115. 16753565 10.1016/j.cub.2006.04.026

[10] Vollmers C, Schmitz RJ, Nathanson J, Yeo G, Ecker JR, Panda S: Circadian oscillations of protein-coding and regulatory RNAs in a highly dynamic mammalian liver epigenome. Cell Metab 2012;16:833–845. 10.1016/j.cmet.2012.11.004 23217262 PMC3541940

[11] Du NH, Arpat AB, De MM, Gatfield D: MicroRNAs shape circadian hepatic gene expression on a transcriptome-wide scale. Elife 2014;3:e02510 10.7554/eLife.02510.:e02510 24867642 PMC4032493

[12] Koike N, Yoo SH, Huang HC, Kumar V, Lee C, Kim TK, et al: Transcriptional architecture and chromatin landscape of the core circadian clock in mammals. Science 2012;%19;338:349–354. 10.1126/science.1226339 22936566 PMC3694775

[13] Pegoraro M, Tauber E: The role of microRNAs (miRNA) in circadian rhythmicity. J Genet 2008;87:505–511. 19147939 10.1007/s12041-008-0073-8

[14] O'Neill JS, Hastings MH: Circadian clocks: timely interference by microRNAs. Curr Biol 2007;17:R760–R762. 17803925 10.1016/j.cub.2007.07.001

[15] Lim C, Allada R: Emerging roles for post-transcriptional regulation in circadian clocks. Nat Neurosci 2013;16:1544–1550. 10.1038/nn.3543 24165681

[16] Chen R, D'Alessandro M, Lee C: miRNAs are required for generating a time delay critical for the circadian oscillator. Curr Biol 2013;23:1959–1968. 10.1016/j.cub.2013.08.005 24094851 PMC3809330

[17] Cheng HY, Papp JW, Varlamova O, Dziema H, Russell B, Curfman JP, et al: microRNA modulation of circadian-clock period and entrainment. Neuron 2007;54:813–829. 17553428 10.1016/j.neuron.2007.05.017 PMC2590749

[18] Shende VR, Goldrick MM, Ramani S, Earnest DJ: Expression and rhythmic modulation of circulating microRNAs targeting the clock gene Bmal1 in mice. PLoS ONE 2011;6:e22586 10.1371/journal.pone.0022586 21799909 PMC3142187

[19] Coon SL, Munson PJ, Cherukuri PF, Sugden D, Rath MF, Moller M, et al: Circadian changes in long noncoding RNAs in the pineal gland. Proc Natl Acad Sci U S A 2012;109:13319–13324. 10.1073/pnas.1207748109 22864914 PMC3421215

[20] Yan Y, Salazar TE, Dominguez JM, Nguyen DV, Li CS, Bhatwadekar AD, et al: Dicer expression exhibits a tissue-specific diurnal pattern that is lost during aging and in diabetes. PLoS ONE 2013;8:e80029 10.1371/journal.pone.0080029 24244599 PMC3820540

[21] Wang H, Fan Z, Zhao M, Li J, Lu M, Liu W, et al: Oscillating primary transcripts harbor miRNAs with circadian functions. Sci Rep 2016;6:21598 10.1038/srep21598 26898952 PMC4761921

[22] Clokie SJ, Lau P, Kim HH, Coon SL, Klein DC: MicroRNAs in the pineal gland: miR-483 regulates melatonin synthesis by targeting arylalkylamine N-acetyltransferase. J Biol Chem 2012;287:25312–25324. 22908386 10.1074/jbc.M112.356733 PMC3408182

[23] Xu S, Witmer PD, Lumayag S, Kovacs B, Valle D: MicroRNA (miRNA) transcriptome of mouse retina and identification of a sensory organ-specific miRNA cluster. J Biol Chem 2007;282:25053–25066. 17597072 10.1074/jbc.M700501200

[24] Saus E, Soria V, Escaramis G, Vivarelli F, Crespo JM, Kagerbauer B, et al: Genetic variants and abnormal processing of pre-miR-182, a circadian clock modulator, in major depression patients with late insomnia. Hum Mol Genet 2010;19:4017–4025. 10.1093/hmg/ddq316 20656788

[25] Alvarez-Saavedra M, Antoun G, Yanagiya A, Oliva-Hernandez R, Cornejo-Palma D, Perez-Iratxeta C, et al: miRNA-132 orchestrates chromatin remodeling and translational control of the circadian clock. Hum Mol Genet 2011;20:731–751. 10.1093/hmg/ddq519 21118894 PMC3115667

[26] Balakrishnan A, Stearns AT, Park PJ, Dreyfuss JM, Ashley SW, Rhoads DB, et al: MicroRNA mir-16 is anti-proliferative in enterocytes and exhibits diurnal rhythmicity in intestinal crypts. Exp Cell Res 2010;316:3512–3521. 10.1016/j.yexcr.2010.07.007 20633552 PMC2976799

[27] Tan X, Zhang P, Zhou L, Yin B, Pan H, Peng X: Clock-controlled mir-142-3p can target its activator, Bmal1. BMC Mol Biol 2012;13:27 10.1186/1471-2199-13-27 22958478 PMC3482555

[28] Figueredo DS, Barbosa MR, Gitai DL, de Andrade TG: Predicted microRNAs for mammalian circadian rhythms. J Biol Rhythms 2013;28:107–116. 10.1177/0748730413476827 23606610

[29] Figueredo DS, Gitai DL, Andrade TG: Daily variations in the expression of miR-16 and miR-181a in human leukocytes. Blood Cells Mol Dis 2015;54:364–368. 10.1016/j.bcmd.2015.01.004 25641414

[30] Cheng HH, Yi HS, Kim Y, Kroh EM, Chien JW, Eaton KD, et al: Plasma Processing Conditions Substantially Influence Circulating microRNA Biomarker Levels. PLoS ONE 2013;8:e64795 10.1371/journal.pone.0064795 23762257 PMC3676411

[31] Sethi P, Lukiw WJ: Micro-RNA abundance and stability in human brain: specific alterations in Alzheimer's disease temporal lobe neocortex. Neurosci Lett 2009;459:100–104. 10.1016/j.neulet.2009.04.052 19406203

[32] Sennels HP, Jorgensen HL, Hansen AL, Goetze JP, Fahrenkrug J: Diurnal variation of hematology parameters in healthy young males: the Bispebjerg study of diurnal variations. Scand J Clin Lab Invest 2011;71:532–541. 10.3109/00365513.2011.602422 21988588

[33] Nelson W, Tong YL, Lee JK, Halberg F: Methods for cosinor-rhythmometry. Chronobiologia 1979;6:305–323. 548245

[34] Marabita F, de Candia P, Torri A, Tegnér J, Abrignani S, Rossi RL: Normalization of circulating microRNA expression data obtained by quantitative real-time RT-PCR. Briefings in Bioinformatics 2015; 1–9. 10.1093/bib/bbv056 PMC4793896 26238539

[35] Wang K, Yuan Y, Cho JH, McClarty S, Baxter D, Galas DJ: Comparing the MicroRNA spectrum between serum and plasma. PLoS ONE 2012;7:e41561 10.1371/journal.pone.0041561 22859996 PMC3409228

[36] Haider BA, Baras AS, McCall MN, Hertel JA, Cornish TC, Halushka MK: A critical evaluation of microRNA biomarkers in non-neoplastic disease. PLoS ONE 2014;9:e89565 10.1371/journal.pone.0089565 24586876 PMC3935874

[37] Witwer KW: Circulating microRNA biomarker studies: pitfalls and potential solutions. Clin Chem 2015;61:56–63. 10.1373/clinchem.2014.221341 25391989

[38] MacLellan SA, MacAulay C, Lam S, Garnis C: Pre-profiling factors influencing serum microRNA levels. BMC Clin Pathol 2014;14:27 10.1186/1472-6890-14-27 25093010 PMC4107491

[39] Ratajczak MZ, Ratajczak J: Horizontal transfer of RNA and proteins between cells by extracellular microvesicles: 14 years later. Clin Transl Med 2016;5:7–0087. 10.1186/s40169-016-0087-4 26943717 PMC4779088

[40] Felicetti F, De FA, Coscia C, Puglisi R, Pedini F, Pasquini L, et al: Exosome-mediated transfer of miR-222 is sufficient to increase tumor malignancy in melanoma. J Transl Med 2016;14:56–0811. 10.1186/s12967-016-0811-2 26912358 PMC4765208

[41] Yan J, Wang H, Liu Y, Shao C: Analysis of gene regulatory networks in the mammalian circadian rhythm. PLoS Comput Biol 2008;4:e1000193 10.1371/journal.pcbi.1000193 18846204 PMC2543109

[42] Martinez de PA, Sanchez-Mut JV, Samitier-Marti M, Petazzi P, Saez M, Szczesna K, et al: Circadian cycle-dependent MeCP2 and brain chromatin changes. PLoS ONE 2015;10:e0123693 10.1371/journal.pone.0123693 25875630 PMC4395115

[43] Takahashi H, Umeda N, Tsutsumi Y, Fukumura R, Ohkaze H, Sujino M, et al: Mouse dexamethasone-induced RAS protein 1 gene is expressed in a circadian rhythmic manner in the suprachiasmatic nucleus. Brain Res Mol Brain Res 2003;110:1–6. 12573527 10.1016/s0169-328x(02)00543-0

[44] Altman BJ, Hsieh AL, Sengupta A, Krishnanaiah SY, Stine ZE, Walton ZE, et al: MYC Disrupts the Circadian Clock and Metabolism in Cancer Cells. Cell Metab 2015;10. 10.1016/j.cmet.2015.09.003 PMC4818967 26387865

[45] Curtis AM, Fagundes CT, Yang G, Palsson-McDermott EM, Wochal P, McGettrick AF, et al: Circadian control of innate immunity in macrophages by miR-155 targeting Bmal1. Proc Natl Acad Sci U S A 2015;112:7231–7236. 10.1073/pnas.1501327112 25995365 PMC4466714

[46] Born J, Lange T, Hansen K, Molle M, Fehm HL: Effects of sleep and circadian rhythm on human circulating immune cells. J Immunol 1997;158:4454–4464. 9127011

[47] Scheiermann C, Kunisaki Y, Frenette PS: Circadian control of the immune system. Nat Rev Immunol 2013;13:190–198. 10.1038/nri3386 23391992 PMC4090048

[48] Weigl Y, Ashkenazi IE, Peleg L: Rhythmic profiles of cell cycle and circadian clock gene transcripts in mice: a possible association between two periodic systems. J Exp Biol 2013;216:2276–2282. 10.1242/jeb.081729 23531816

[49] Jakobsen JS, Waage J, Rapin N, Bisgaard HC, Larsen FS, Porse BT: Temporal mapping of CEBPA and CEBPB binding during liver regeneration reveals dynamic occupancy and specific regulatory codes for homeostatic and cell cycle gene batteries. Genome Res 2013;23:592–603. 10.1101/gr.146399.112 23403033 PMC3613577

[50] Zak DE, Hao H, Vadigepalli R, Miller GM, Ogunnaike BA, Schwaber JS: Systems analysis of circadian time-dependent neuronal epidermal growth factor receptor signaling. Genome Biol 2006;7:R48 16784547 10.1186/gb-2006-7-6-r48 PMC1779538

[51] Wu J, Dauchy RT, Tirrell PC, Wu SS, Lynch DT, Jitawatanarat P, et al: Light at night activates IGF-1R/PDK1 signaling and accelerates tumor growth in human breast cancer xenografts. Cancer Res 2011;71:2622–2631. 10.1158/0008-5472.CAN-10-3837 21310824

[52] Saito T, Hirano M, Ide T, Ichiki T, Koibuchi N, Sunagawa K, et al: Pivotal role of Rho-associated kinase 2 in generating the intrinsic circadian rhythm of vascular contractility. Circulation 2013;127:104–114. 10.1161/CIRCULATIONAHA.112.135608 23172836

[53] Ogawa S, Kwon CH, Zhou J, Koovakkattu D, Parada LF, Sinton CM: A seizure-prone phenotype is associated with altered free-running rhythm in Pten mutant mice. Brain Res 2007;1168:112–23. Epub;%2007 Jul;%20.:112–123. 17706614 10.1016/j.brainres.2007.06.074

[54] Fogg PC, O'Neill JS, Dobrzycki T, Calvert S, Lord EC, McIntosh RL, et al: Class IIa histone deacetylases are conserved regulators of circadian function. J Biol Chem 2014;289:34341–34348. 10.1074/jbc.M114.606392 25271152 PMC4256363

[55] Curtis AM, Seo SB, Westgate EJ, Rudic RD, Smyth EM, Chakravarti D, et al: Histone acetyltransferase-dependent chromatin remodeling and the vascular clock. J Biol Chem 2004;%20;279:7091–7097. 14645221 10.1074/jbc.M311973200

