# Supplementary material for: Diurnal Variations of Human Circulating Cell-Free Micro-RNA
Source: PLoS One. 2016 Aug 5;11(8):e0160577. doi: 10.1371/journal.pone.0160577 (PMC4975411; doi:10.1371/journal.pone.0160577)

## Slide 1
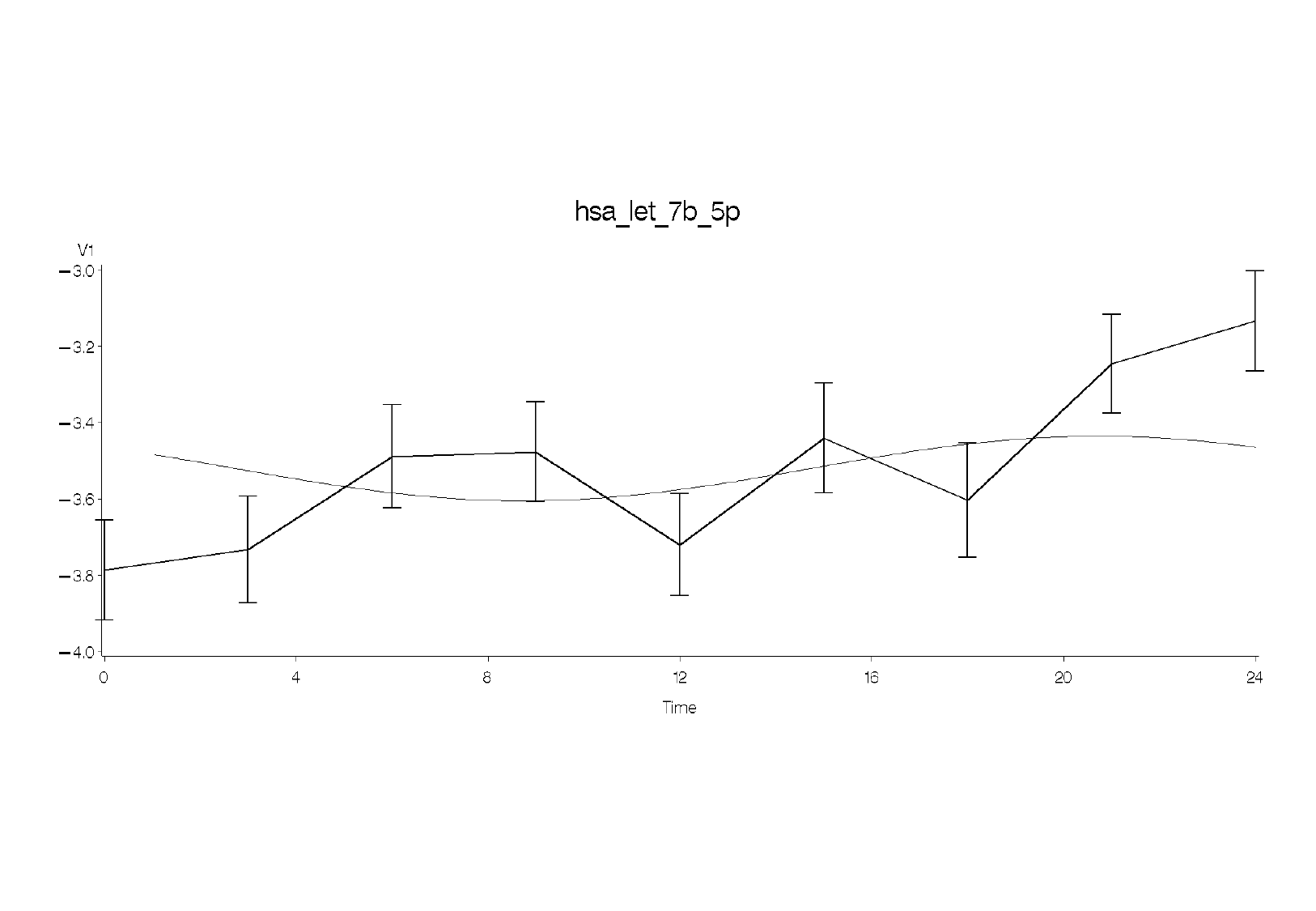

## Slide 2
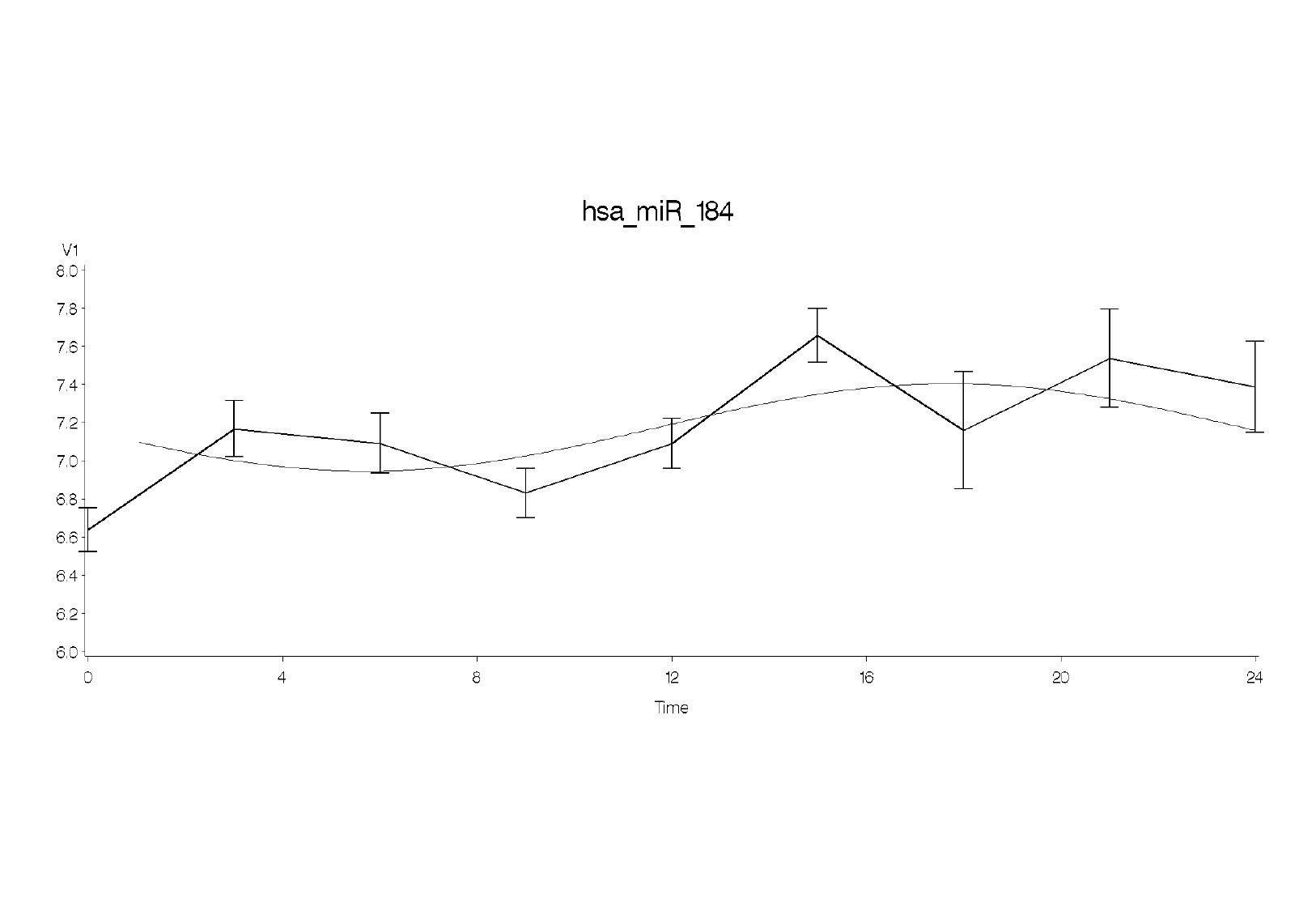

## Slide 3
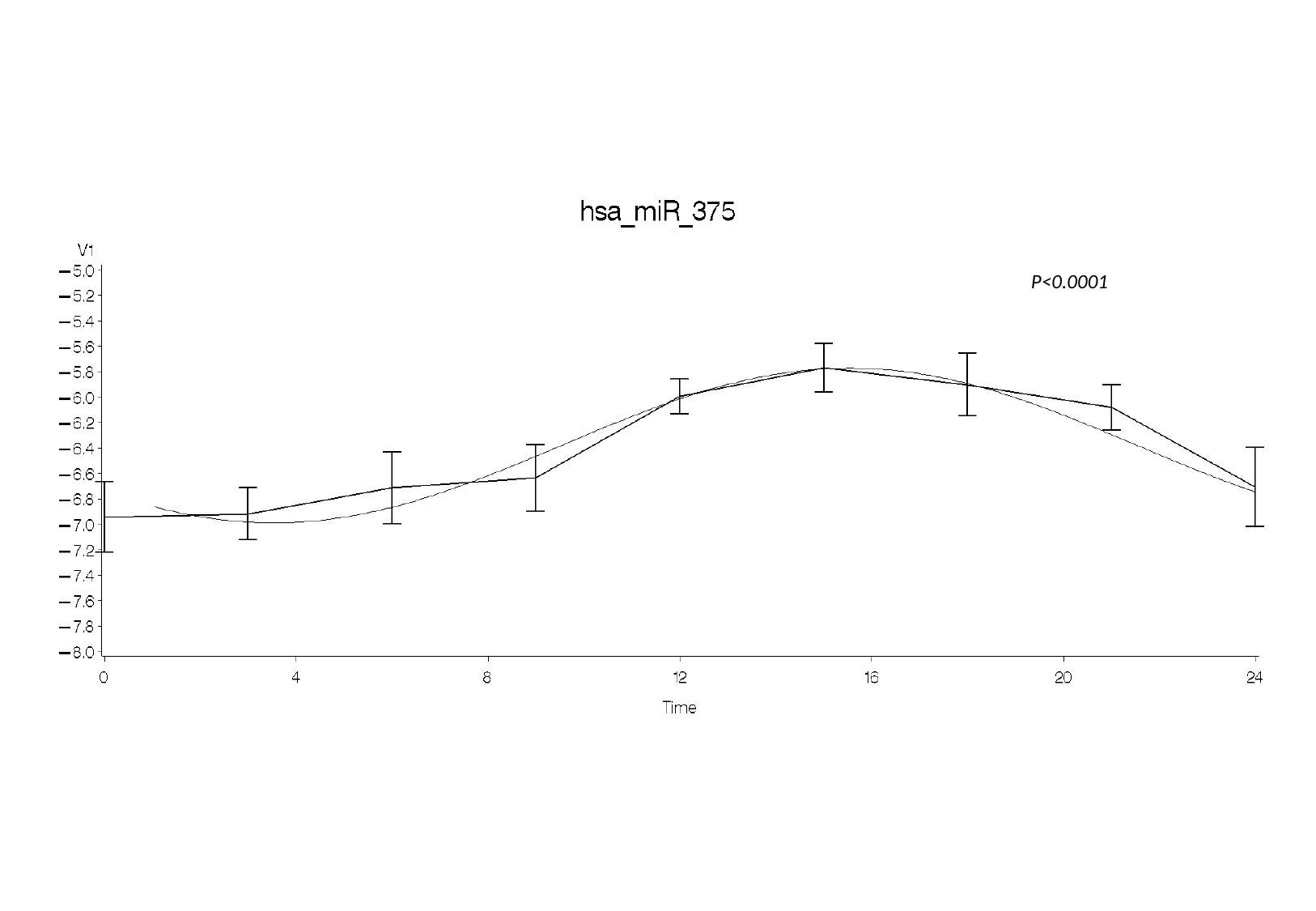

P<0.0001

## Slide 4
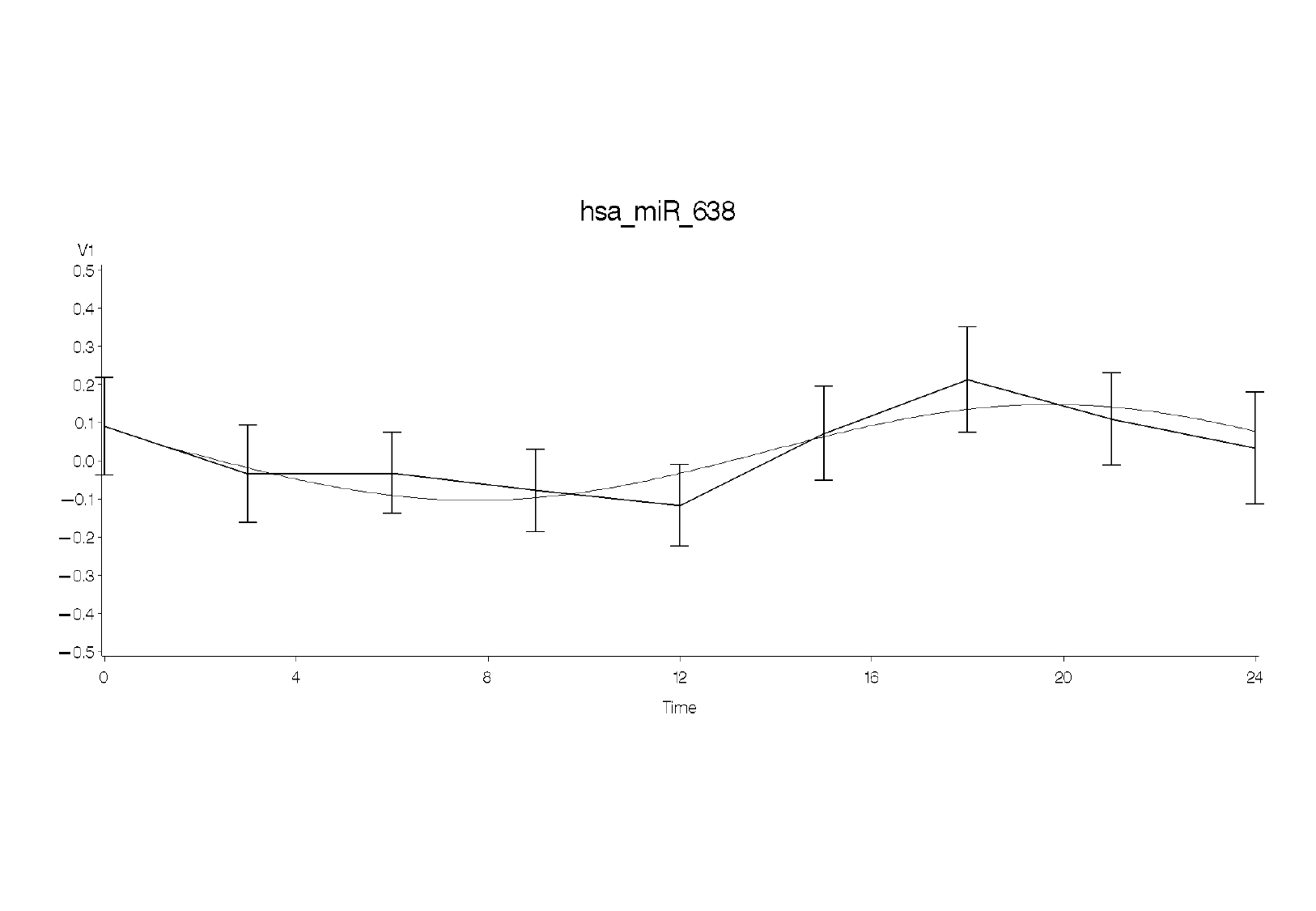

## Slide 5
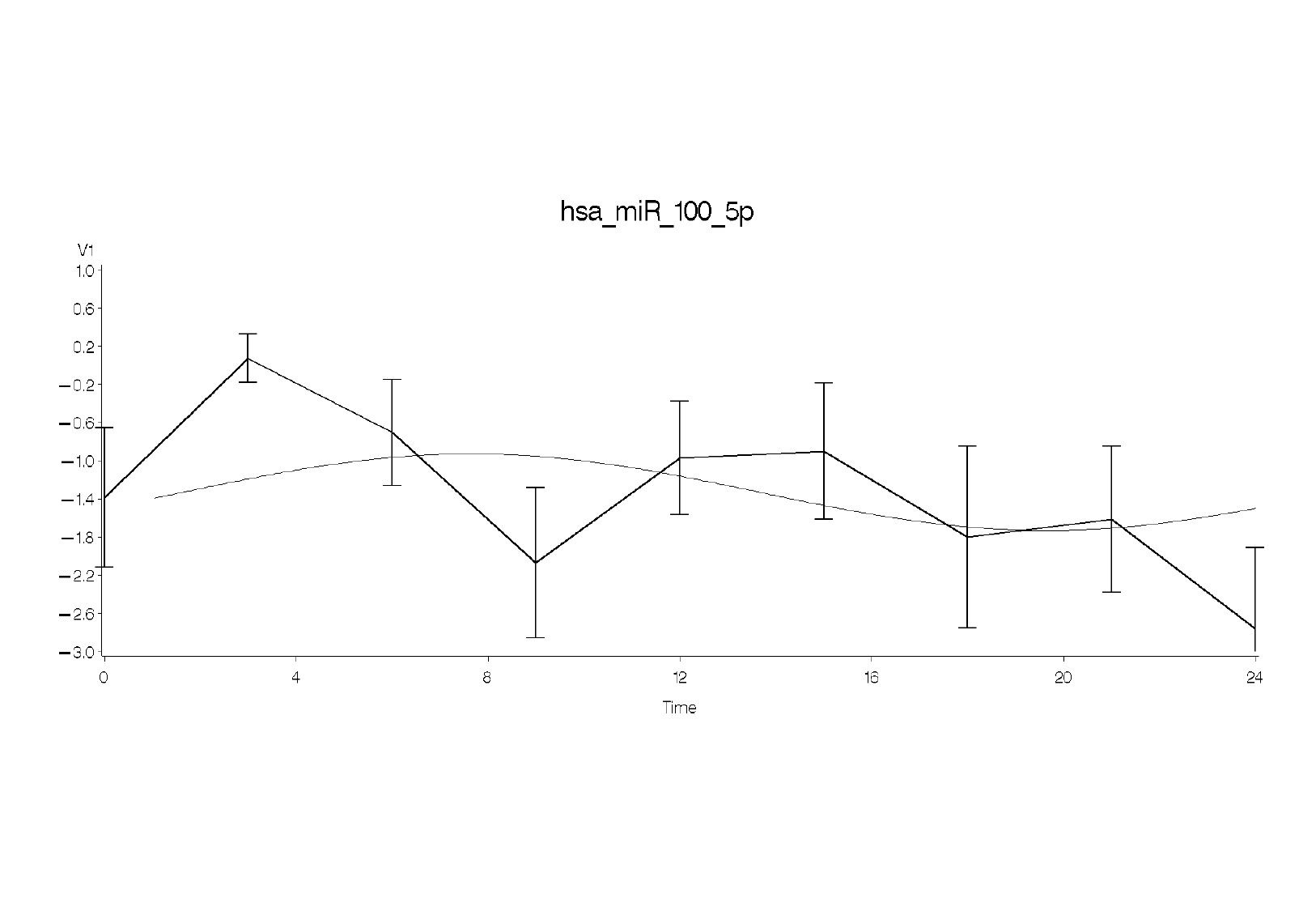

## Slide 6
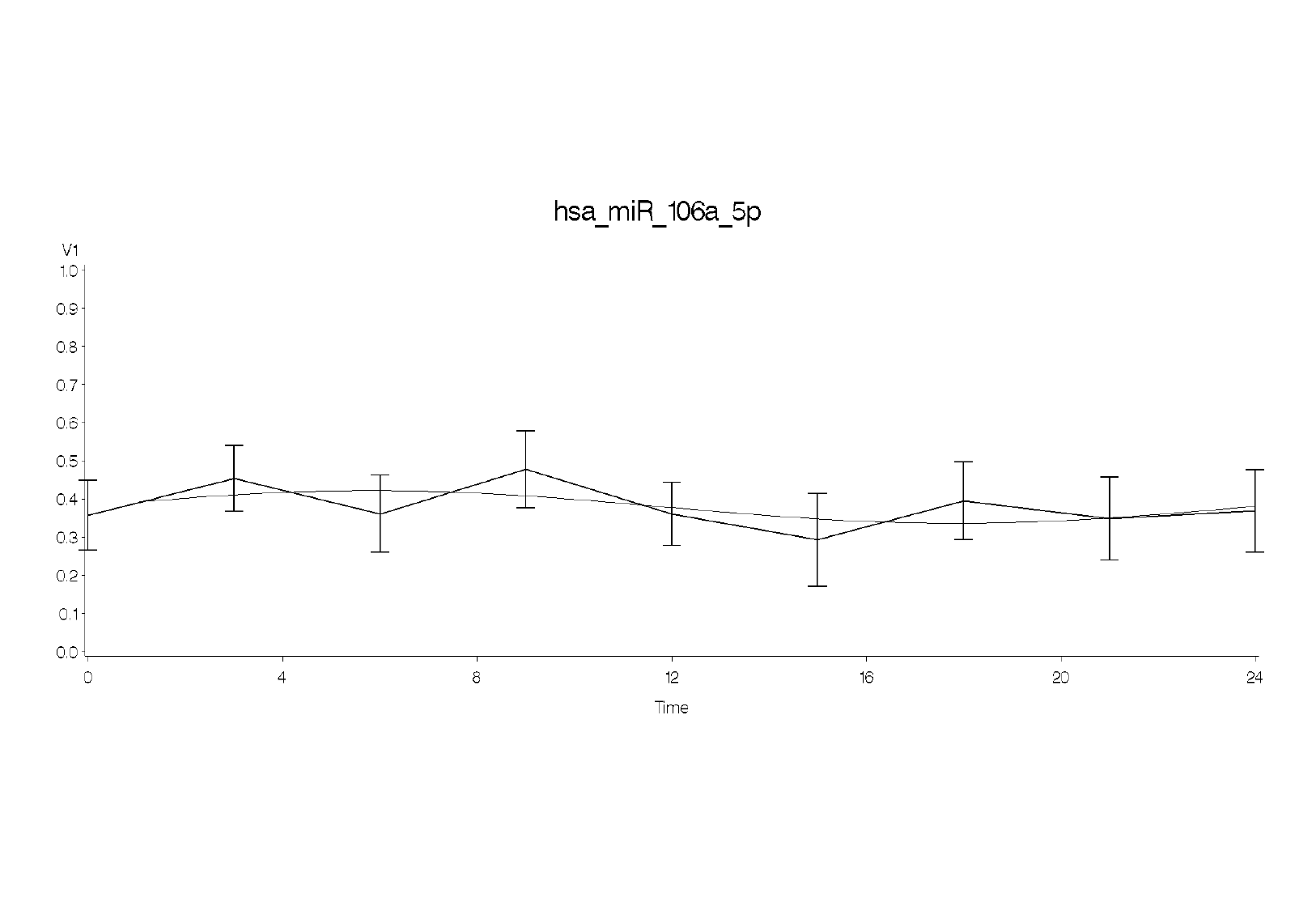

## Slide 7
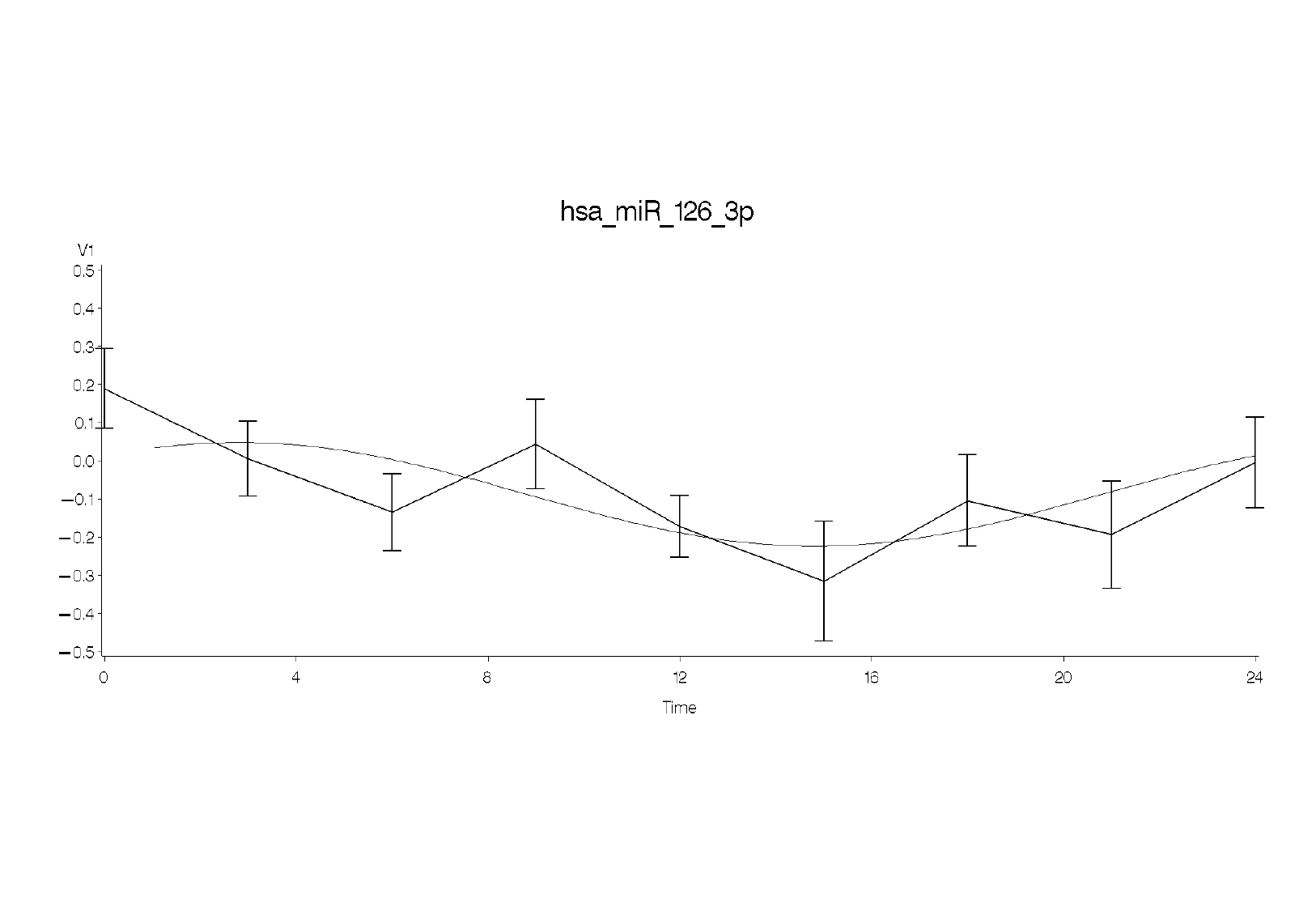

## Slide 8
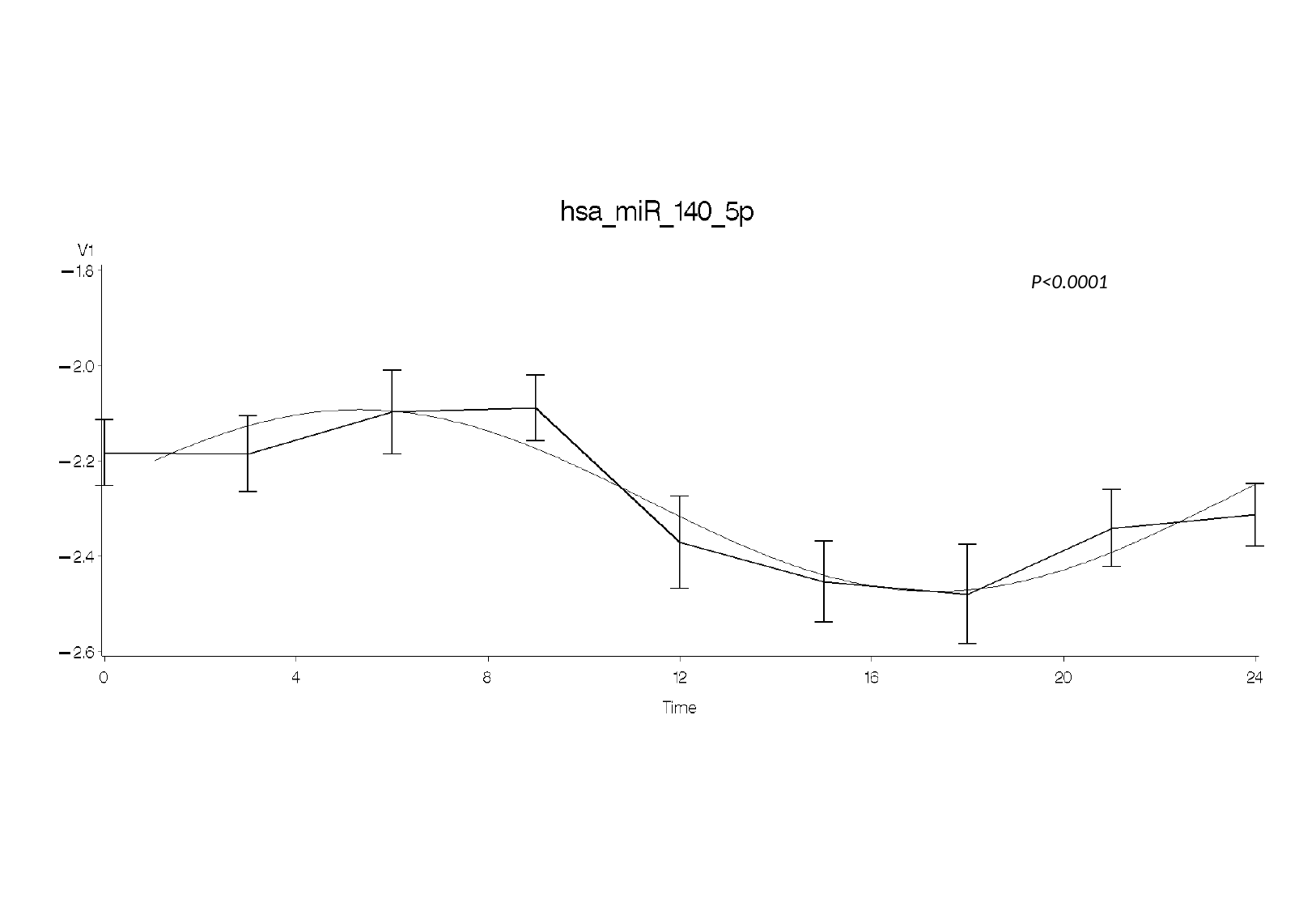

P<0.0001

## Slide 9
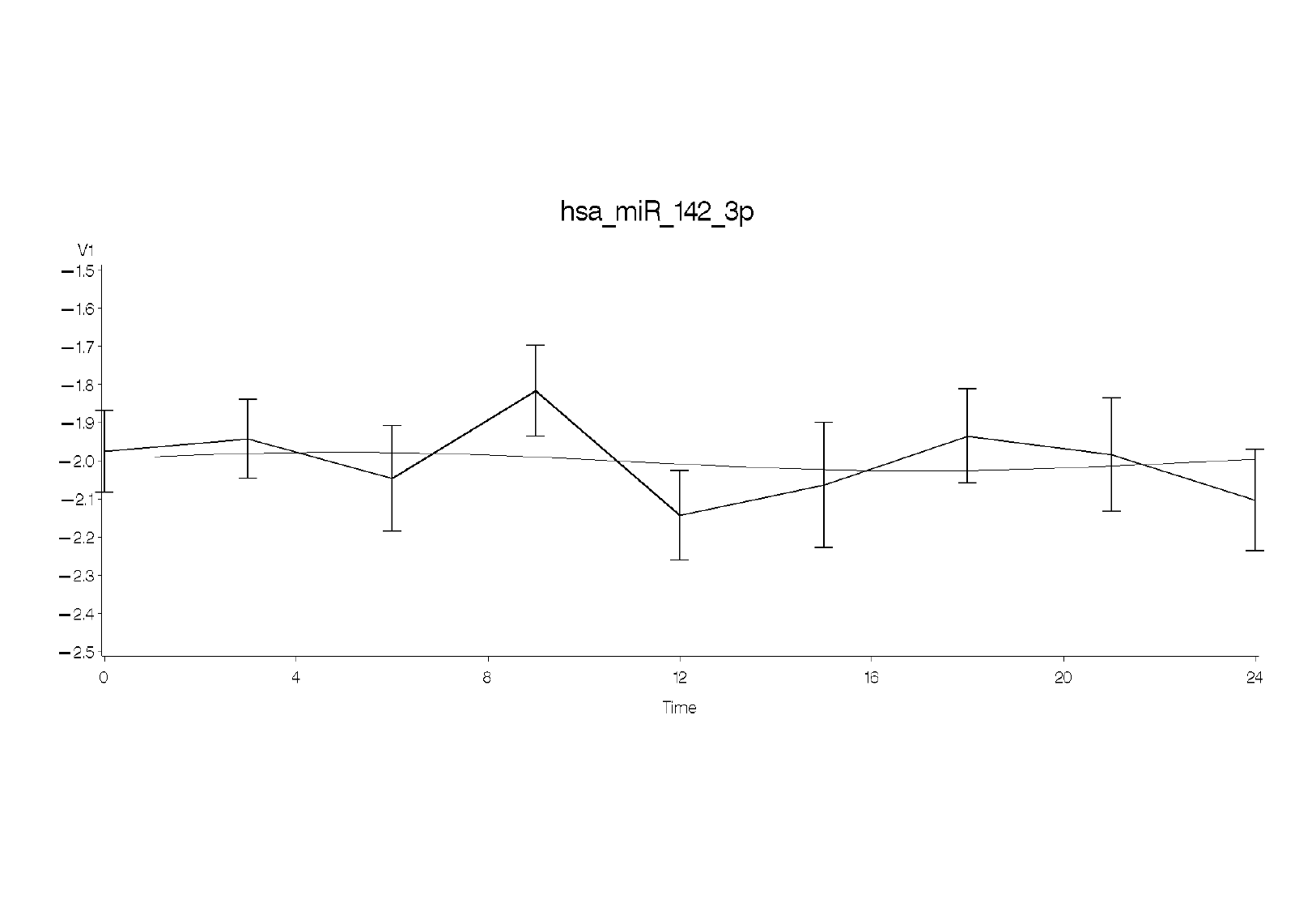

## Slide 10
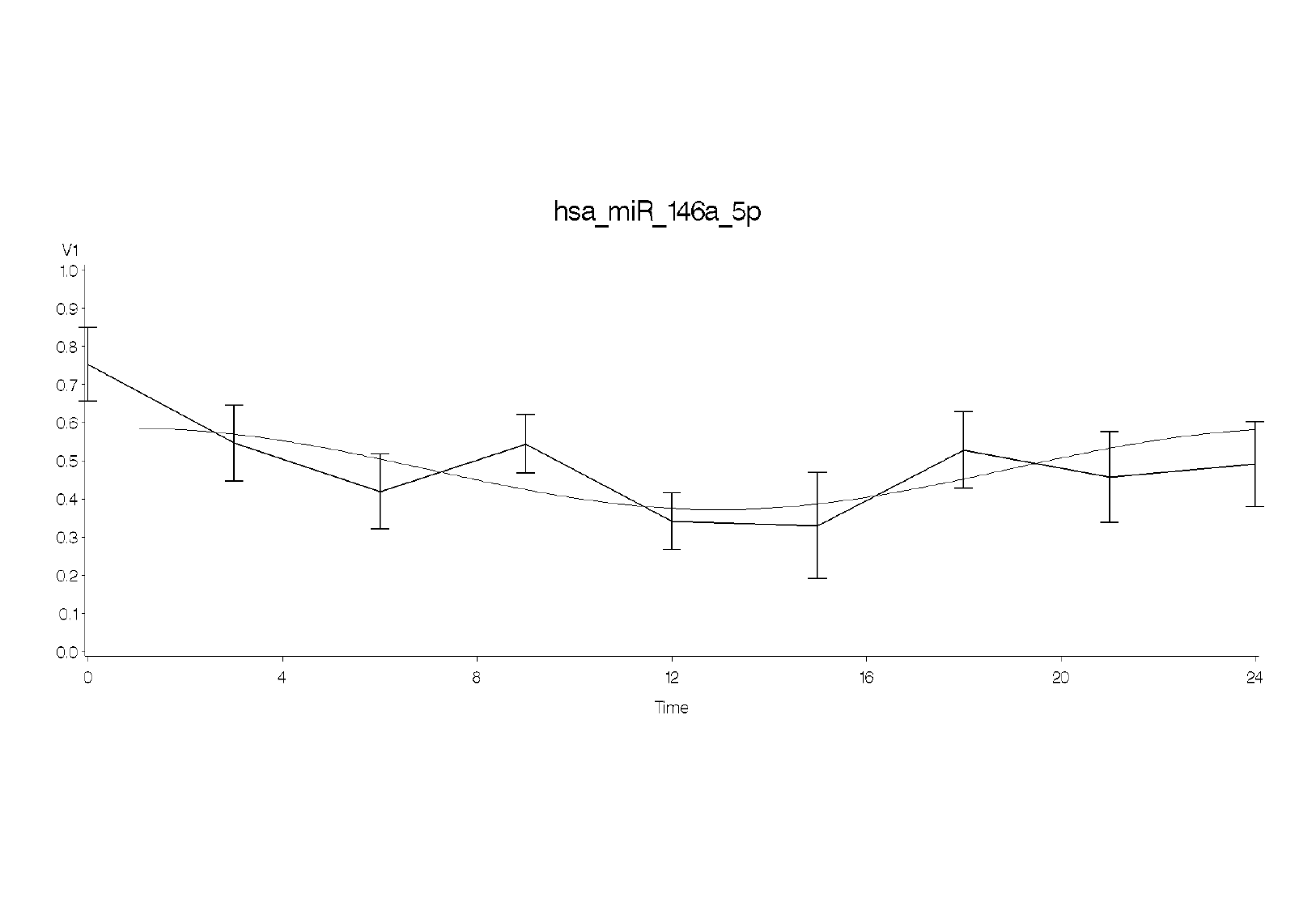

## Slide 11
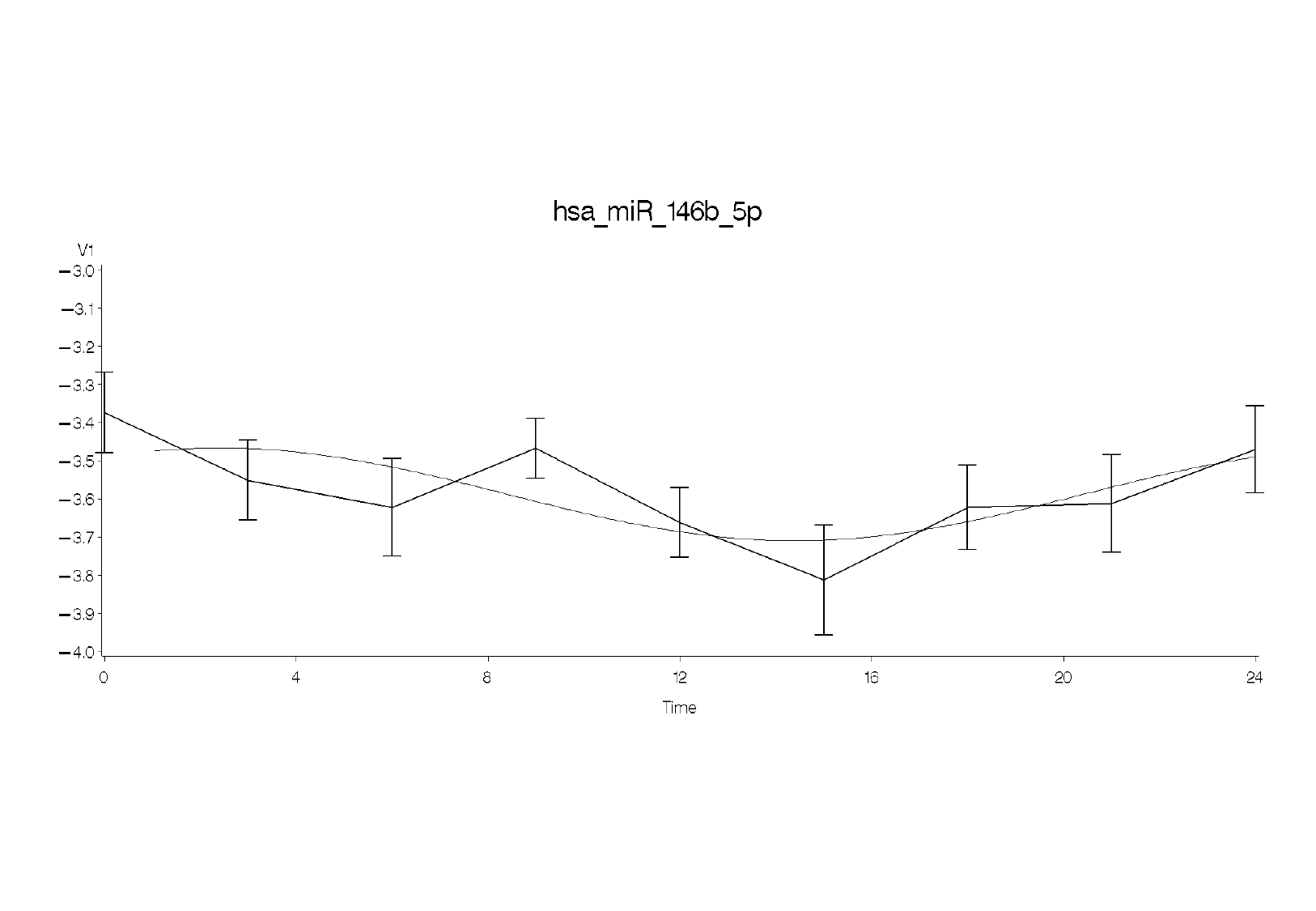

## Slide 12
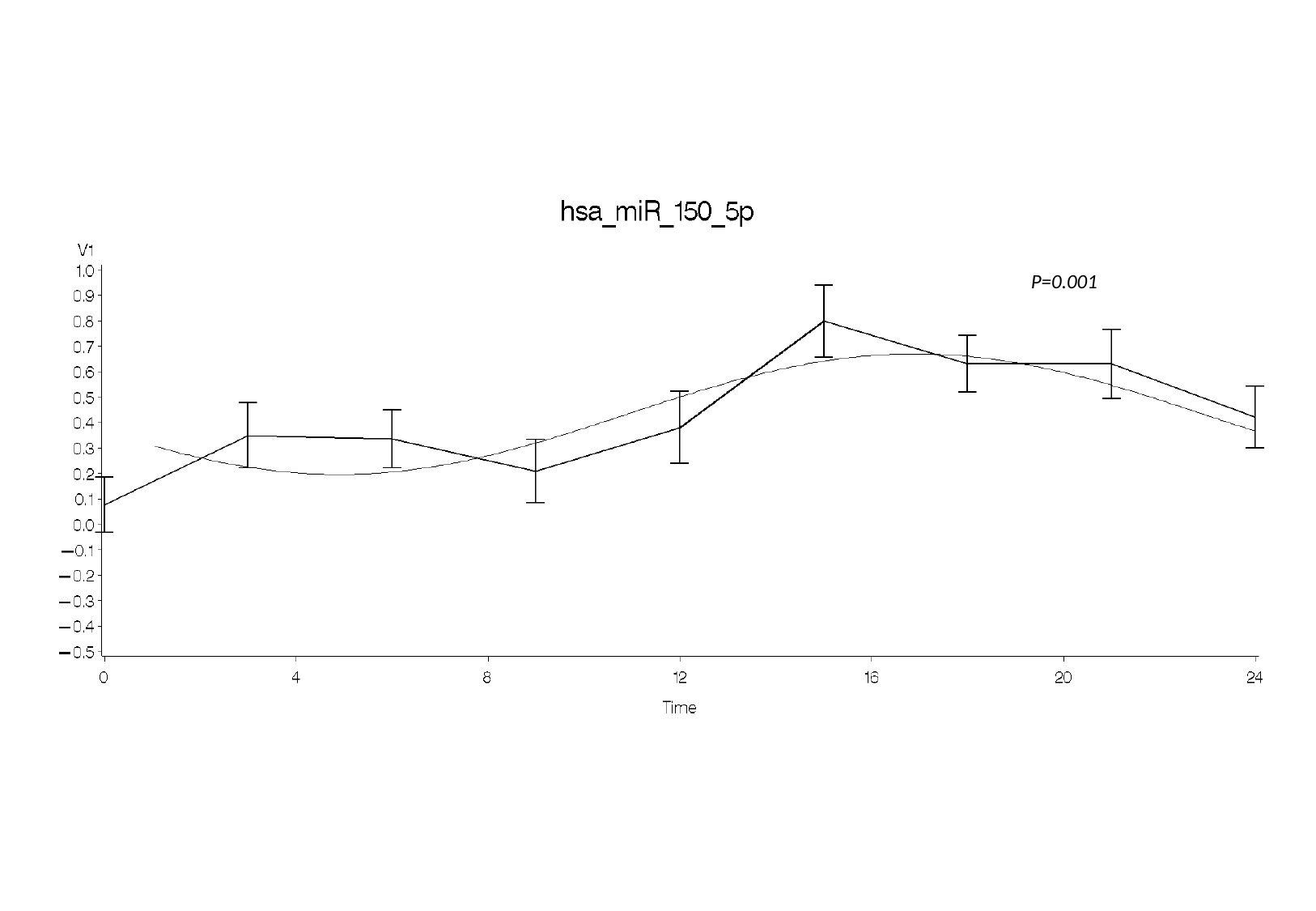

P=0.001

## Slide 13
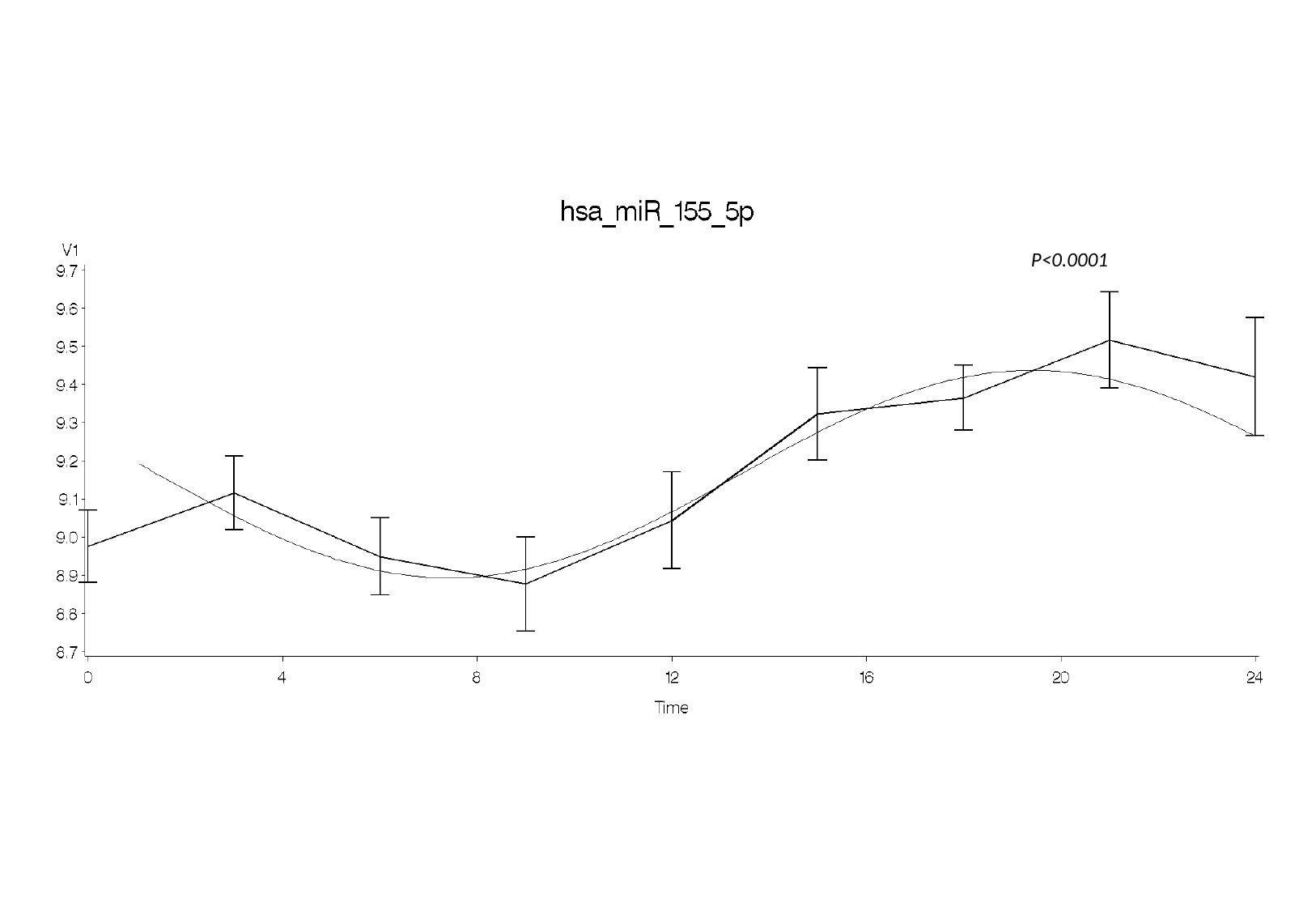

P<0.0001

## Slide 14
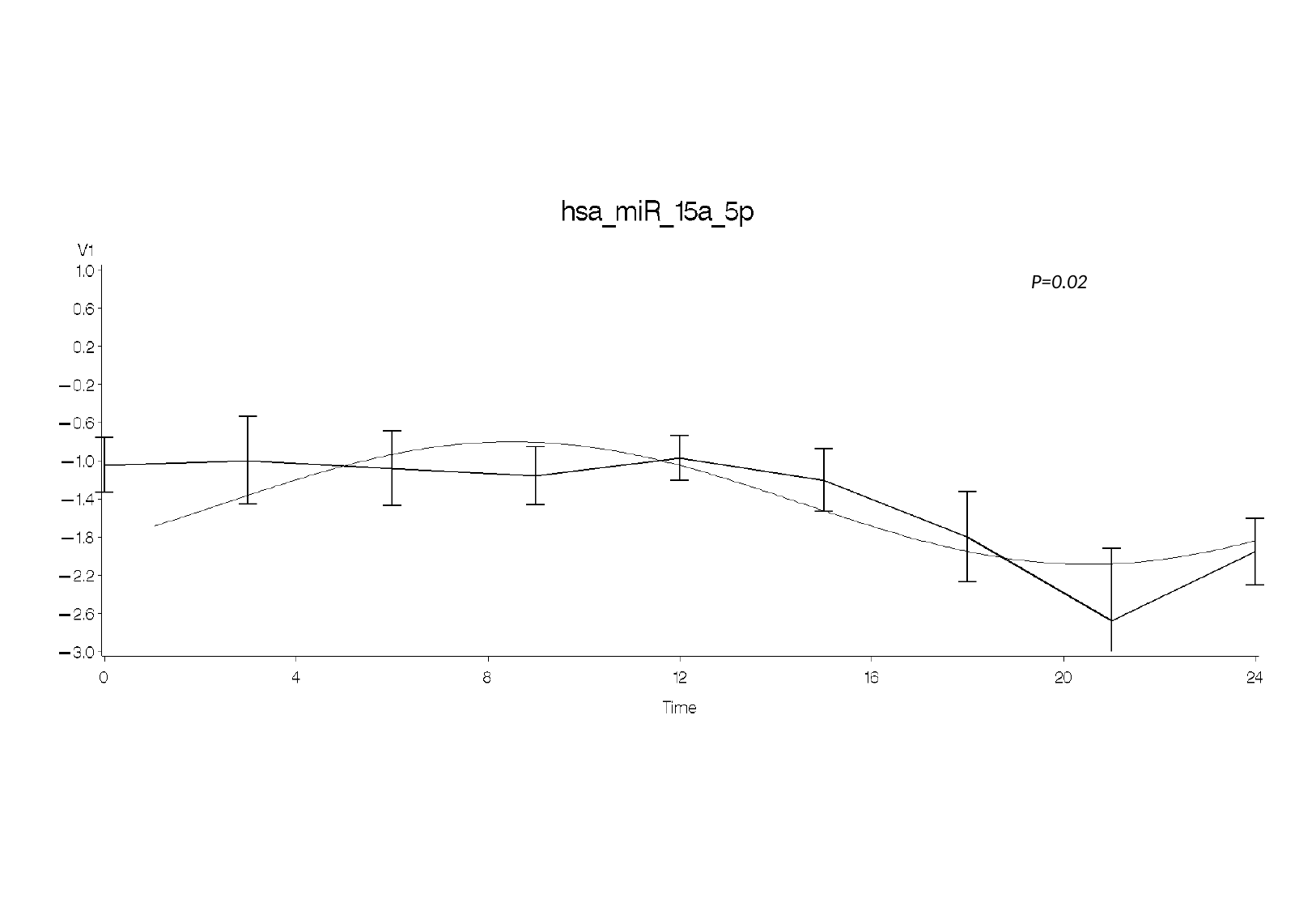

P=0.02

## Slide 15
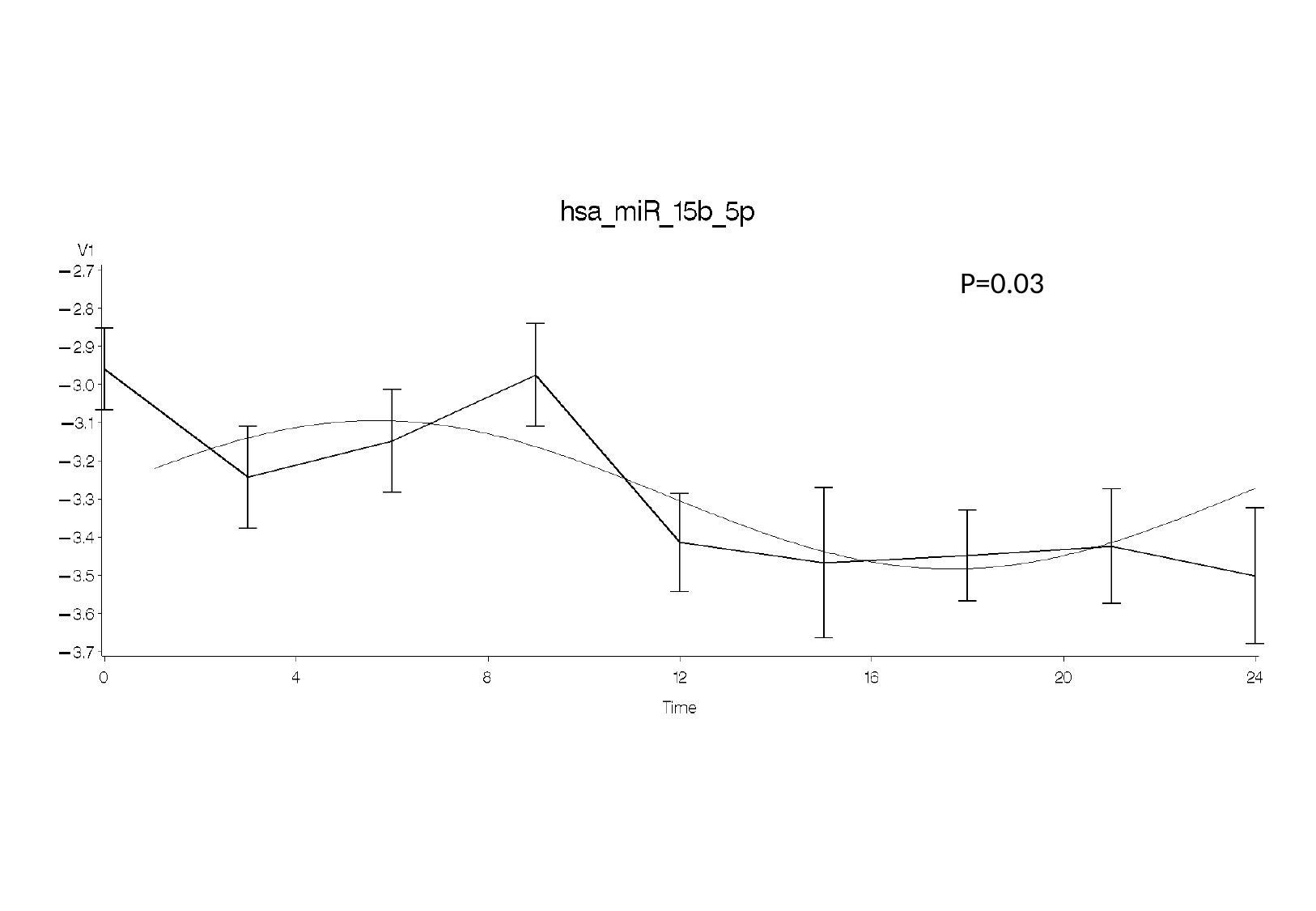

P=0.03

## Slide 16
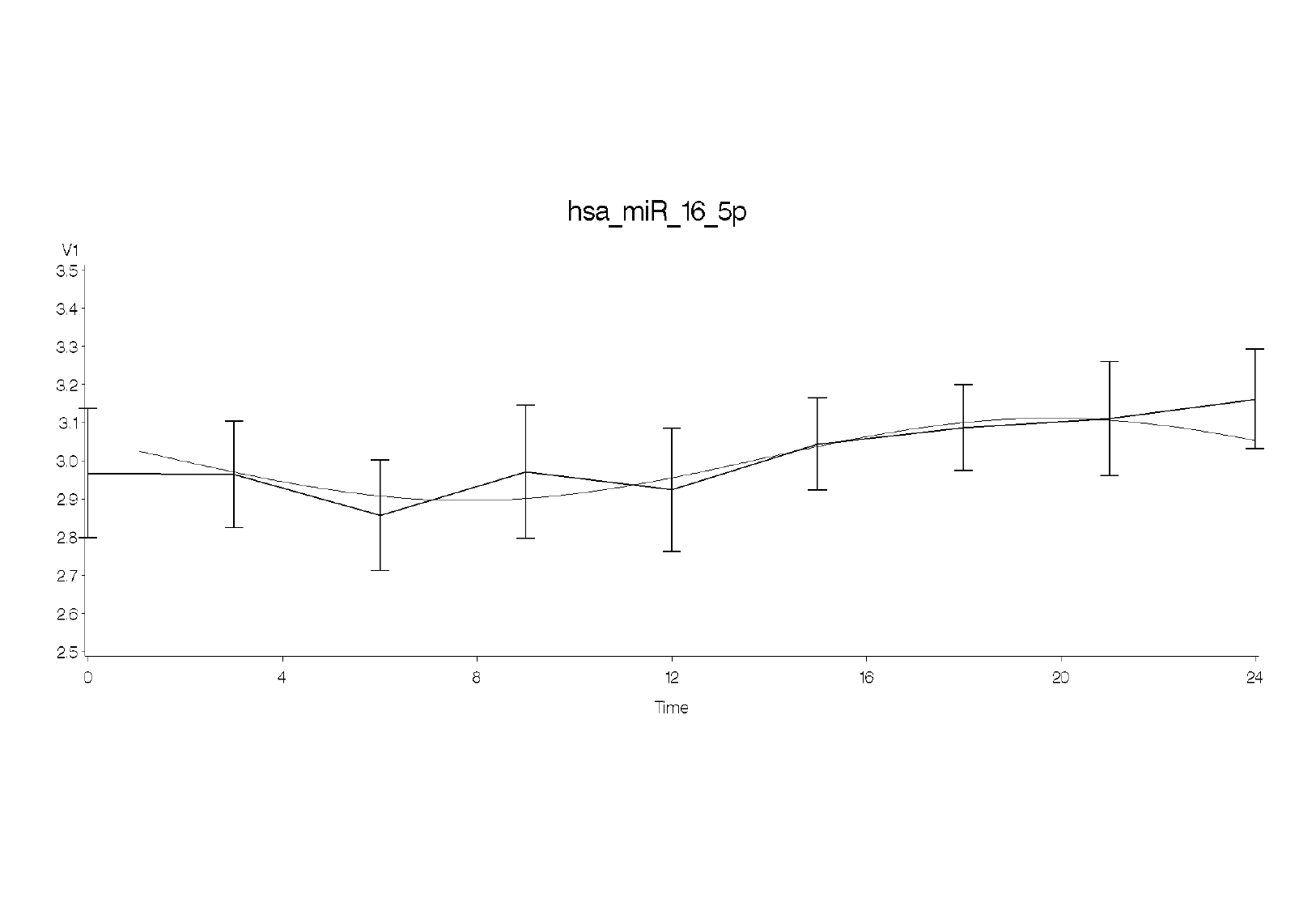

## Slide 17
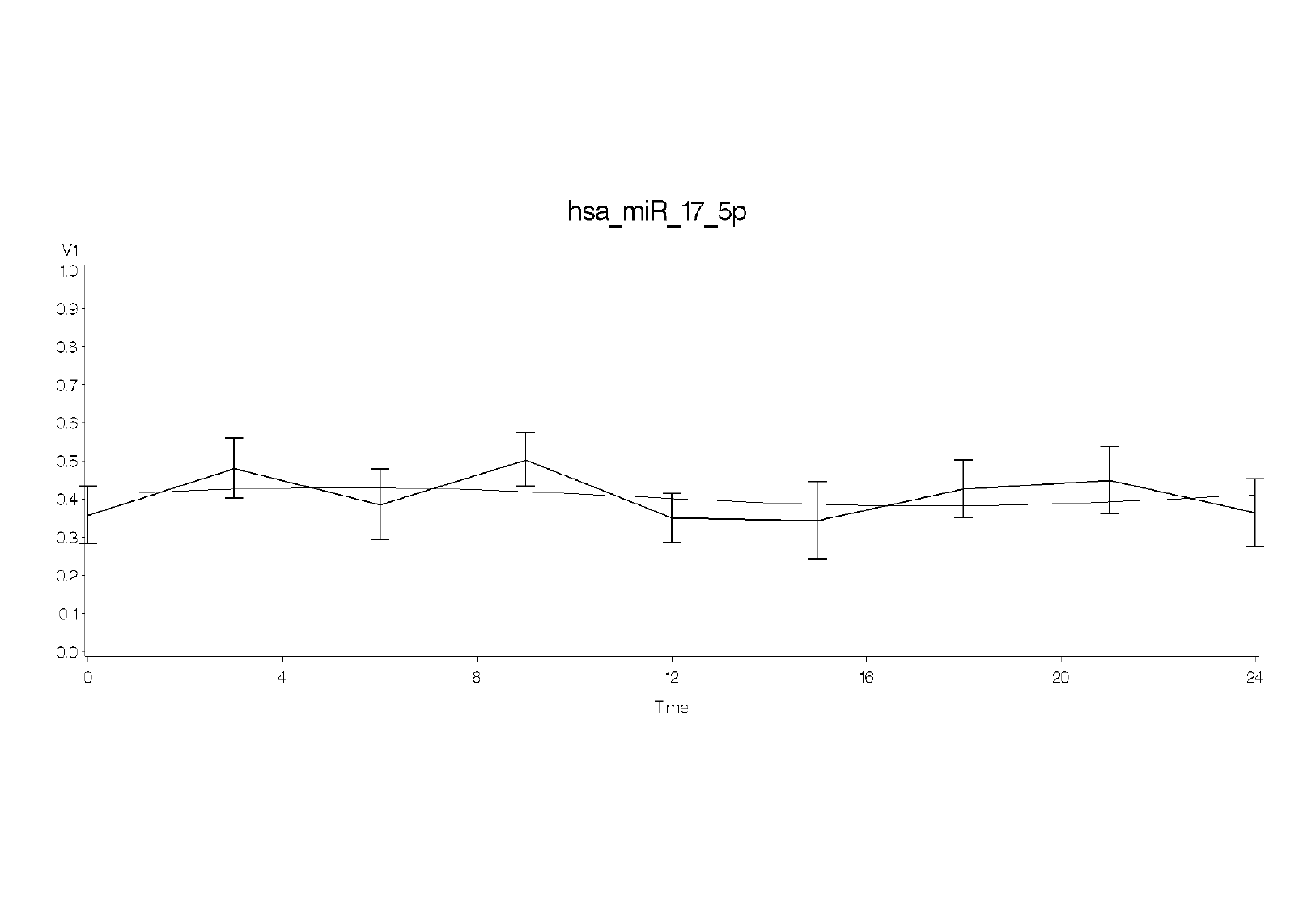

## Slide 18
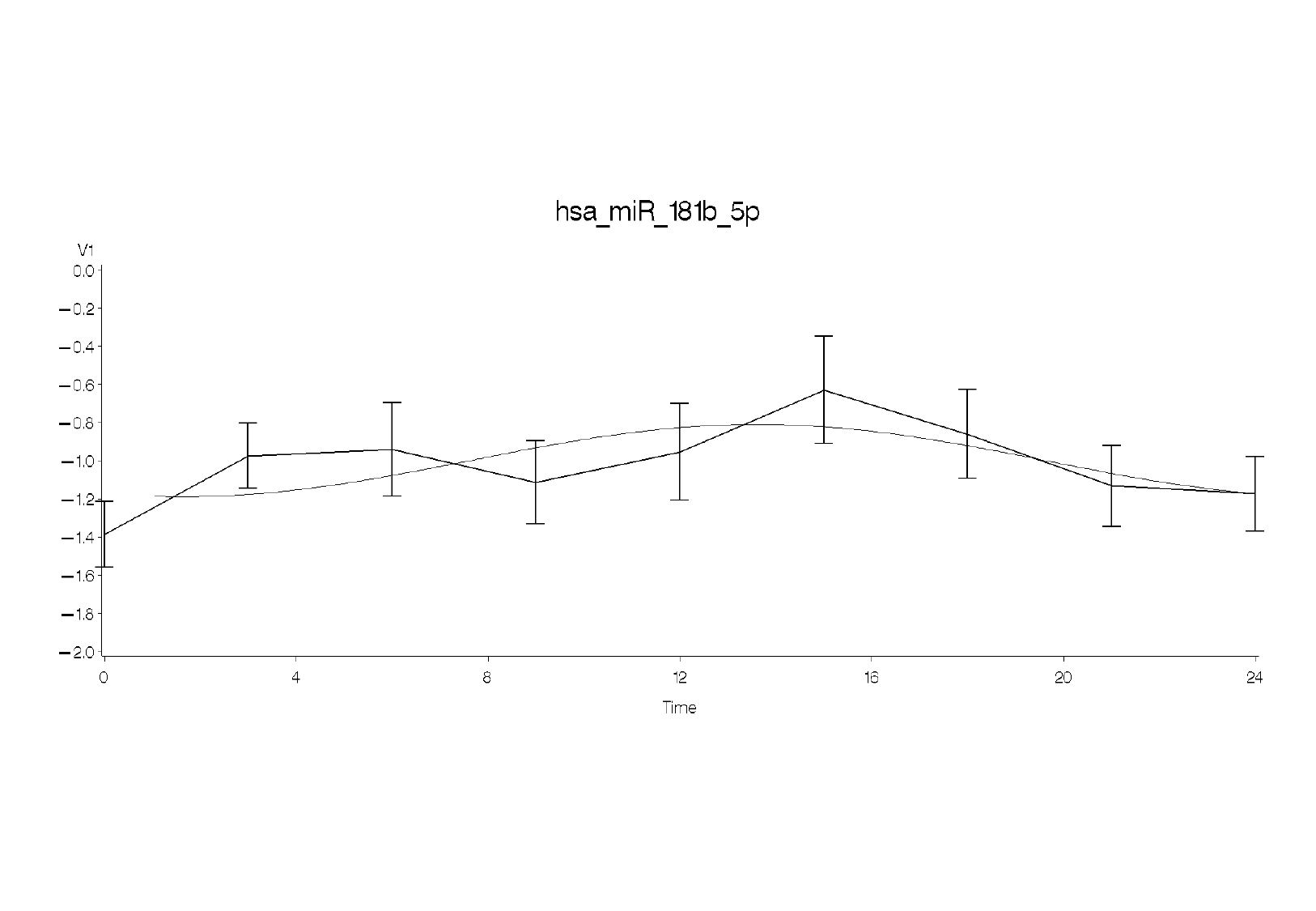

## Slide 19
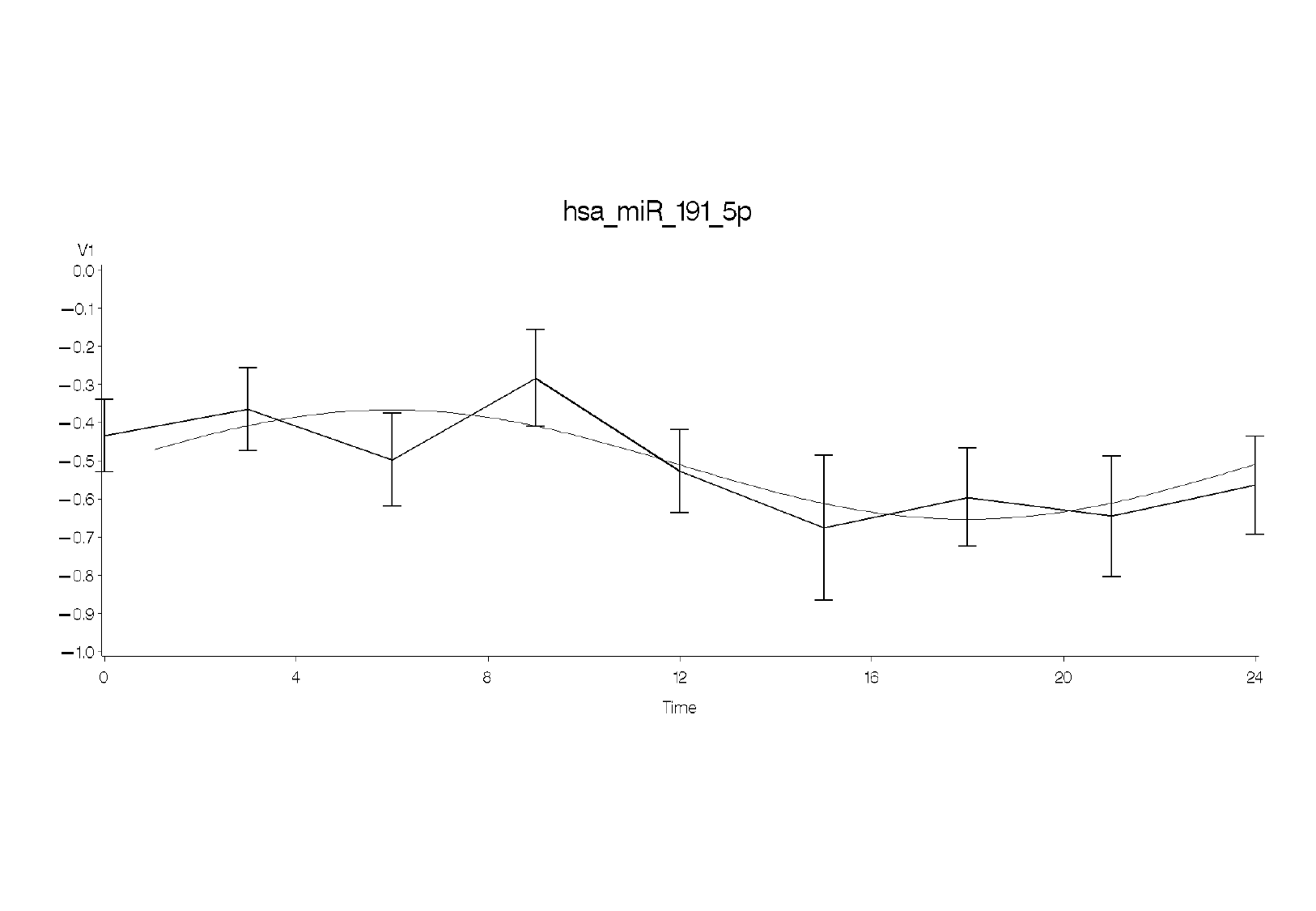

## Slide 20
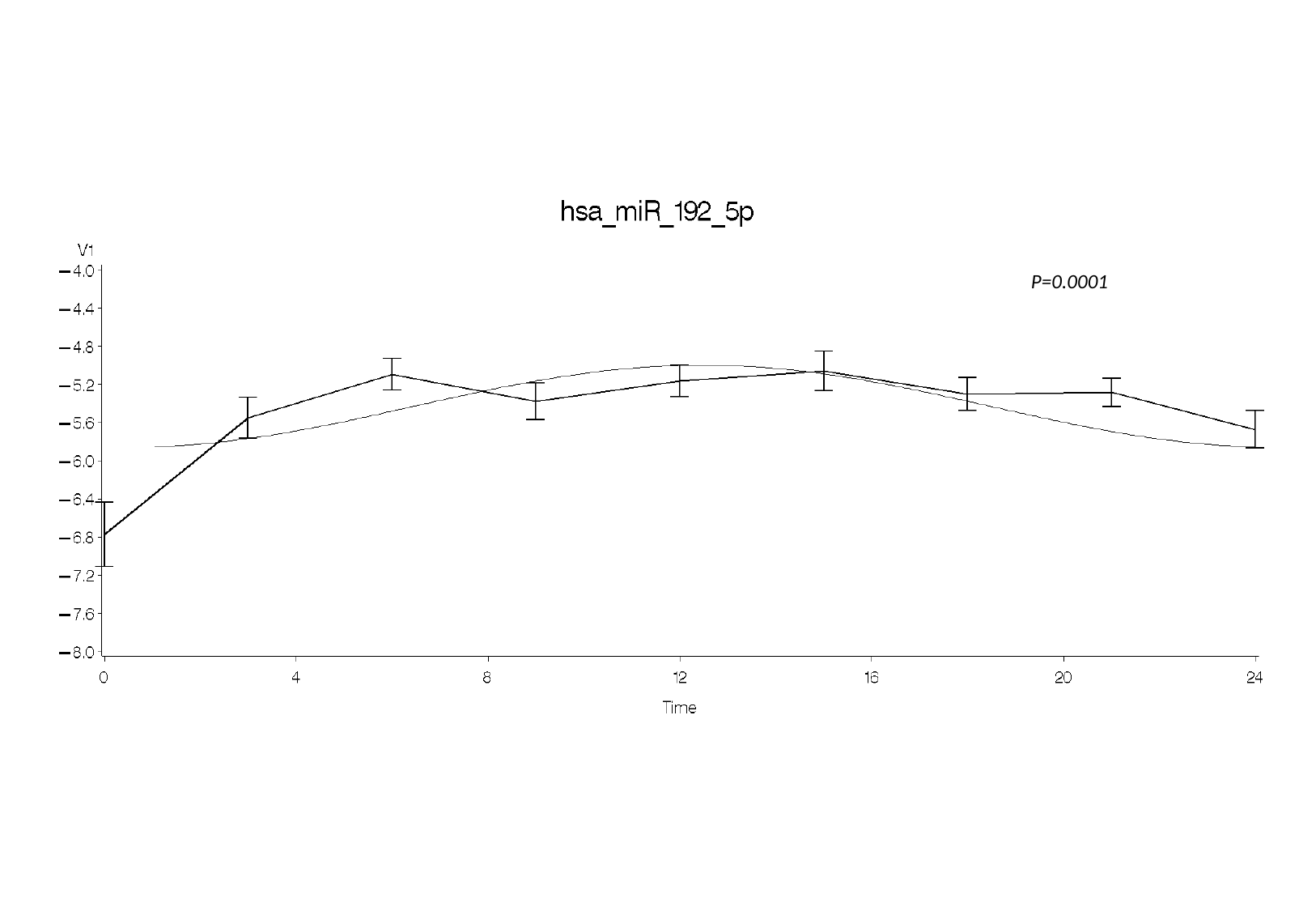

P=0.0001

## Slide 21
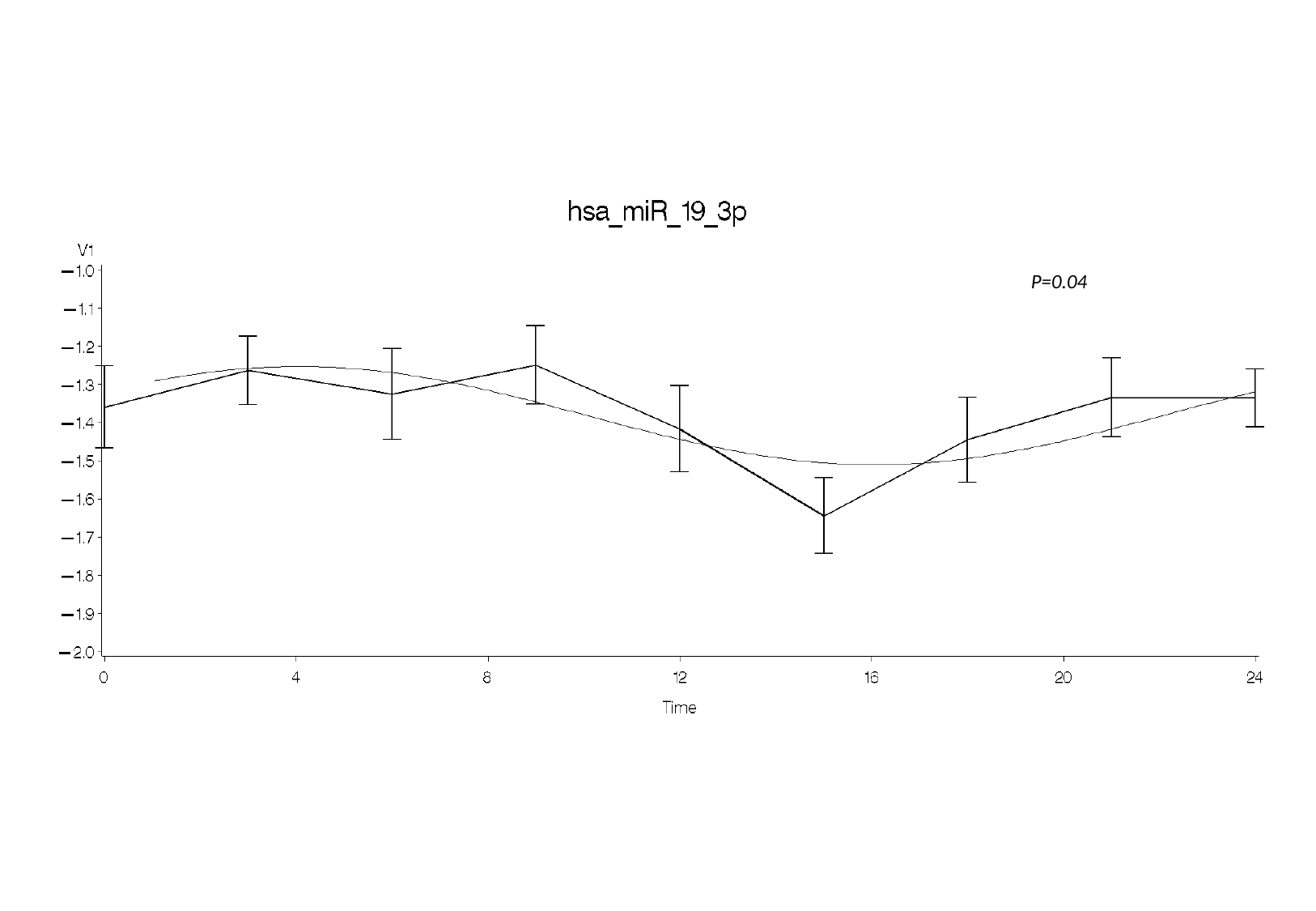

P=0.04

## Slide 22
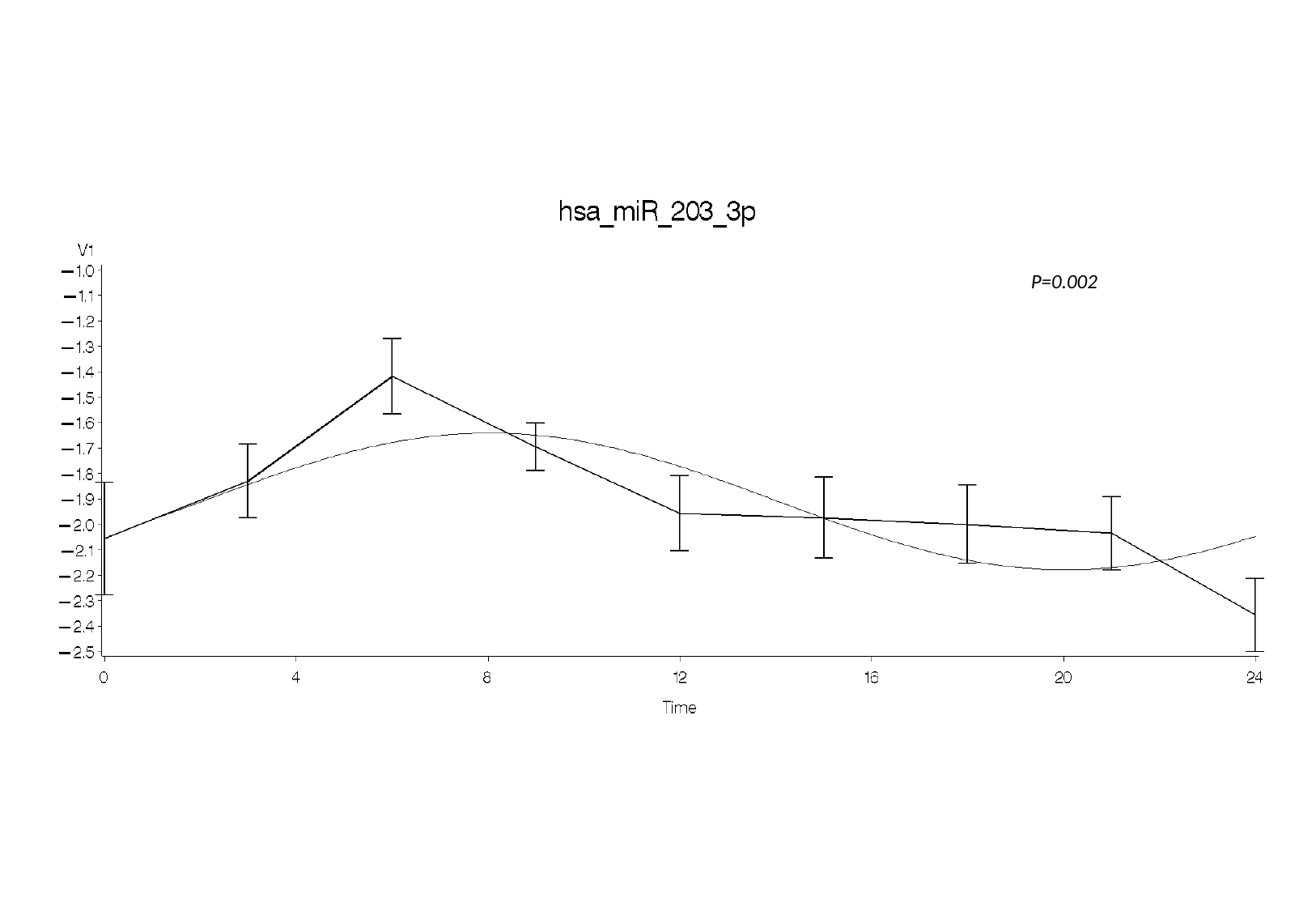

P=0.002

## Slide 23
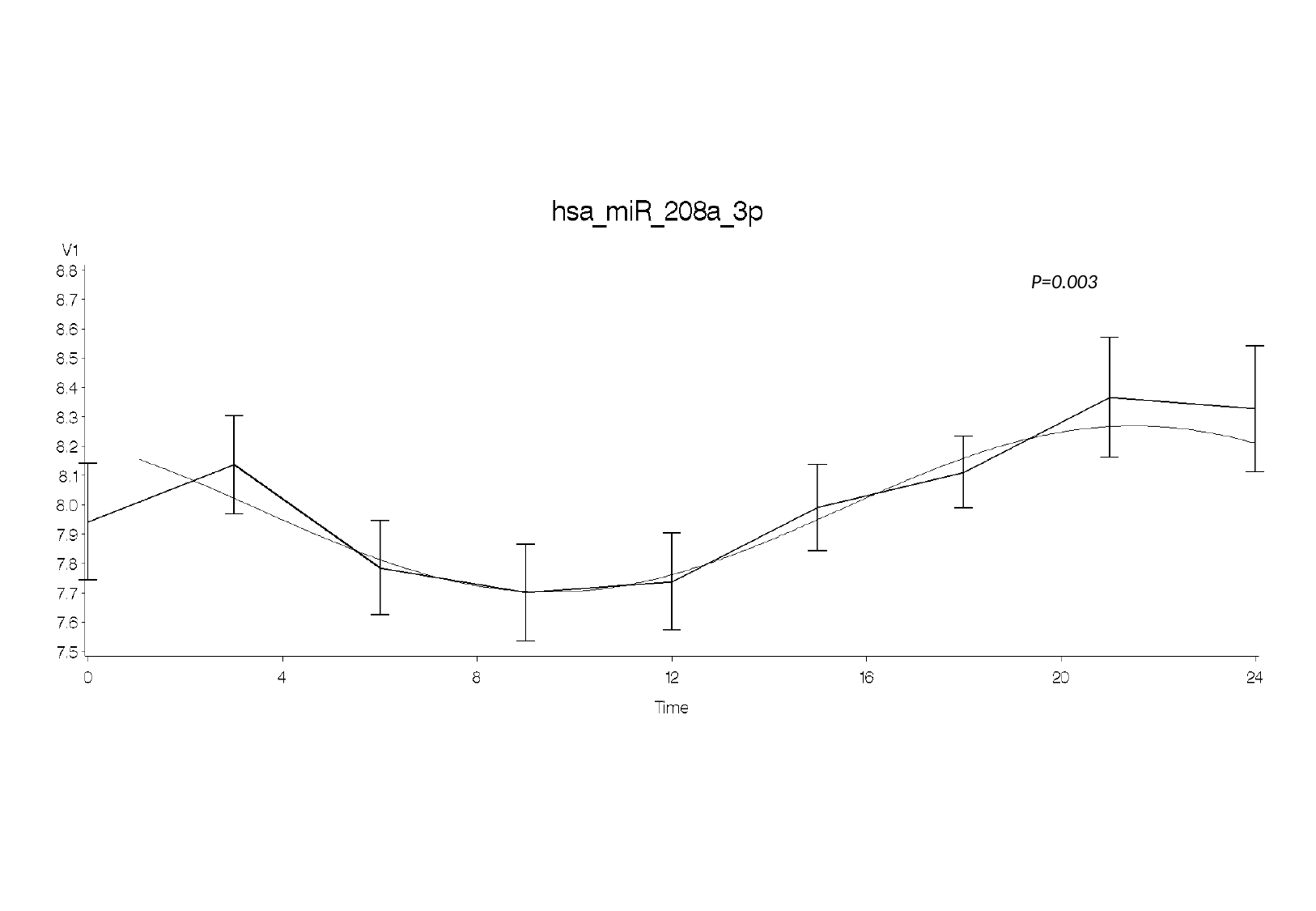

P=0.003

## Slide 24
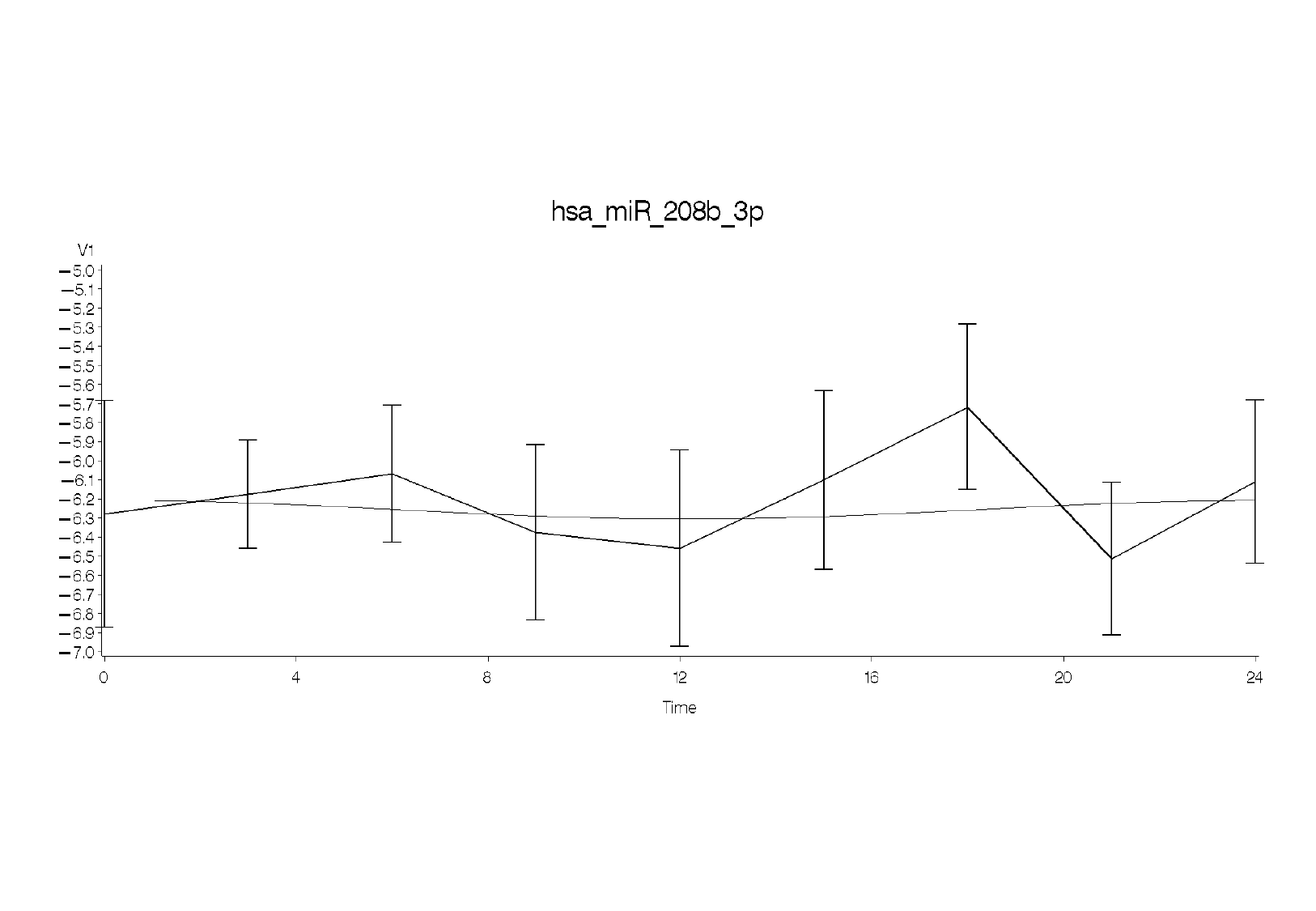

## Slide 25
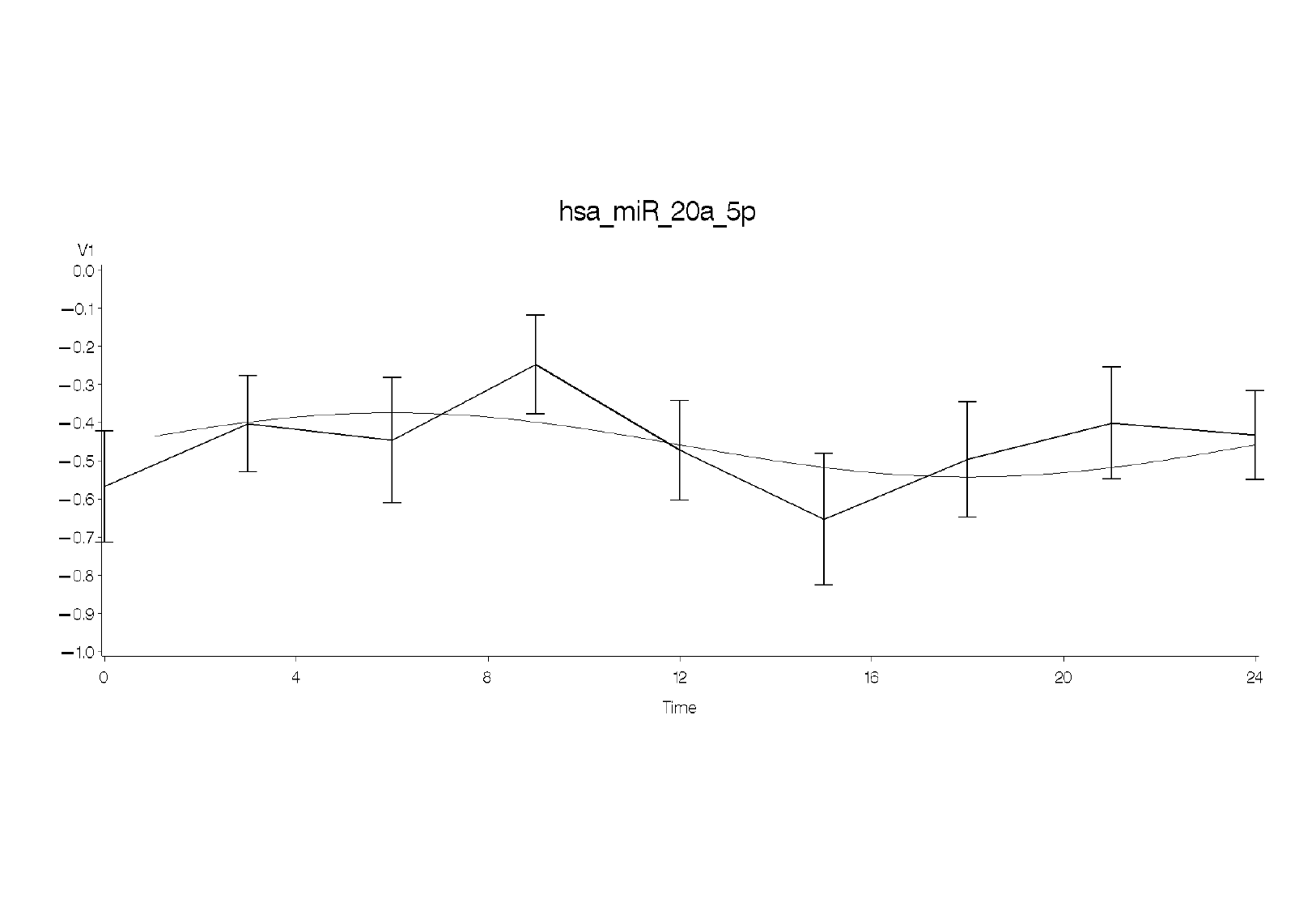

## Slide 26
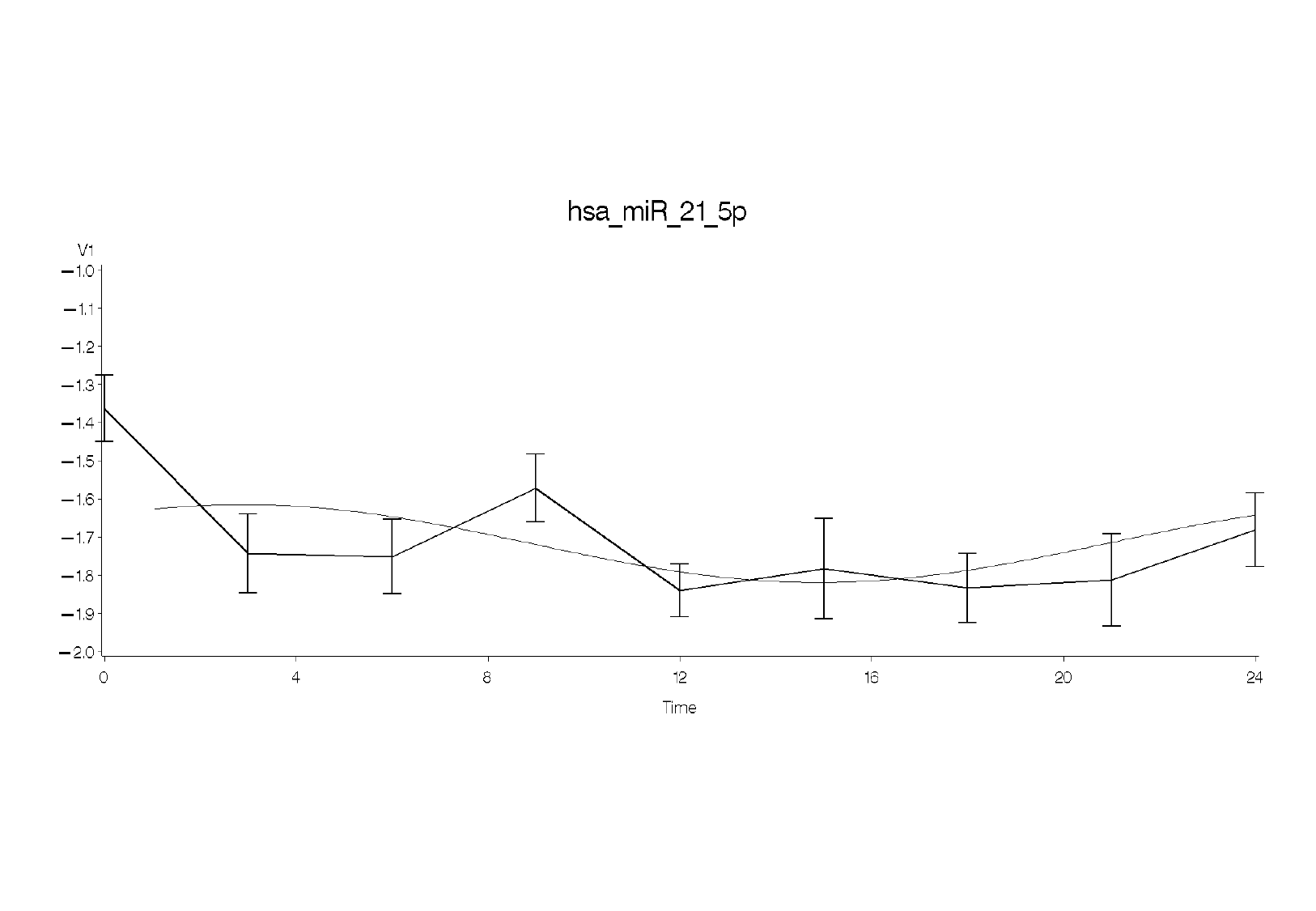

## Slide 27
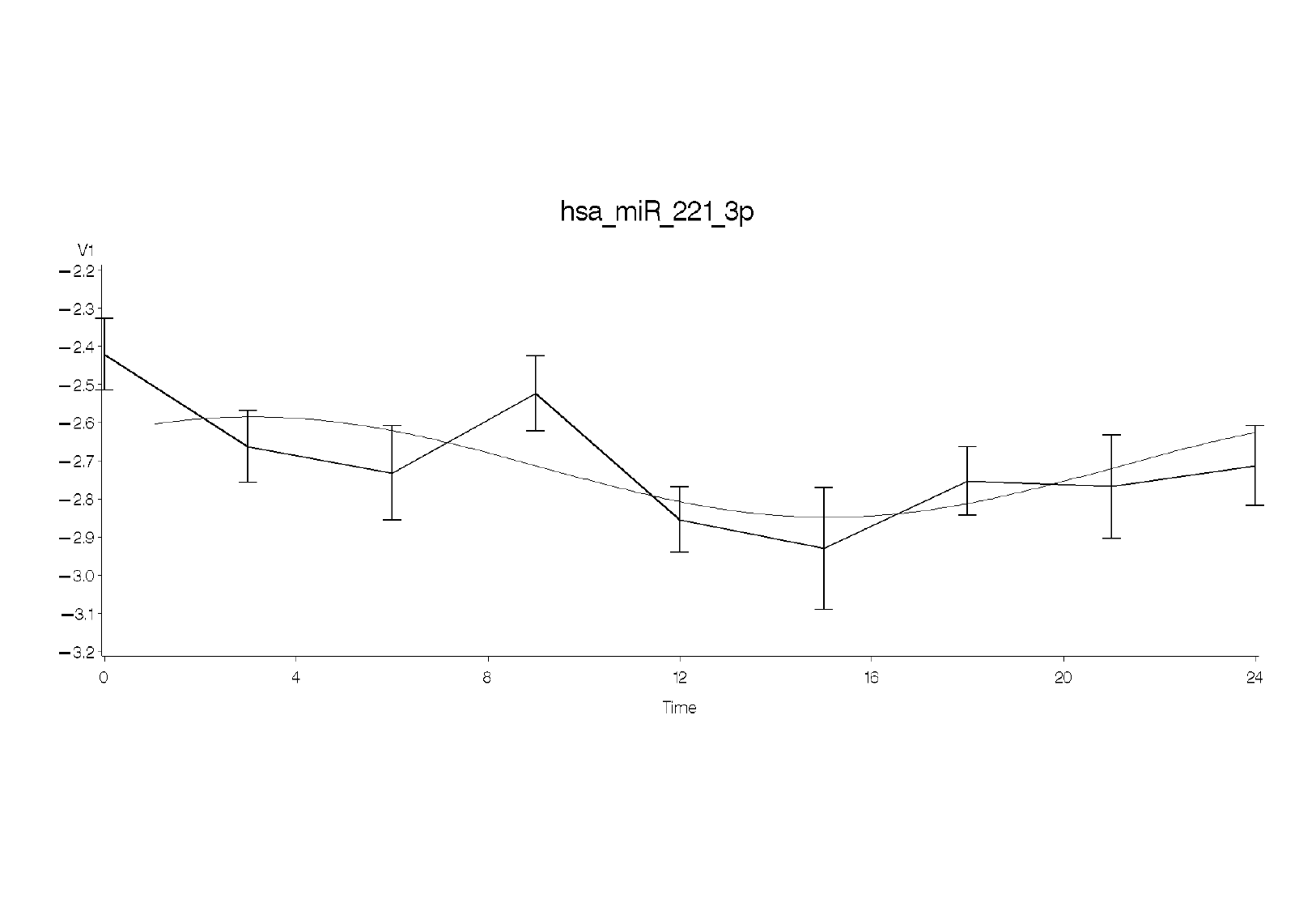

## Slide 28
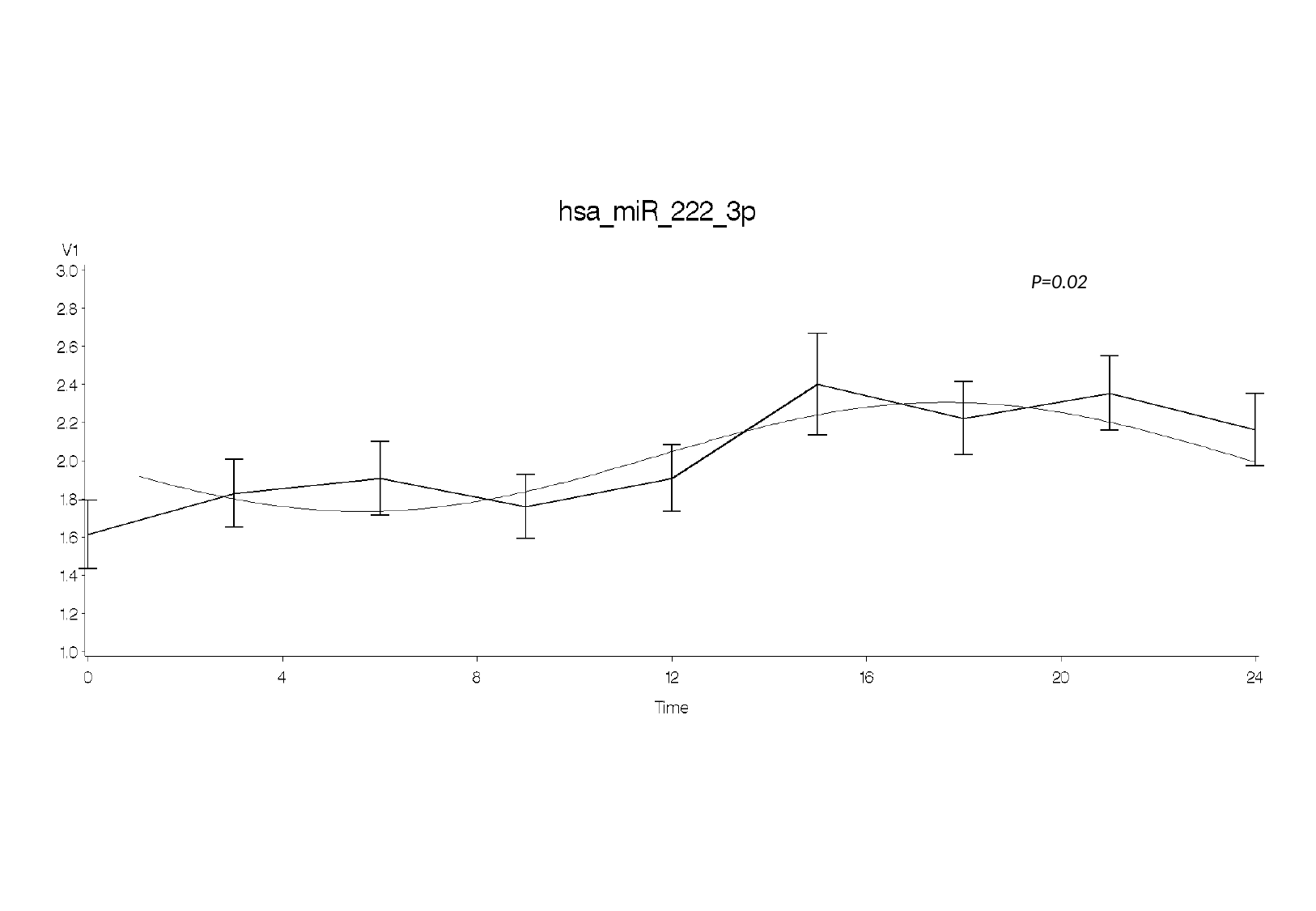

P=0.02

## Slide 29
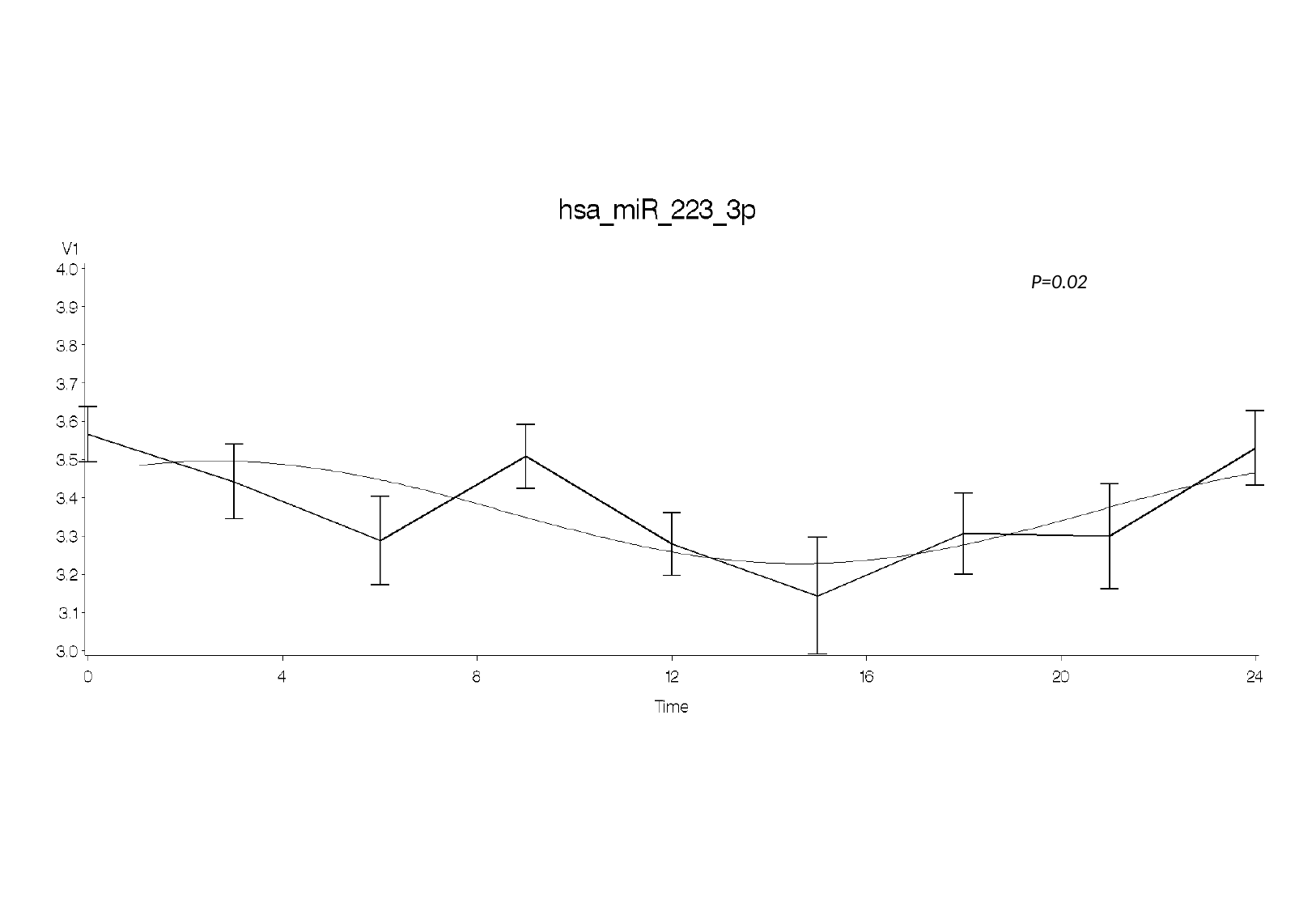

P=0.02

## Slide 30
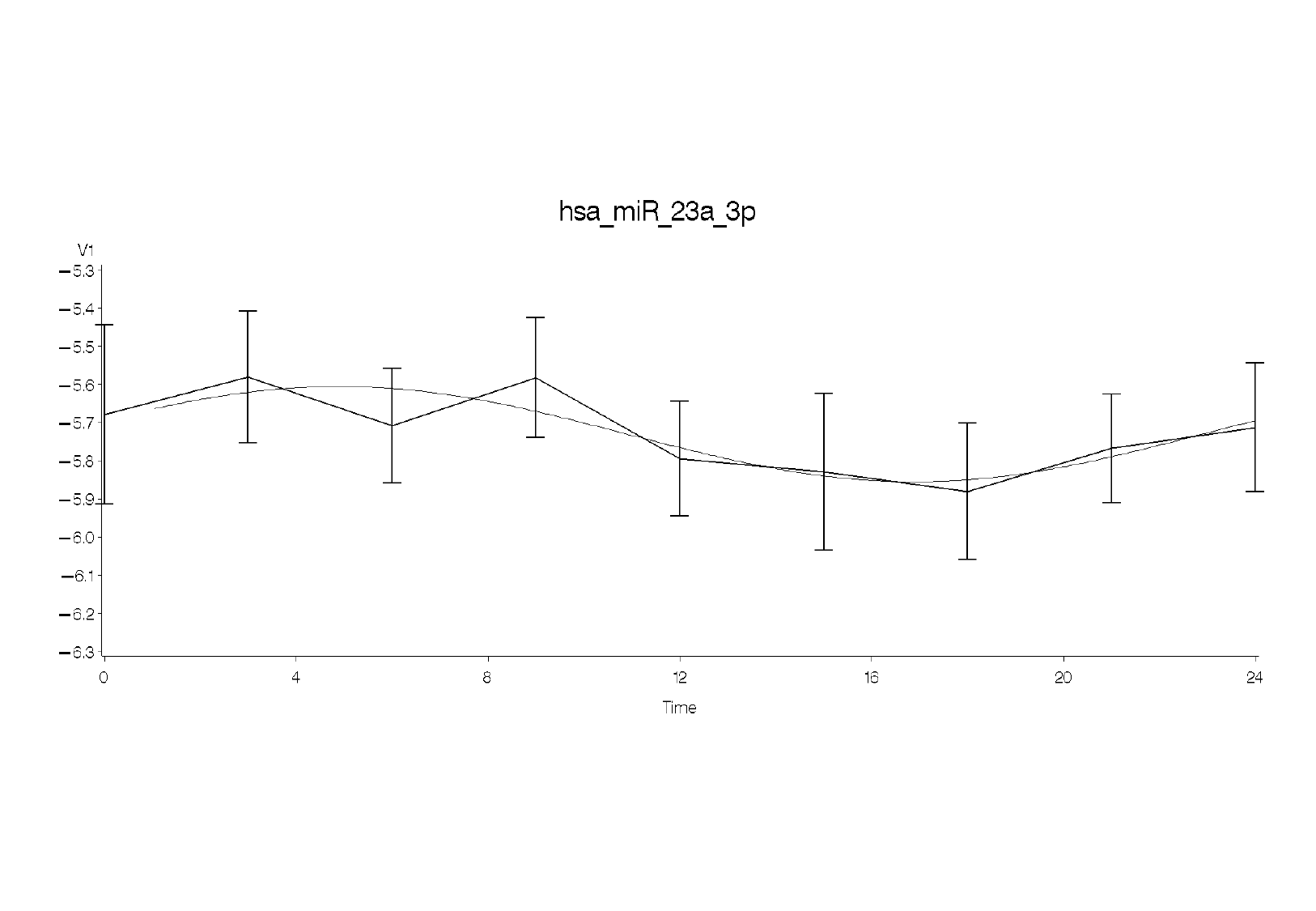

## Slide 31
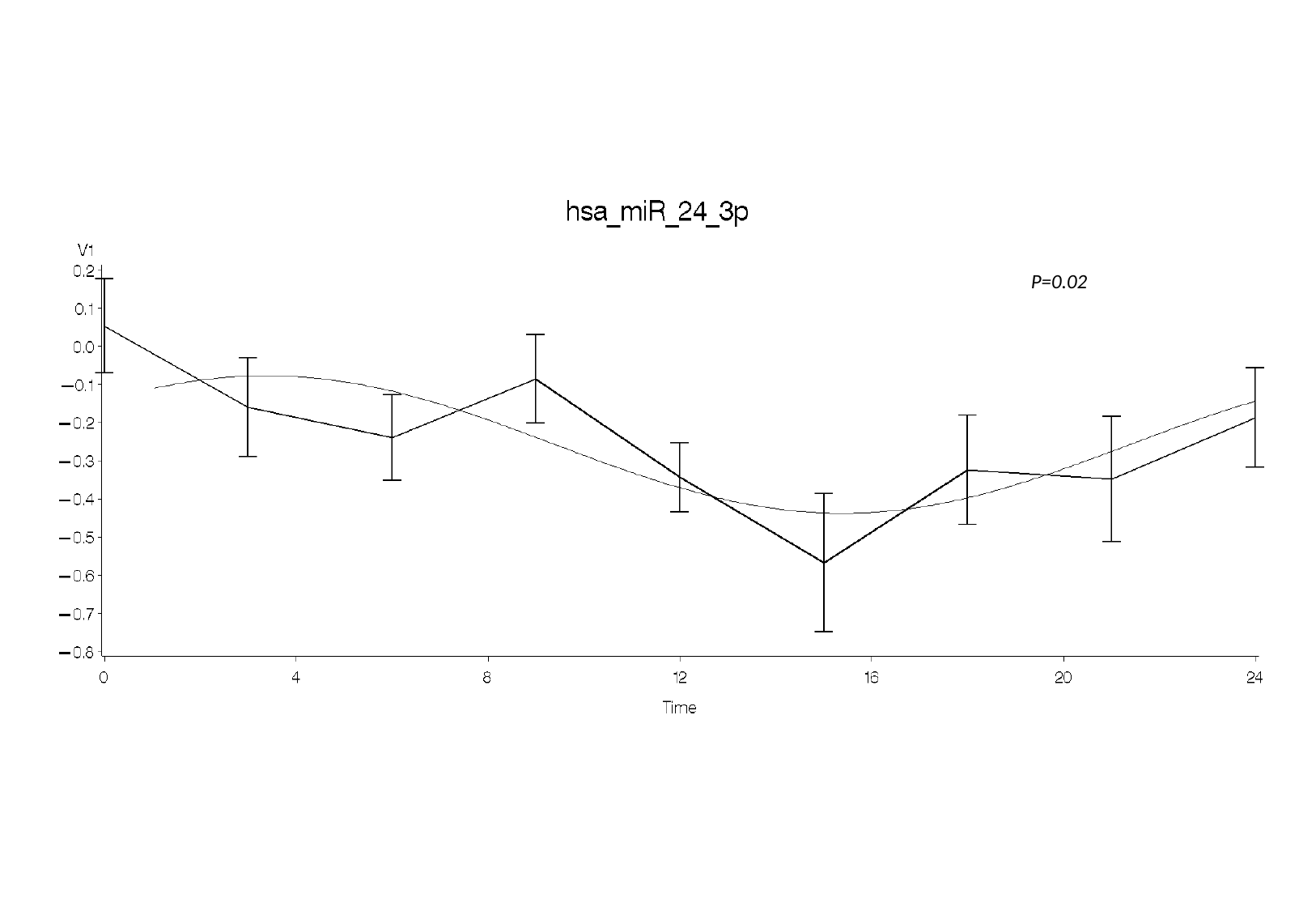

P=0.02

## Slide 32
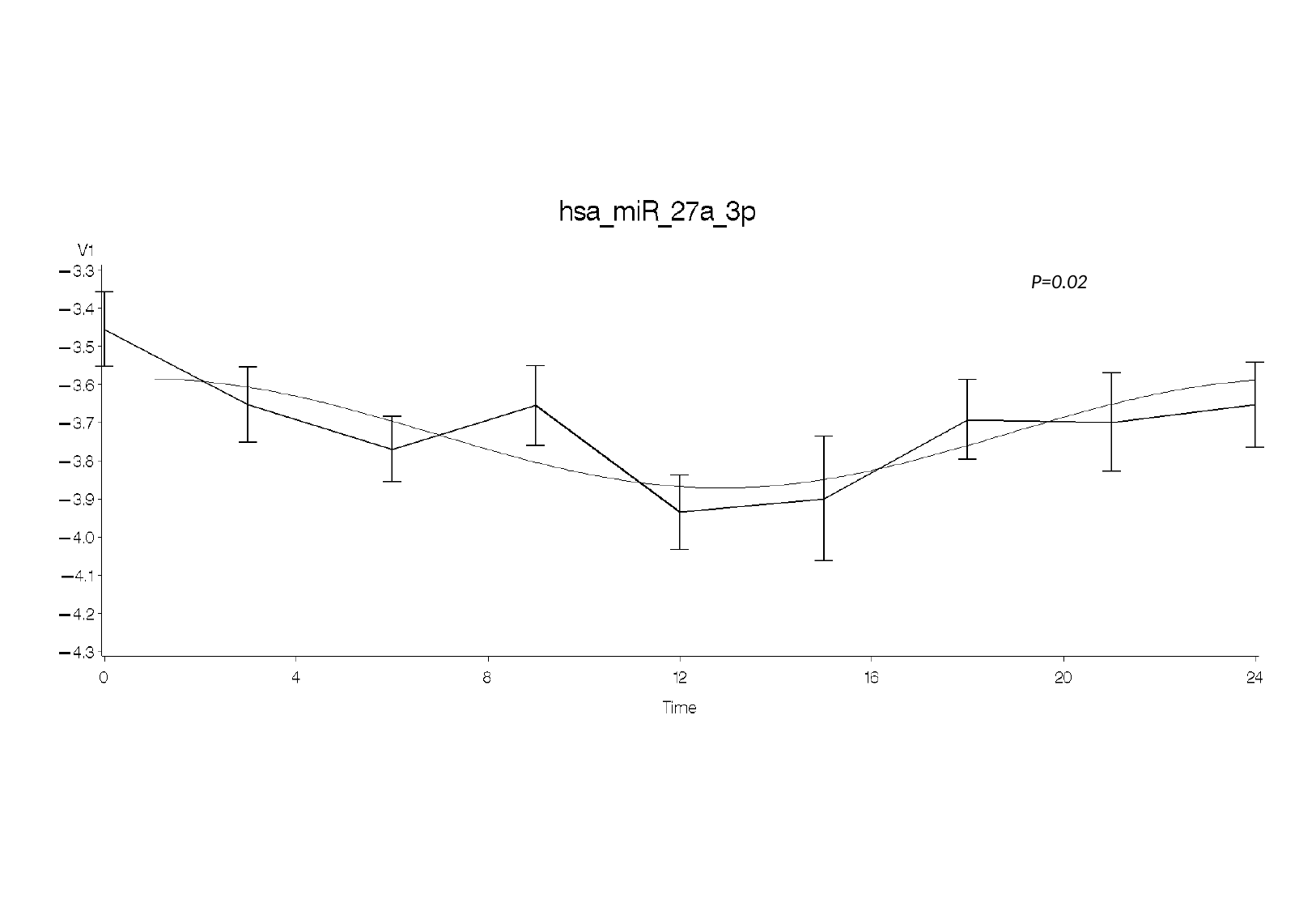

P=0.02

## Slide 33
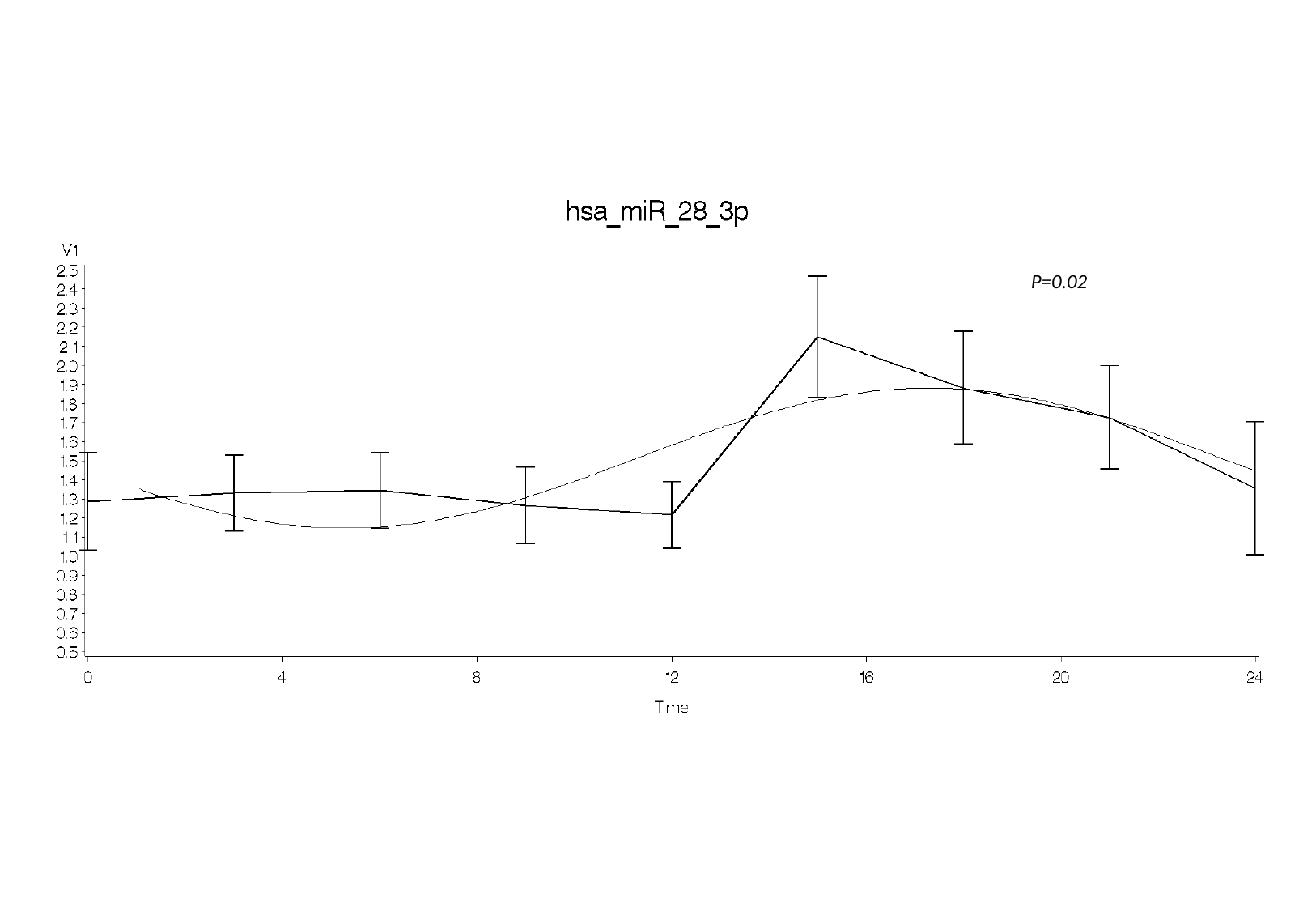

P=0.02

## Slide 34
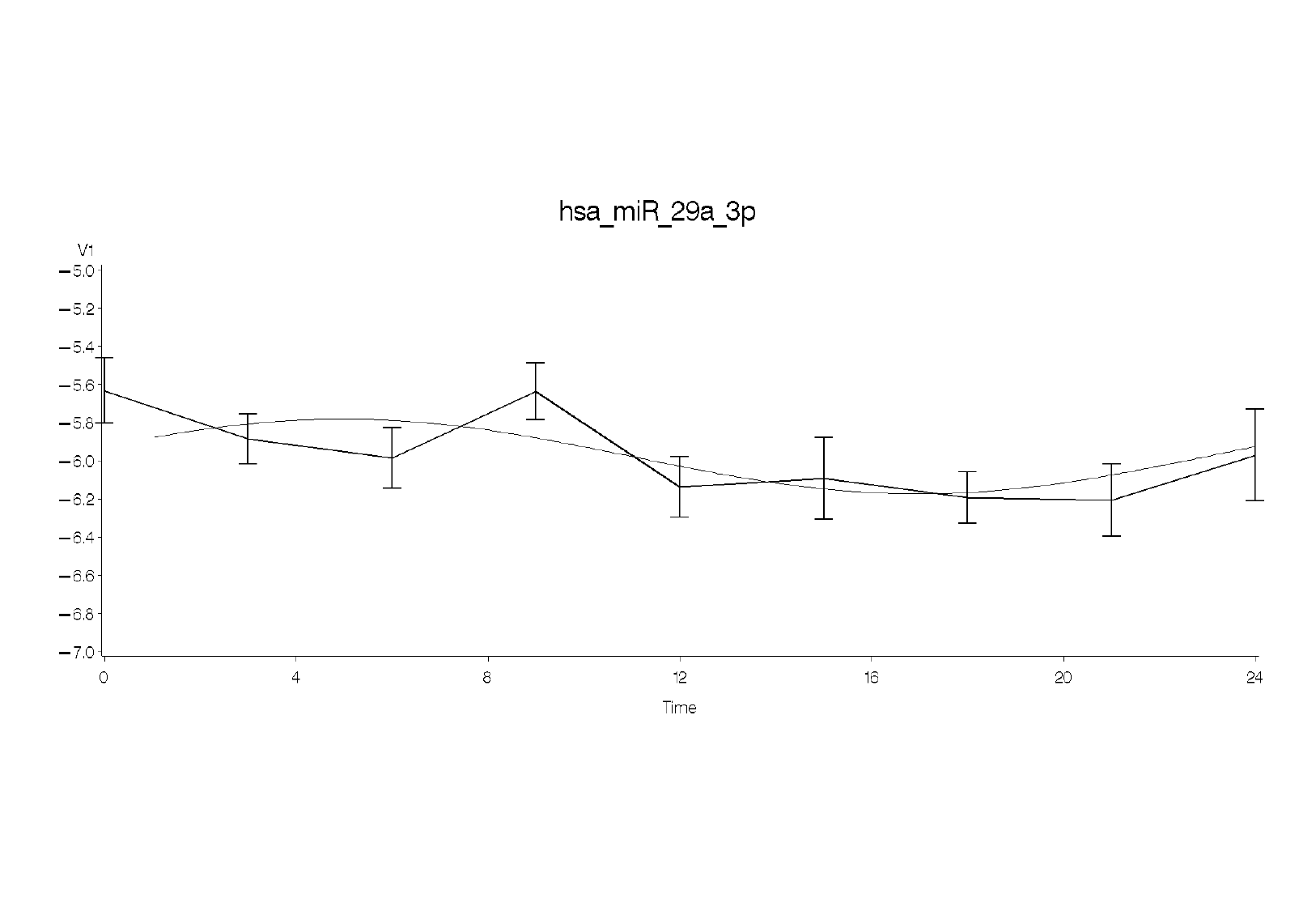

## Slide 35
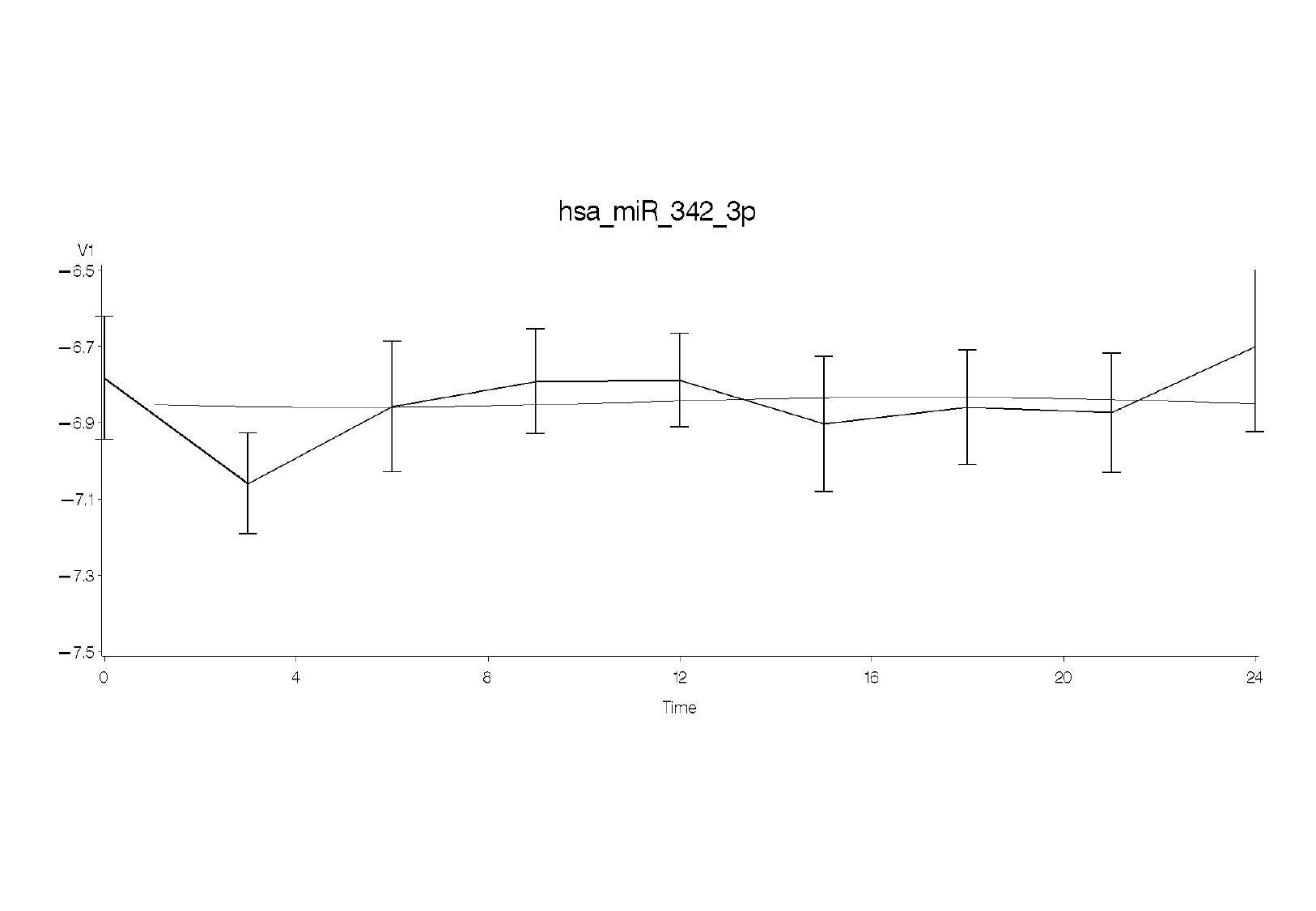

## Slide 36
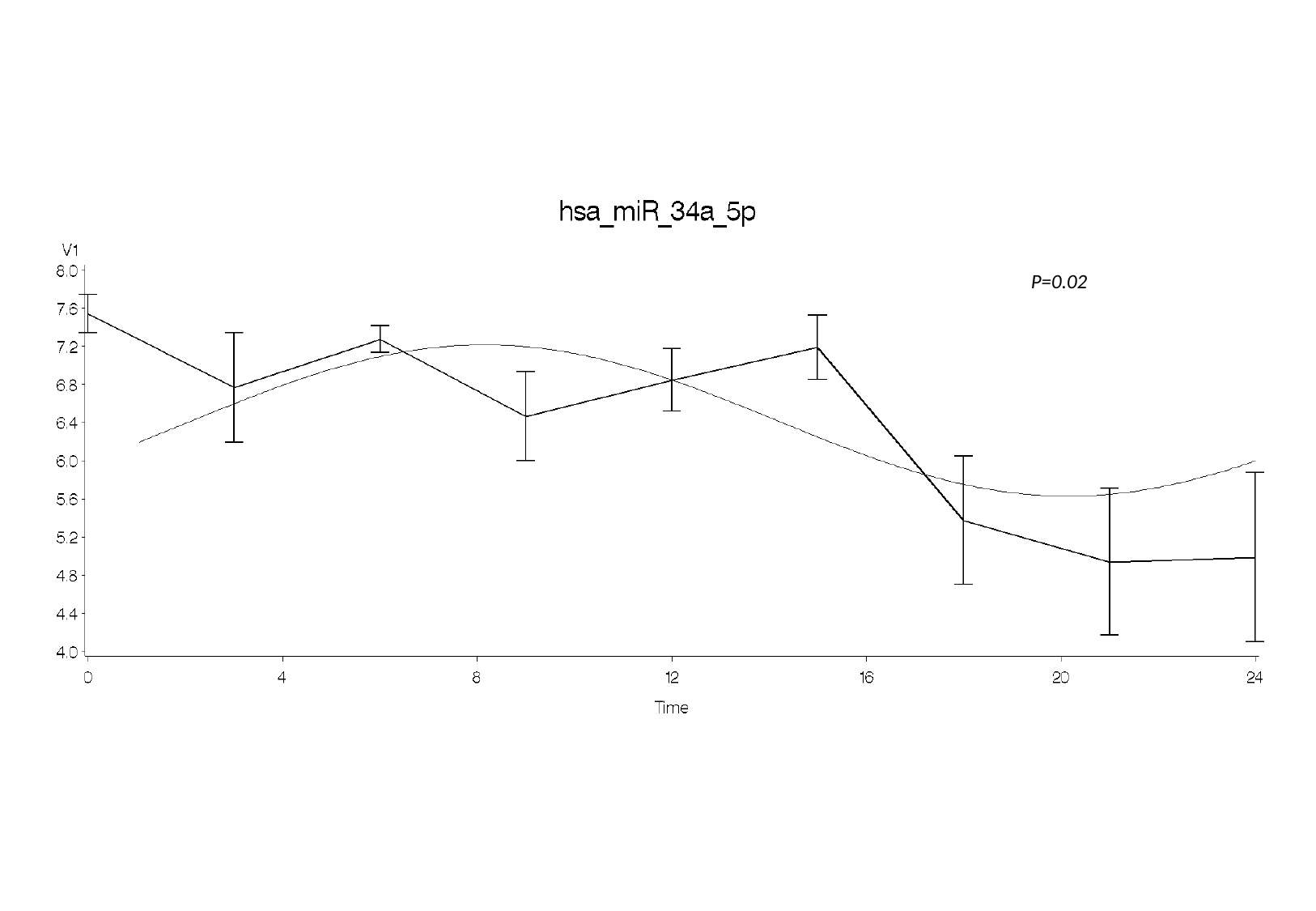

P=0.02

## Slide 37
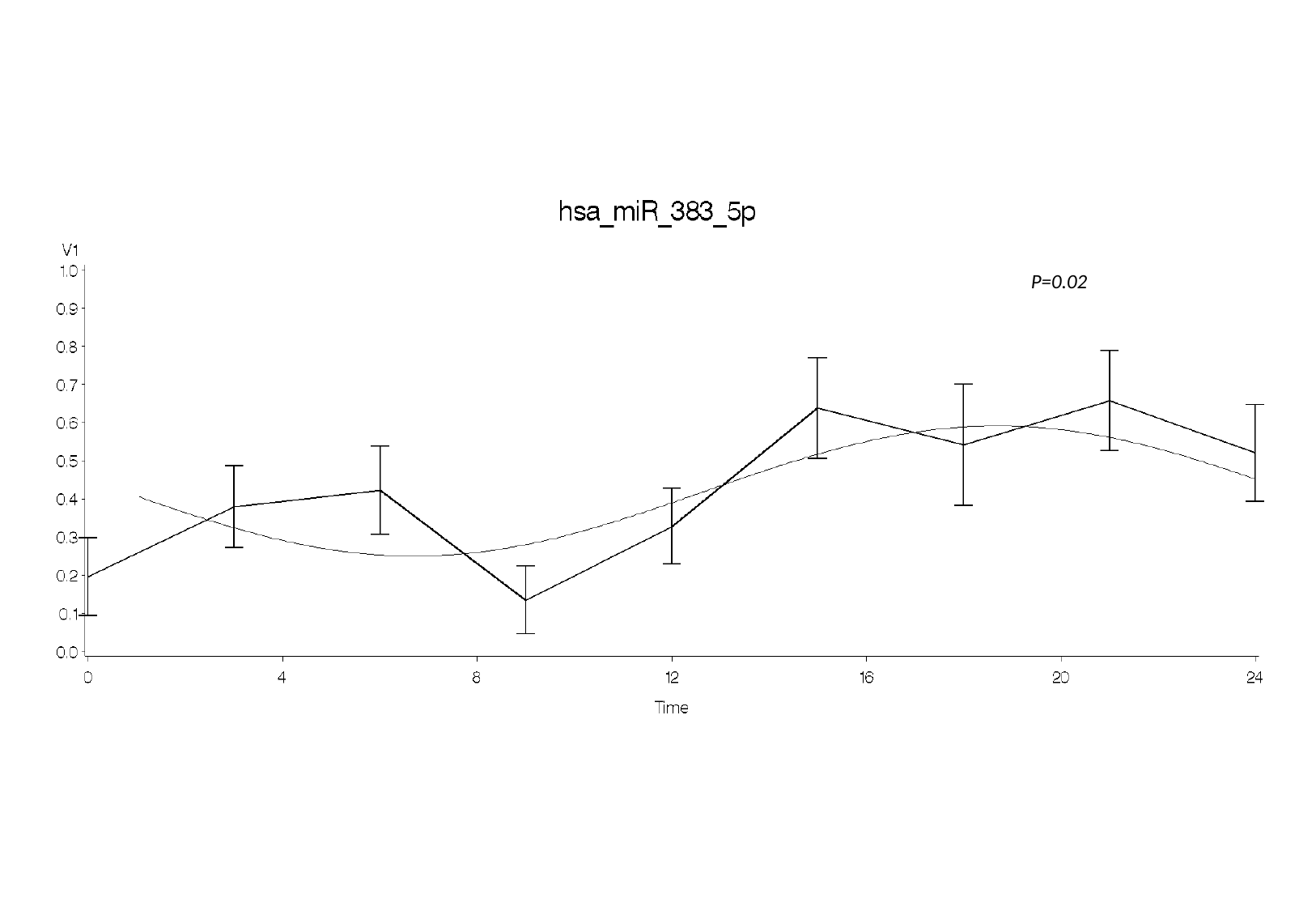

P=0.02

## Slide 38
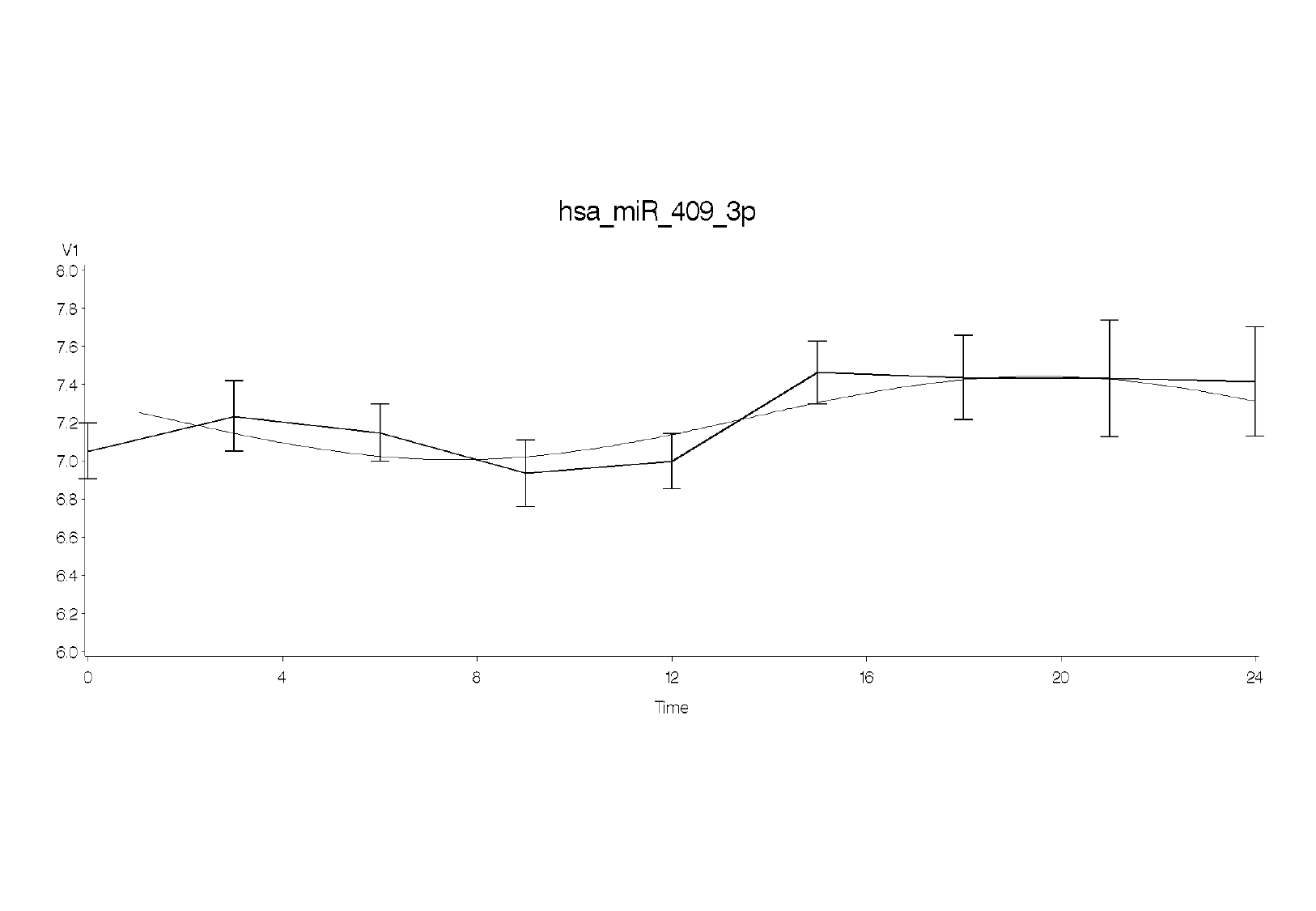

## Slide 39
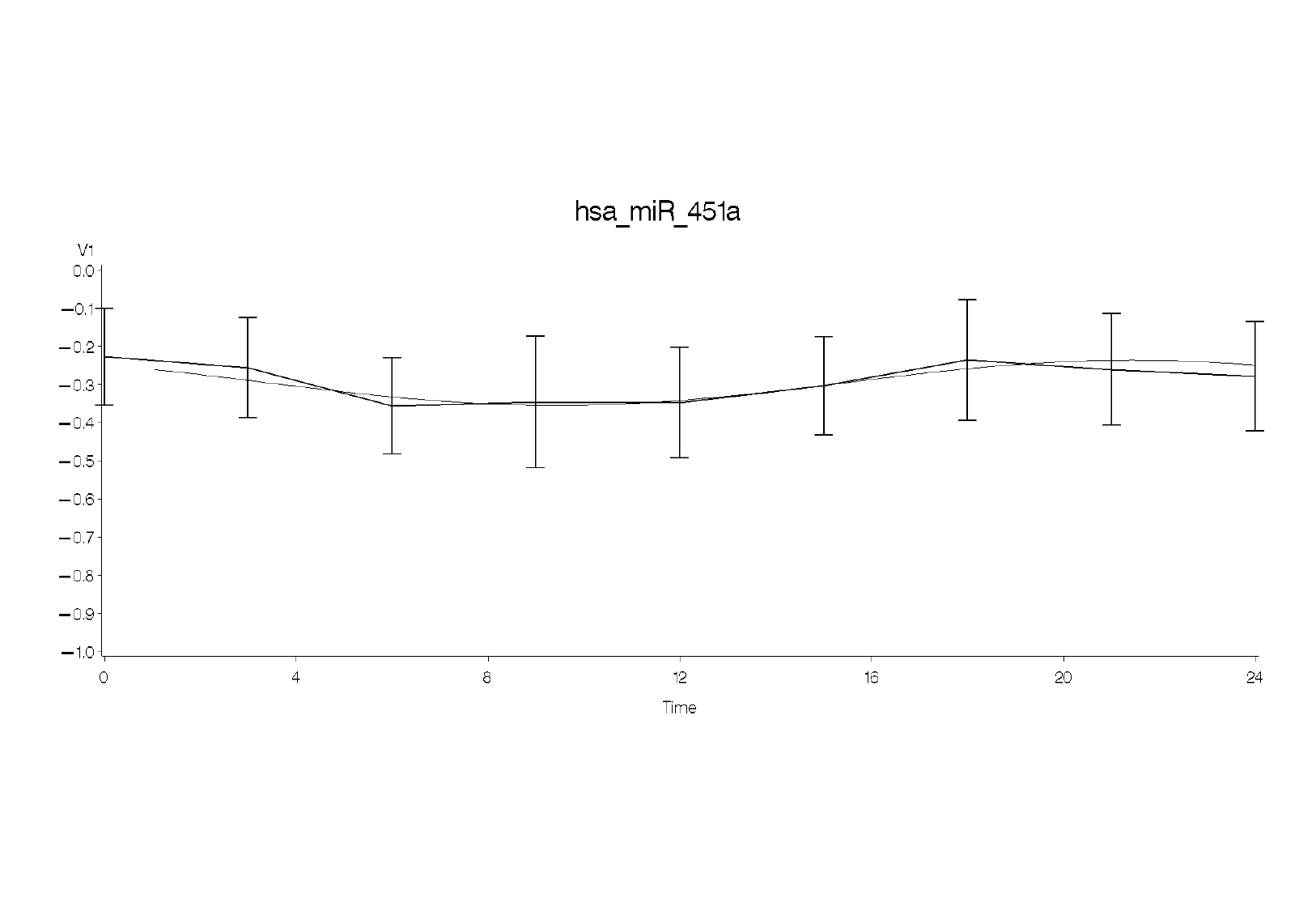

## Slide 40
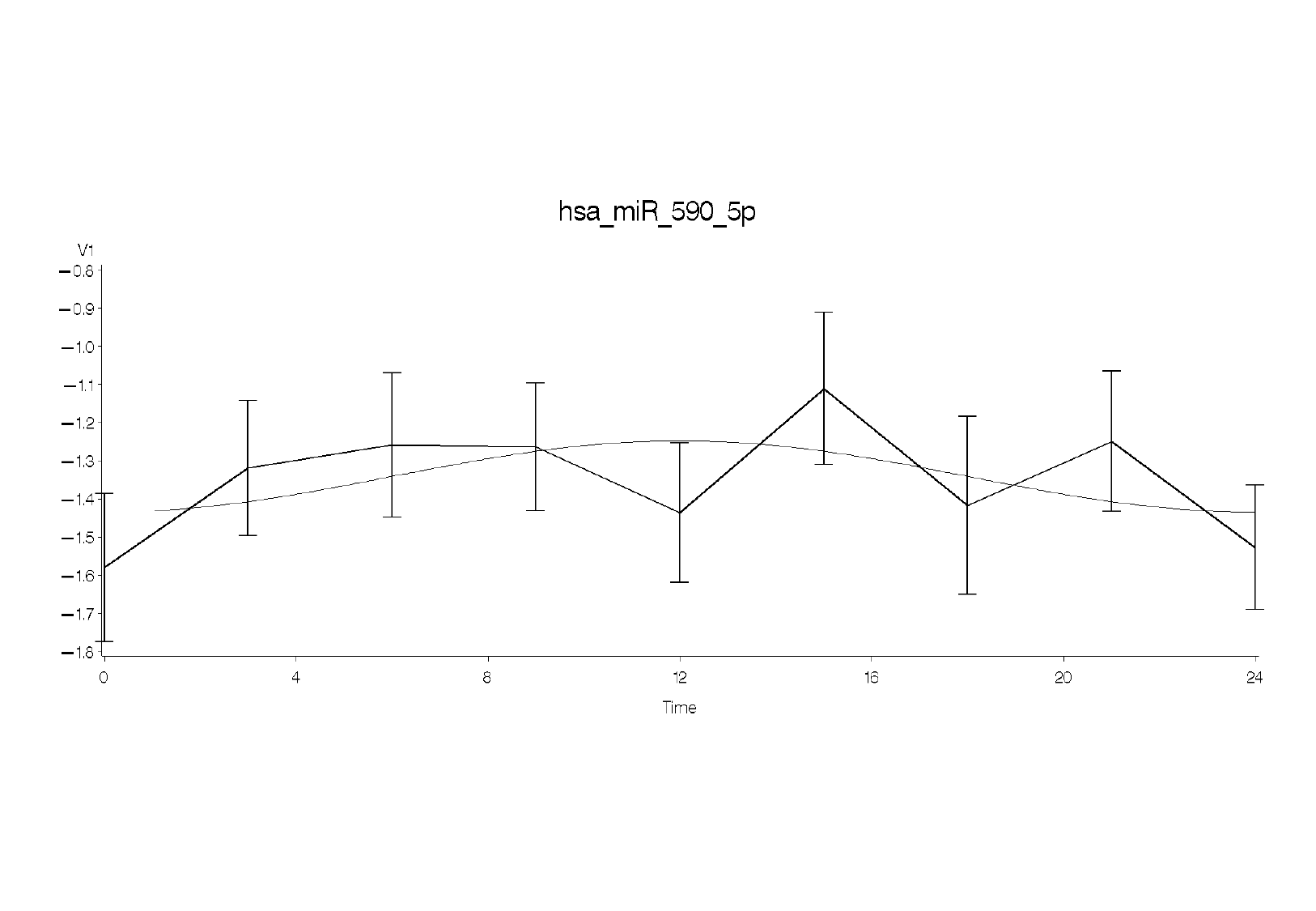

## Slide 41
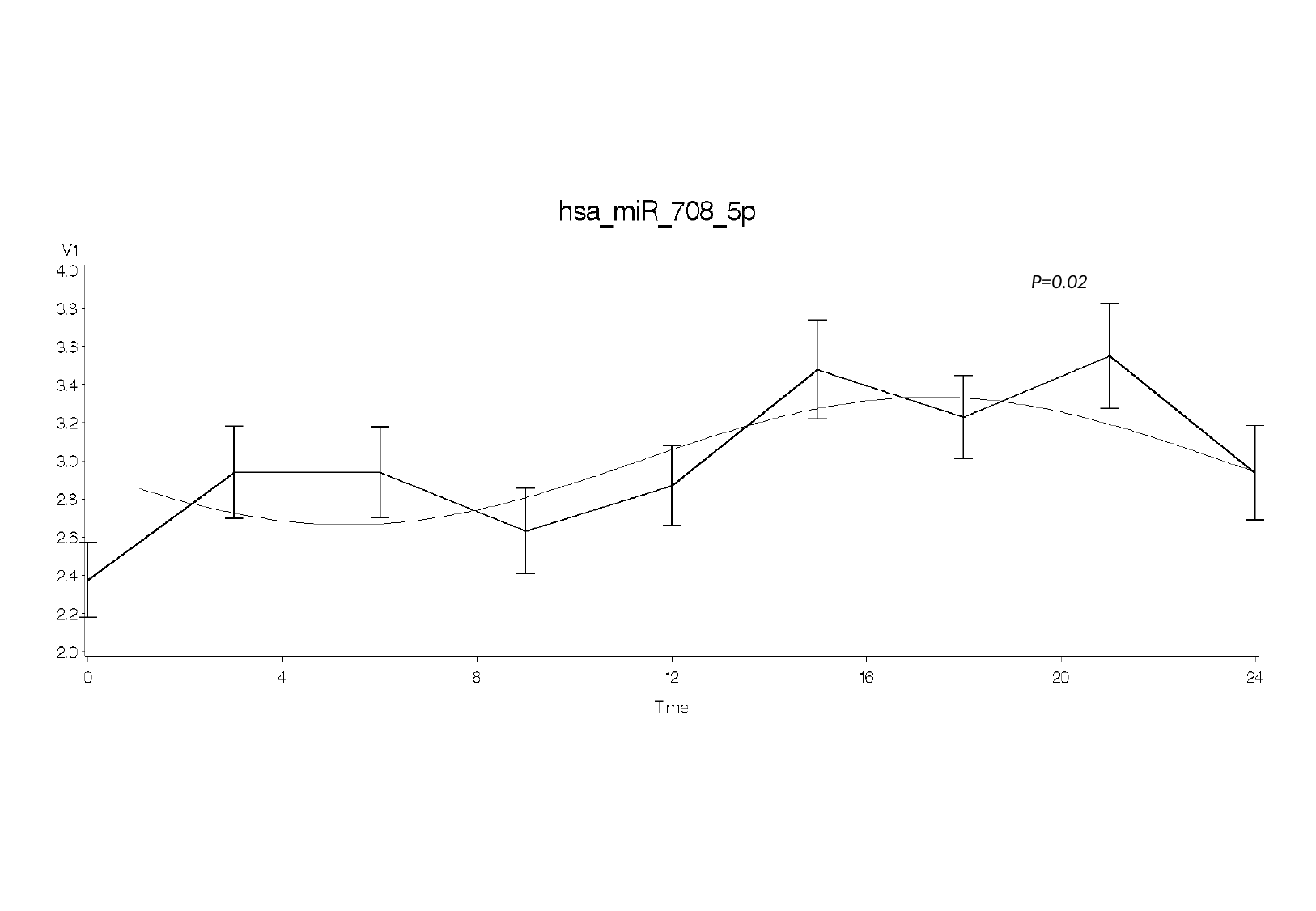

P=0.02

## Slide 42
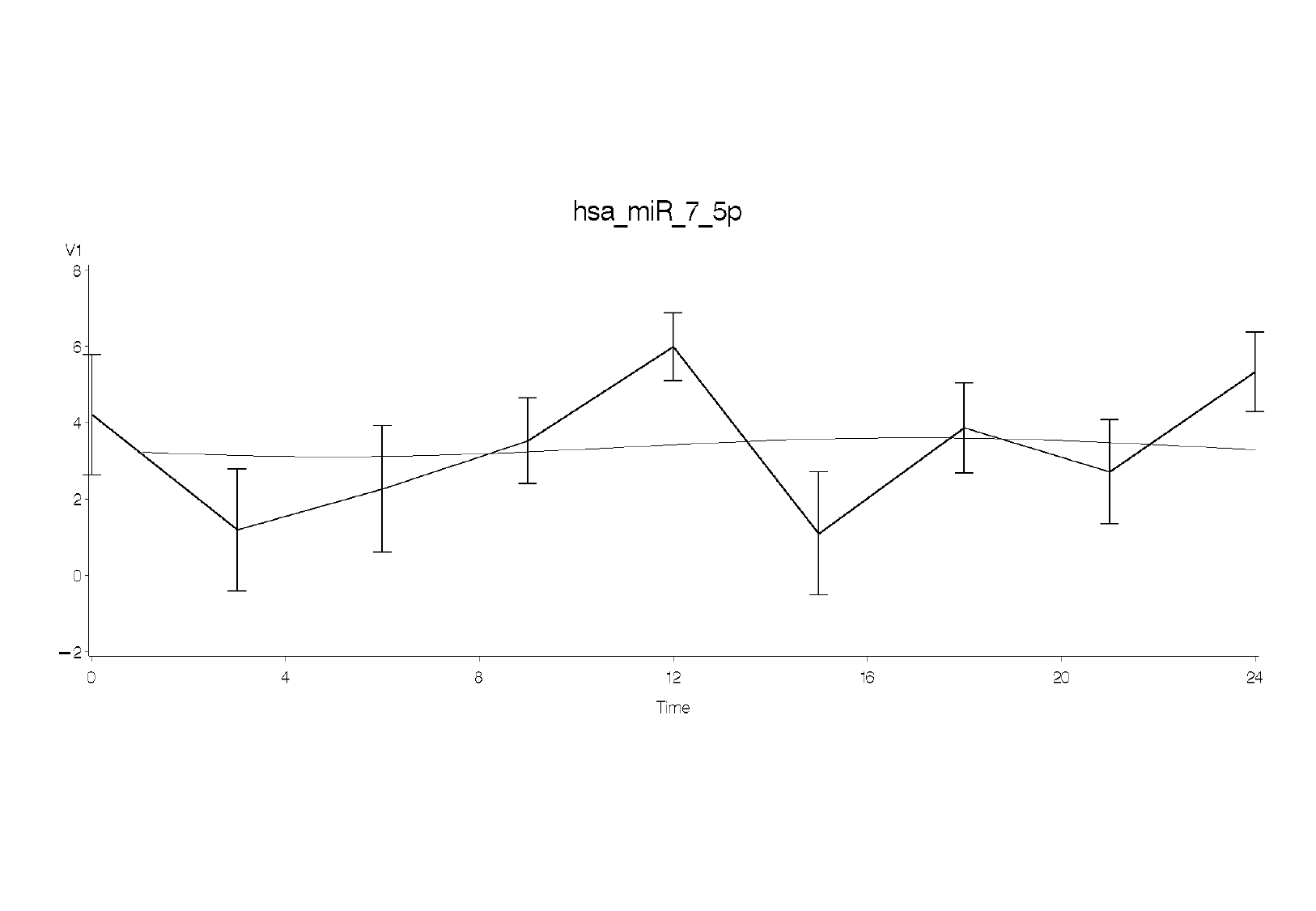

## Slide 43
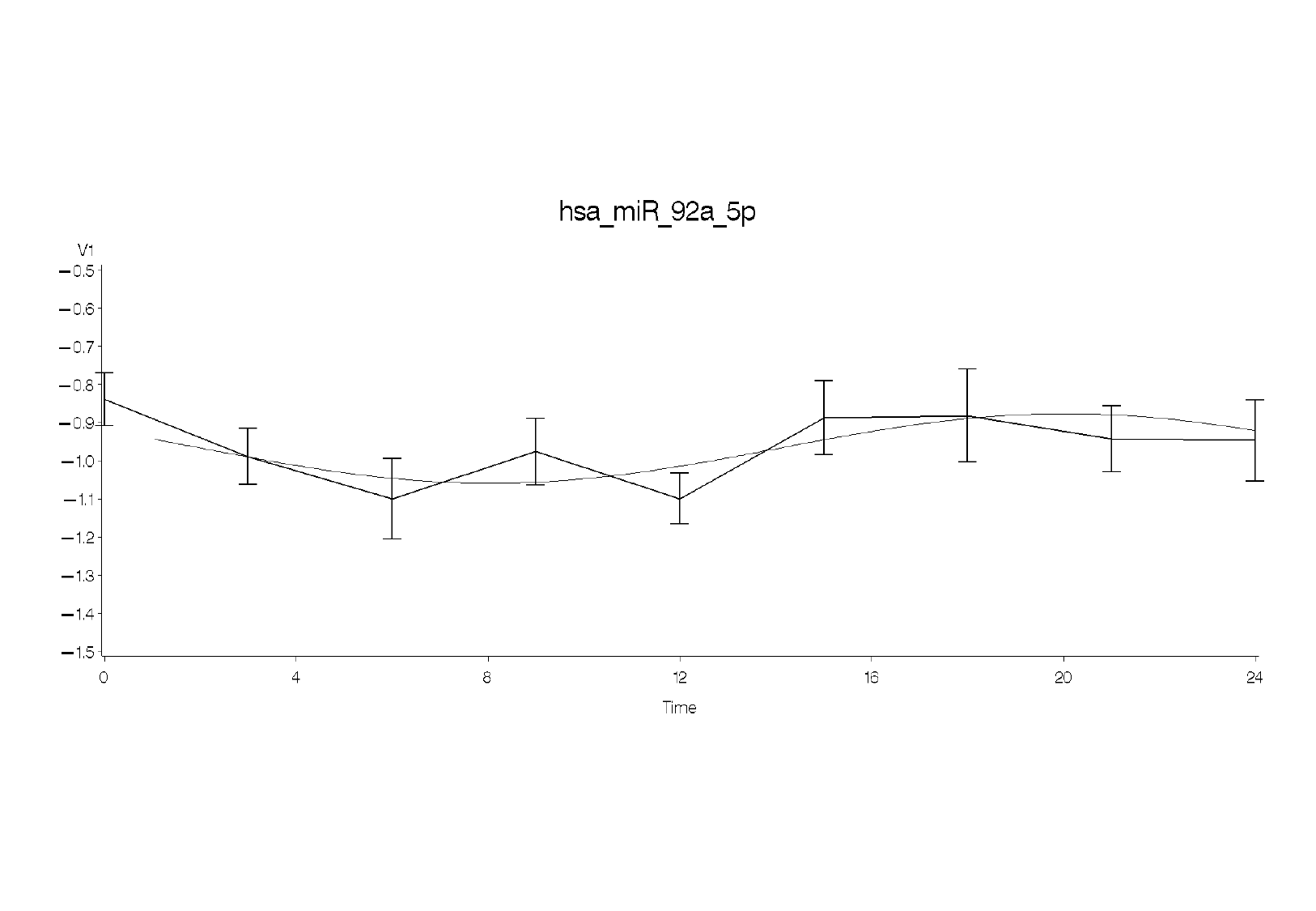

## Slide 44
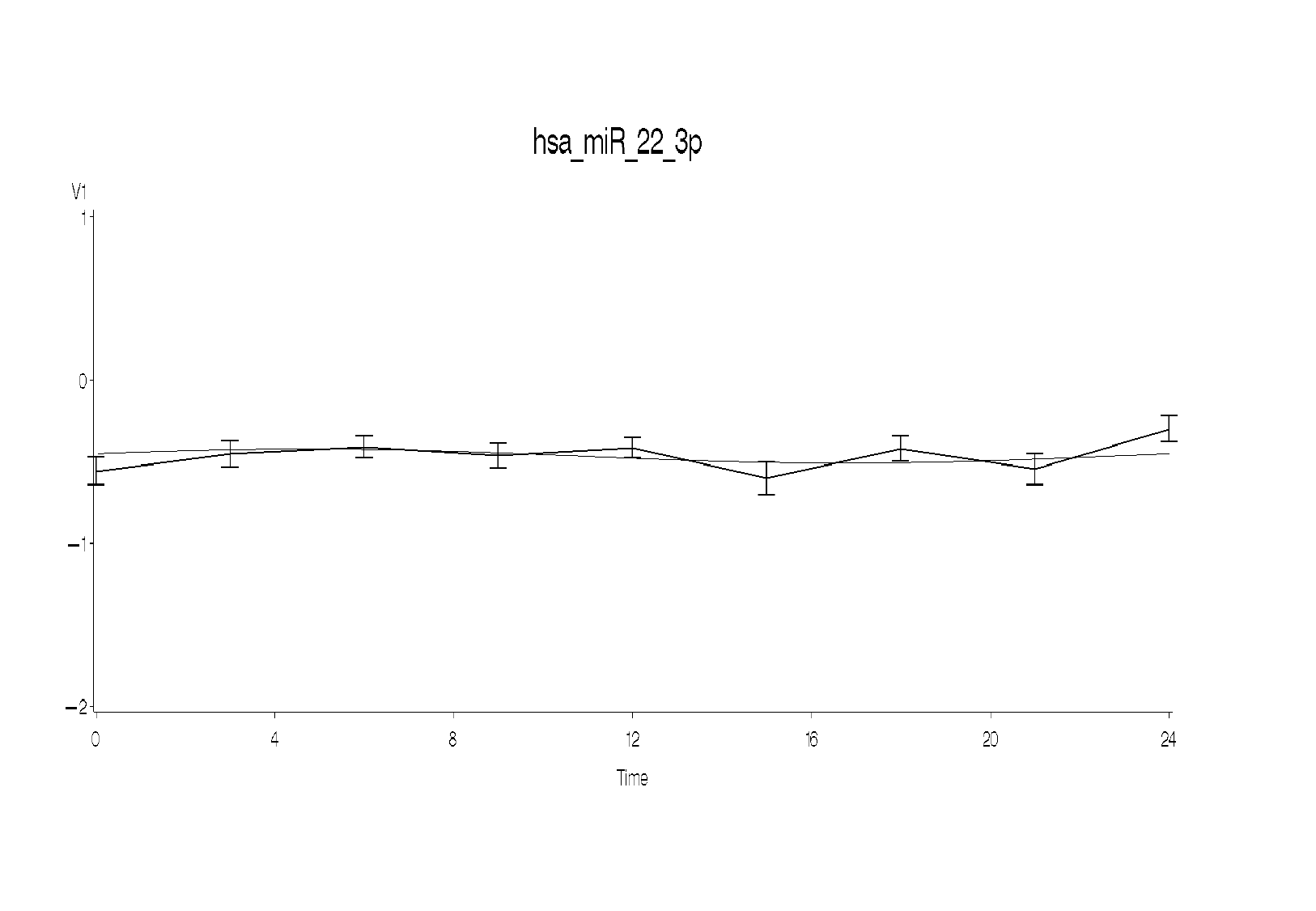

## Slide 45
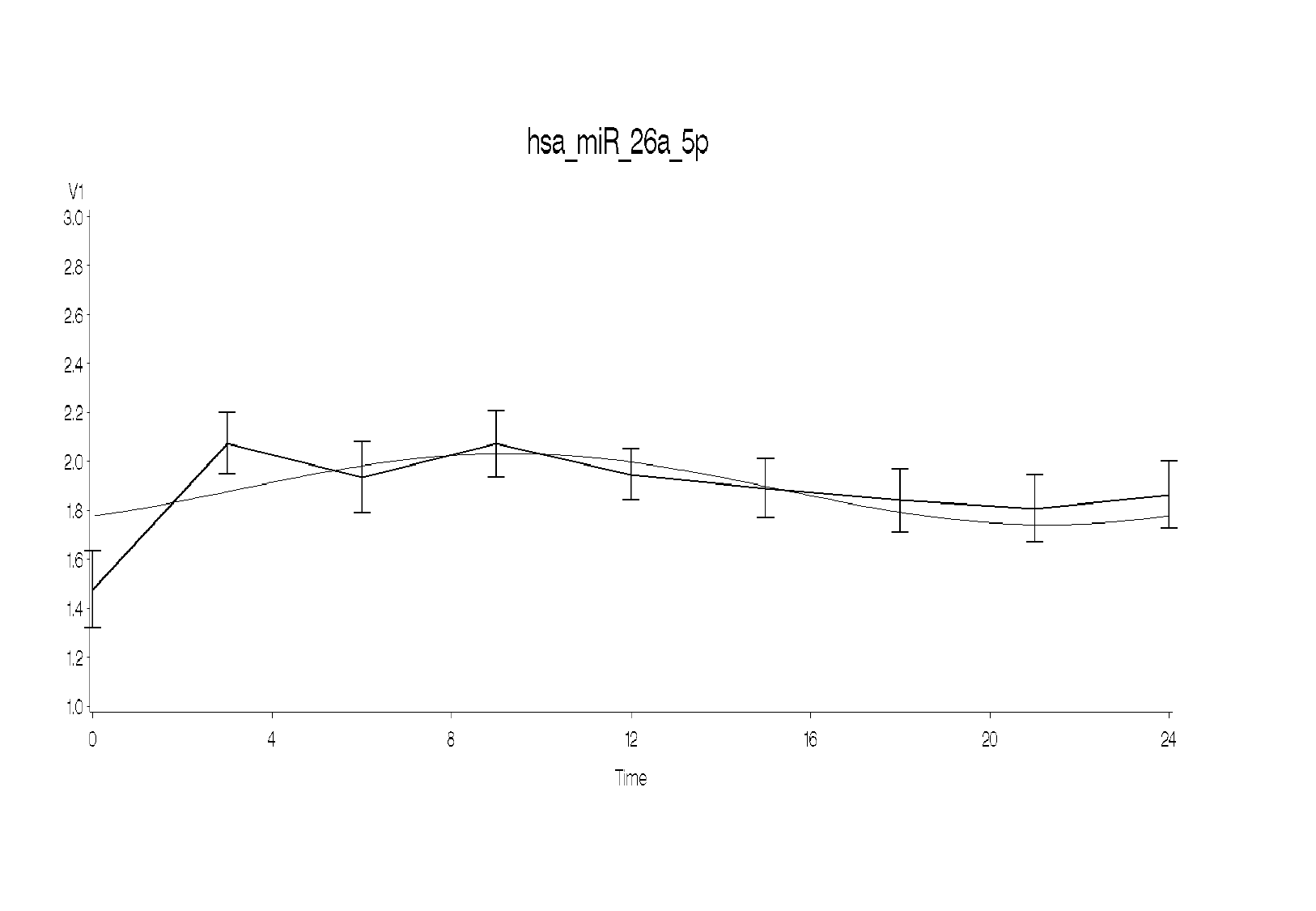

## Slide 46
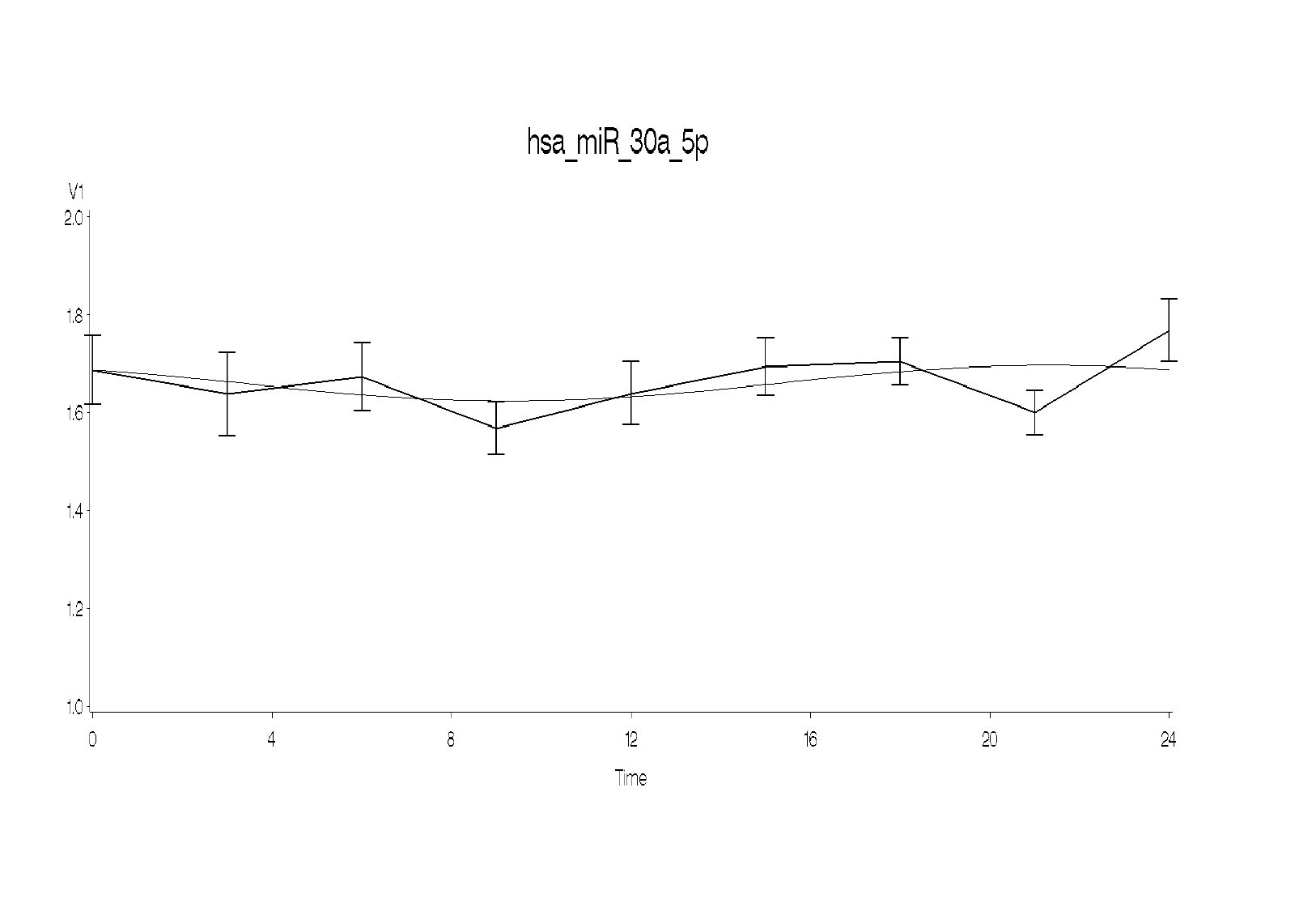

## Slide 47
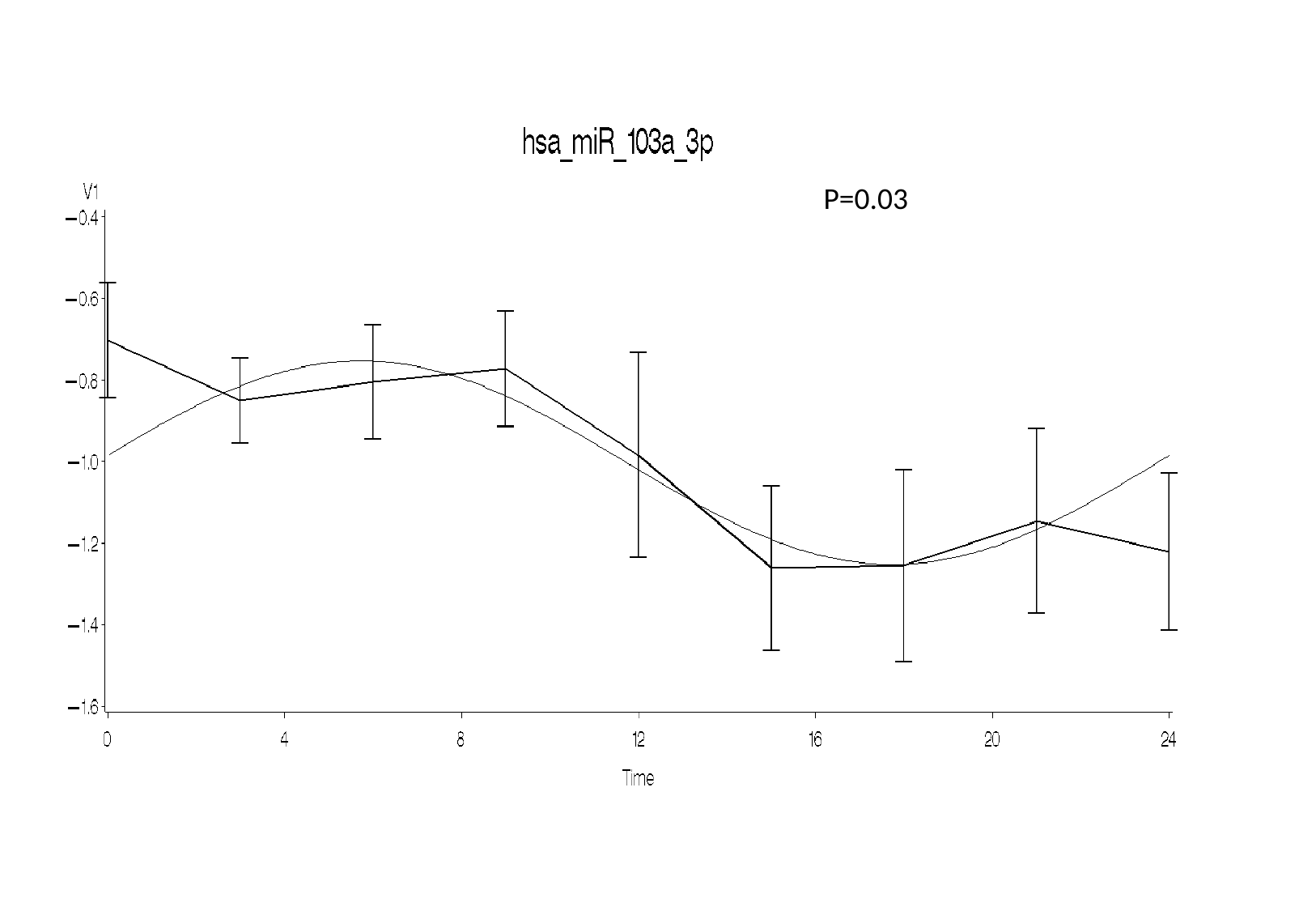

P=0.03

## Slide 48
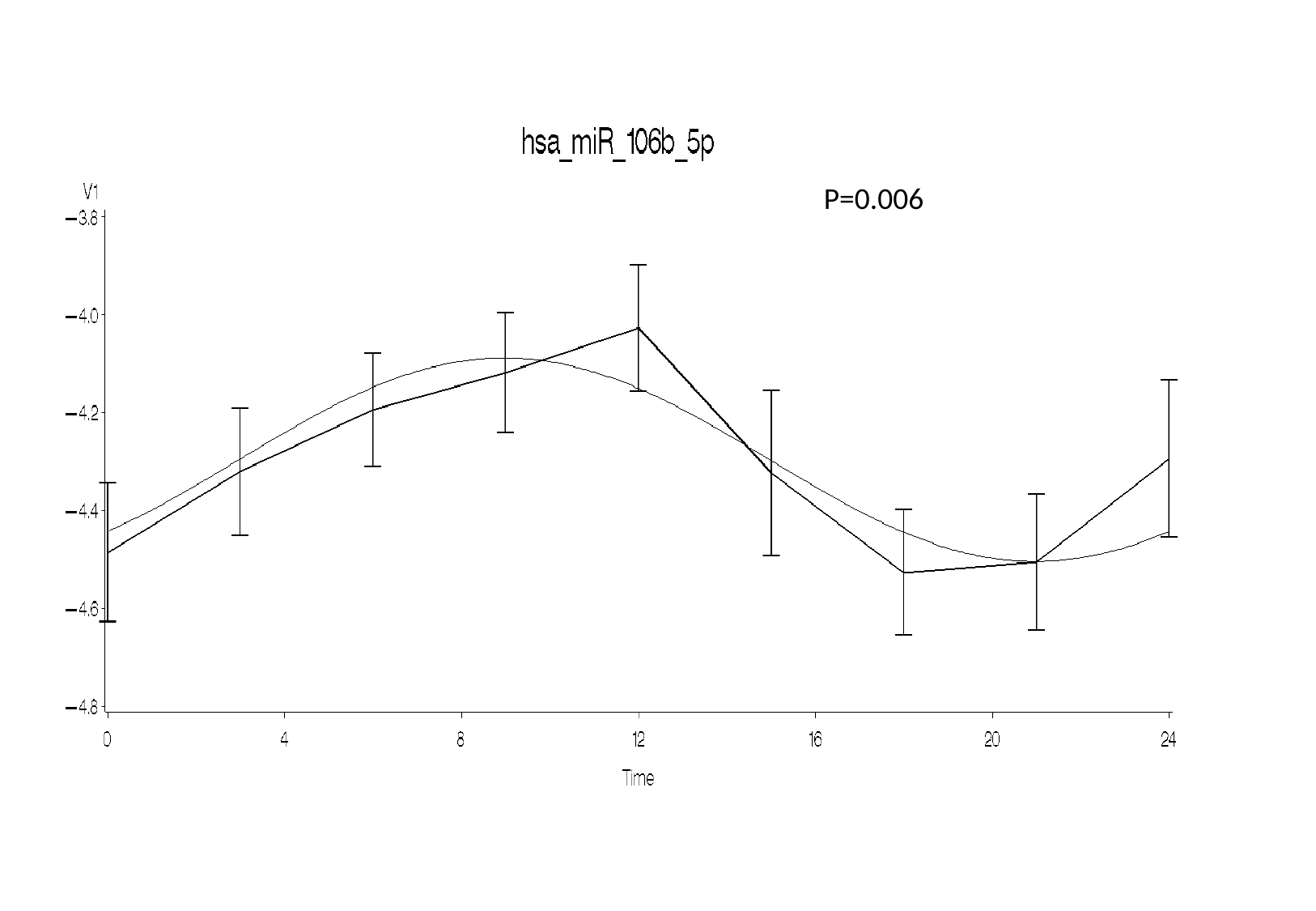

P=0.006

## Slide 49
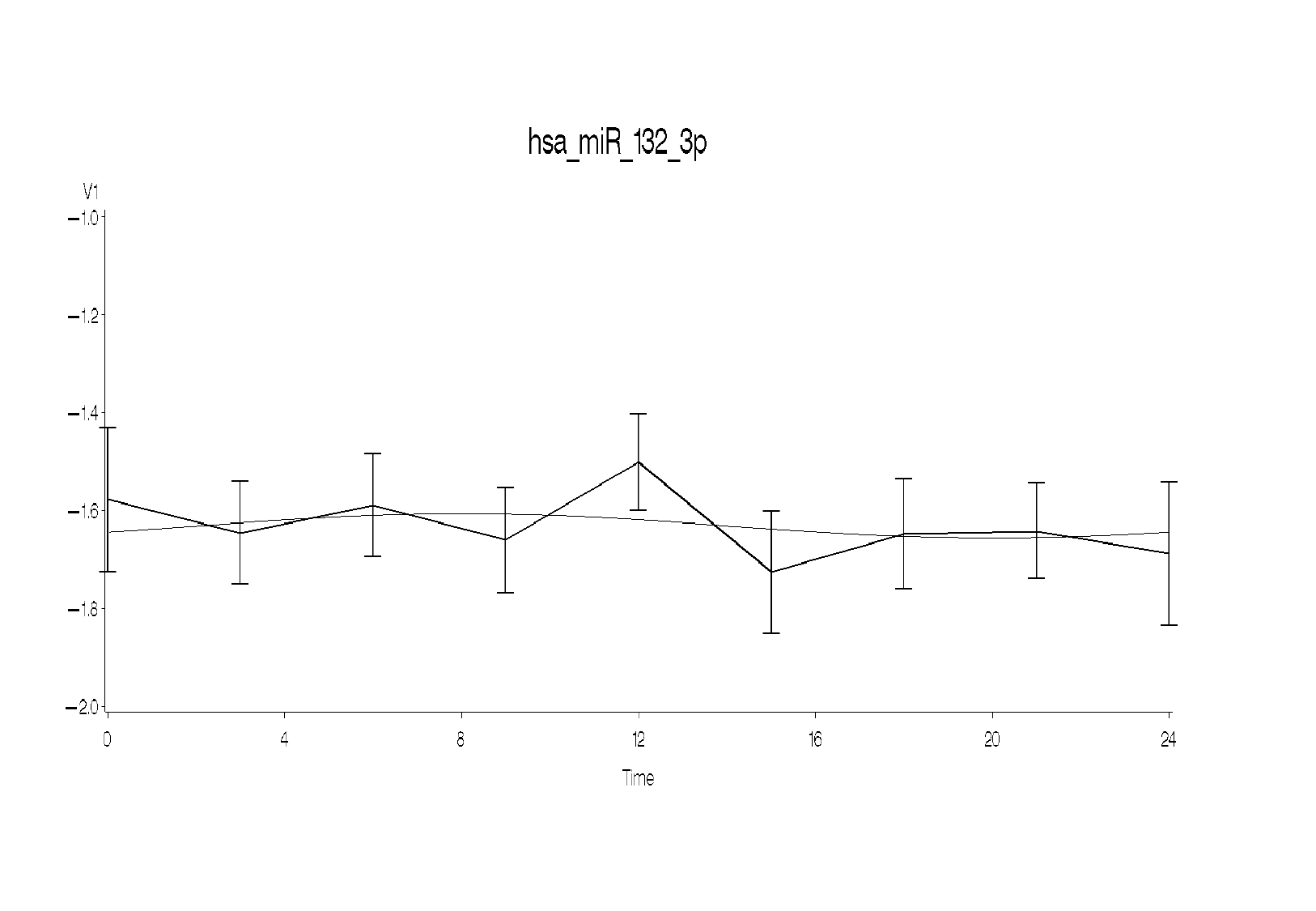

## Slide 50
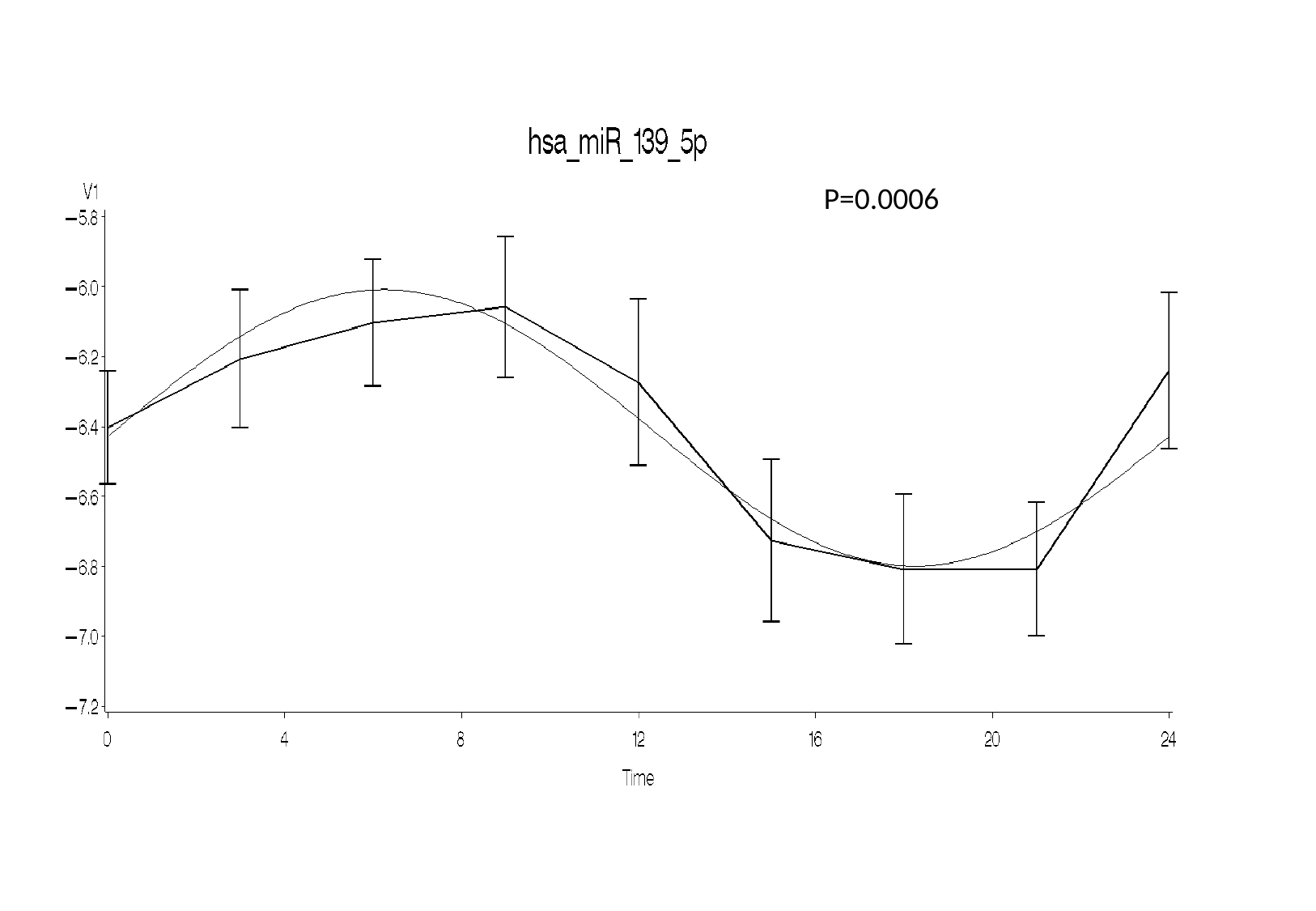

P=0.0006

## Slide 51
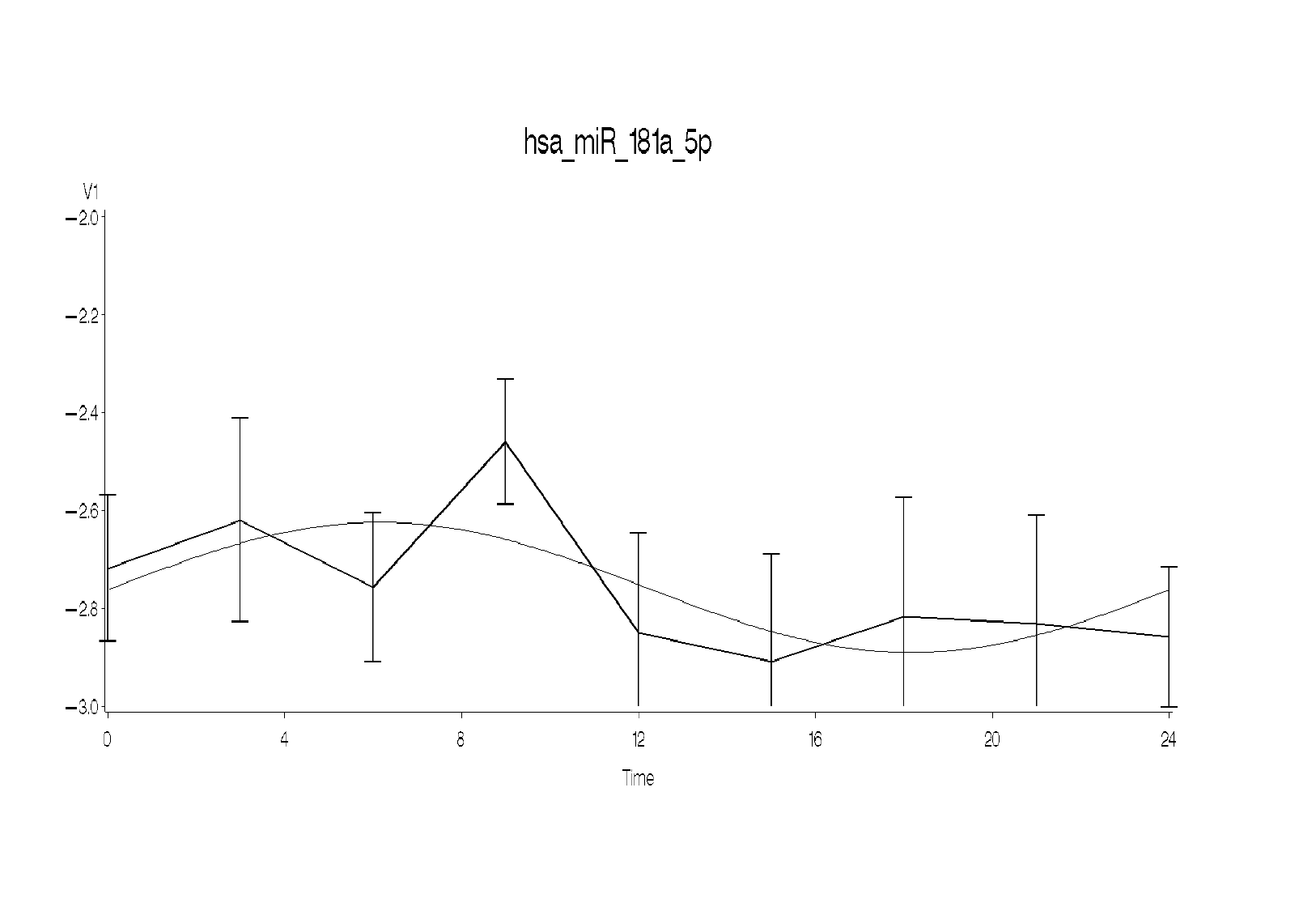

## Slide 52
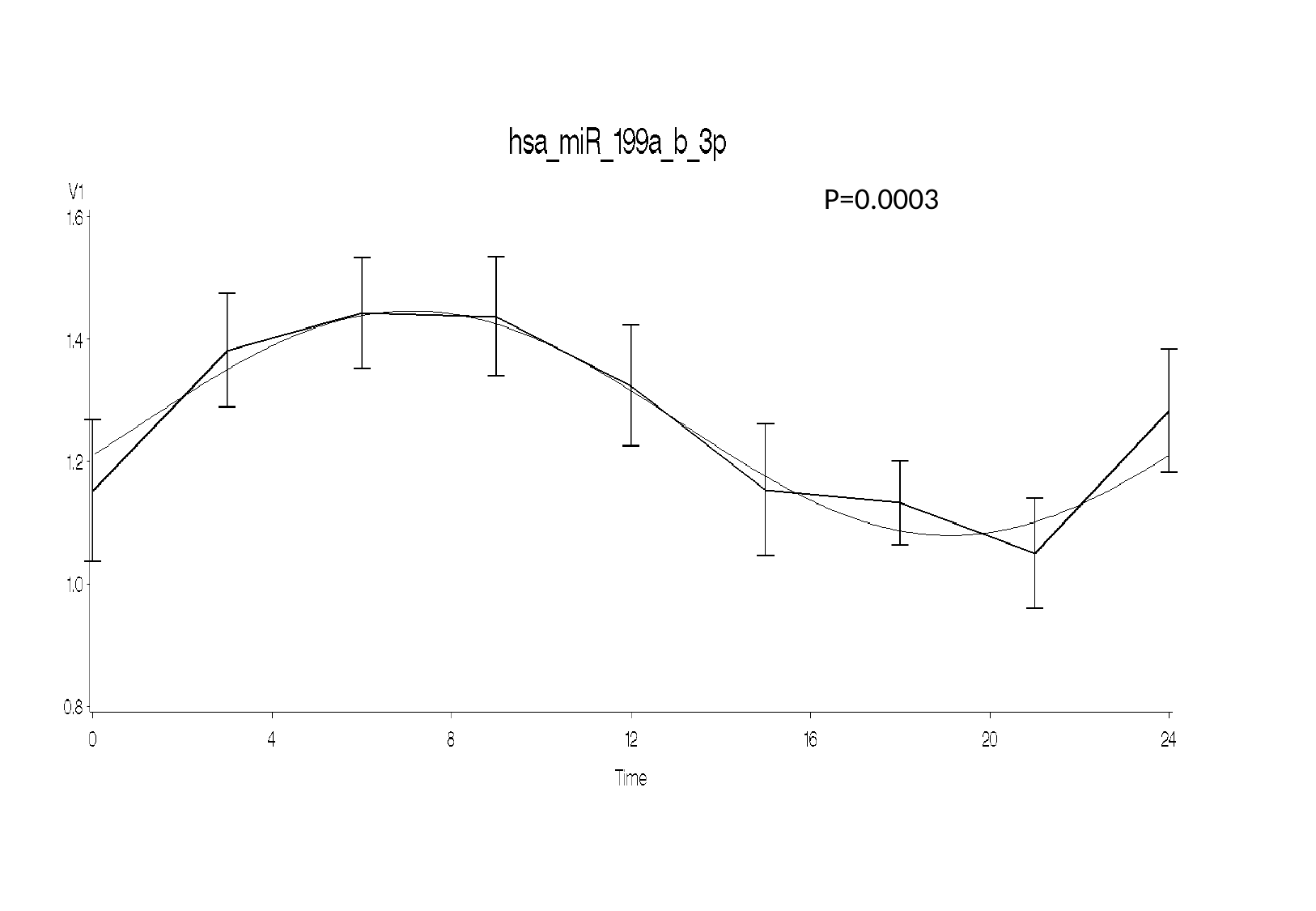

P=0.0003

## Slide 53
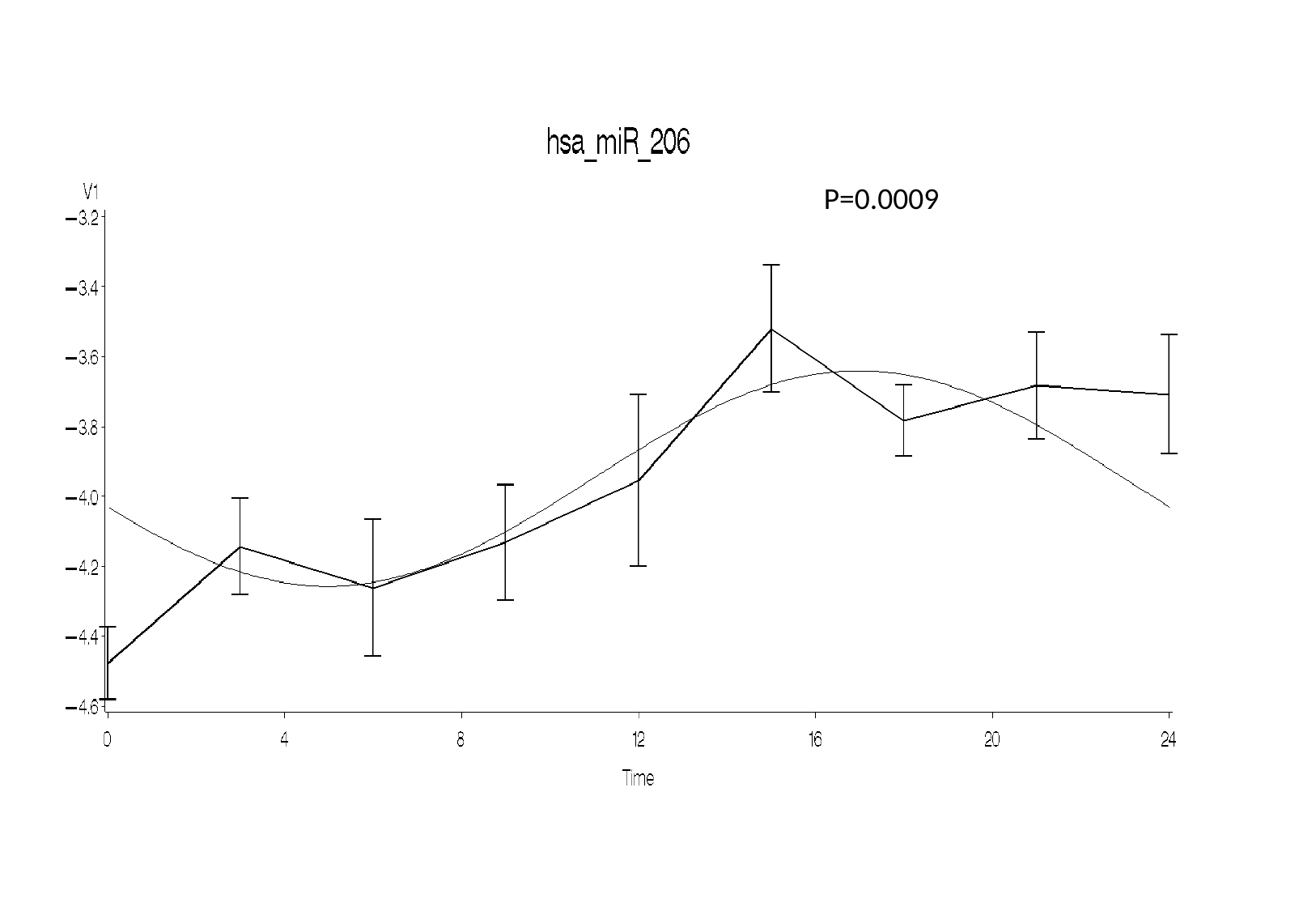

P=0.0009

## Slide 54
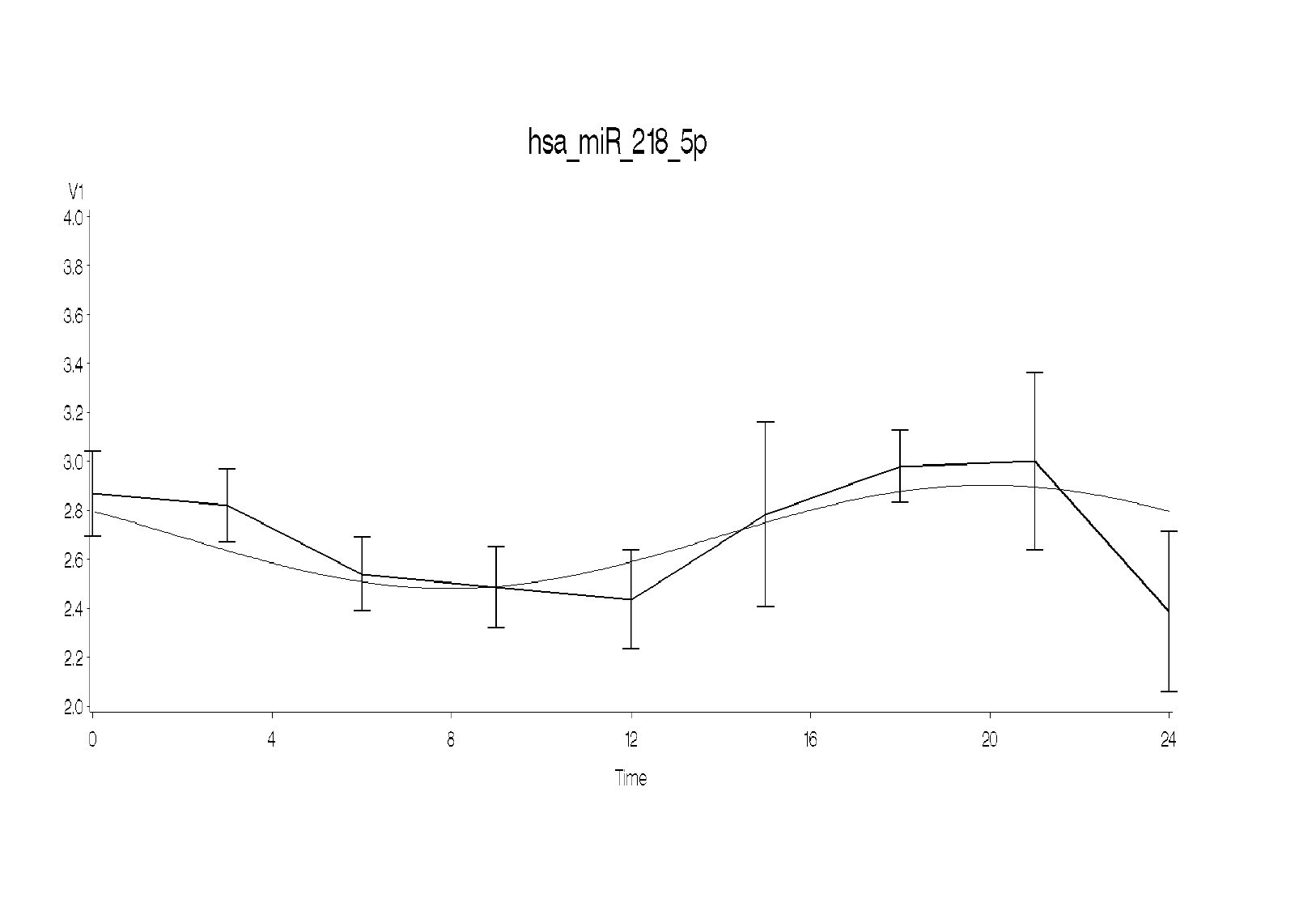

## Slide 55
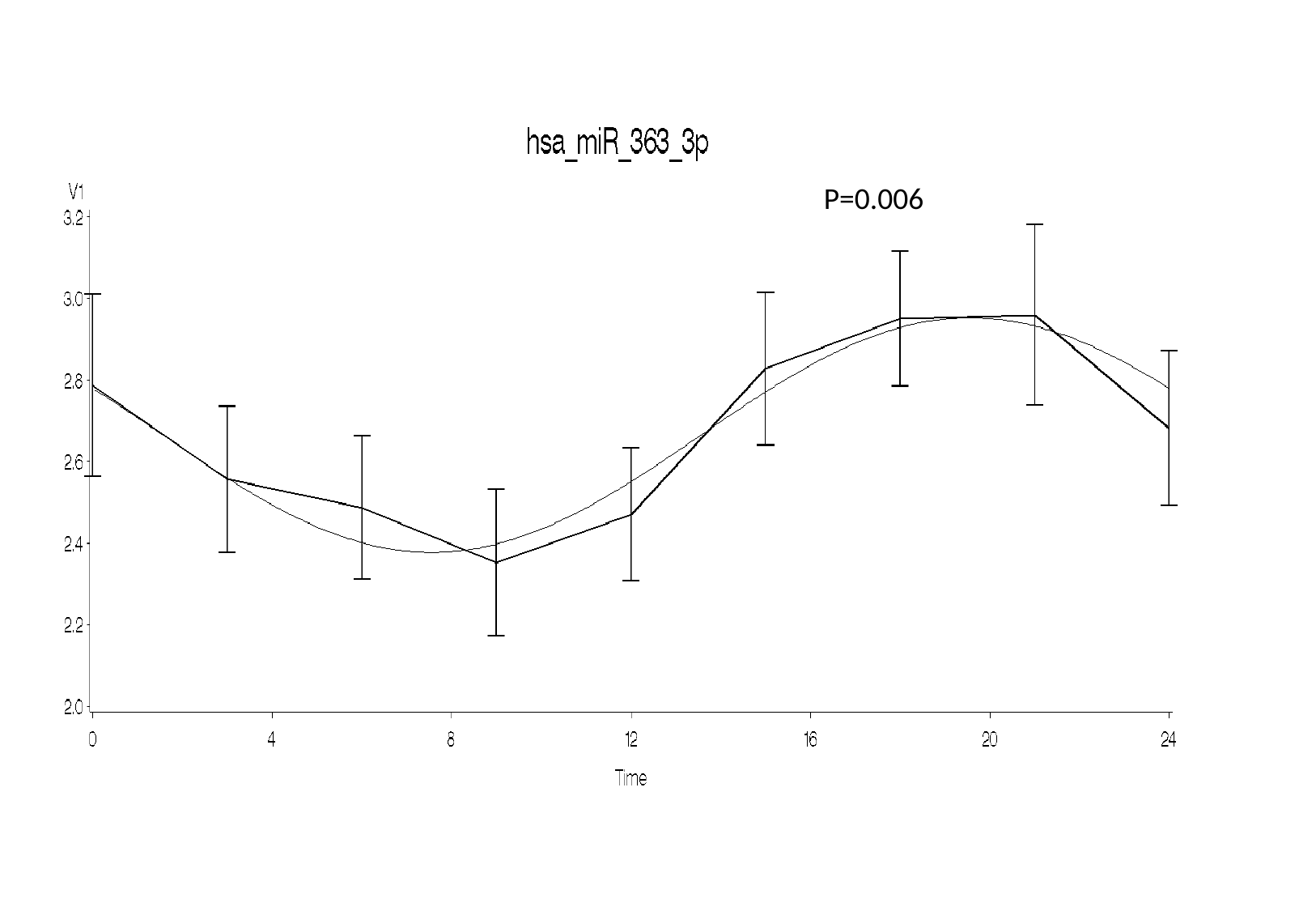

P=0.006

## Slide 56
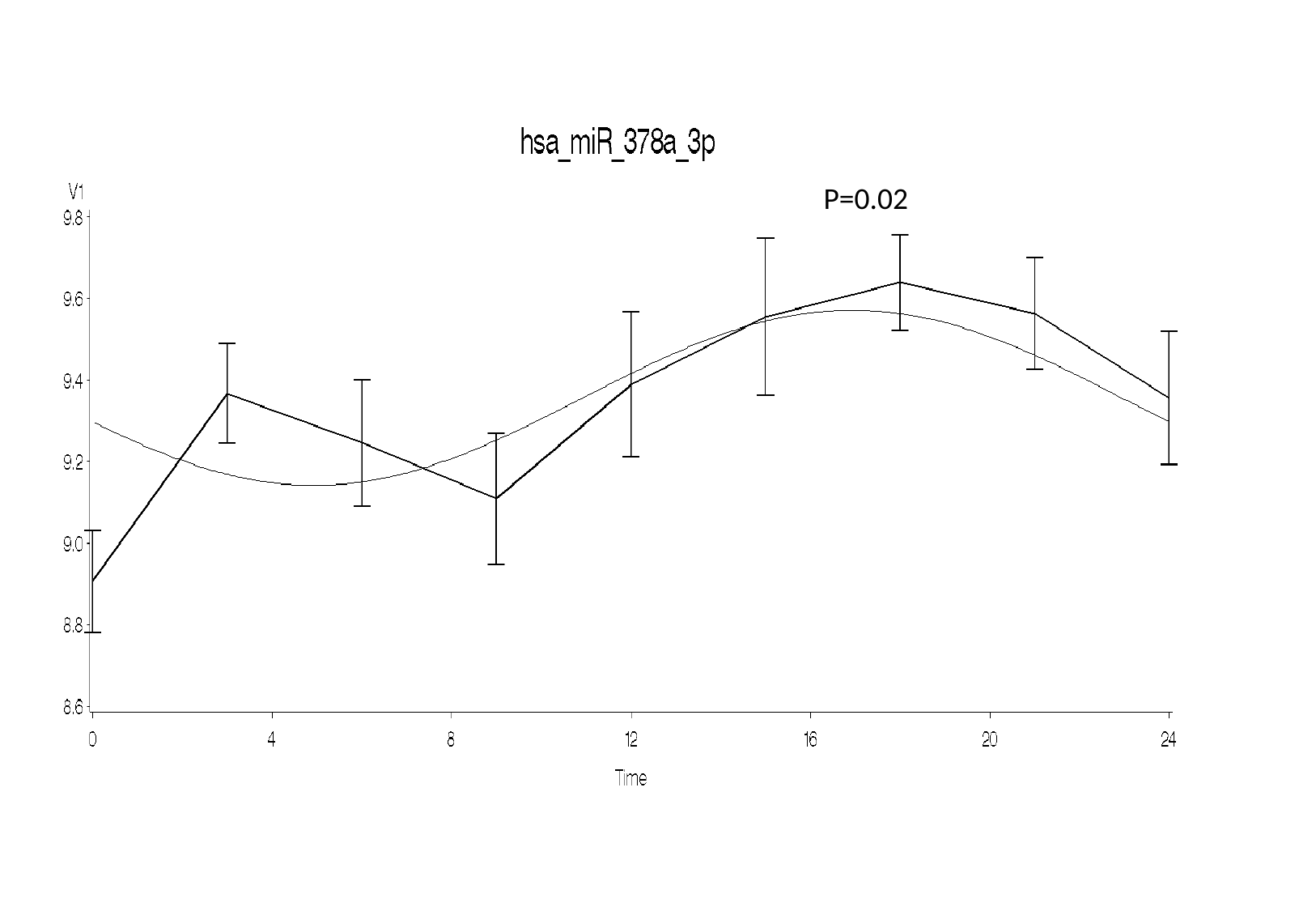

P=0.02

## Slide 57
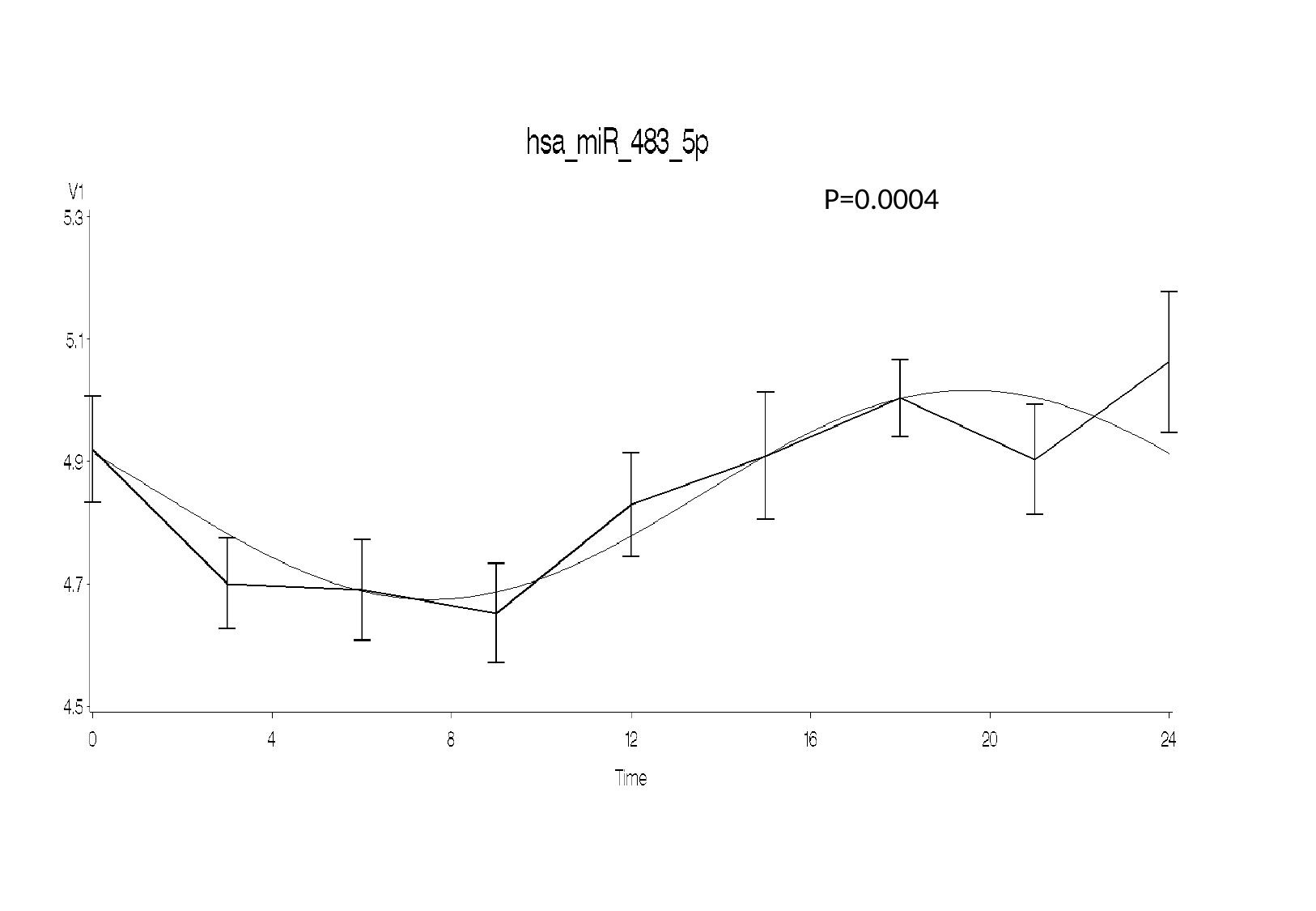

P=0.0004

## Slide 58
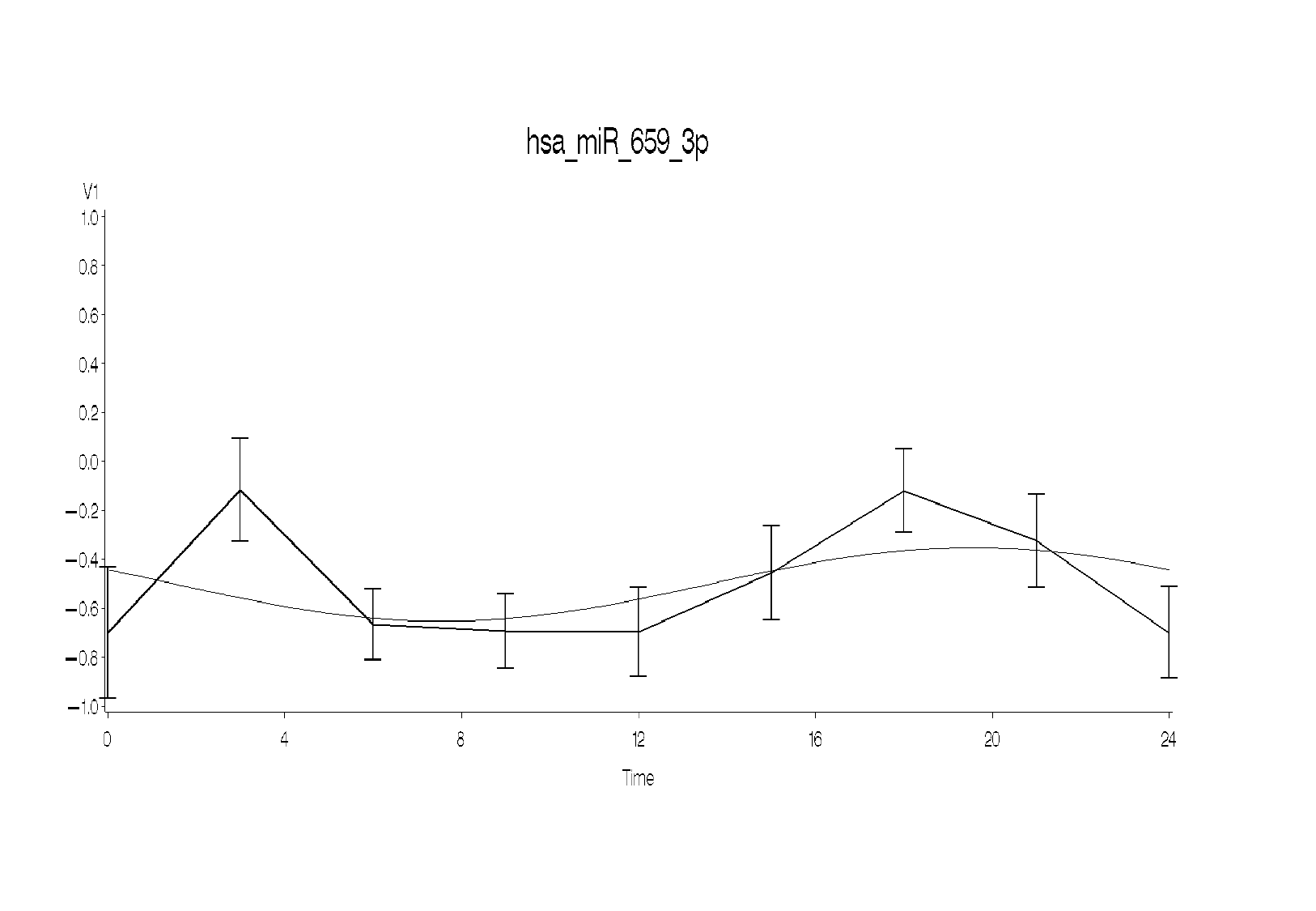

## Slide 59
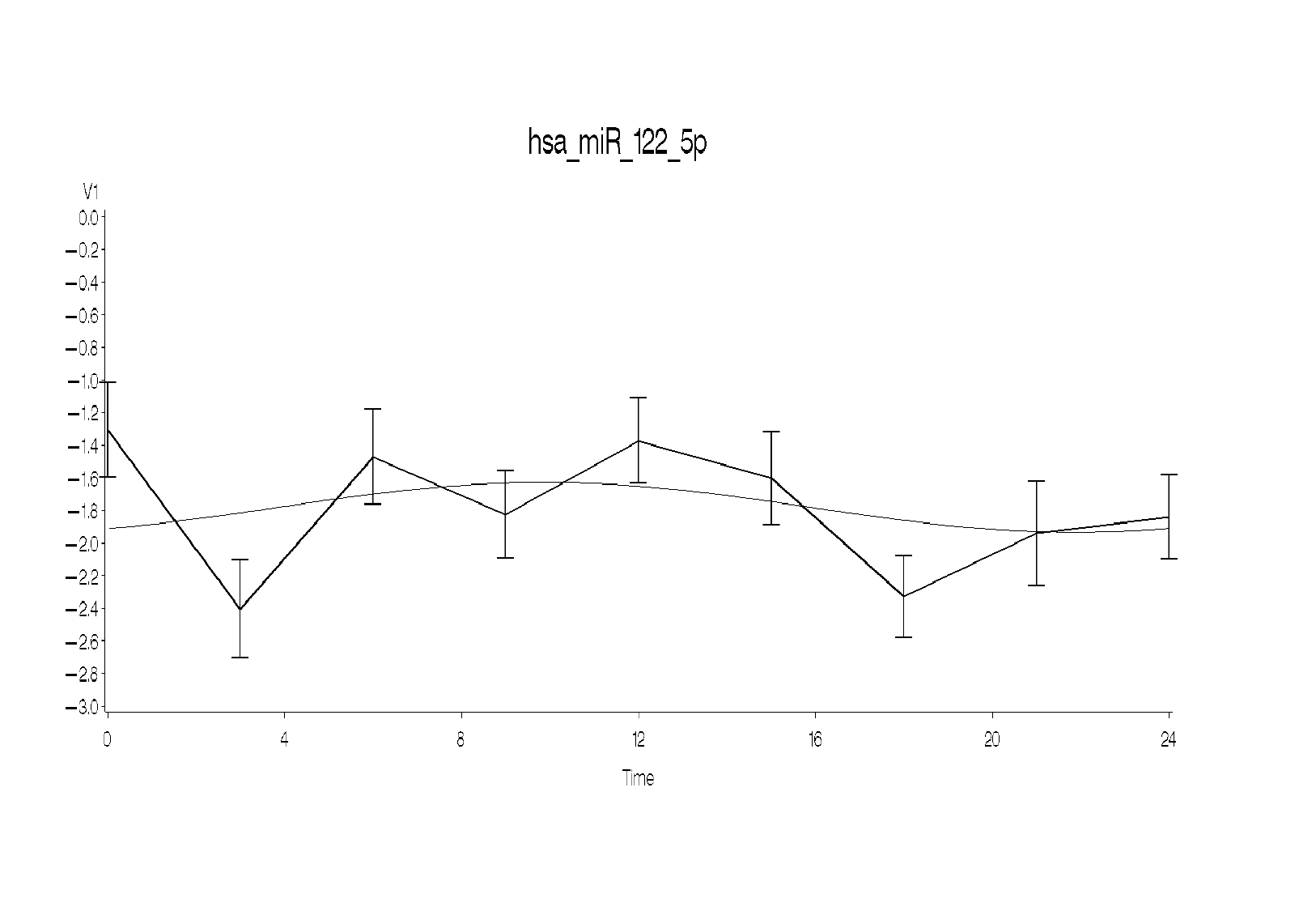

## Slide 60
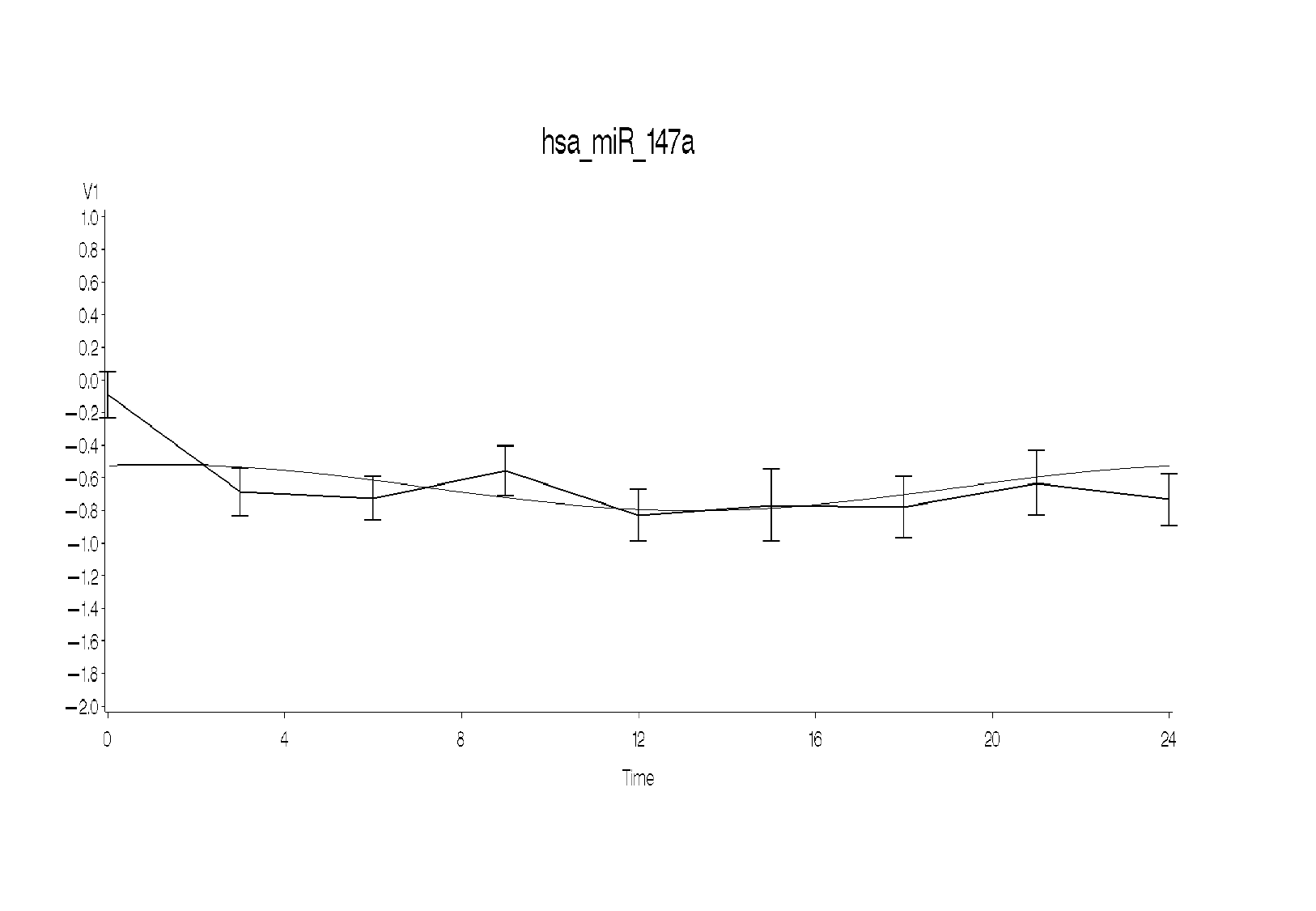

## Slide 61
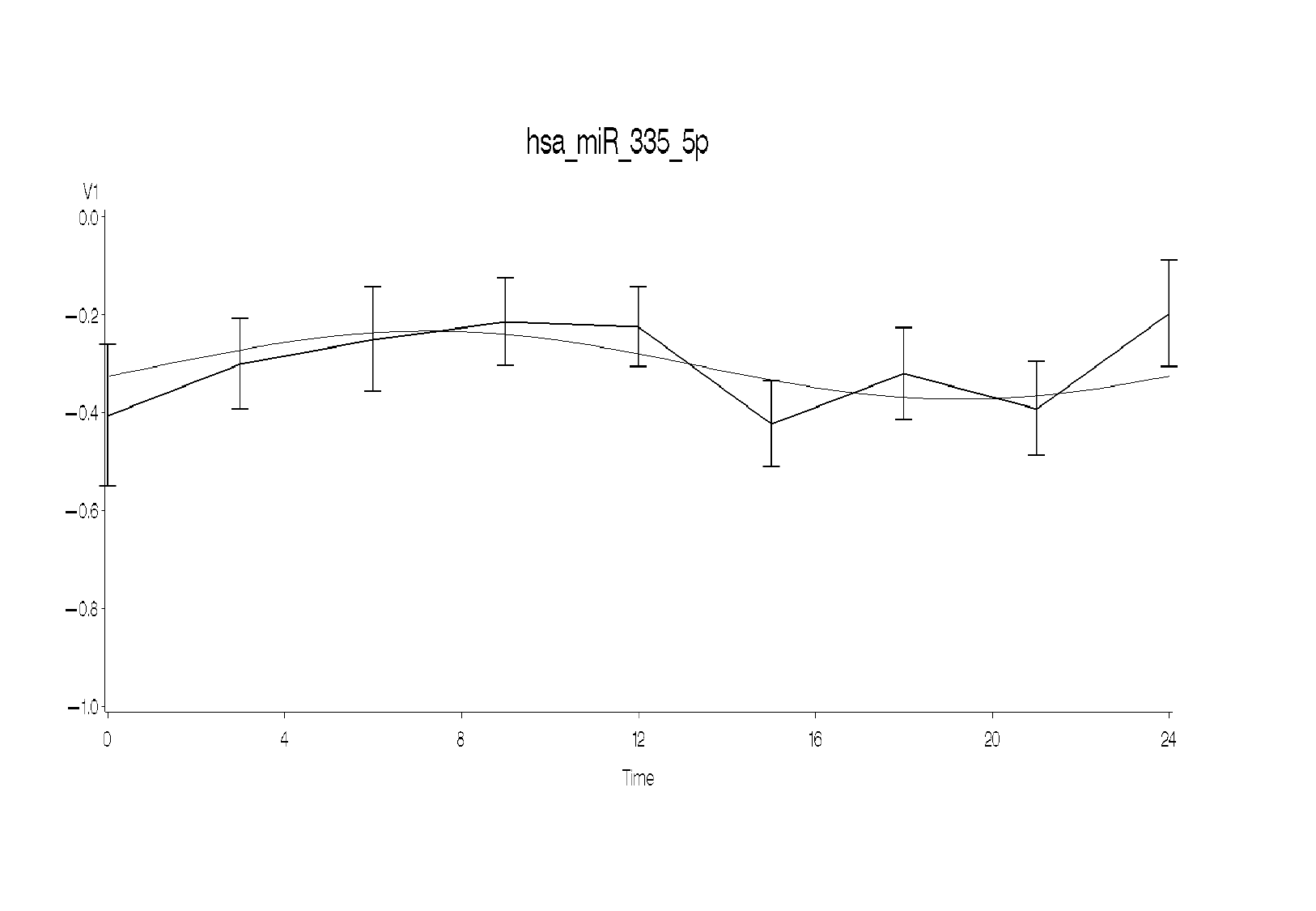

## Slide 62
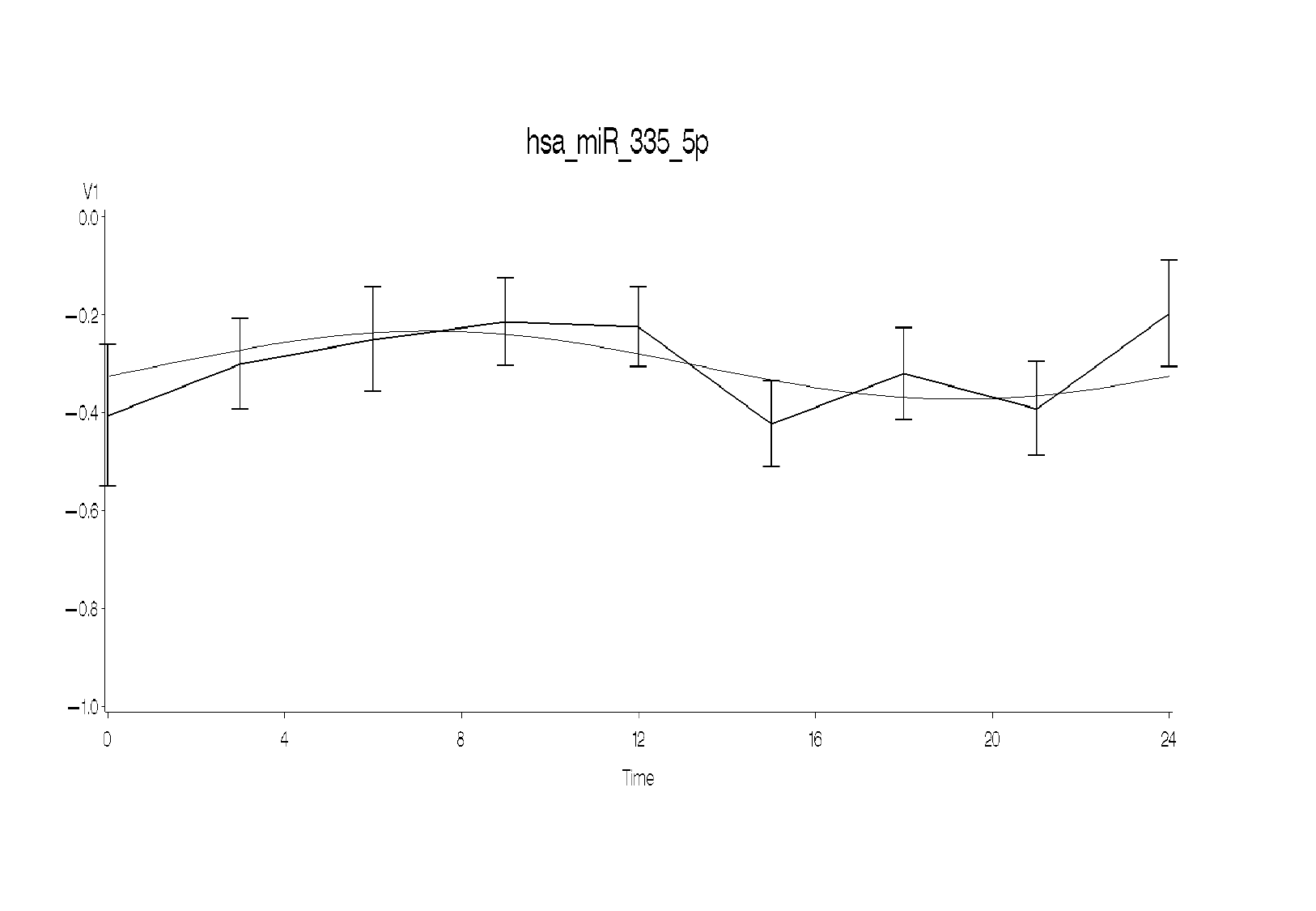

## Slide 63
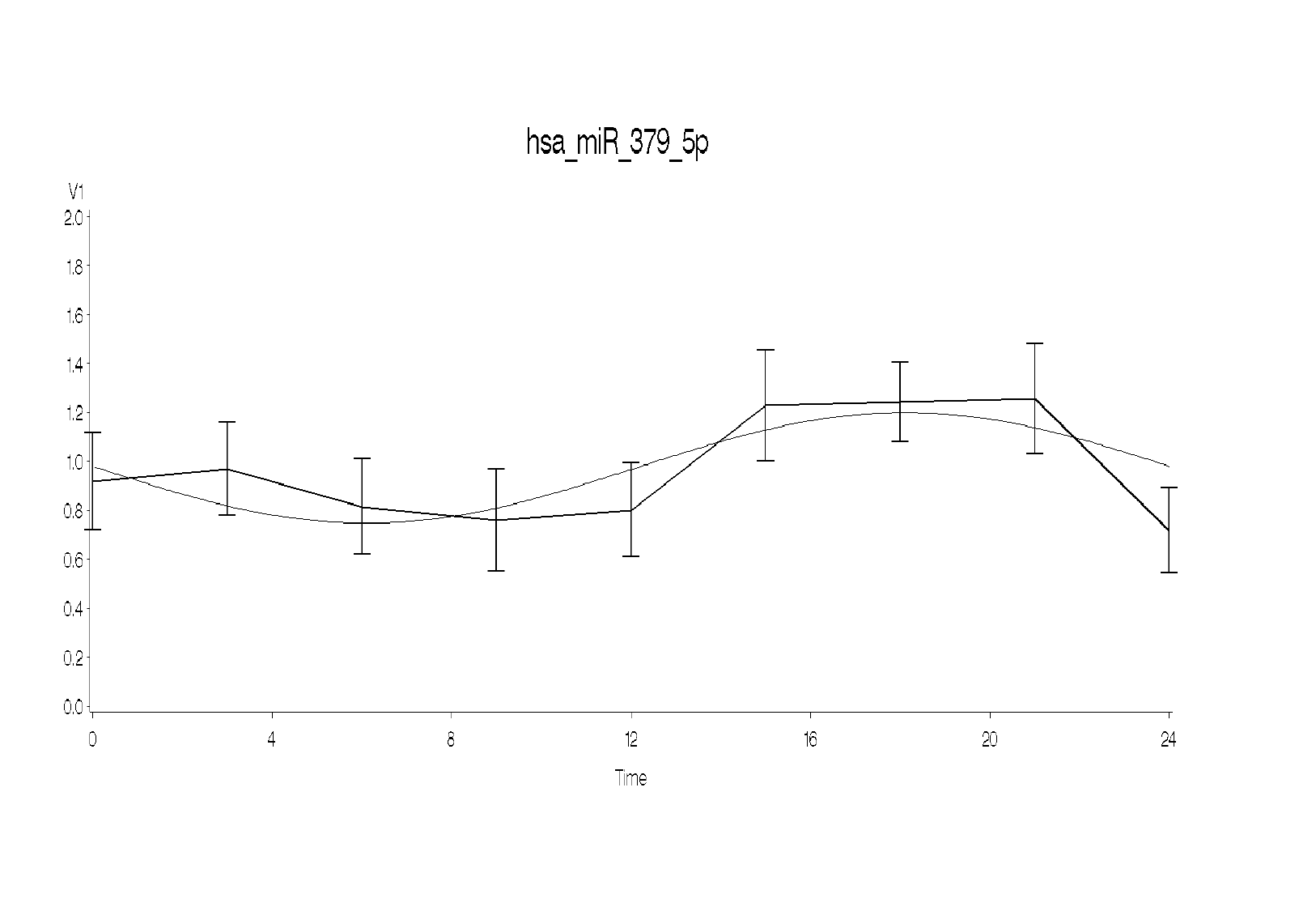

## Slide 64
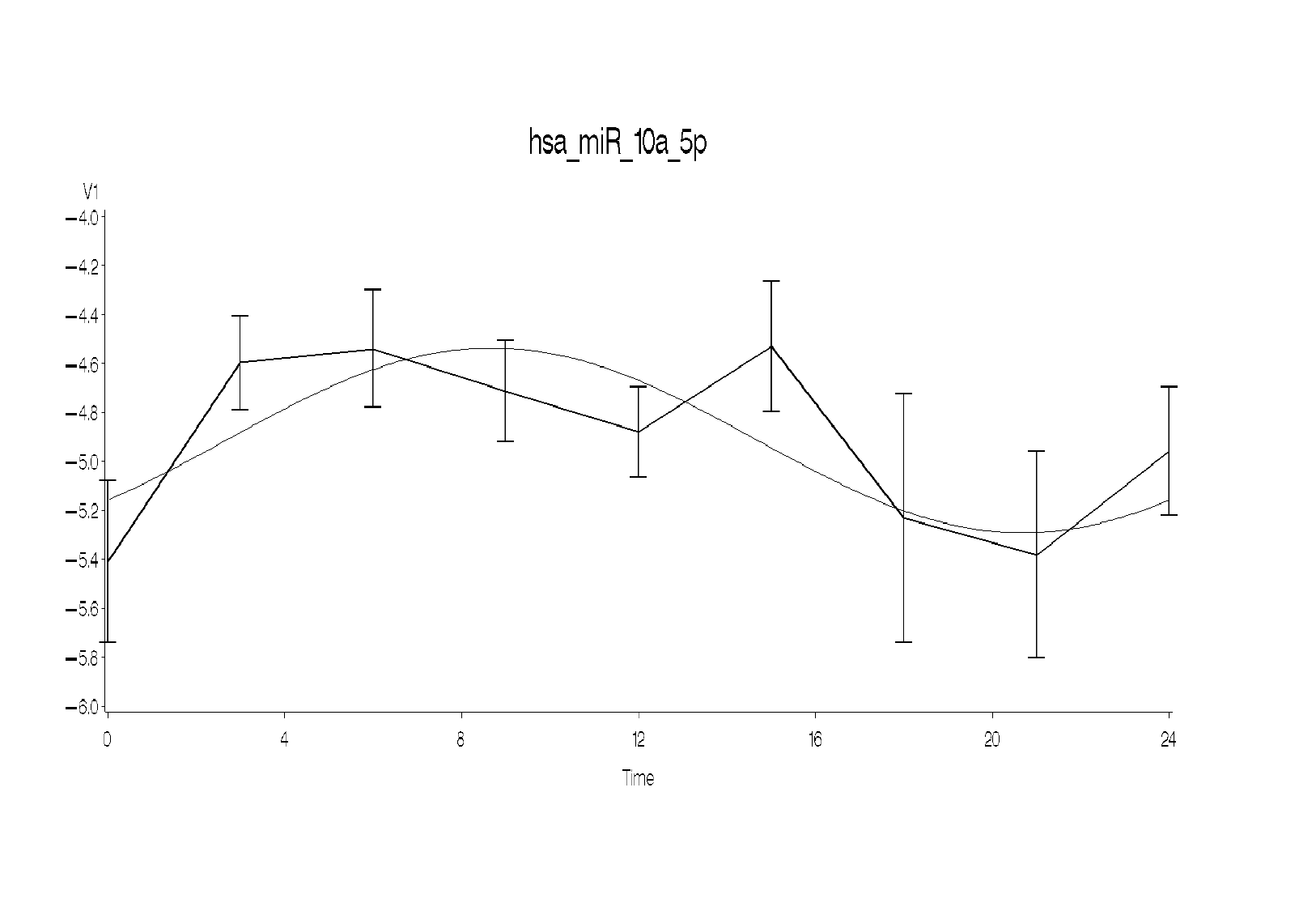

## Slide 65
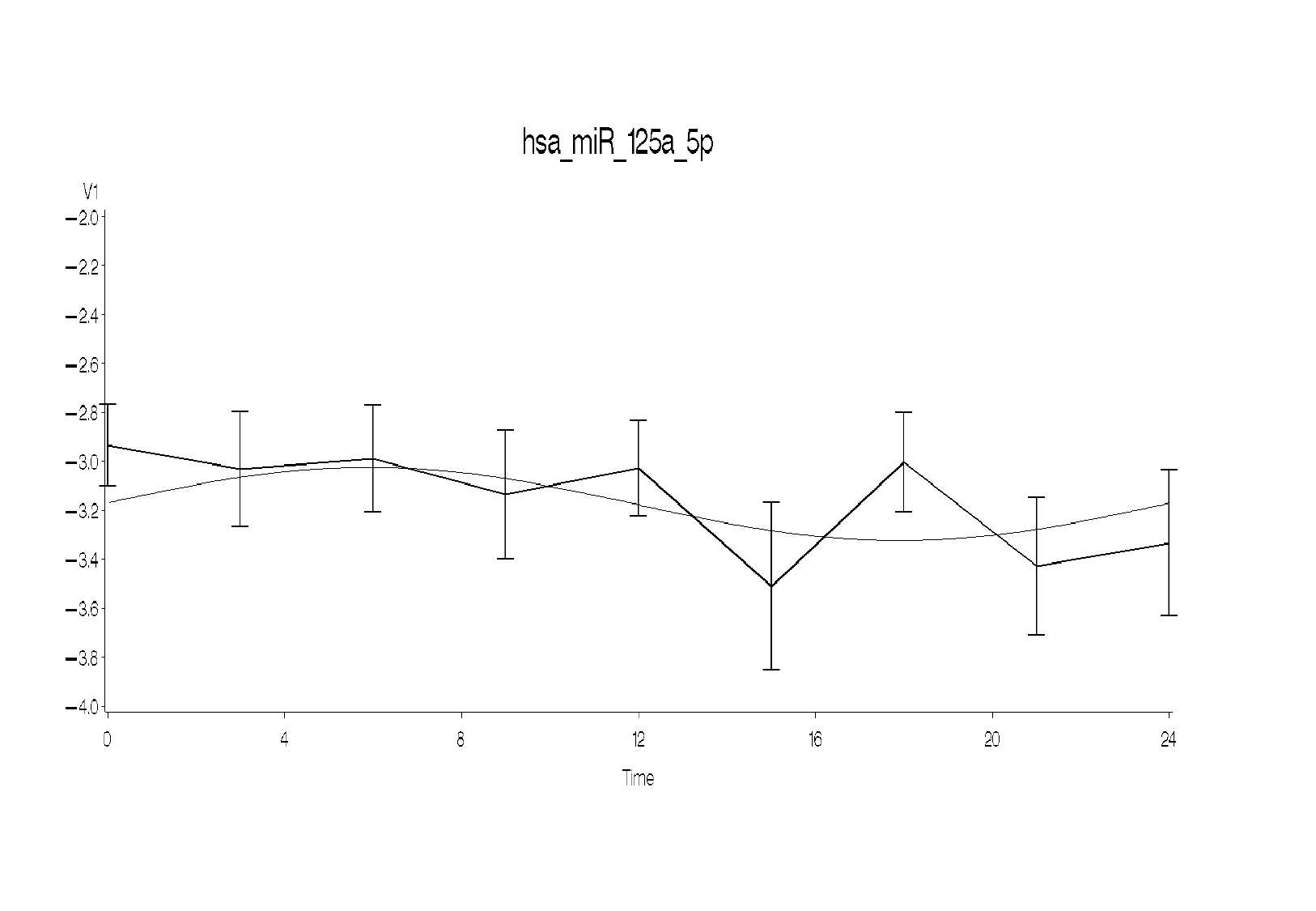

## Slide 66
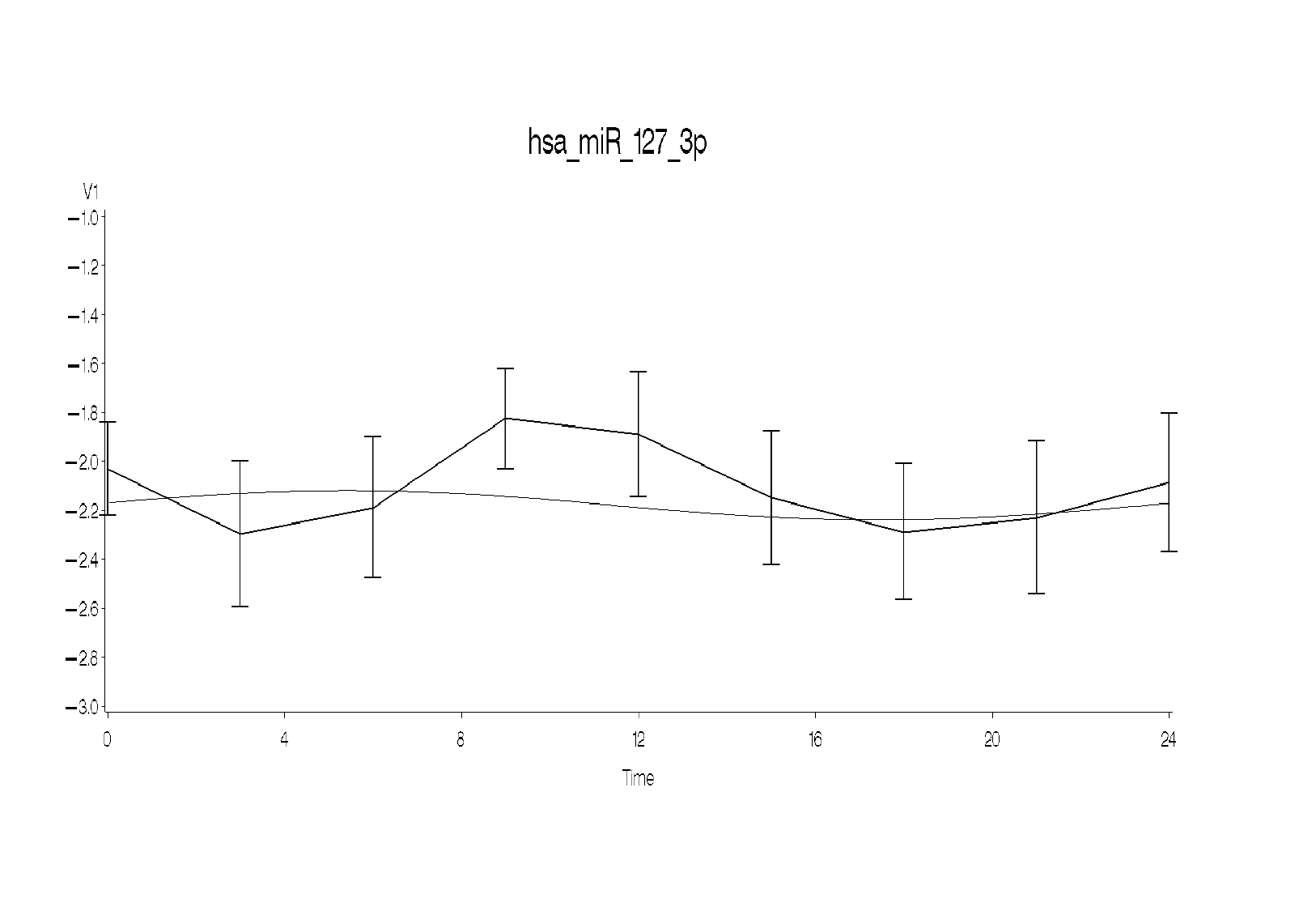

## Slide 67
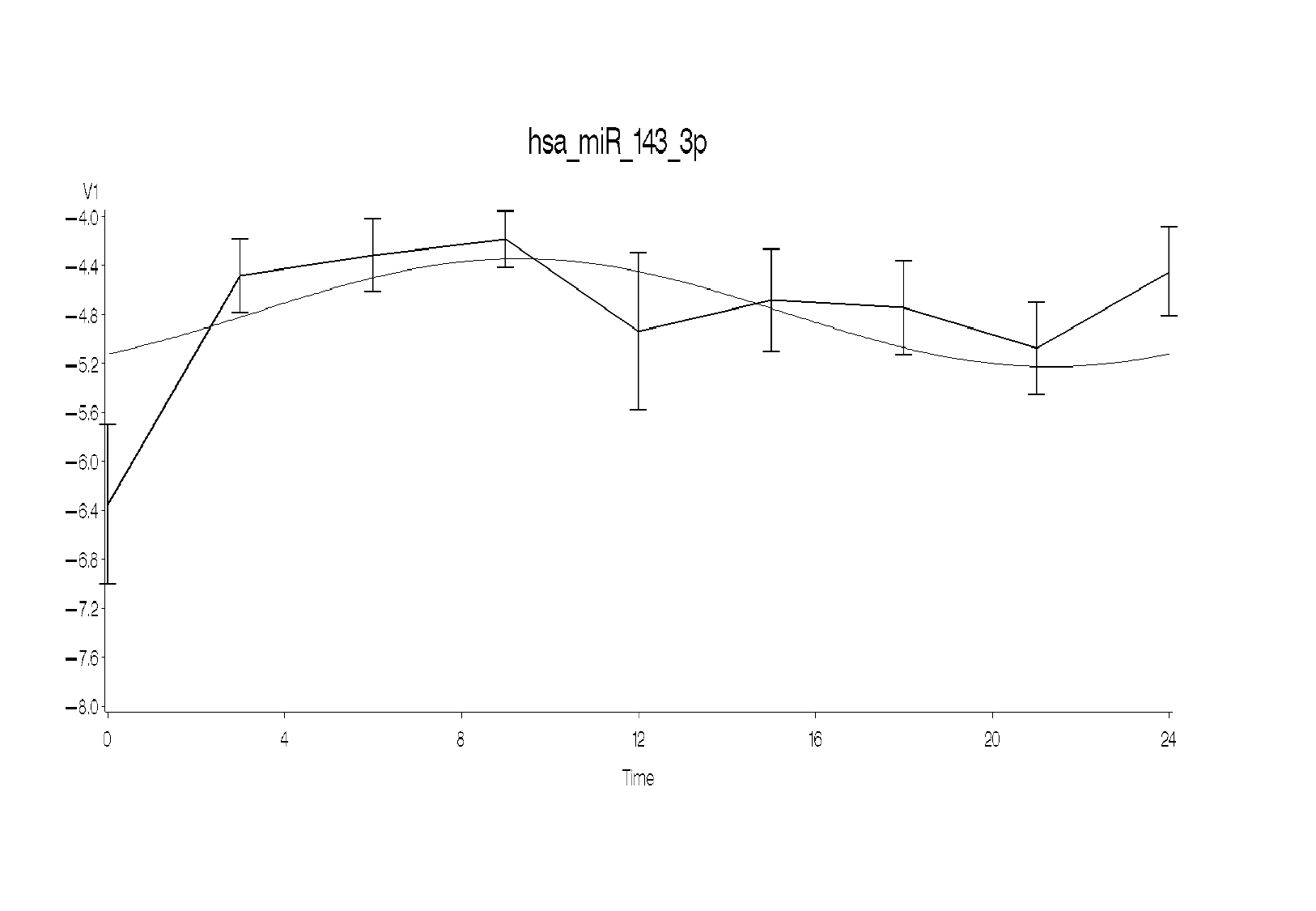

## Slide 68
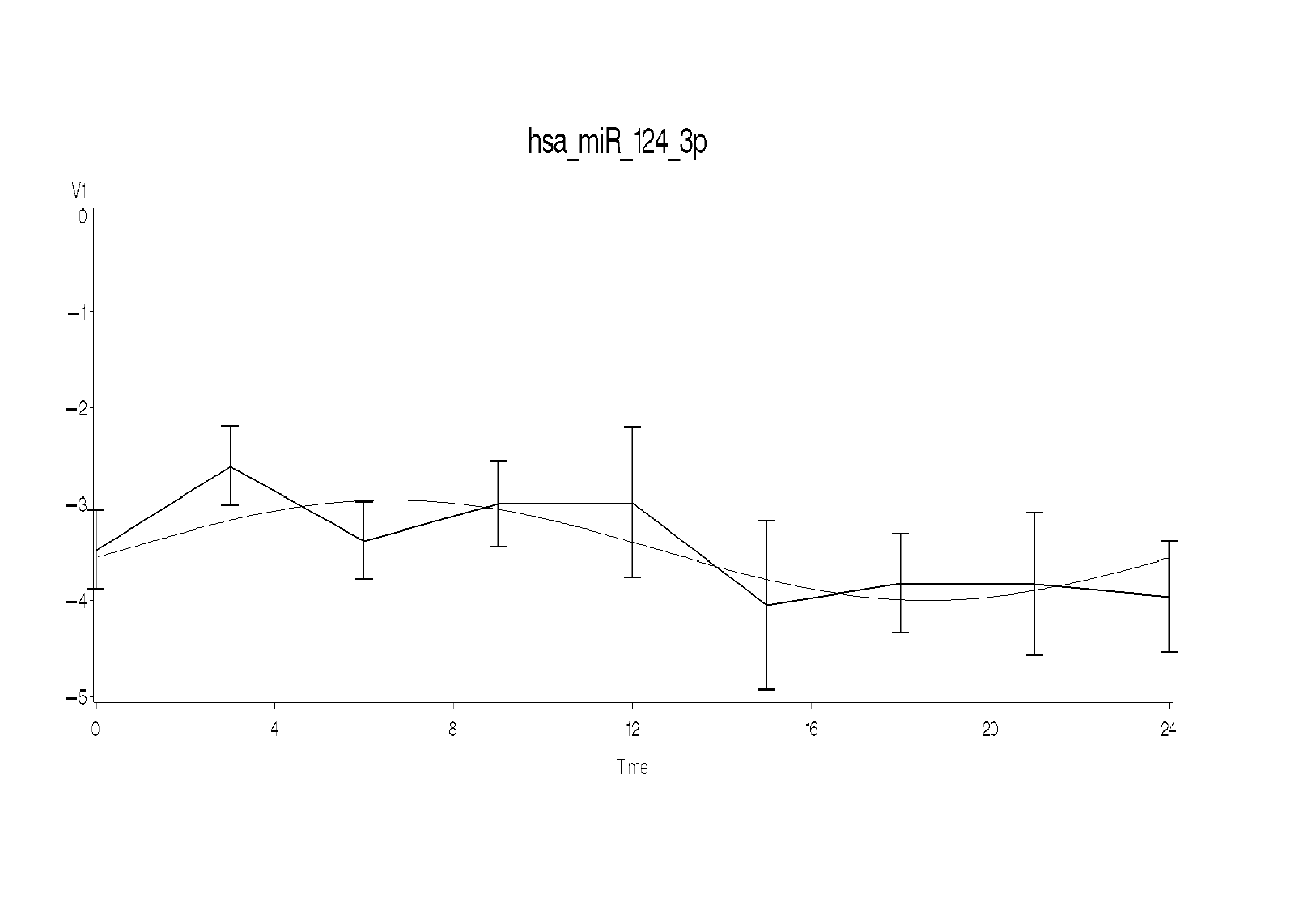

## Slide 69
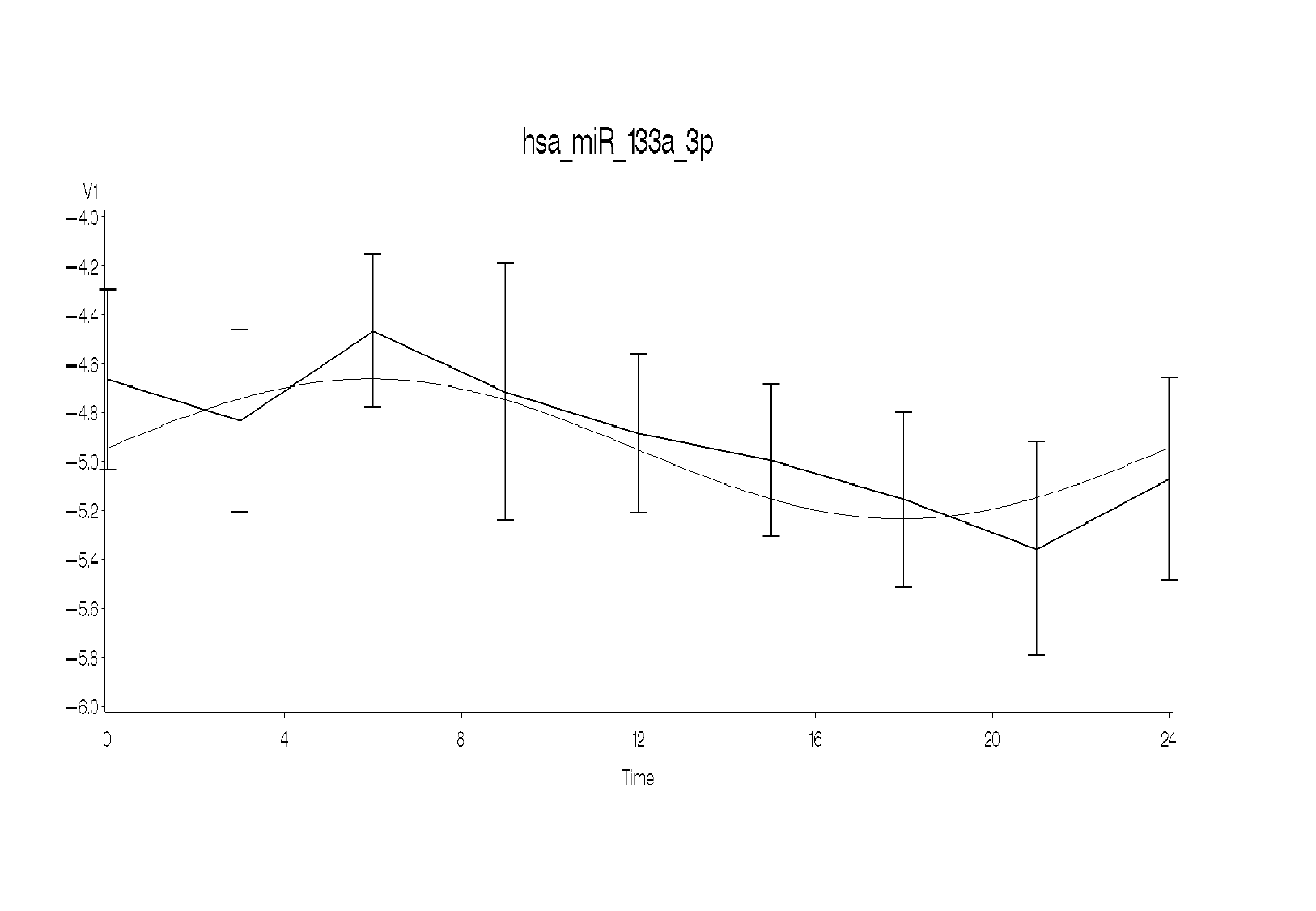

## Slide 70
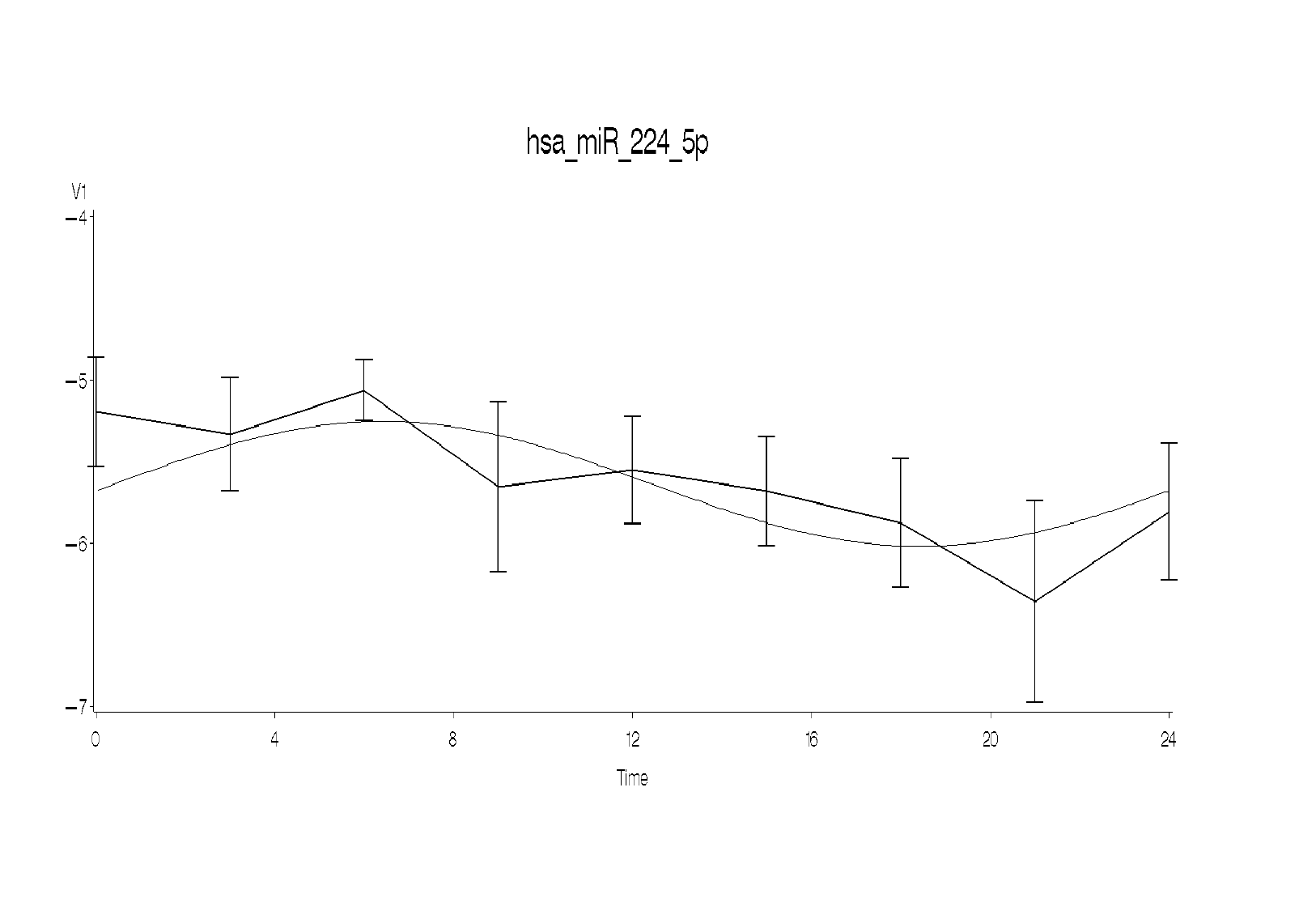

## Slide 71
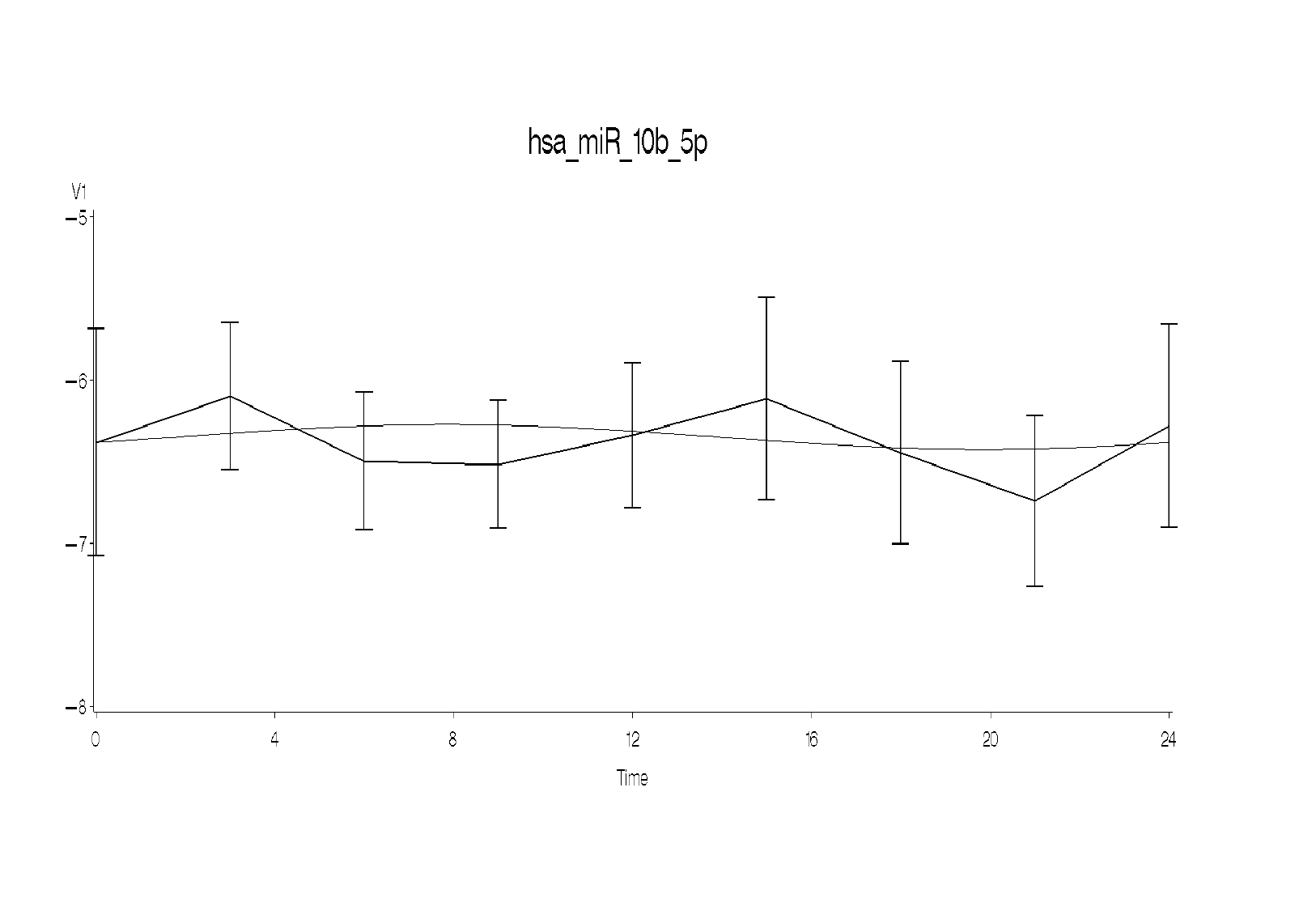

## Slide 72
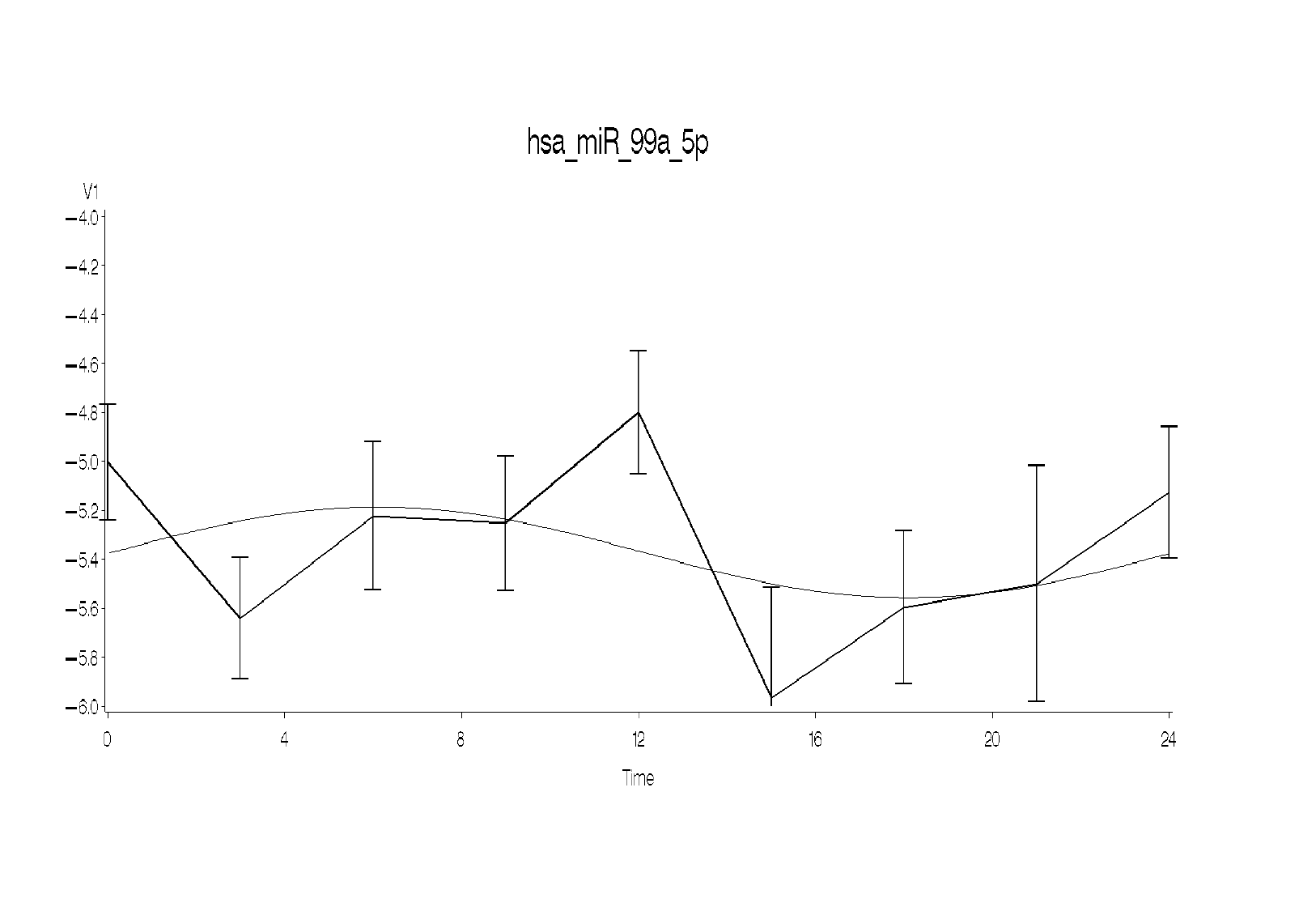

## Slide 73
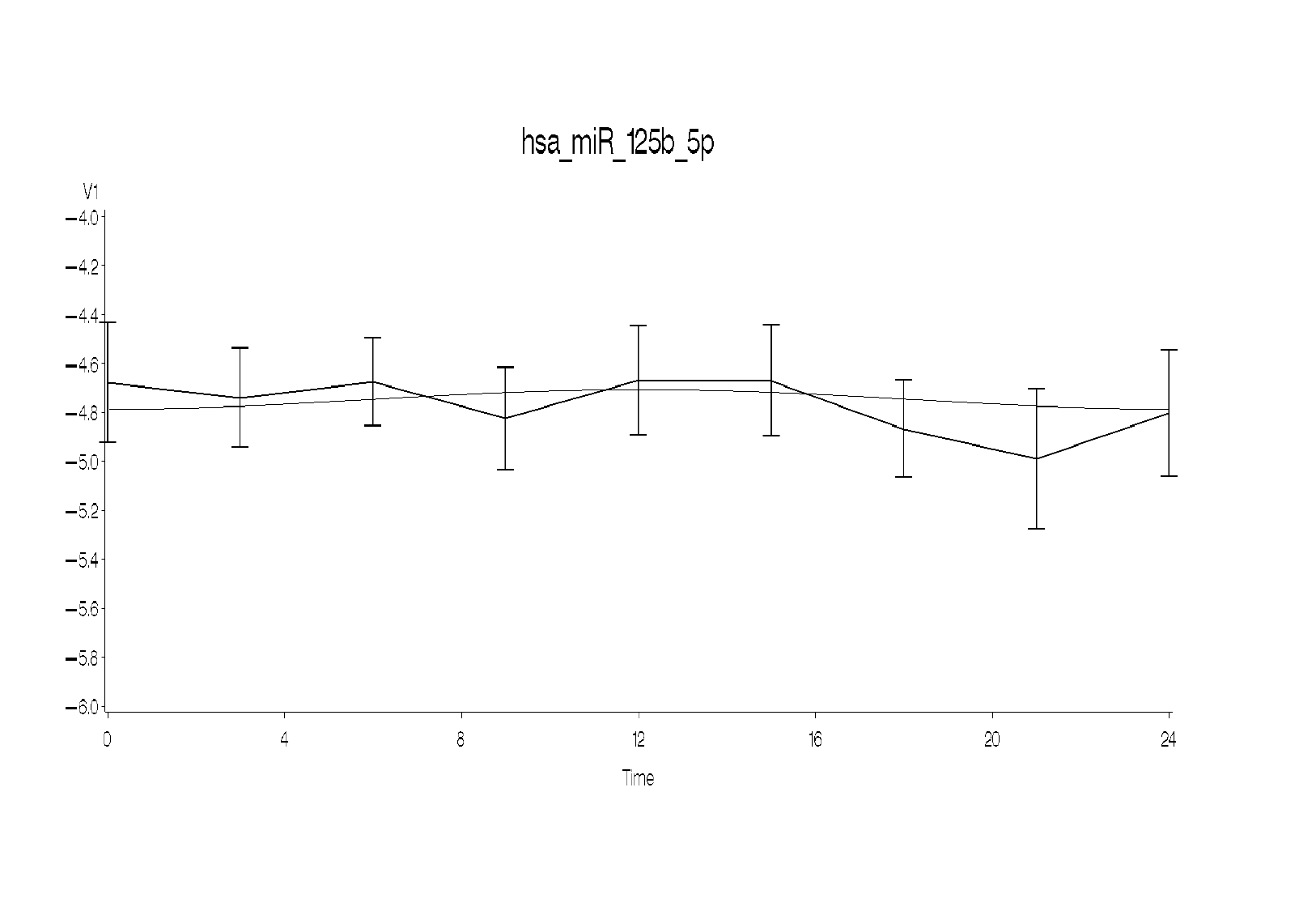

## Slide 74
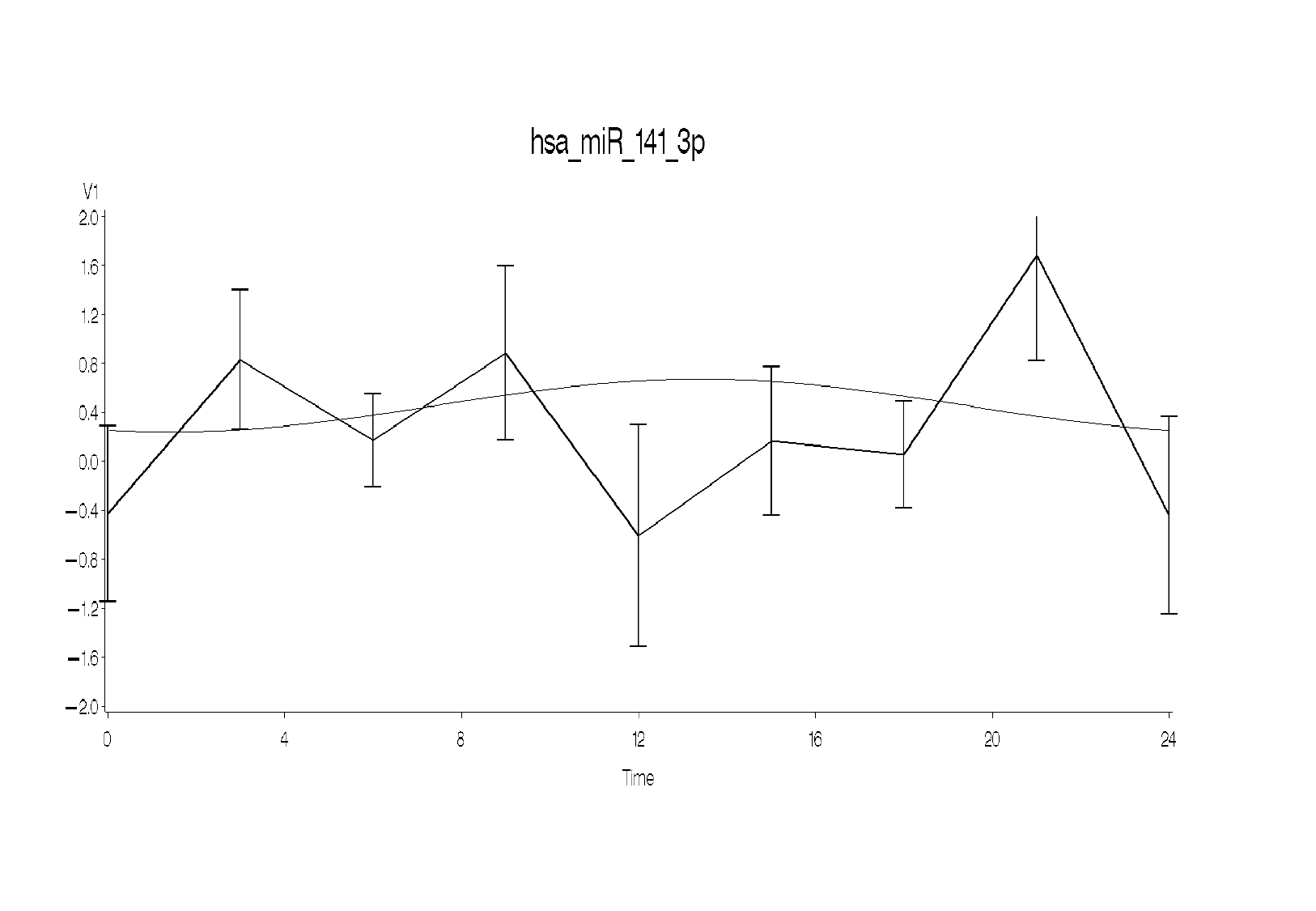

## Slide 75
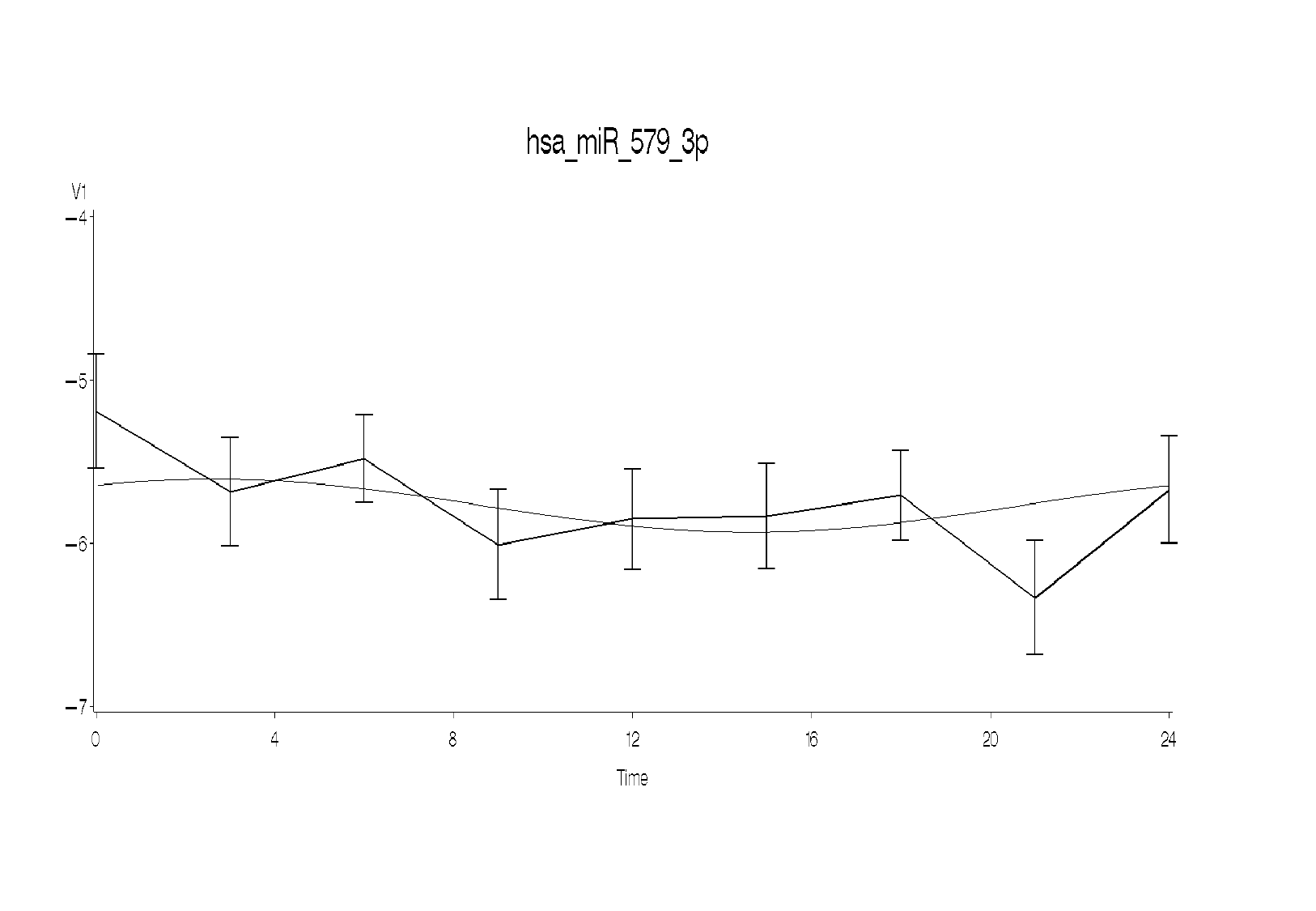

## Slide 76
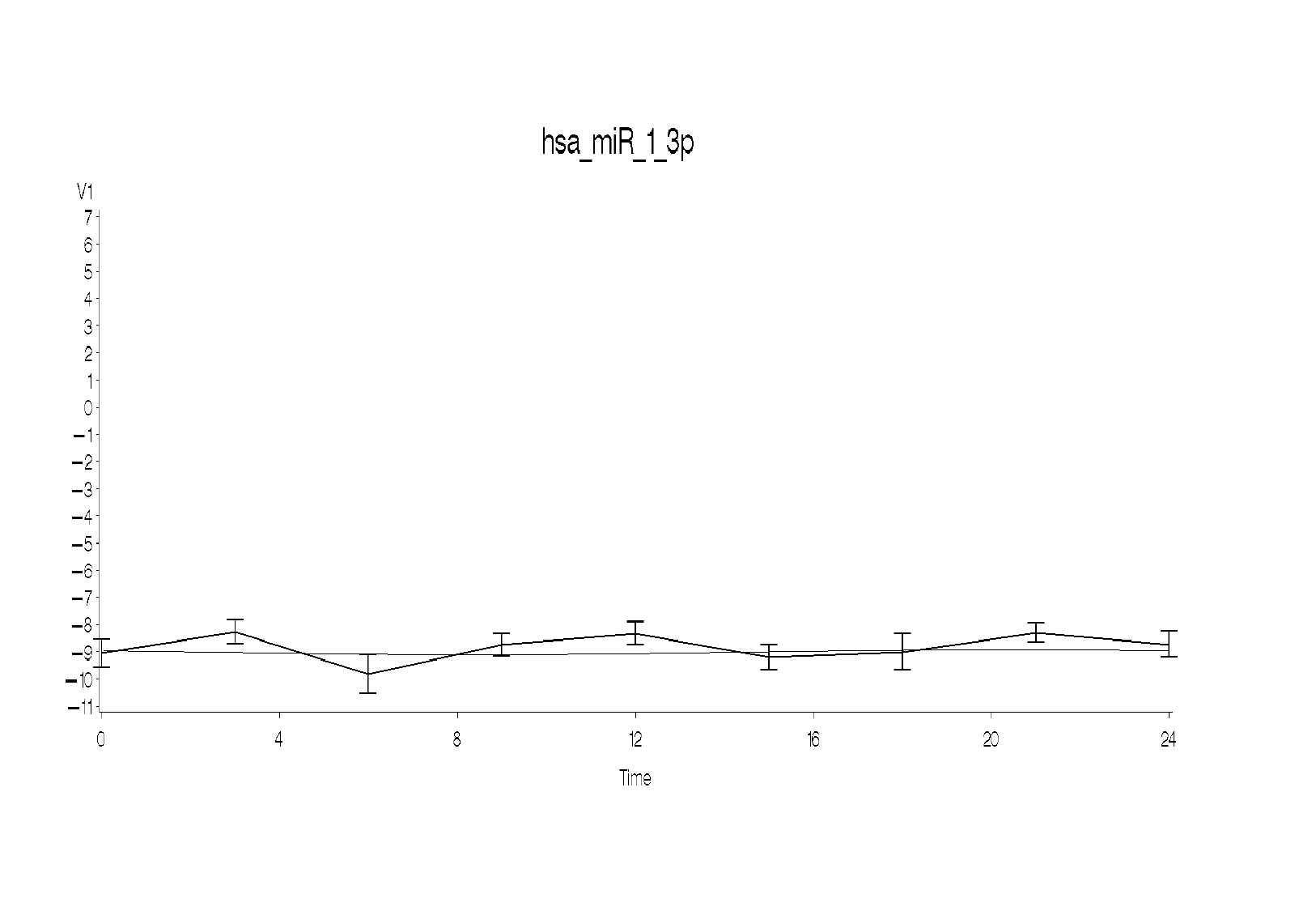

## Slide 77
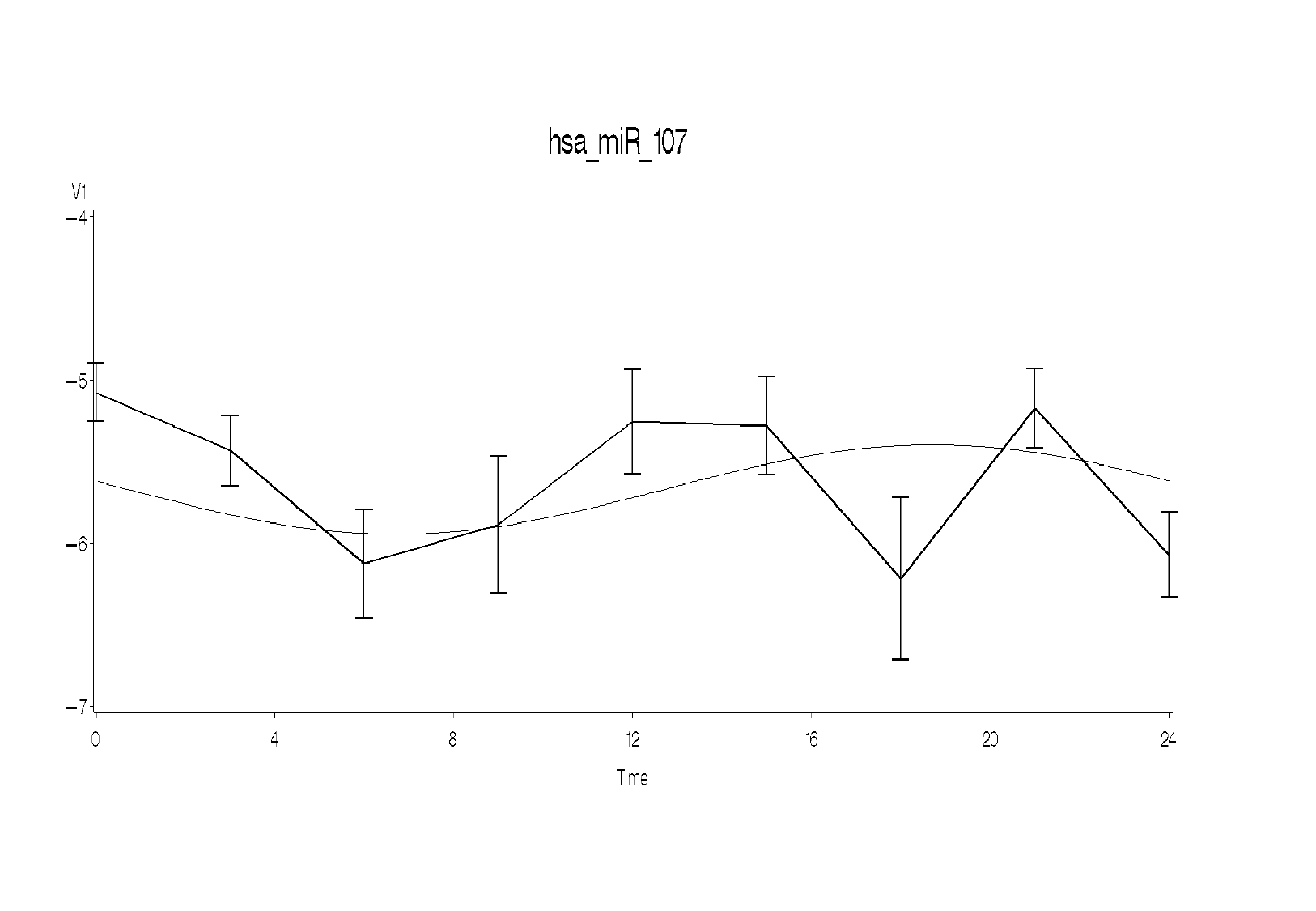

## Slide 78
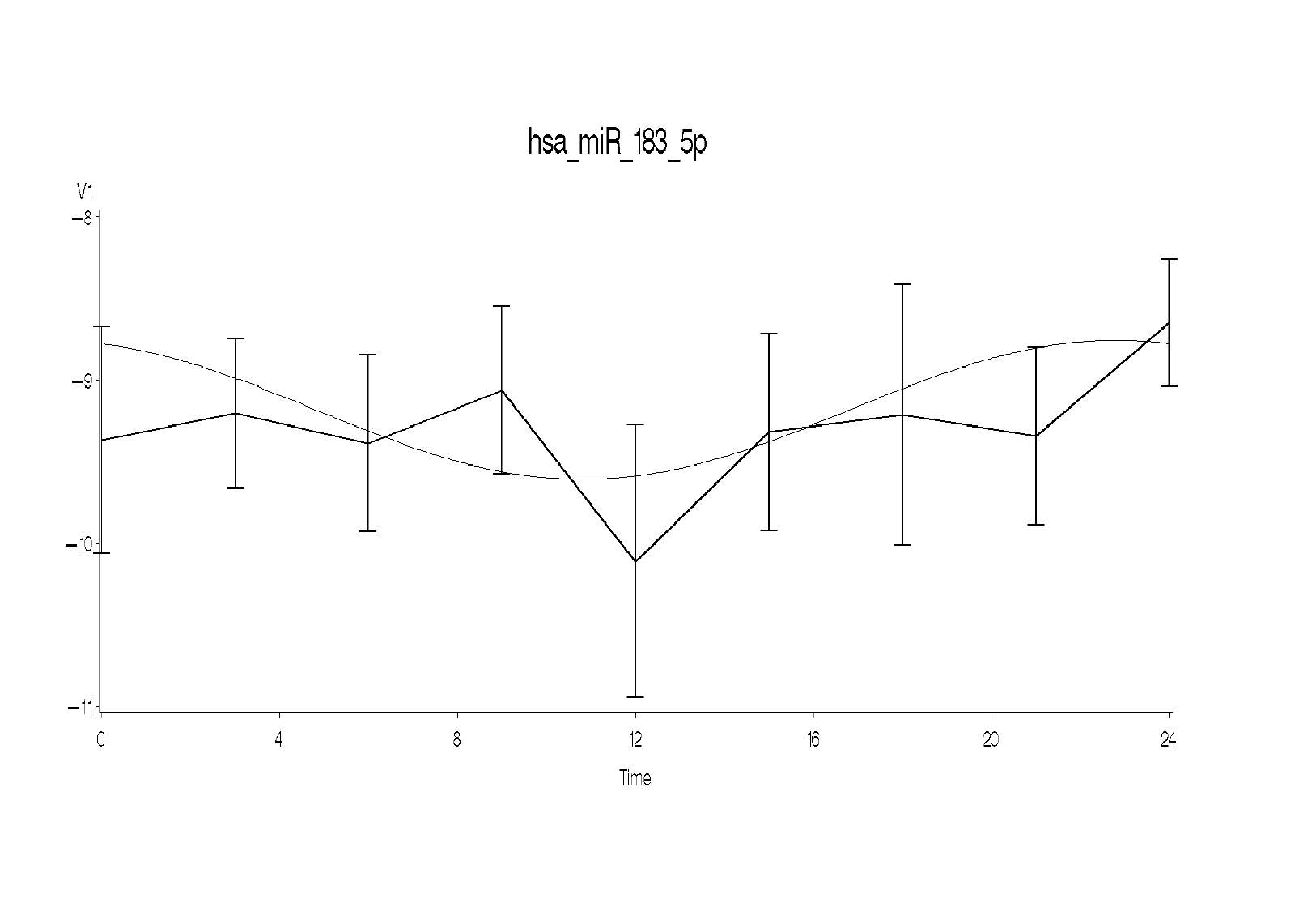

## Slide 79
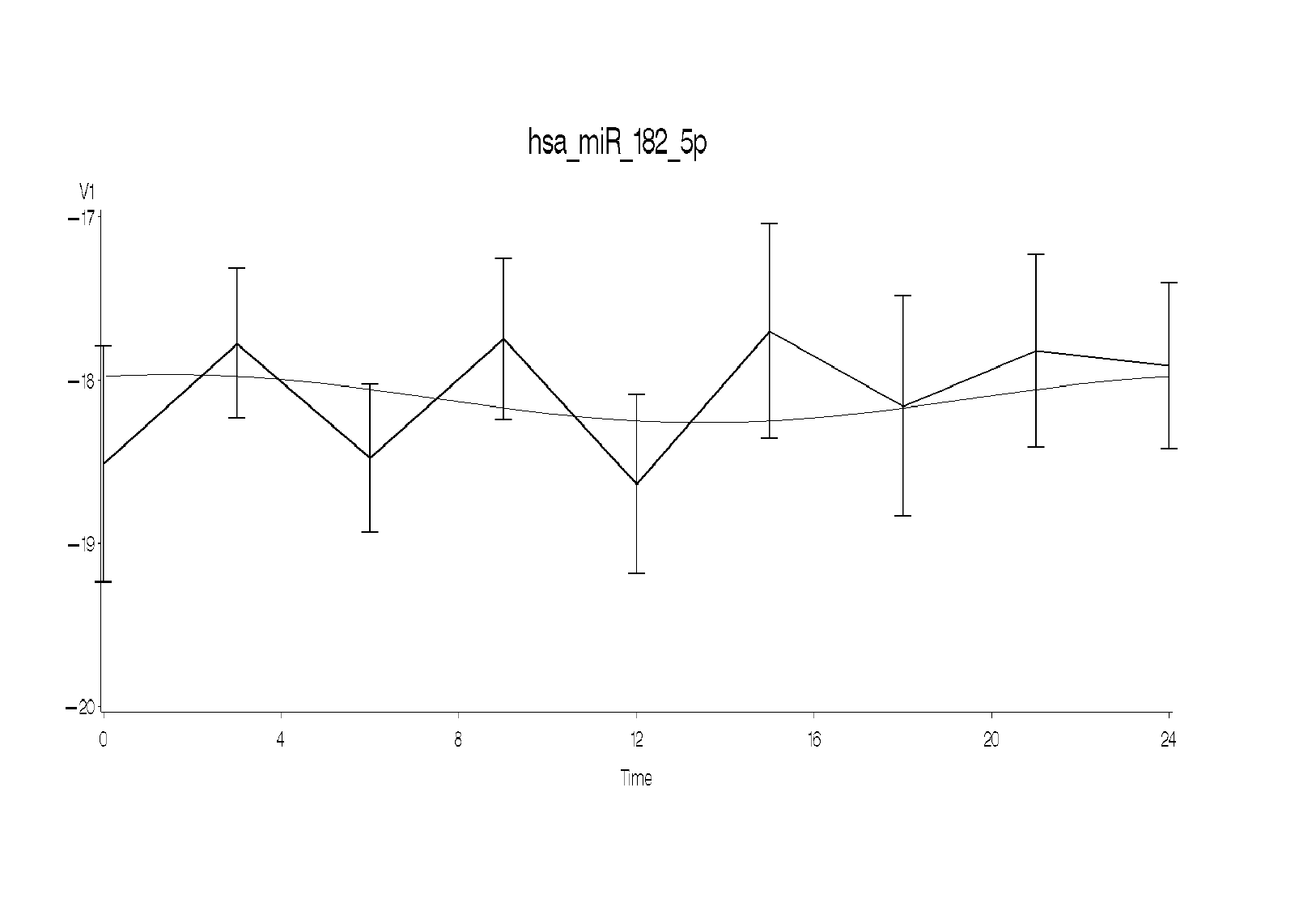

Supplement: S1 Fig — Individual points represent the mean and SEM of the values of the 24 individuals included in the study. In case of significant rhythmicity, the p-values are included on the graphs. (PPTX) [file pone.0160577.s001.pptx]
