# Supplementary material for: Diurnal Variations of Human Circulating Cell-Free Micro-RNA
Source: PLoS One. 2016 Aug 5;11(8):e0160577. doi: 10.1371/journal.pone.0160577 (PMC4975411; doi:10.1371/journal.pone.0160577)
Supplement: S2 Fig — Individual data points, mean and SEM are shown for row mean-normalized, technically normalized, and raw (not normalized) miR-16 data. (PPTX) [file pone.0160577.s002.pptx]

## Slide 1
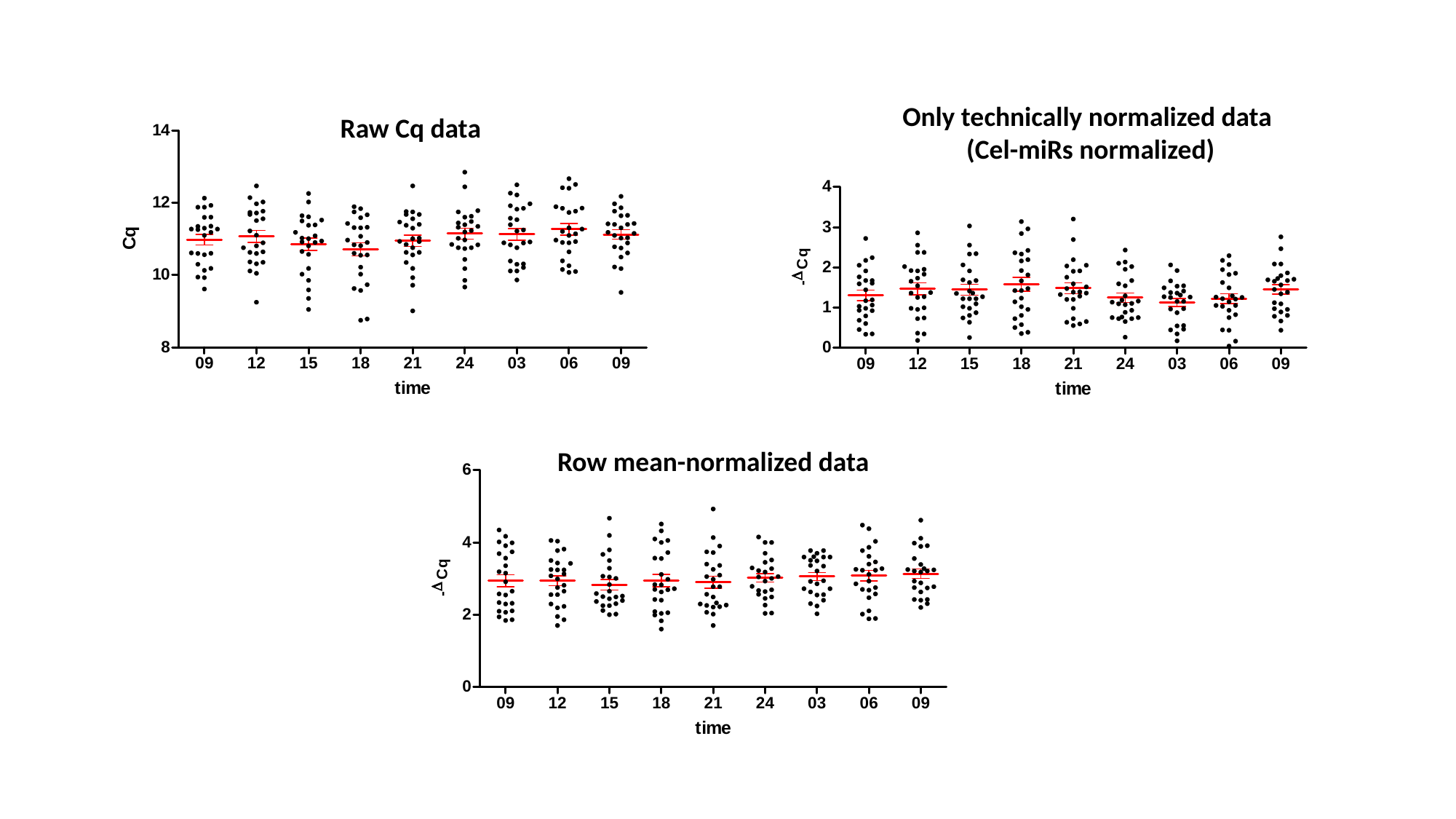

Only technically normalized data
(Cel-miRs normalized)
Raw Cq data
Row mean-normalized data
