# Supplementary material for: Diurnal Variations of Human Circulating Cell-Free Micro-RNA
Source: PLoS One. 2016 Aug 5;11(8):e0160577. doi: 10.1371/journal.pone.0160577 (PMC4975411; doi:10.1371/journal.pone.0160577)
Supplement: S3 Fig — Comparison side-by-side of row mean-normalized with row miR-16 normalized data. Data points (mean and SEM) and curve fitting are shown. (PPTX) [file pone.0160577.s003.pptx]

## Slide 1
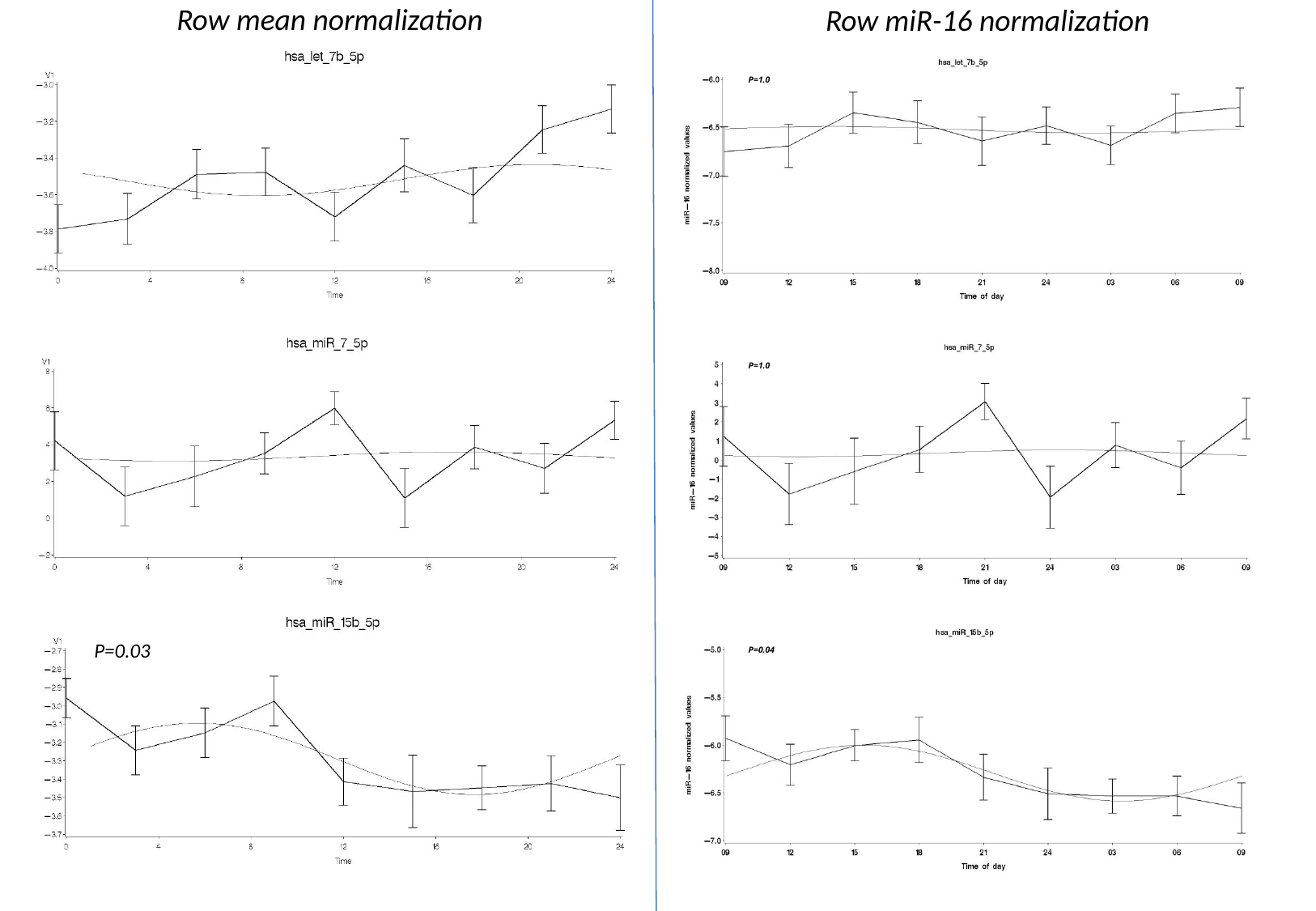

Row mean normalization
Row miR-16 normalization
P=1.0
P=1.0
P=0.03
P=0.04

## Slide 2
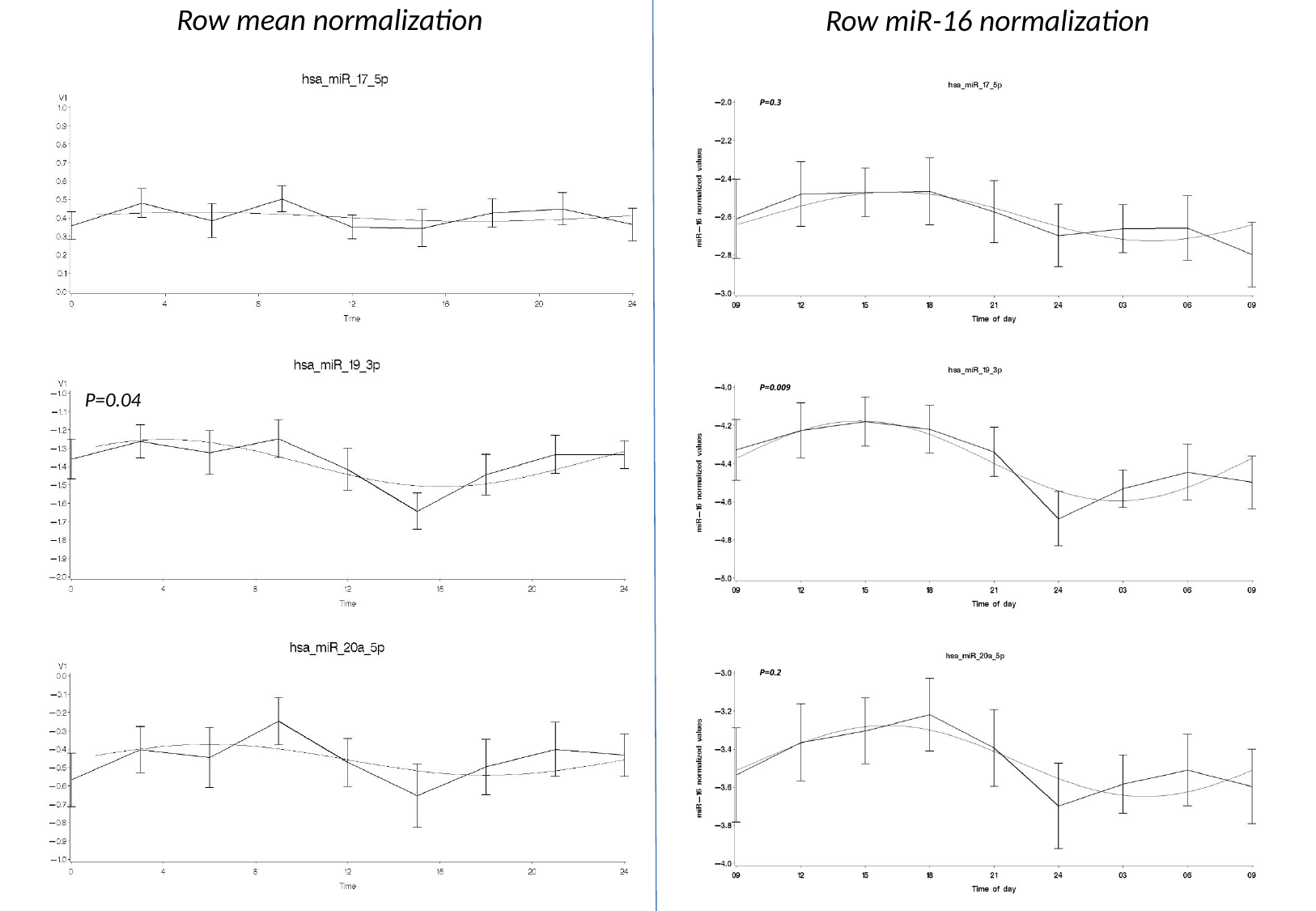

Row mean normalization
Row miR-16 normalization
P=0.3
P=0.009
P=0.04
P=0.2

## Slide 3
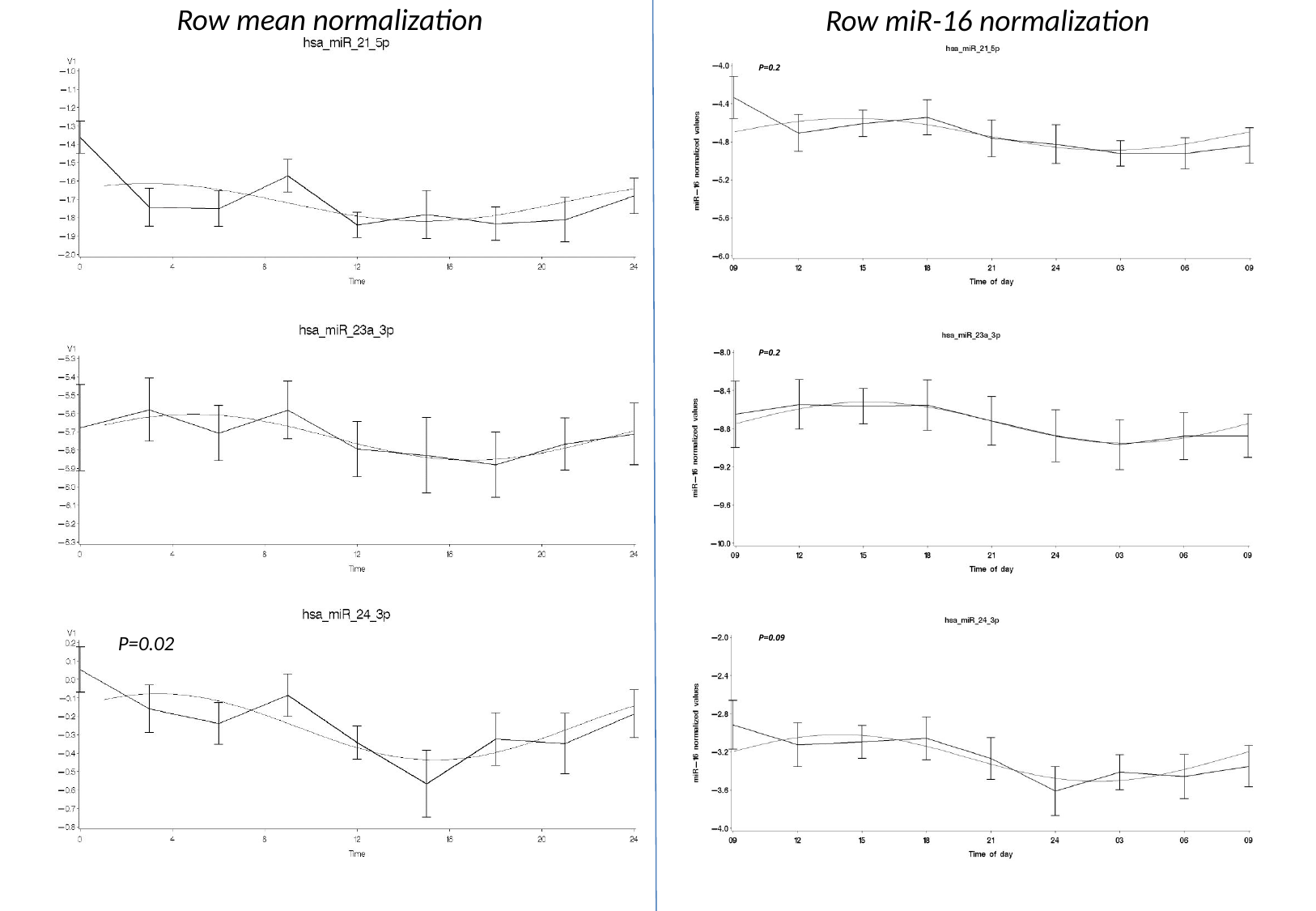

Row mean normalization
Row miR-16 normalization
P=0.2
P=0.2
P=0.02
P=0.09

## Slide 4
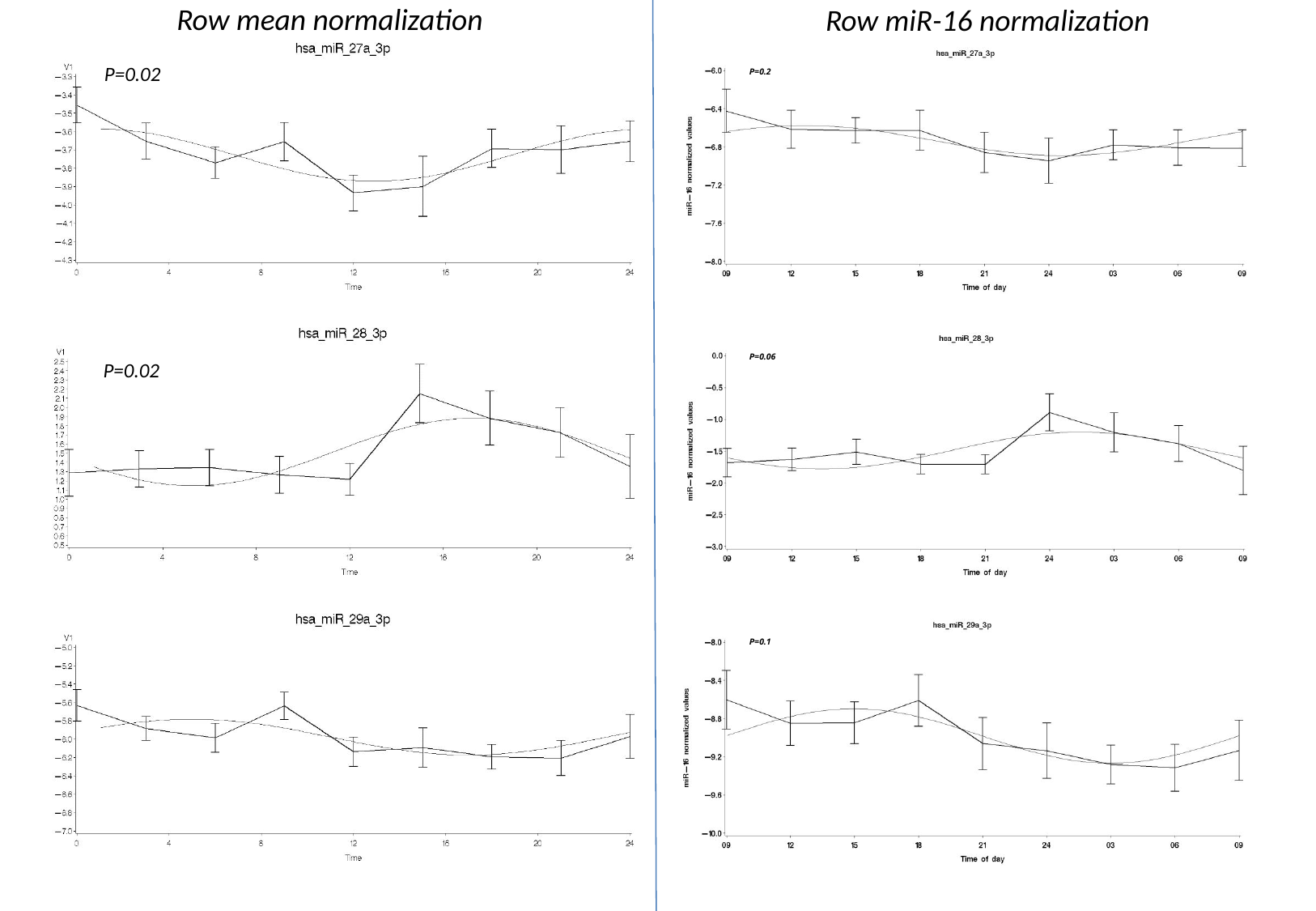

Row mean normalization
Row miR-16 normalization
P=0.02
P=0.2
P=0.06
P=0.02
P=0.1

## Slide 5
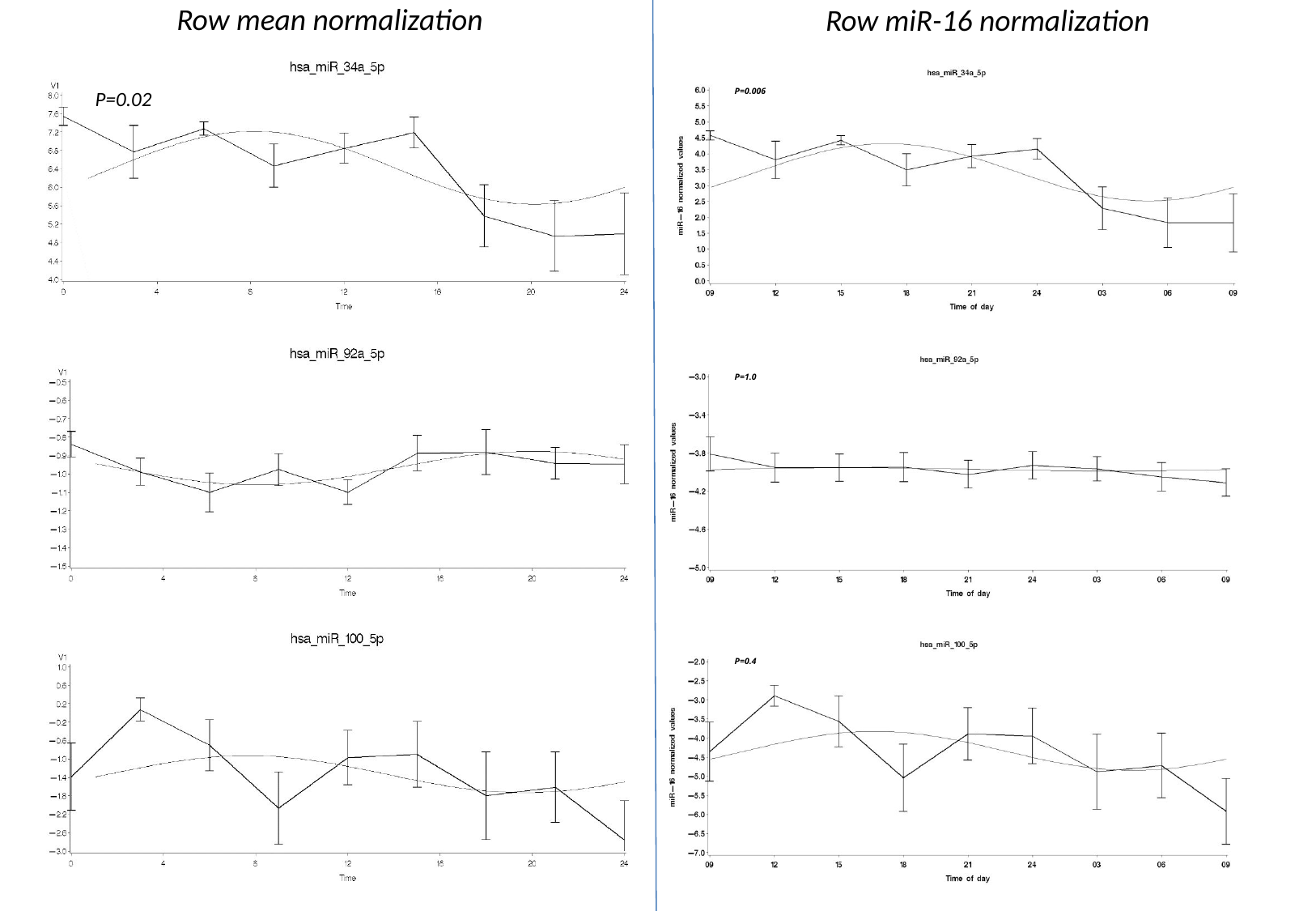

Row mean normalization
Row miR-16 normalization
P=0.02
P=0.006
P=1.0
P=0.4

## Slide 6
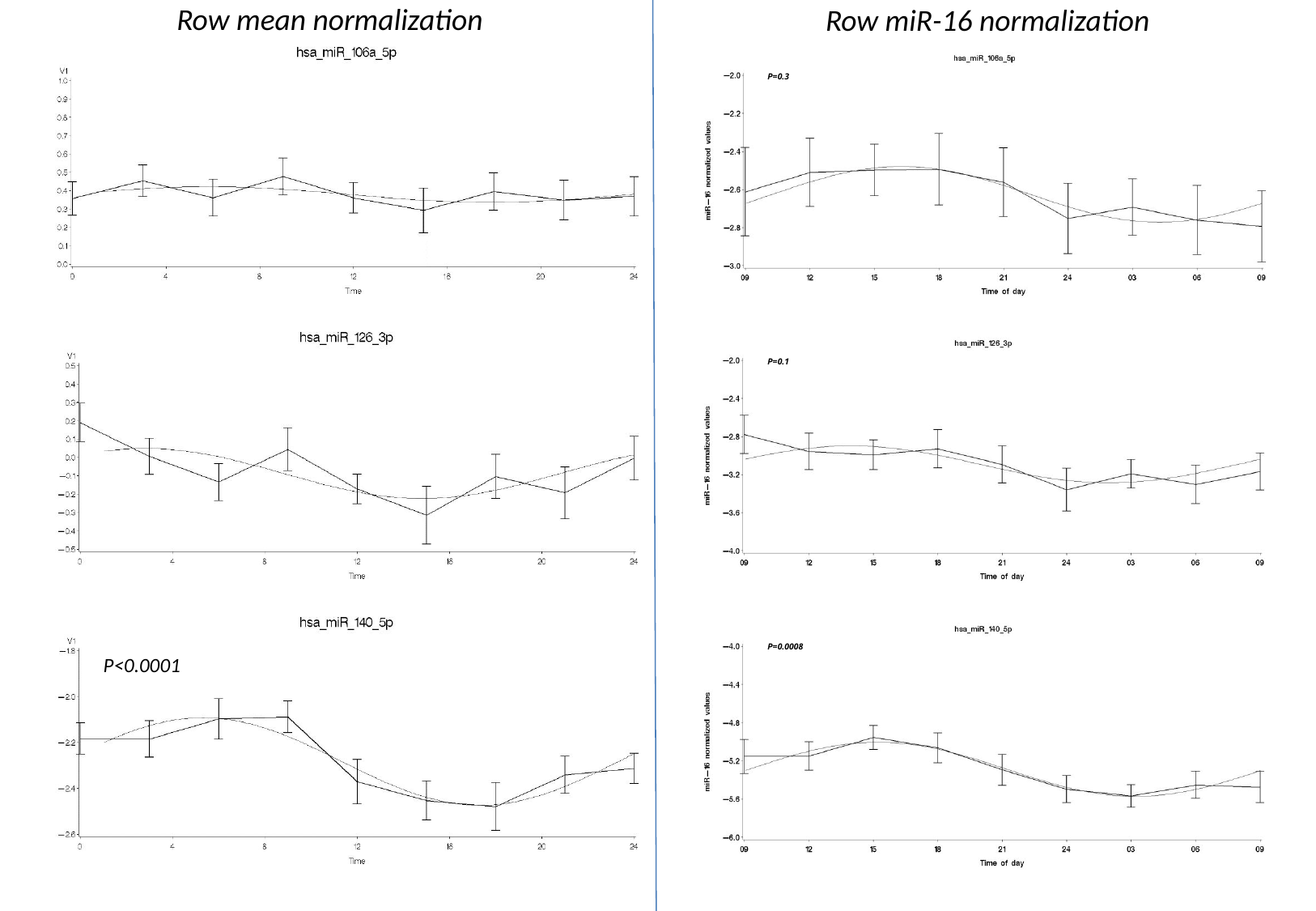

Row mean normalization
Row miR-16 normalization
P=0.3
P=0.1
P=0.0008
P<0.0001

## Slide 7
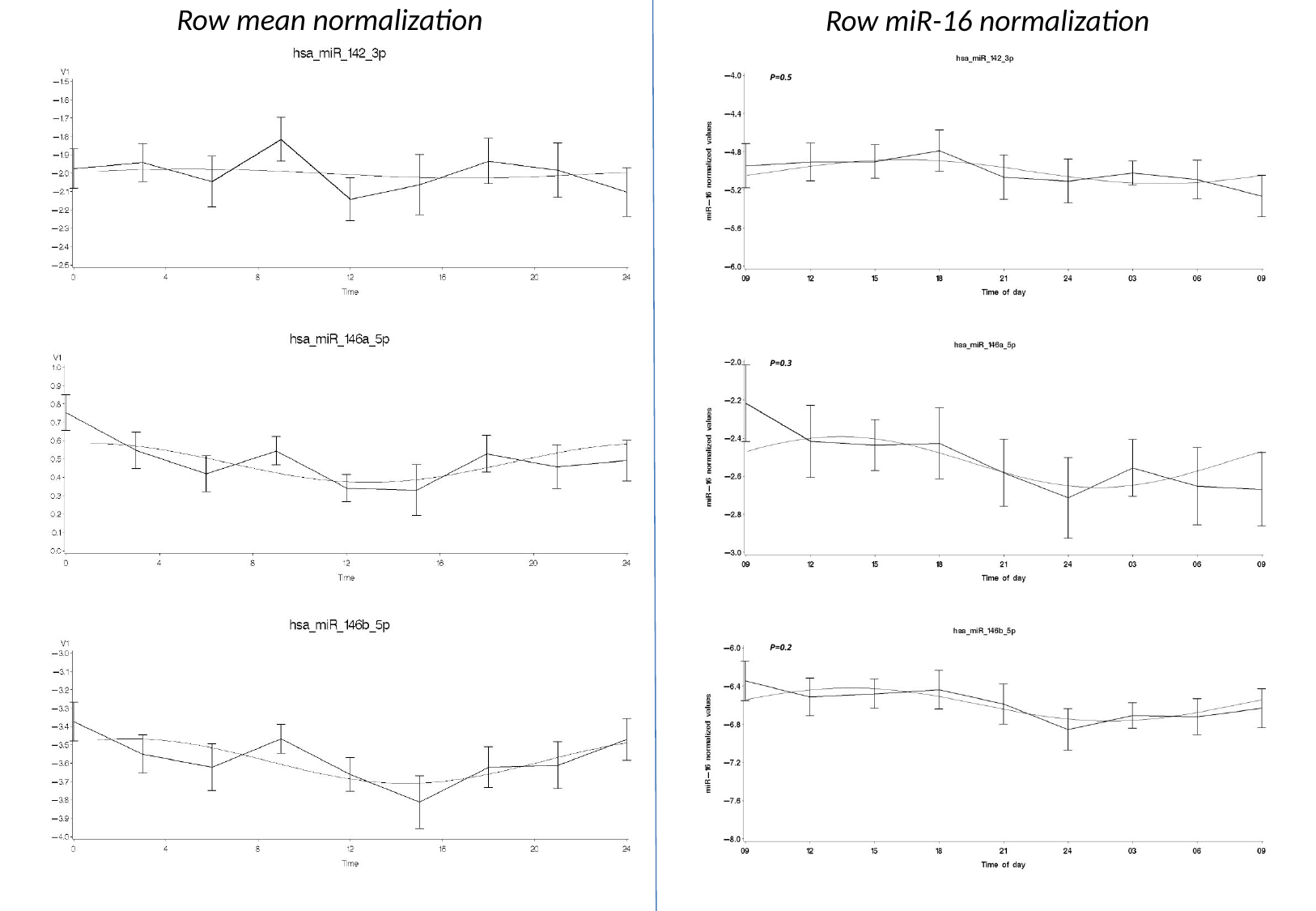

Row mean normalization
Row miR-16 normalization
P=0.5
P=0.3
P=0.2

## Slide 8
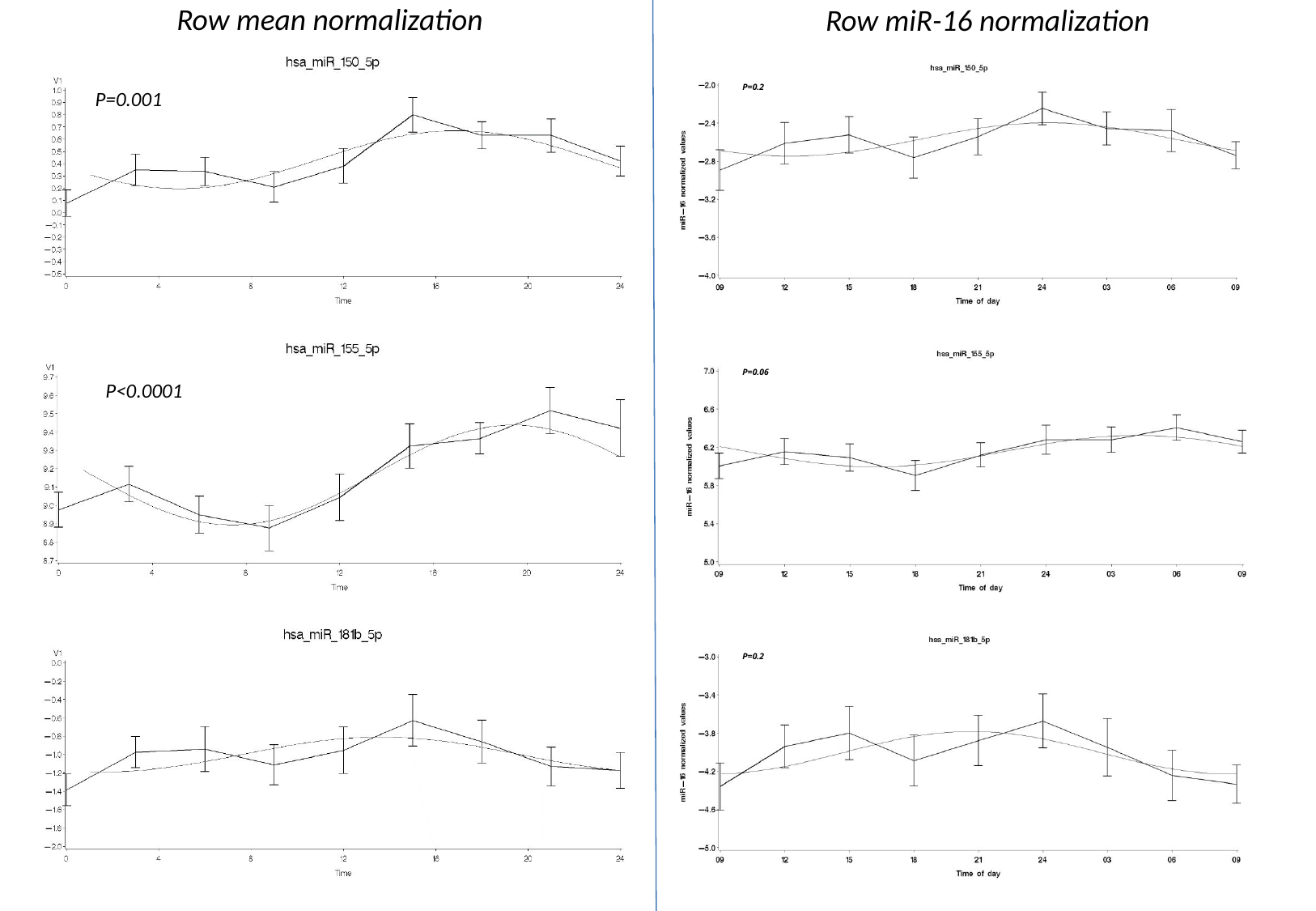

Row mean normalization
Row miR-16 normalization
P=0.2
P=0.001
P=0.06
P<0.0001
P=0.2

## Slide 9
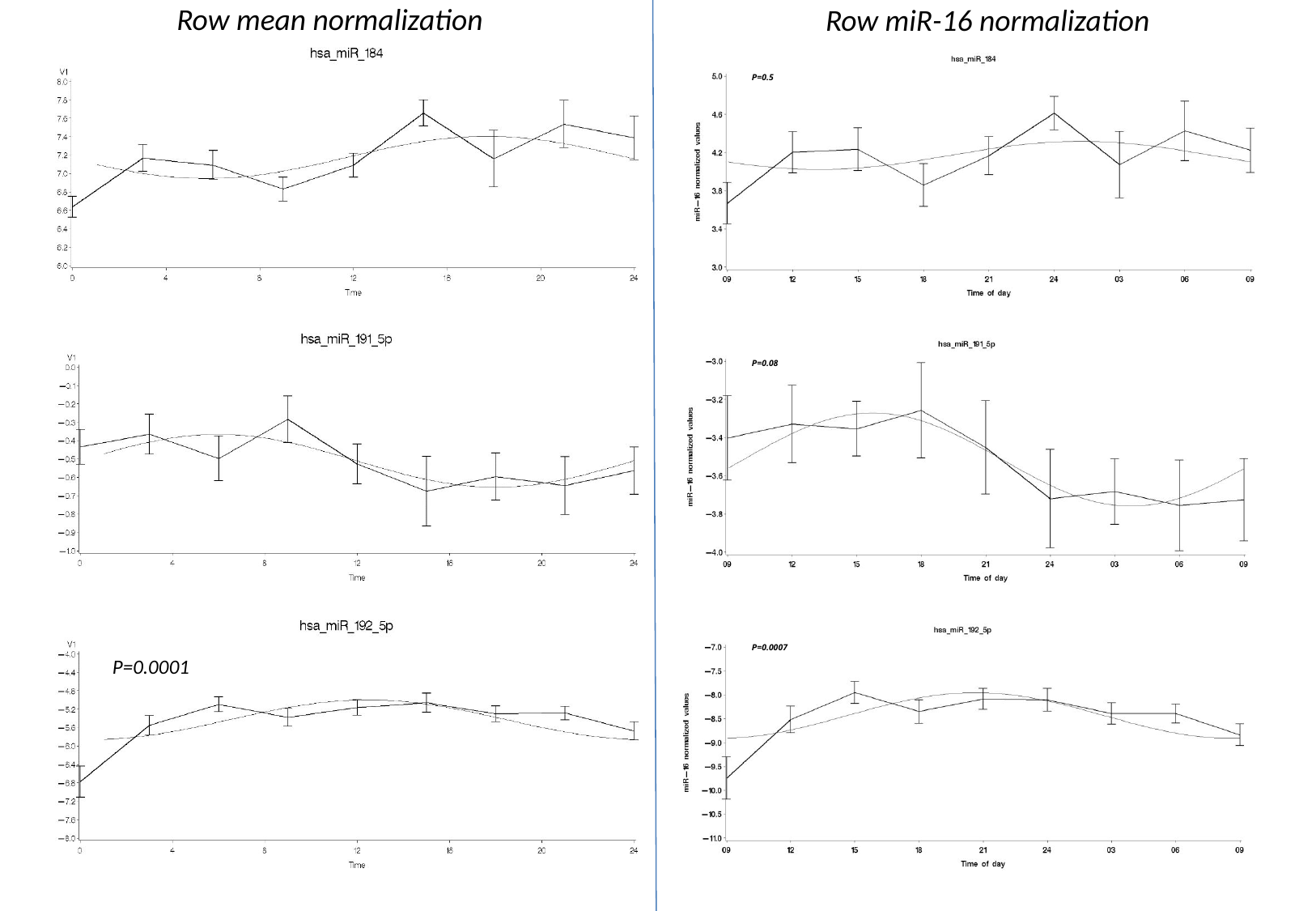

Row mean normalization
Row miR-16 normalization
P=0.5
P=0.08
P=0.0007
P=0.0001

## Slide 10
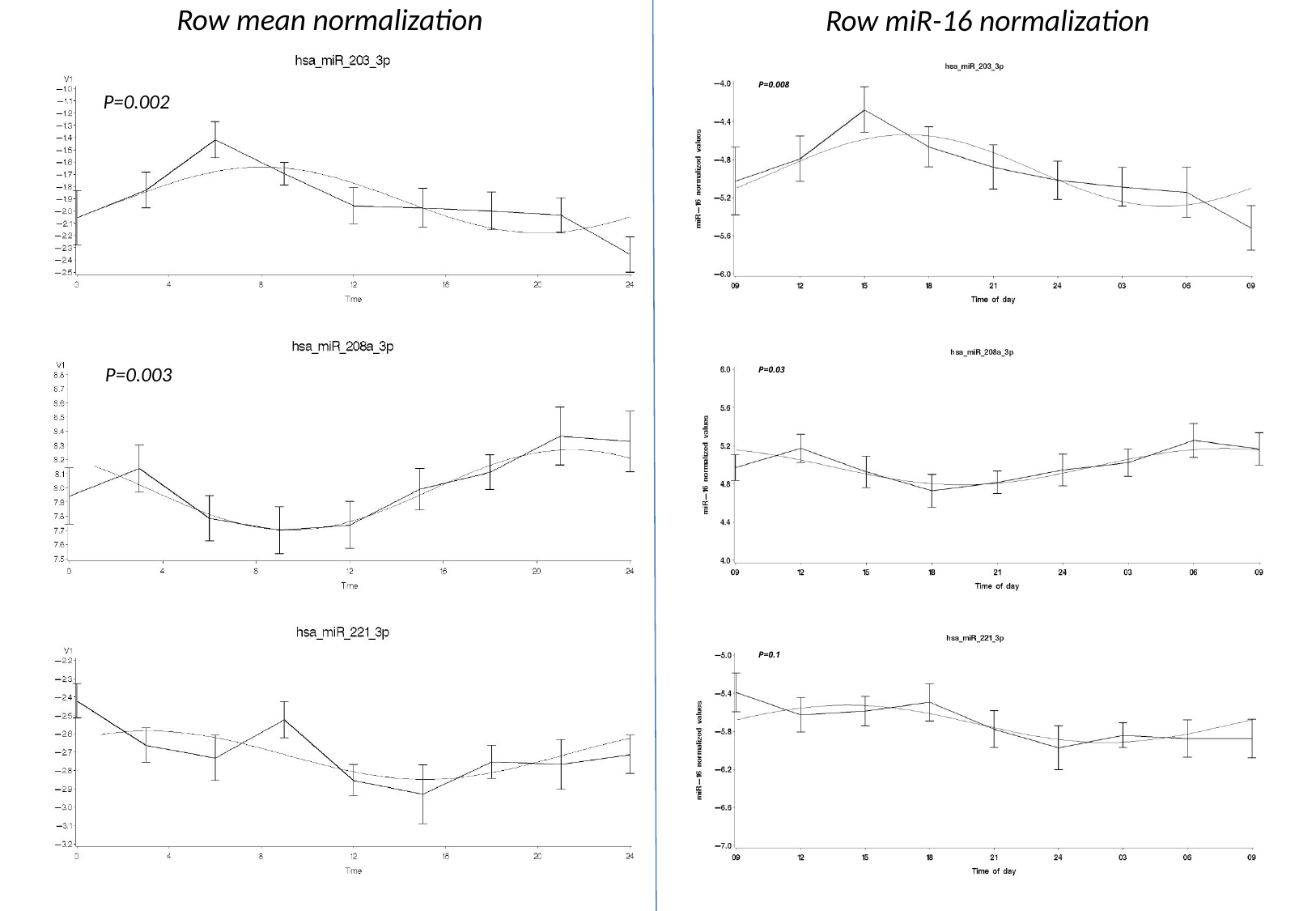

Row mean normalization
Row miR-16 normalization
P=0.008
P=0.002
P=0.003
P=0.03
P=0.1

## Slide 11
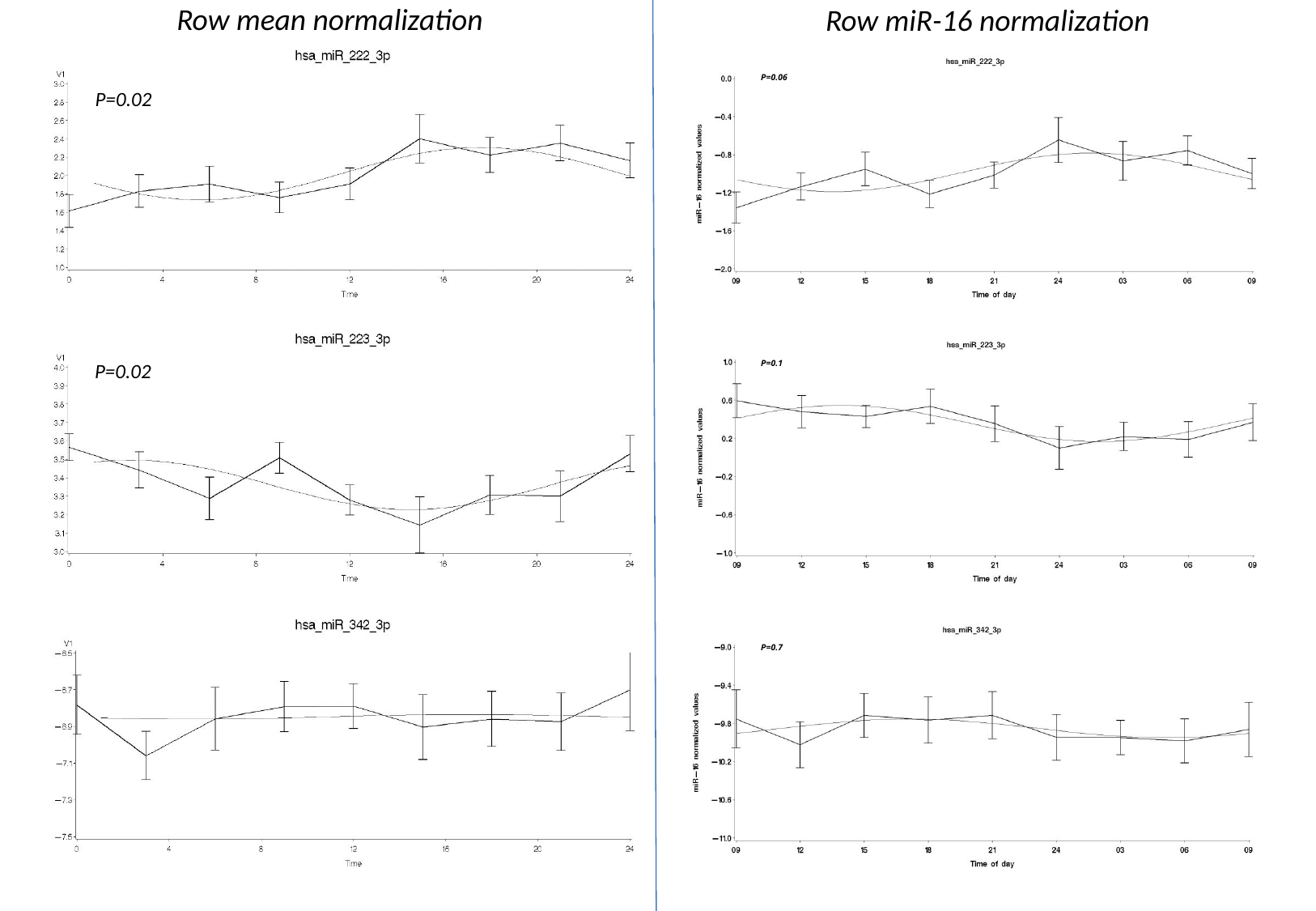

Row mean normalization
Row miR-16 normalization
P=0.06
P=0.02
P=0.1
P=0.02
P=0.7

## Slide 12
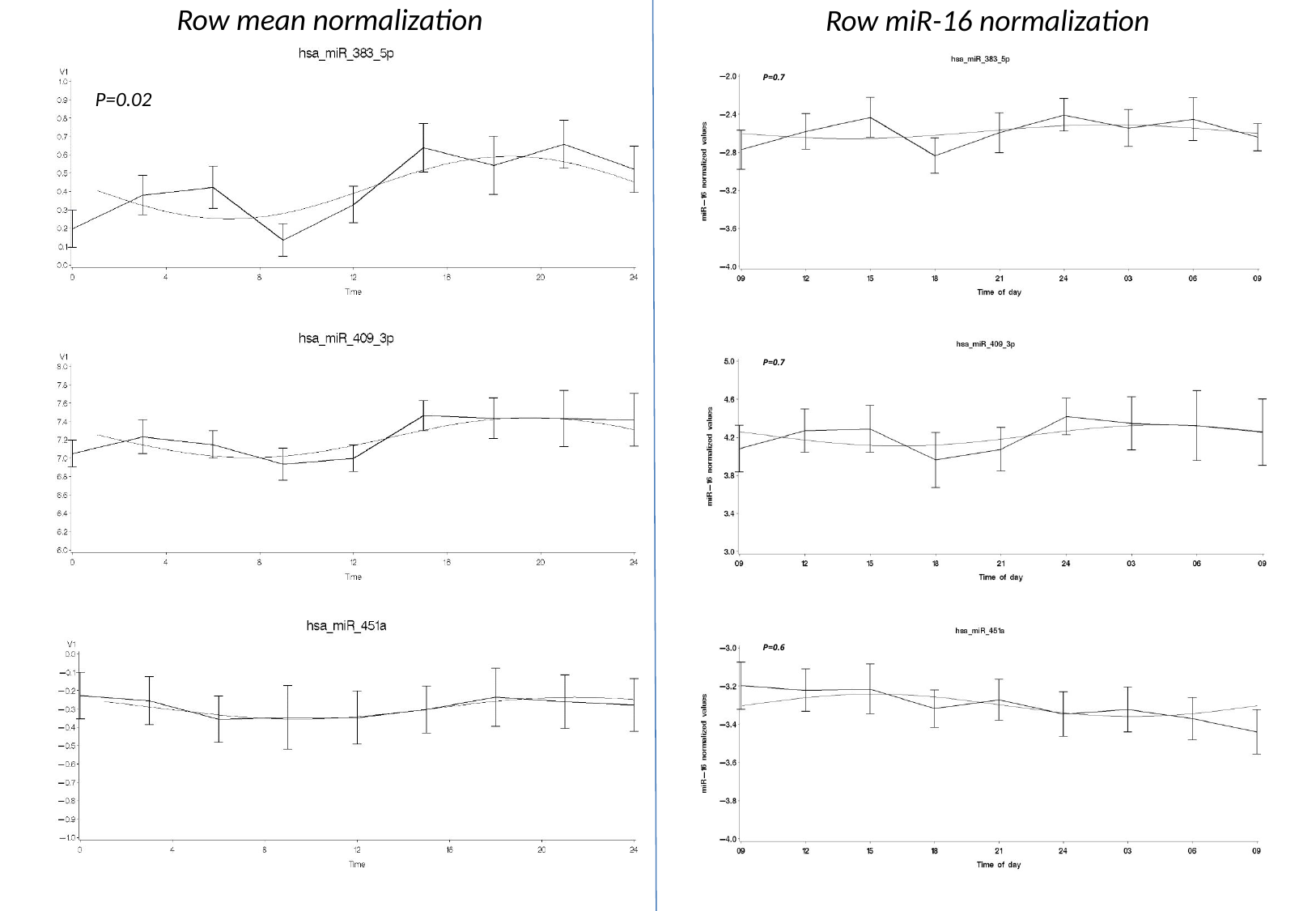

Row mean normalization
Row miR-16 normalization
P=0.7
P=0.02
P=0.7
P=0.6

## Slide 13
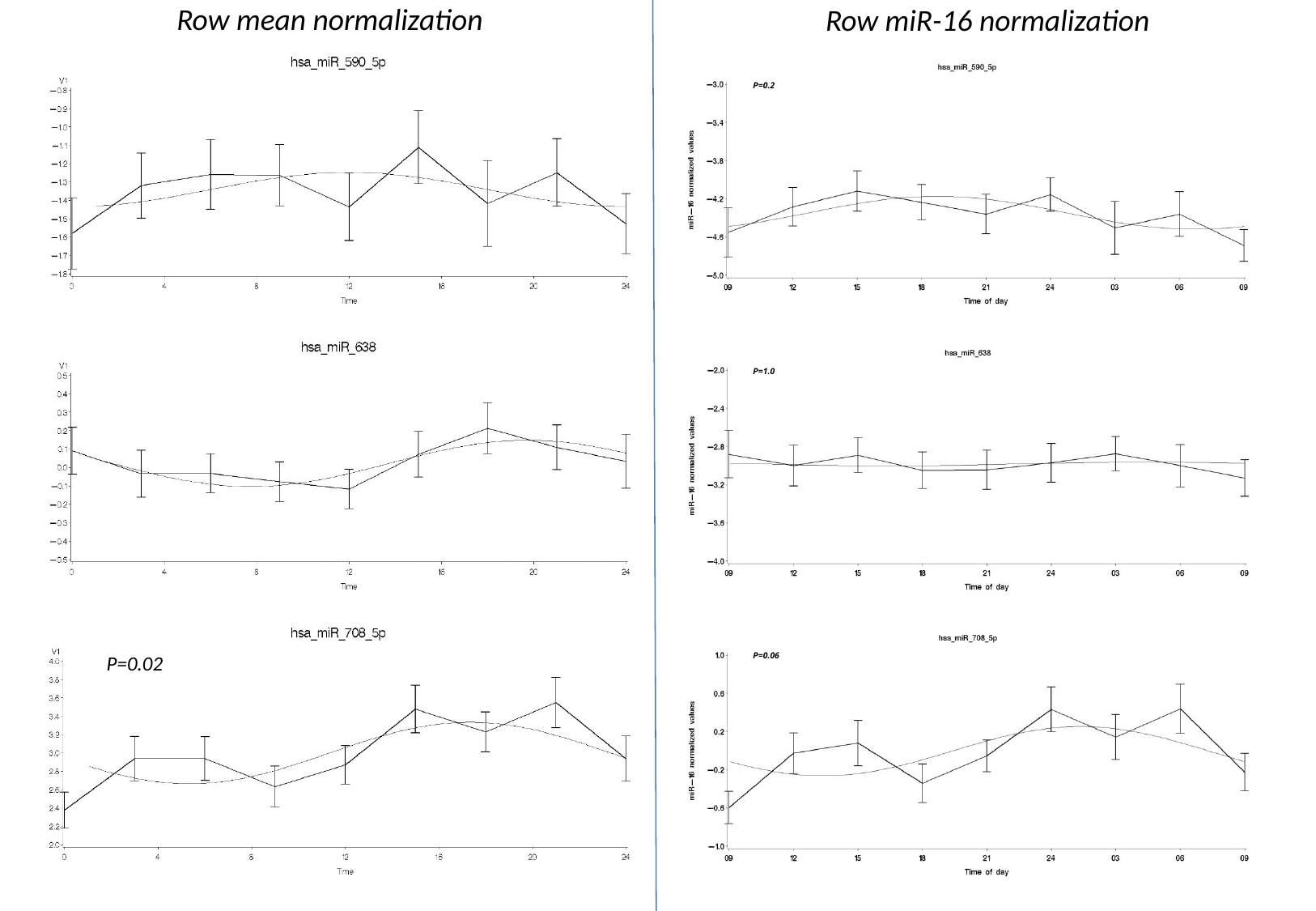

Row mean normalization
Row miR-16 normalization
P=0.2
P=1.0
P=0.02
P=0.06

## Slide 14
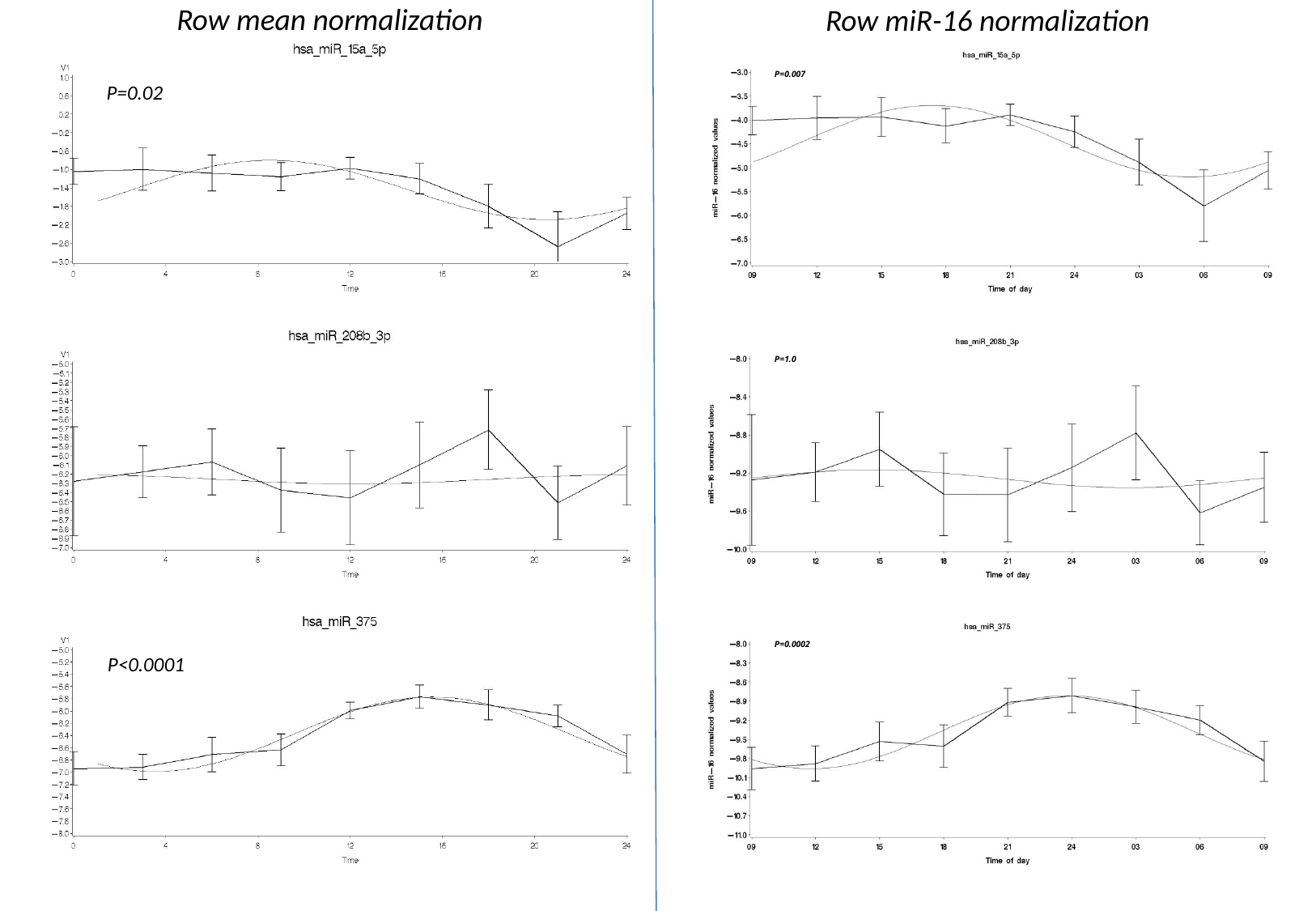

Row mean normalization
Row miR-16 normalization
P=0.007
P=0.02
P=1.0
P=0.0002
P<0.0001
