# Supplementary material for: Diurnal Variations of Human Circulating Cell-Free Micro-RNA
Source: PLoS One. 2016 Aug 5;11(8):e0160577. doi: 10.1371/journal.pone.0160577 (PMC4975411; doi:10.1371/journal.pone.0160577)
Supplement: S1 File — The p-values are indicated in red. (DOCX) [file pone.0160577.s004.docx]

**Supplementary Data File S4, RHYTHMICITY ANALYSIS, row mean normalization**

hsa_miR_22_3p 1

The MEANS Procedure

Variable Label Mean Std Dev Std Error Minimum Maximum Pr > |t|

ƒƒƒƒƒƒƒƒƒƒƒƒƒƒƒƒƒƒƒƒƒƒƒƒƒƒƒƒƒƒƒƒƒƒƒƒƒƒƒƒƒƒƒƒƒƒƒƒƒƒƒƒƒƒƒƒƒƒƒƒƒƒƒƒƒƒƒƒƒƒƒƒƒƒƒƒƒƒƒƒƒƒƒƒƒƒƒƒƒƒƒƒƒƒƒƒƒƒƒƒƒƒƒƒƒƒƒƒƒƒƒƒƒƒ

COS 0.0140986 0.1458271 0.0297668 -0.1549550 0.3193816 0.6402

SIN 0.0418024 0.1855733 0.0378800 -0.2128122 0.4120153 0.2812

Intercept Intercept -0.4665132 0.2225080 0.0454193 -0.9016717 -0.1457370 <.0001

ƒƒƒƒƒƒƒƒƒƒƒƒƒƒƒƒƒƒƒƒƒƒƒƒƒƒƒƒƒƒƒƒƒƒƒƒƒƒƒƒƒƒƒƒƒƒƒƒƒƒƒƒƒƒƒƒƒƒƒƒƒƒƒƒƒƒƒƒƒƒƒƒƒƒƒƒƒƒƒƒƒƒƒƒƒƒƒƒƒƒƒƒƒƒƒƒƒƒƒƒƒƒƒƒƒƒƒƒƒƒƒƒƒƒ

hsa_miR_22_3p 2

model test

The GLM Procedure

Number of observations 215

hsa_miR_22_3p 3

model test

The GLM Procedure

Dependent Variable: variabel

Sum of

Source DF Squares Mean Square F Value Pr > F

Model 2 0.20045722 0.10022861 0.62 **0.5409**

Error 212 34.48006651 0.16264182

Corrected Total 214 34.68052372

R-Square Coeff Var Root MSE variabel Mean

0.005780 -86.81995 0.403289 -0.464512

Source DF Type I SS Mean Square F Value Pr > F

COS 1 0.02303130 0.02303130 0.14 0.7071

SIN 1 0.17742592 0.17742592 1.09 0.2975

Source DF Type III SS Mean Square F Value Pr > F

COS 1 0.02289628 0.02289628 0.14 0.7079

SIN 1 0.17742592 0.17742592 1.09 0.2975

Standard

Parameter Estimate Error t Value Pr > |t|

Intercept -.4658700536 0.02781702 -16.75 <.0001

COS 0.0139699548 0.03723305 0.38 0.7079

SIN 0.0432172971 0.04137759 1.04 0.2975

AMPLITUDE, TMAX AND TMIN FOR hsa_miR_22_3p 4

Obs AMPL TMAX TMIN

1 0.088232 4.45 16.45

hsa_miR_26a_5p 5

The MEANS Procedure

Variable Label Mean Std Dev Std Error Minimum Maximum Pr > |t|

ƒƒƒƒƒƒƒƒƒƒƒƒƒƒƒƒƒƒƒƒƒƒƒƒƒƒƒƒƒƒƒƒƒƒƒƒƒƒƒƒƒƒƒƒƒƒƒƒƒƒƒƒƒƒƒƒƒƒƒƒƒƒƒƒƒƒƒƒƒƒƒƒƒƒƒƒƒƒƒƒƒƒƒƒƒƒƒƒƒƒƒƒƒƒƒƒƒƒƒƒƒƒƒƒƒƒƒƒƒƒƒƒƒƒ

COS -0.1101606 0.1845222 0.0376654 -0.4918648 0.1707285 0.0076

SIN 0.0950562 0.1577090 0.0321922 -0.2144759 0.3888998 0.0071

Intercept Intercept 1.8871005 0.5643534 0.1151982 0.7340062 2.8307047 <.0001

ƒƒƒƒƒƒƒƒƒƒƒƒƒƒƒƒƒƒƒƒƒƒƒƒƒƒƒƒƒƒƒƒƒƒƒƒƒƒƒƒƒƒƒƒƒƒƒƒƒƒƒƒƒƒƒƒƒƒƒƒƒƒƒƒƒƒƒƒƒƒƒƒƒƒƒƒƒƒƒƒƒƒƒƒƒƒƒƒƒƒƒƒƒƒƒƒƒƒƒƒƒƒƒƒƒƒƒƒƒƒƒƒƒƒ

hsa_miR_26a_5p 6

model test

The GLM Procedure

Number of observations 215

hsa_miR_26a_5p 7

model test

The GLM Procedure

Dependent Variable: variabel

Sum of

Source DF Squares Mean Square F Value Pr > F

Model 2 2.45496665 1.22748332 2.86 **0.0592**

Error 212 90.85007335 0.42853808

Corrected Total 214 93.30504000

R-Square Coeff Var Root MSE variabel Mean

0.026311 34.85773 0.654628 1.878000

Source DF Type I SS Mean Square F Value Pr > F

COS 1 1.44068330 1.44068330 3.36 0.0681

SIN 1 1.01428334 1.01428334 2.37 0.1254

Source DF Type III SS Mean Square F Value Pr > F

COS 1 1.44323919 1.44323919 3.37 0.0679

SIN 1 1.01428334 1.01428334 2.37 0.1254

Standard

Parameter Estimate Error t Value Pr > |t|

Intercept 1.890861569 0.04515327 41.88 <.0001

COS -0.110912796 0.06043760 -1.84 0.0679

SIN 0.103330544 0.06716512 1.54 0.1254

AMPLITUDE, TMAX AND TMIN FOR hsa_miR_26a_5p 8

Obs AMPL TMAX TMIN

1 0.29101 9.17 21.17

hsa_miR_27b_3p 9

The MEANS Procedure

Variable Label Mean Std Dev Std Error Minimum Maximum Pr > |t|

ƒƒƒƒƒƒƒƒƒƒƒƒƒƒƒƒƒƒƒƒƒƒƒƒƒƒƒƒƒƒƒƒƒƒƒƒƒƒƒƒƒƒƒƒƒƒƒƒƒƒƒƒƒƒƒƒƒƒƒƒƒƒƒƒƒƒƒƒƒƒƒƒƒƒƒƒƒƒƒƒƒƒƒƒƒƒƒƒƒƒƒƒƒƒƒƒƒƒƒƒƒƒƒƒƒƒƒƒƒƒƒƒƒƒ

COS 0.1000802 0.1819359 0.0371375 -0.1999728 0.3889095 0.0129

SIN 0.0449002 0.2313889 0.0472321 -0.4160749 0.5399442 0.3517

Intercept Intercept -1.5871270 0.3097060 0.0632185 -2.1732122 -1.0758102 <.0001

ƒƒƒƒƒƒƒƒƒƒƒƒƒƒƒƒƒƒƒƒƒƒƒƒƒƒƒƒƒƒƒƒƒƒƒƒƒƒƒƒƒƒƒƒƒƒƒƒƒƒƒƒƒƒƒƒƒƒƒƒƒƒƒƒƒƒƒƒƒƒƒƒƒƒƒƒƒƒƒƒƒƒƒƒƒƒƒƒƒƒƒƒƒƒƒƒƒƒƒƒƒƒƒƒƒƒƒƒƒƒƒƒƒƒ

hsa_miR_27b_3p 10

model test

The GLM Procedure

Number of observations 215

hsa_miR_27b_3p 11

model test

The GLM Procedure

Dependent Variable: variabel

Sum of

Source DF Squares Mean Square F Value Pr > F

Model 2 1.41916122 0.70958061 2.52 **0.0827**

Error 212 59.64473366 0.28134308

Corrected Total 214 61.06389488

R-Square Coeff Var Root MSE variabel Mean

0.023241 -33.72064 0.530418 -1.572977

Source DF Type I SS Mean Square F Value Pr > F

COS 1 1.16082653 1.16082653 4.13 0.0435

SIN 1 0.25833469 0.25833469 0.92 0.3390

Source DF Type III SS Mean Square F Value Pr > F

COS 1 1.15966746 1.15966746 4.12 0.0436

SIN 1 0.25833469 0.25833469 0.92 0.3390

Standard

Parameter Estimate Error t Value Pr > |t|

Intercept -1.583832378 0.03658581 -43.29 <.0001

COS 0.099421252 0.04897006 2.03 0.0436

SIN 0.052148333 0.05442108 0.96 0.3390

AMPLITUDE, TMAX AND TMIN FOR hsa_miR_27b_3p 12

Obs AMPL TMAX TMIN

1 0.21938 1.37 13.37

hsa_miR_30a_5p 13

The MEANS Procedure

Variable Label Mean Std Dev Std Error Minimum Maximum Pr > |t|

ƒƒƒƒƒƒƒƒƒƒƒƒƒƒƒƒƒƒƒƒƒƒƒƒƒƒƒƒƒƒƒƒƒƒƒƒƒƒƒƒƒƒƒƒƒƒƒƒƒƒƒƒƒƒƒƒƒƒƒƒƒƒƒƒƒƒƒƒƒƒƒƒƒƒƒƒƒƒƒƒƒƒƒƒƒƒƒƒƒƒƒƒƒƒƒƒƒƒƒƒƒƒƒƒƒƒƒƒƒƒƒƒƒƒ

COS 0.0276963 0.1054600 0.0215269 -0.1950225 0.2090123 0.2110

SIN -0.0237843 0.1673086 0.0341517 -0.3394759 0.3646536 0.4931

Intercept Intercept 1.6598468 0.1878572 0.0383462 1.2531885 1.9821967 <.0001

ƒƒƒƒƒƒƒƒƒƒƒƒƒƒƒƒƒƒƒƒƒƒƒƒƒƒƒƒƒƒƒƒƒƒƒƒƒƒƒƒƒƒƒƒƒƒƒƒƒƒƒƒƒƒƒƒƒƒƒƒƒƒƒƒƒƒƒƒƒƒƒƒƒƒƒƒƒƒƒƒƒƒƒƒƒƒƒƒƒƒƒƒƒƒƒƒƒƒƒƒƒƒƒƒƒƒƒƒƒƒƒƒƒƒ

hsa_miR_30a_5p 14

model test

The GLM Procedure

Number of observations 215

hsa_miR_30a_5p 15

model test

The GLM Procedure

Dependent Variable: variabel

Sum of

Source DF Squares Mean Square F Value Pr > F

Model 2 0.14334718 0.07167359 0.75 **0.4718**

Error 212 20.15362677 0.09506428

Corrected Total 214 20.29697395

R-Square Coeff Var Root MSE variabel Mean

0.007062 18.53951 0.308325 1.663070

Source DF Type I SS Mean Square F Value Pr > F

COS 1 0.08982061 0.08982061 0.94 0.3321

SIN 1 0.05352657 0.05352657 0.56 0.4539

Source DF Type III SS Mean Square F Value Pr > F

COS 1 0.08996720 0.08996720 0.95 0.3318

SIN 1 0.05352657 0.05352657 0.56 0.4539

Standard

Parameter Estimate Error t Value Pr > |t|

Intercept 1.659868159 0.02126685 78.05 <.0001

COS 0.027692018 0.02846565 0.97 0.3318

SIN -0.023737434 0.03163426 -0.75 0.4539

AMPLITUDE, TMAX AND TMIN FOR hsa_miR_30a_5p 16

Obs AMPL TMAX TMIN

1 0.073014 21.17 9.17

hsa_miR_103a_3p 17

The MEANS Procedure

Variable Label Mean Std Dev Std Error Minimum Maximum Pr > |t|

ƒƒƒƒƒƒƒƒƒƒƒƒƒƒƒƒƒƒƒƒƒƒƒƒƒƒƒƒƒƒƒƒƒƒƒƒƒƒƒƒƒƒƒƒƒƒƒƒƒƒƒƒƒƒƒƒƒƒƒƒƒƒƒƒƒƒƒƒƒƒƒƒƒƒƒƒƒƒƒƒƒƒƒƒƒƒƒƒƒƒƒƒƒƒƒƒƒƒƒƒƒƒƒƒƒƒƒƒƒƒƒƒƒƒ

COS 0.0174551 0.3003077 0.0613001 -0.3120704 0.9908698 0.7784

SIN 0.2488356 0.4337877 0.0885465 -0.7474748 1.5282805 0.0099

Intercept Intercept -1.0031873 0.6943224 0.1417280 -2.7934300 -0.1005656 <.0001

ƒƒƒƒƒƒƒƒƒƒƒƒƒƒƒƒƒƒƒƒƒƒƒƒƒƒƒƒƒƒƒƒƒƒƒƒƒƒƒƒƒƒƒƒƒƒƒƒƒƒƒƒƒƒƒƒƒƒƒƒƒƒƒƒƒƒƒƒƒƒƒƒƒƒƒƒƒƒƒƒƒƒƒƒƒƒƒƒƒƒƒƒƒƒƒƒƒƒƒƒƒƒƒƒƒƒƒƒƒƒƒƒƒƒ

hsa_miR_103a_3p 18

model test

The GLM Procedure

Number of observations 215

hsa_miR_103a_3p 19

model test

The GLM Procedure

Dependent Variable: variabel

Sum of

Source DF Squares Mean Square F Value Pr > F

Model 2 6.0560737 3.0280369 3.59 **0.0293**

Error 212 178.7796583 0.8433003

Corrected Total 214 184.8357321

R-Square Coeff Var Root MSE variabel Mean

0.032765 -91.72899 0.918314 -1.001116

Source DF Type I SS Mean Square F Value Pr > F

COS 1 0.03564268 0.03564268 0.04 0.8373

SIN 1 6.02043107 6.02043107 7.14 0.0081

Source DF Type III SS Mean Square F Value Pr > F

COS 1 0.03466975 0.03466975 0.04 0.8395

SIN 1 6.02043107 6.02043107 7.14 0.0081

Standard

Parameter Estimate Error t Value Pr > |t|

Intercept -1.001864301 0.06334111 -15.82 <.0001

COS 0.017190473 0.08478199 0.20 0.8395

SIN 0.251746165 0.09421937 2.67 0.0081

AMPLITUDE, TMAX AND TMIN FOR hsa_miR_103a_3p 20

Obs AMPL TMAX TMIN

1 0.49889 5.44 17.44

hsa_miR_106b_5p 21

The MEANS Procedure

Variable Label Mean Std Dev Std Error Minimum Maximum Pr > |t|

ƒƒƒƒƒƒƒƒƒƒƒƒƒƒƒƒƒƒƒƒƒƒƒƒƒƒƒƒƒƒƒƒƒƒƒƒƒƒƒƒƒƒƒƒƒƒƒƒƒƒƒƒƒƒƒƒƒƒƒƒƒƒƒƒƒƒƒƒƒƒƒƒƒƒƒƒƒƒƒƒƒƒƒƒƒƒƒƒƒƒƒƒƒƒƒƒƒƒƒƒƒƒƒƒƒƒƒƒƒƒƒƒƒƒ

COS -0.1457778 0.1664749 0.0339815 -0.5097223 0.2388853 0.0003

SIN 0.1483087 0.2403673 0.0490648 -0.2753643 0.5895153 0.0061

Intercept Intercept -4.2968023 0.5627745 0.1148759 -5.3639113 -3.3628718 <.0001

ƒƒƒƒƒƒƒƒƒƒƒƒƒƒƒƒƒƒƒƒƒƒƒƒƒƒƒƒƒƒƒƒƒƒƒƒƒƒƒƒƒƒƒƒƒƒƒƒƒƒƒƒƒƒƒƒƒƒƒƒƒƒƒƒƒƒƒƒƒƒƒƒƒƒƒƒƒƒƒƒƒƒƒƒƒƒƒƒƒƒƒƒƒƒƒƒƒƒƒƒƒƒƒƒƒƒƒƒƒƒƒƒƒƒ

hsa_miR_106b_5p 22

model test

The GLM Procedure

Number of observations 215

hsa_miR_106b_5p 23

model test

The GLM Procedure

Dependent Variable: variabel

Sum of

Source DF Squares Mean Square F Value Pr > F

Model 2 4.7183873 2.3591936 5.23 **0.0061**

Error 212 95.6652053 0.4512510

Corrected Total 214 100.3835926

R-Square Coeff Var Root MSE variabel Mean

0.047004 -15.57934 0.671752 -4.311814

Source DF Type I SS Mean Square F Value Pr > F

COS 1 2.50204067 2.50204067 5.54 0.0195

SIN 1 2.21634662 2.21634662 4.91 0.0277

Source DF Type III SS Mean Square F Value Pr > F

COS 1 2.50702030 2.50702030 5.56 0.0193

SIN 1 2.21634662 2.21634662 4.91 0.0277

Standard

Parameter Estimate Error t Value Pr > |t|

Intercept -4.294785614 0.04633441 -92.69 <.0001

COS -0.146181165 0.06201854 -2.36 0.0193

SIN 0.152745319 0.06892204 2.22 0.0277

AMPLITUDE, TMAX AND TMIN FOR hsa_miR_106b_5p 24

Obs AMPL TMAX TMIN

1 0.41592 8.58 20.58

hsa_miR_132_3p 25

The MEANS Procedure

Variable Label Mean Std Dev Std Error Minimum Maximum Pr > |t|

ƒƒƒƒƒƒƒƒƒƒƒƒƒƒƒƒƒƒƒƒƒƒƒƒƒƒƒƒƒƒƒƒƒƒƒƒƒƒƒƒƒƒƒƒƒƒƒƒƒƒƒƒƒƒƒƒƒƒƒƒƒƒƒƒƒƒƒƒƒƒƒƒƒƒƒƒƒƒƒƒƒƒƒƒƒƒƒƒƒƒƒƒƒƒƒƒƒƒƒƒƒƒƒƒƒƒƒƒƒƒƒƒƒƒ

COS -0.0126889 0.2518765 0.0514141 -0.6494934 0.3392697 0.8073

SIN 0.0215175 0.1703961 0.0347820 -0.2537745 0.3726130 0.5422

Intercept Intercept -1.6317286 0.2617170 0.0534228 -2.1864625 -1.0915606 <.0001

ƒƒƒƒƒƒƒƒƒƒƒƒƒƒƒƒƒƒƒƒƒƒƒƒƒƒƒƒƒƒƒƒƒƒƒƒƒƒƒƒƒƒƒƒƒƒƒƒƒƒƒƒƒƒƒƒƒƒƒƒƒƒƒƒƒƒƒƒƒƒƒƒƒƒƒƒƒƒƒƒƒƒƒƒƒƒƒƒƒƒƒƒƒƒƒƒƒƒƒƒƒƒƒƒƒƒƒƒƒƒƒƒƒƒ

hsa_miR_132_3p 26

model test

The GLM Procedure

Number of observations 215

hsa_miR_132_3p 27

model test

The GLM Procedure

Dependent Variable: variabel

Sum of

Source DF Squares Mean Square F Value Pr > F

Model 2 0.08174286 0.04087143 0.13 **0.8813**

Error 212 68.54398086 0.32332066

Corrected Total 214 68.62572372

R-Square Coeff Var Root MSE variabel Mean

0.001191 -34.85240 0.568613 -1.631488

Source DF Type I SS Mean Square F Value Pr > F

COS 1 0.01991219 0.01991219 0.06 0.8042

SIN 1 0.06183067 0.06183067 0.19 0.6623

Source DF Type III SS Mean Square F Value Pr > F

COS 1 0.01998644 0.01998644 0.06 0.8039

SIN 1 0.06183067 0.06183067 0.19 0.6623

Standard

Parameter Estimate Error t Value Pr > |t|

Intercept -1.629912732 0.03922033 -41.56 <.0001

COS -0.013052093 0.05249636 -0.25 0.8039

SIN 0.025512385 0.05833992 0.44 0.6623

AMPLITUDE, TMAX AND TMIN FOR hsa_miR_132_3p 28

Obs AMPL TMAX TMIN

1 0.049961 8.02 20.02

hsa_miR_139_5p 29

The MEANS Procedure

Variable Label Mean Std Dev Std Error Minimum Maximum Pr > |t|

ƒƒƒƒƒƒƒƒƒƒƒƒƒƒƒƒƒƒƒƒƒƒƒƒƒƒƒƒƒƒƒƒƒƒƒƒƒƒƒƒƒƒƒƒƒƒƒƒƒƒƒƒƒƒƒƒƒƒƒƒƒƒƒƒƒƒƒƒƒƒƒƒƒƒƒƒƒƒƒƒƒƒƒƒƒƒƒƒƒƒƒƒƒƒƒƒƒƒƒƒƒƒƒƒƒƒƒƒƒƒƒƒƒƒ

COS -0.0254991 0.3620487 0.0739029 -0.9696878 0.6699431 0.7332

SIN 0.3947803 0.4096028 0.0836098 -0.1930724 1.6327654 <.0001

Intercept Intercept -6.4034723 0.7142731 0.1458004 -8.0254144 -4.6722833 <.0001

ƒƒƒƒƒƒƒƒƒƒƒƒƒƒƒƒƒƒƒƒƒƒƒƒƒƒƒƒƒƒƒƒƒƒƒƒƒƒƒƒƒƒƒƒƒƒƒƒƒƒƒƒƒƒƒƒƒƒƒƒƒƒƒƒƒƒƒƒƒƒƒƒƒƒƒƒƒƒƒƒƒƒƒƒƒƒƒƒƒƒƒƒƒƒƒƒƒƒƒƒƒƒƒƒƒƒƒƒƒƒƒƒƒƒ

hsa_miR_139_5p 30

model test

The GLM Procedure

Number of observations 215

hsa_miR_139_5p 31

model test

The GLM Procedure

Dependent Variable: variabel

Sum of

Source DF Squares Mean Square F Value Pr > F

Model 2 15.4301173 7.7150586 7.77 **0.0006**

Error 212 210.5268576 0.9930512

Corrected Total 214 225.9569749

R-Square Coeff Var Root MSE variabel Mean

0.068288 -15.55852 0.996520 -6.404977

Source DF Type I SS Mean Square F Value Pr > F

COS 1 0.07793390 0.07793390 0.08 0.7796

SIN 1 15.35218337 15.35218337 15.46 0.0001

Source DF Type III SS Mean Square F Value Pr > F

COS 1 0.08026415 0.08026415 0.08 0.7765

SIN 1 15.35218337 15.35218337 15.46 0.0001

Standard

Parameter Estimate Error t Value Pr > |t|

Intercept -6.400187187 0.06873538 -93.11 <.0001

COS -0.026156118 0.09200222 -0.28 0.7765

SIN 0.402007489 0.10224331 3.93 0.0001

AMPLITUDE, TMAX AND TMIN FOR hsa_miR_139_5p 32

Obs AMPL TMAX TMIN

1 0.79121 6.15 18.15

hsa_miR_181a_5p 33

The MEANS Procedure

Variable Label Mean Std Dev Std Error Minimum Maximum Pr > |t|

ƒƒƒƒƒƒƒƒƒƒƒƒƒƒƒƒƒƒƒƒƒƒƒƒƒƒƒƒƒƒƒƒƒƒƒƒƒƒƒƒƒƒƒƒƒƒƒƒƒƒƒƒƒƒƒƒƒƒƒƒƒƒƒƒƒƒƒƒƒƒƒƒƒƒƒƒƒƒƒƒƒƒƒƒƒƒƒƒƒƒƒƒƒƒƒƒƒƒƒƒƒƒƒƒƒƒƒƒƒƒƒƒƒƒ

COS -0.0054584 0.3682438 0.0751674 -0.9331692 0.6871281 0.9427

SIN 0.1329049 0.3221839 0.0657655 -0.6917426 0.9322792 0.0551

Intercept Intercept -2.7574163 0.5991863 0.1223084 -4.4685698 -1.8090614 <.0001

ƒƒƒƒƒƒƒƒƒƒƒƒƒƒƒƒƒƒƒƒƒƒƒƒƒƒƒƒƒƒƒƒƒƒƒƒƒƒƒƒƒƒƒƒƒƒƒƒƒƒƒƒƒƒƒƒƒƒƒƒƒƒƒƒƒƒƒƒƒƒƒƒƒƒƒƒƒƒƒƒƒƒƒƒƒƒƒƒƒƒƒƒƒƒƒƒƒƒƒƒƒƒƒƒƒƒƒƒƒƒƒƒƒƒ

hsa_miR_181a_5p 34

model test

The GLM Procedure

Number of observations 215

hsa_miR_181a_5p 35

model test

The GLM Procedure

Dependent Variable: variabel

Sum of

Source DF Squares Mean Square F Value Pr > F

Model 2 1.7024028 0.8512014 1.00 **0.3686**

Error 212 179.9736605 0.8489324

Corrected Total 214 181.6760633

R-Square Coeff Var Root MSE variabel Mean

0.009371 -33.40399 0.921375 -2.758279

Source DF Type I SS Mean Square F Value Pr > F

COS 1 0.00342939 0.00342939 0.00 0.9494

SIN 1 1.69897341 1.69897341 2.00 0.1586

Source DF Type III SS Mean Square F Value Pr > F

COS 1 0.00359271 0.00359271 0.00 0.9482

SIN 1 1.69897341 1.69897341 2.00 0.1586

Standard

Parameter Estimate Error t Value Pr > |t|

Intercept -2.757039323 0.06355227 -43.38 <.0001

COS -0.005533798 0.08506463 -0.07 0.9482

SIN 0.133734162 0.09453347 1.41 0.1586

AMPLITUDE, TMAX AND TMIN FOR hsa_miR_181a_5p 36

Obs AMPL TMAX TMIN

1 0.26603 6.09 18.09

hsa_miR_199a_b_3p 37

The MEANS Procedure

Variable Label Mean Std Dev Std Error Minimum Maximum Pr > |t|

ƒƒƒƒƒƒƒƒƒƒƒƒƒƒƒƒƒƒƒƒƒƒƒƒƒƒƒƒƒƒƒƒƒƒƒƒƒƒƒƒƒƒƒƒƒƒƒƒƒƒƒƒƒƒƒƒƒƒƒƒƒƒƒƒƒƒƒƒƒƒƒƒƒƒƒƒƒƒƒƒƒƒƒƒƒƒƒƒƒƒƒƒƒƒƒƒƒƒƒƒƒƒƒƒƒƒƒƒƒƒƒƒƒƒ

COS -0.0525425 0.1665613 0.0339992 -0.4573861 0.2312023 0.1359

SIN 0.1761831 0.1505137 0.0307235 -0.1621682 0.4226866 <.0001

Intercept Intercept 1.2627152 0.3547250 0.0724079 0.4757133 1.9206294 <.0001

ƒƒƒƒƒƒƒƒƒƒƒƒƒƒƒƒƒƒƒƒƒƒƒƒƒƒƒƒƒƒƒƒƒƒƒƒƒƒƒƒƒƒƒƒƒƒƒƒƒƒƒƒƒƒƒƒƒƒƒƒƒƒƒƒƒƒƒƒƒƒƒƒƒƒƒƒƒƒƒƒƒƒƒƒƒƒƒƒƒƒƒƒƒƒƒƒƒƒƒƒƒƒƒƒƒƒƒƒƒƒƒƒƒƒ

hsa_miR_199a_b_3p 38

model test

The GLM Procedure

Number of observations 215

hsa_miR_199a_b_3p 39

model test

The GLM Procedure

Dependent Variable: variabel

Sum of

Source DF Squares Mean Square F Value Pr > F

Model 2 3.64445004 1.82222502 8.34 **0.0003**

Error 212 46.29945880 0.21839367

Corrected Total 214 49.94390884

R-Square Coeff Var Root MSE variabel Mean

0.072971 37.07021 0.467326 1.260651

Source DF Type I SS Mean Square F Value Pr > F

COS 1 0.33354706 0.33354706 1.53 0.2179

SIN 1 3.31090297 3.31090297 15.16 0.0001

Source DF Type III SS Mean Square F Value Pr > F

COS 1 0.33577273 0.33577273 1.54 0.2164

SIN 1 3.31090297 3.31090297 15.16 0.0001

Standard

Parameter Estimate Error t Value Pr > |t|

Intercept 1.267491329 0.03223403 39.32 <.0001

COS -0.053497712 0.04314520 -1.24 0.2164

SIN 0.186690533 0.04794785 3.89 0.0001

AMPLITUDE, TMAX AND TMIN FOR hsa_miR_199a_b_3p 40

Obs AMPL TMAX TMIN

1 0.36770 7.06 19.06

hsa_miR_206 41

The MEANS Procedure

Variable Label Mean Std Dev Std Error Minimum Maximum Pr > |t|

ƒƒƒƒƒƒƒƒƒƒƒƒƒƒƒƒƒƒƒƒƒƒƒƒƒƒƒƒƒƒƒƒƒƒƒƒƒƒƒƒƒƒƒƒƒƒƒƒƒƒƒƒƒƒƒƒƒƒƒƒƒƒƒƒƒƒƒƒƒƒƒƒƒƒƒƒƒƒƒƒƒƒƒƒƒƒƒƒƒƒƒƒƒƒƒƒƒƒƒƒƒƒƒƒƒƒƒƒƒƒƒƒƒƒ

COS -0.0815415 0.3038875 0.0620308 -0.4962313 0.8672689 0.2016

SIN -0.2973425 0.2726115 0.0556466 -0.9792336 0.3612742 <.0001

Intercept Intercept -3.9484142 0.6054659 0.1235902 -5.6741410 -2.9497435 <.0001

ƒƒƒƒƒƒƒƒƒƒƒƒƒƒƒƒƒƒƒƒƒƒƒƒƒƒƒƒƒƒƒƒƒƒƒƒƒƒƒƒƒƒƒƒƒƒƒƒƒƒƒƒƒƒƒƒƒƒƒƒƒƒƒƒƒƒƒƒƒƒƒƒƒƒƒƒƒƒƒƒƒƒƒƒƒƒƒƒƒƒƒƒƒƒƒƒƒƒƒƒƒƒƒƒƒƒƒƒƒƒƒƒƒƒ

hsa_miR_206 42

model test

The GLM Procedure

Number of observations 215

hsa_miR_206 43

model test

The GLM Procedure

Dependent Variable: variabel

Sum of

Source DF Squares Mean Square F Value Pr > F

Model 2 9.9016559 4.9508280 7.19 **0.0009**

Error 212 145.9194725 0.6882994

Corrected Total 214 155.8211284

R-Square Coeff Var Root MSE variabel Mean

0.063545 -20.94111 0.829638 -3.961767

Source DF Type I SS Mean Square F Value Pr > F

COS 1 0.76351943 0.76351943 1.11 0.2934

SIN 1 9.13813649 9.13813649 13.28 0.0003

Source DF Type III SS Mean Square F Value Pr > F

COS 1 0.75794283 0.75794283 1.10 0.2952

SIN 1 9.13813649 9.13813649 13.28 0.0003

Standard

Parameter Estimate Error t Value Pr > |t|

Intercept -3.954237729 0.05722466 -69.10 <.0001

COS -0.080376801 0.07659514 -1.05 0.2952

SIN -0.310154359 0.08512121 -3.64 0.0003

AMPLITUDE, TMAX AND TMIN FOR hsa_miR_206 44

Obs AMPL TMAX TMIN

1 0.61664 16.59 4.59

hsa_miR_218_5p 45

The MEANS Procedure

Variable Label Mean Std Dev Std Error Minimum Maximum Pr > |t|

ƒƒƒƒƒƒƒƒƒƒƒƒƒƒƒƒƒƒƒƒƒƒƒƒƒƒƒƒƒƒƒƒƒƒƒƒƒƒƒƒƒƒƒƒƒƒƒƒƒƒƒƒƒƒƒƒƒƒƒƒƒƒƒƒƒƒƒƒƒƒƒƒƒƒƒƒƒƒƒƒƒƒƒƒƒƒƒƒƒƒƒƒƒƒƒƒƒƒƒƒƒƒƒƒƒƒƒƒƒƒƒƒƒƒ

COS 0.1033252 0.3133982 0.0639721 -0.5185692 0.6590621 0.1199

SIN -0.1846638 0.4907801 0.1001801 -0.7372539 1.6085981 0.0782

Intercept Intercept 2.6926279 0.7570307 0.1545282 1.3513979 3.9679857 <.0001

ƒƒƒƒƒƒƒƒƒƒƒƒƒƒƒƒƒƒƒƒƒƒƒƒƒƒƒƒƒƒƒƒƒƒƒƒƒƒƒƒƒƒƒƒƒƒƒƒƒƒƒƒƒƒƒƒƒƒƒƒƒƒƒƒƒƒƒƒƒƒƒƒƒƒƒƒƒƒƒƒƒƒƒƒƒƒƒƒƒƒƒƒƒƒƒƒƒƒƒƒƒƒƒƒƒƒƒƒƒƒƒƒƒƒ

hsa_miR_218_5p 46

model test

The GLM Procedure

Number of observations 215

hsa_miR_218_5p 47

model test

The GLM Procedure

Dependent Variable: variabel

Sum of

Source DF Squares Mean Square F Value Pr > F

Model 2 4.8757003 2.4378502 1.68 **0.1882**

Error 212 307.0376327 1.4482907

Corrected Total 214 311.9133330

R-Square Coeff Var Root MSE variabel Mean

0.015632 44.56299 1.203450 2.700558

Source DF Type I SS Mean Square F Value Pr > F

COS 1 1.27046628 1.27046628 0.88 0.3500

SIN 1 3.60523405 3.60523405 2.49 0.1161

Source DF Type III SS Mean Square F Value Pr > F

COS 1 1.27499481 1.27499481 0.88 0.3492

SIN 1 3.60523405 3.60523405 2.49 0.1161

Standard

Parameter Estimate Error t Value Pr > |t|

Intercept 2.688015079 0.08300847 32.38 <.0001

COS 0.104247753 0.11110673 0.94 0.3492

SIN -0.194812043 0.12347441 -1.58 0.1161

AMPLITUDE, TMAX AND TMIN FOR hsa_miR_218_5p 48

Obs AMPL TMAX TMIN

1 0.42321 19.57 7.57

hsa_miR_363_3p 49

The MEANS Procedure

Variable Label Mean Std Dev Std Error Minimum Maximum Pr > |t|

ƒƒƒƒƒƒƒƒƒƒƒƒƒƒƒƒƒƒƒƒƒƒƒƒƒƒƒƒƒƒƒƒƒƒƒƒƒƒƒƒƒƒƒƒƒƒƒƒƒƒƒƒƒƒƒƒƒƒƒƒƒƒƒƒƒƒƒƒƒƒƒƒƒƒƒƒƒƒƒƒƒƒƒƒƒƒƒƒƒƒƒƒƒƒƒƒƒƒƒƒƒƒƒƒƒƒƒƒƒƒƒƒƒƒ

COS 0.1139356 0.3368346 0.0687561 -0.5767428 1.0696645 0.1111

SIN -0.2639047 0.2837557 0.0579214 -0.9048796 0.2893503 0.0001

Intercept Intercept 2.6645807 0.6772261 0.1382382 1.6005676 3.7958732 <.0001

ƒƒƒƒƒƒƒƒƒƒƒƒƒƒƒƒƒƒƒƒƒƒƒƒƒƒƒƒƒƒƒƒƒƒƒƒƒƒƒƒƒƒƒƒƒƒƒƒƒƒƒƒƒƒƒƒƒƒƒƒƒƒƒƒƒƒƒƒƒƒƒƒƒƒƒƒƒƒƒƒƒƒƒƒƒƒƒƒƒƒƒƒƒƒƒƒƒƒƒƒƒƒƒƒƒƒƒƒƒƒƒƒƒƒ

hsa_miR_363_3p 50

model test

The GLM Procedure

Number of observations 215

hsa_miR_363_3p 51

model test

The GLM Procedure

Dependent Variable: variabel

Sum of

Source DF Squares Mean Square F Value Pr > F

Model 2 8.5626547 4.2813274 5.17 **0.0064**

Error 212 175.5457201 0.8280458

Corrected Total 214 184.1083749

R-Square Coeff Var Root MSE variabel Mean

0.046509 34.01788 0.909970 2.674977

Source DF Type I SS Mean Square F Value Pr > F

COS 1 1.53570768 1.53570768 1.85 0.1747

SIN 1 7.02694706 7.02694706 8.49 0.0040

Source DF Type III SS Mean Square F Value Pr > F

COS 1 1.54266081 1.54266081 1.86 0.1737

SIN 1 7.02694706 7.02694706 8.49 0.0040

Standard

Parameter Estimate Error t Value Pr > |t|

Intercept 2.660911424 0.06276560 42.39 <.0001

COS 0.114669447 0.08401168 1.36 0.1737

SIN -0.271977144 0.09336331 -2.91 0.0040

AMPLITUDE, TMAX AND TMIN FOR hsa_miR_363_3p 52

Obs AMPL TMAX TMIN

1 0.57490 19.33 7.33

hsa_miR_378a_3p 53

The MEANS Procedure

Variable Label Mean Std Dev Std Error Minimum Maximum Pr > |t|

ƒƒƒƒƒƒƒƒƒƒƒƒƒƒƒƒƒƒƒƒƒƒƒƒƒƒƒƒƒƒƒƒƒƒƒƒƒƒƒƒƒƒƒƒƒƒƒƒƒƒƒƒƒƒƒƒƒƒƒƒƒƒƒƒƒƒƒƒƒƒƒƒƒƒƒƒƒƒƒƒƒƒƒƒƒƒƒƒƒƒƒƒƒƒƒƒƒƒƒƒƒƒƒƒƒƒƒƒƒƒƒƒƒƒ

COS -0.0594550 0.2630756 0.0537001 -0.5929090 0.4455310 0.2797

SIN -0.2065822 0.3218129 0.0656898 -0.7103553 0.3542462 0.0045

Intercept Intercept 9.3562029 0.5379595 0.1098105 8.2282743 10.0541864 <.0001

ƒƒƒƒƒƒƒƒƒƒƒƒƒƒƒƒƒƒƒƒƒƒƒƒƒƒƒƒƒƒƒƒƒƒƒƒƒƒƒƒƒƒƒƒƒƒƒƒƒƒƒƒƒƒƒƒƒƒƒƒƒƒƒƒƒƒƒƒƒƒƒƒƒƒƒƒƒƒƒƒƒƒƒƒƒƒƒƒƒƒƒƒƒƒƒƒƒƒƒƒƒƒƒƒƒƒƒƒƒƒƒƒƒƒ

hsa_miR_378a_3p 54

model test

The GLM Procedure

Number of observations 215

hsa_miR_378a_3p 55

model test

The GLM Procedure

Dependent Variable: variabel

Sum of

Source DF Squares Mean Square F Value Pr > F

Model 2 4.7302683 2.3651342 4.20 **0.0163**

Error 212 119.4636740 0.5635079

Corrected Total 214 124.1939423

R-Square Coeff Var Root MSE variabel Mean

0.038088 8.030651 0.750672 9.347581

Source DF Type I SS Mean Square F Value Pr > F

COS 1 0.40907291 0.40907291 0.73 0.3952

SIN 1 4.32119542 4.32119542 7.67 0.0061

Source DF Type III SS Mean Square F Value Pr > F

COS 1 0.40626564 0.40626564 0.72 0.3968

SIN 1 4.32119542 4.32119542 7.67 0.0061

Standard

Parameter Estimate Error t Value Pr > |t|

Intercept 9.353158256 0.05177791 180.64 <.0001

COS -0.058846080 0.06930467 -0.85 0.3968

SIN -0.213280437 0.07701921 -2.77 0.0061

AMPLITUDE, TMAX AND TMIN FOR hsa_miR_378a_3p 56

Obs AMPL TMAX TMIN

1 0.42994 16.56 4.56

hsa_miR_483_5p 57

The MEANS Procedure

Variable Label Mean Std Dev Std Error Minimum Maximum Pr > |t|

ƒƒƒƒƒƒƒƒƒƒƒƒƒƒƒƒƒƒƒƒƒƒƒƒƒƒƒƒƒƒƒƒƒƒƒƒƒƒƒƒƒƒƒƒƒƒƒƒƒƒƒƒƒƒƒƒƒƒƒƒƒƒƒƒƒƒƒƒƒƒƒƒƒƒƒƒƒƒƒƒƒƒƒƒƒƒƒƒƒƒƒƒƒƒƒƒƒƒƒƒƒƒƒƒƒƒƒƒƒƒƒƒƒƒ

COS 0.0674813 0.1617488 0.0330168 -0.1957838 0.4745503 0.0526

SIN -0.1574459 0.1218907 0.0248808 -0.3954324 0.0436396 <.0001

Intercept Intercept 4.8454399 0.3114328 0.0635710 4.2226036 5.3000128 <.0001

ƒƒƒƒƒƒƒƒƒƒƒƒƒƒƒƒƒƒƒƒƒƒƒƒƒƒƒƒƒƒƒƒƒƒƒƒƒƒƒƒƒƒƒƒƒƒƒƒƒƒƒƒƒƒƒƒƒƒƒƒƒƒƒƒƒƒƒƒƒƒƒƒƒƒƒƒƒƒƒƒƒƒƒƒƒƒƒƒƒƒƒƒƒƒƒƒƒƒƒƒƒƒƒƒƒƒƒƒƒƒƒƒƒƒ

hsa_miR_483_5p 58

model test

The GLM Procedure

Number of observations 215

hsa_miR_483_5p 59

model test

The GLM Procedure

Dependent Variable: variabel

Sum of

Source DF Squares Mean Square F Value Pr > F

Model 2 2.95902120 1.47951060 8.02 **0.0004**

Error 212 39.11381415 0.18449912

Corrected Total 214 42.07283535

R-Square Coeff Var Root MSE variabel Mean

0.070331 8.851440 0.429534 4.852698

Source DF Type I SS Mean Square F Value Pr > F

COS 1 0.53513932 0.53513932 2.90 0.0900

SIN 1 2.42388188 2.42388188 13.14 0.0004

Source DF Type III SS Mean Square F Value Pr > F

COS 1 0.53754994 0.53754994 2.91 0.0893

SIN 1 2.42388188 2.42388188 13.14 0.0004

Standard

Parameter Estimate Error t Value Pr > |t|

Intercept 4.844398665 0.02962727 163.51 <.0001

COS 0.067689593 0.03965607 1.71 0.0893

SIN -0.159736732 0.04407032 -3.62 0.0004

AMPLITUDE, TMAX AND TMIN FOR hsa_miR_483_5p 60

Obs AMPL TMAX TMIN

1 0.34260 19.33 7.33

hsa_miR_659_3p 61

The MEANS Procedure

Variable Label Mean Std Dev Std Error Minimum Maximum Pr > |t|

ƒƒƒƒƒƒƒƒƒƒƒƒƒƒƒƒƒƒƒƒƒƒƒƒƒƒƒƒƒƒƒƒƒƒƒƒƒƒƒƒƒƒƒƒƒƒƒƒƒƒƒƒƒƒƒƒƒƒƒƒƒƒƒƒƒƒƒƒƒƒƒƒƒƒƒƒƒƒƒƒƒƒƒƒƒƒƒƒƒƒƒƒƒƒƒƒƒƒƒƒƒƒƒƒƒƒƒƒƒƒƒƒƒƒ

COS 0.0600258 0.3232100 0.0659750 -0.8015820 0.8691009 0.3723

SIN -0.1383132 0.3037475 0.0620022 -0.6479773 0.4266295 0.0357

Intercept Intercept -0.5028899 0.6132328 0.1251756 -1.8465082 0.5504554 0.0005

ƒƒƒƒƒƒƒƒƒƒƒƒƒƒƒƒƒƒƒƒƒƒƒƒƒƒƒƒƒƒƒƒƒƒƒƒƒƒƒƒƒƒƒƒƒƒƒƒƒƒƒƒƒƒƒƒƒƒƒƒƒƒƒƒƒƒƒƒƒƒƒƒƒƒƒƒƒƒƒƒƒƒƒƒƒƒƒƒƒƒƒƒƒƒƒƒƒƒƒƒƒƒƒƒƒƒƒƒƒƒƒƒƒƒ

hsa_miR_659_3p 62

model test

The GLM Procedure

Number of observations 215

hsa_miR_659_3p 63

model test

The GLM Procedure

Dependent Variable: variabel

Sum of

Source DF Squares Mean Square F Value Pr > F

Model 2 2.3347669 1.1673835 1.28 **0.2795**

Error 212 192.9918870 0.9103391

Corrected Total 214 195.3266540

R-Square Coeff Var Root MSE variabel Mean

0.011953 -191.9483 0.954117 -0.497070

Source DF Type I SS Mean Square F Value Pr > F

COS 1 0.42525972 0.42525972 0.47 0.4951

SIN 1 1.90950720 1.90950720 2.10 0.1490

Source DF Type III SS Mean Square F Value Pr > F

COS 1 0.42716705 0.42716705 0.47 0.4941

SIN 1 1.90950720 1.90950720 2.10 0.1490

Standard

Parameter Estimate Error t Value Pr > |t|

Intercept -.5044649232 0.06581064 -7.67 <.0001

COS 0.0603408420 0.08808746 0.69 0.4941

SIN -.1417782850 0.09789278 -1.45 0.1490

AMPLITUDE, TMAX AND TMIN FOR hsa_miR_659_3p 64

Obs AMPL TMAX TMIN

1 0.30155 19.34 7.34

hsa_miR_122_5p 65

The MEANS Procedure

Variable Label Mean Std Dev Std Error Minimum Maximum Pr > |t|

ƒƒƒƒƒƒƒƒƒƒƒƒƒƒƒƒƒƒƒƒƒƒƒƒƒƒƒƒƒƒƒƒƒƒƒƒƒƒƒƒƒƒƒƒƒƒƒƒƒƒƒƒƒƒƒƒƒƒƒƒƒƒƒƒƒƒƒƒƒƒƒƒƒƒƒƒƒƒƒƒƒƒƒƒƒƒƒƒƒƒƒƒƒƒƒƒƒƒƒƒƒƒƒƒƒƒƒƒƒƒƒƒƒƒ

COS -0.1294169 0.3716365 0.0758600 -0.9275805 0.5132824 0.1015

SIN 0.0805877 0.6199897 0.1265549 -0.8739266 0.9955293 0.5306

Intercept Intercept -1.7808021 1.1500152 0.2347459 -4.3081425 0.0820769 <.0001

ƒƒƒƒƒƒƒƒƒƒƒƒƒƒƒƒƒƒƒƒƒƒƒƒƒƒƒƒƒƒƒƒƒƒƒƒƒƒƒƒƒƒƒƒƒƒƒƒƒƒƒƒƒƒƒƒƒƒƒƒƒƒƒƒƒƒƒƒƒƒƒƒƒƒƒƒƒƒƒƒƒƒƒƒƒƒƒƒƒƒƒƒƒƒƒƒƒƒƒƒƒƒƒƒƒƒƒƒƒƒƒƒƒƒ

hsa_miR_122_5p 66

model test

The GLM Procedure

Number of observations 215

hsa_miR_122_5p 67

model test

The GLM Procedure

Dependent Variable: variabel

Sum of

Source DF Squares Mean Square F Value Pr > F

Model 2 2.7724575 1.3862287 0.70 **0.4953**

Error 212 416.9419518 1.9667073

Corrected Total 214 419.7144093

R-Square Coeff Var Root MSE variabel Mean

0.006606 -78.29514 1.402393 -1.791163

Source DF Type I SS Mean Square F Value Pr > F

COS 1 1.99043999 1.99043999 1.01 0.3156

SIN 1 0.78201746 0.78201746 0.40 0.5290

Source DF Type III SS Mean Square F Value Pr > F

COS 1 1.99307705 1.99307705 1.01 0.3152

SIN 1 0.78201746 0.78201746 0.40 0.5290

Standard

Parameter Estimate Error t Value Pr > |t|

Intercept -1.776191311 0.09673071 -18.36 <.0001

COS -0.130339046 0.12947394 -1.01 0.3152

SIN 0.090731334 0.14388613 0.63 0.5290

AMPLITUDE, TMAX AND TMIN FOR hsa_miR_122_5p 68

Obs AMPL TMAX TMIN

1 0.30491 9.52 21.52

hsa_miR_147a 69

The MEANS Procedure

Variable Label Mean Std Dev Std Error Minimum Maximum Pr > |t|

ƒƒƒƒƒƒƒƒƒƒƒƒƒƒƒƒƒƒƒƒƒƒƒƒƒƒƒƒƒƒƒƒƒƒƒƒƒƒƒƒƒƒƒƒƒƒƒƒƒƒƒƒƒƒƒƒƒƒƒƒƒƒƒƒƒƒƒƒƒƒƒƒƒƒƒƒƒƒƒƒƒƒƒƒƒƒƒƒƒƒƒƒƒƒƒƒƒƒƒƒƒƒƒƒƒƒƒƒƒƒƒƒƒƒ

COS 0.1341007 0.2642383 0.0539374 -0.3313774 0.7808001 0.0206

SIN 0.0446569 0.3467397 0.0707779 -0.6285014 0.7345369 0.5343

Intercept Intercept -0.6591941 0.6141870 0.1253704 -1.8380944 0.2367327 <.0001

ƒƒƒƒƒƒƒƒƒƒƒƒƒƒƒƒƒƒƒƒƒƒƒƒƒƒƒƒƒƒƒƒƒƒƒƒƒƒƒƒƒƒƒƒƒƒƒƒƒƒƒƒƒƒƒƒƒƒƒƒƒƒƒƒƒƒƒƒƒƒƒƒƒƒƒƒƒƒƒƒƒƒƒƒƒƒƒƒƒƒƒƒƒƒƒƒƒƒƒƒƒƒƒƒƒƒƒƒƒƒƒƒƒƒ

hsa_miR_147a 70

model test

The GLM Procedure

Number of observations 215

hsa_miR_147a 71

model test

The GLM Procedure

Dependent Variable: variabel

Sum of

Source DF Squares Mean Square F Value Pr > F

Model 2 2.2864471 1.1432236 1.62 **0.2009**

Error 212 149.8748552 0.7069569

Corrected Total 214 152.1613023

R-Square Coeff Var Root MSE variabel Mean

0.015026 -130.2403 0.840807 -0.645581

Source DF Type I SS Mean Square F Value Pr > F

COS 1 2.11852273 2.11852273 3.00 0.0849

SIN 1 0.16792438 0.16792438 0.24 0.6265

Source DF Type III SS Mean Square F Value Pr > F

COS 1 2.11725921 2.11725921 2.99 0.0850

SIN 1 0.16792438 0.16792438 0.24 0.6265

Standard

Parameter Estimate Error t Value Pr > |t|

Intercept -.6603817304 0.05799506 -11.39 <.0001

COS 0.1343381954 0.07762631 1.73 0.0850

SIN 0.0420441856 0.08626717 0.49 0.6265

AMPLITUDE, TMAX AND TMIN FOR hsa_miR_147a 72

Obs AMPL TMAX TMIN

1 0.28268 1.14 13.14

hsa_miR_335_5p 73

The MEANS Procedure

Variable Label Mean Std Dev Std Error Minimum Maximum Pr > |t|

ƒƒƒƒƒƒƒƒƒƒƒƒƒƒƒƒƒƒƒƒƒƒƒƒƒƒƒƒƒƒƒƒƒƒƒƒƒƒƒƒƒƒƒƒƒƒƒƒƒƒƒƒƒƒƒƒƒƒƒƒƒƒƒƒƒƒƒƒƒƒƒƒƒƒƒƒƒƒƒƒƒƒƒƒƒƒƒƒƒƒƒƒƒƒƒƒƒƒƒƒƒƒƒƒƒƒƒƒƒƒƒƒƒƒ

COS -0.0226593 0.1775785 0.0362481 -0.5689569 0.2472174 0.5380

SIN 0.0661677 0.1053959 0.0215138 -0.0928338 0.2816511 0.0053

Intercept Intercept -0.3029218 0.3802750 0.0776233 -0.8734492 0.4939530 0.0007

ƒƒƒƒƒƒƒƒƒƒƒƒƒƒƒƒƒƒƒƒƒƒƒƒƒƒƒƒƒƒƒƒƒƒƒƒƒƒƒƒƒƒƒƒƒƒƒƒƒƒƒƒƒƒƒƒƒƒƒƒƒƒƒƒƒƒƒƒƒƒƒƒƒƒƒƒƒƒƒƒƒƒƒƒƒƒƒƒƒƒƒƒƒƒƒƒƒƒƒƒƒƒƒƒƒƒƒƒƒƒƒƒƒƒ

hsa_miR_335_5p 74

model test

The GLM Procedure

Number of observations 215

hsa_miR_335_5p 75

model test

The GLM Procedure

Dependent Variable: variabel

Sum of

Source DF Squares Mean Square F Value Pr > F

Model 2 0.53686810 0.26843405 1.10 **0.3349**

Error 212 51.75258027 0.24411594

Corrected Total 214 52.28944837

R-Square Coeff Var Root MSE variabel Mean

0.010267 -162.6510 0.494081 -0.303767

Source DF Type I SS Mean Square F Value Pr > F

COS 1 0.06208294 0.06208294 0.25 0.6146

SIN 1 0.47478516 0.47478516 1.94 0.1646

Source DF Type III SS Mean Square F Value Pr > F

COS 1 0.06244648 0.06244648 0.26 0.6135

SIN 1 0.47478516 0.47478516 1.94 0.1646

Standard

Parameter Estimate Error t Value Pr > |t|

Intercept -.3008632534 0.03407945 -8.83 <.0001

COS -.0230710022 0.04561530 -0.51 0.6135

SIN 0.0706964548 0.05069290 1.39 0.1646

AMPLITUDE, TMAX AND TMIN FOR hsa_miR_335_5p 76

Obs AMPL TMAX TMIN

1 0.13988 7.16 19.16

hsa_miR_379_5p 77

The MEANS Procedure

Variable Label Mean Std Dev Std Error Minimum Maximum Pr > |t|

ƒƒƒƒƒƒƒƒƒƒƒƒƒƒƒƒƒƒƒƒƒƒƒƒƒƒƒƒƒƒƒƒƒƒƒƒƒƒƒƒƒƒƒƒƒƒƒƒƒƒƒƒƒƒƒƒƒƒƒƒƒƒƒƒƒƒƒƒƒƒƒƒƒƒƒƒƒƒƒƒƒƒƒƒƒƒƒƒƒƒƒƒƒƒƒƒƒƒƒƒƒƒƒƒƒƒƒƒƒƒƒƒƒƒ

COS 0.0060866 0.2552363 0.0520999 -0.4106930 0.4785490 0.9080

SIN -0.2258161 0.2732296 0.0557728 -0.6020674 0.2361396 0.0005

Intercept Intercept 0.9735109 0.6945292 0.1417702 -0.2281294 2.2499772 <.0001

ƒƒƒƒƒƒƒƒƒƒƒƒƒƒƒƒƒƒƒƒƒƒƒƒƒƒƒƒƒƒƒƒƒƒƒƒƒƒƒƒƒƒƒƒƒƒƒƒƒƒƒƒƒƒƒƒƒƒƒƒƒƒƒƒƒƒƒƒƒƒƒƒƒƒƒƒƒƒƒƒƒƒƒƒƒƒƒƒƒƒƒƒƒƒƒƒƒƒƒƒƒƒƒƒƒƒƒƒƒƒƒƒƒƒ

hsa_miR_379_5p 78

model test

The GLM Procedure

Number of observations 215

hsa_miR_379_5p 79

model test

The GLM Procedure

Dependent Variable: variabel

Sum of

Source DF Squares Mean Square F Value Pr > F

Model 2 5.5387183 2.7693592 2.99 **0.0525**

Error 212 196.4263440 0.9265394

Corrected Total 214 201.9650623

R-Square Coeff Var Root MSE variabel Mean

0.027424 99.39598 0.962569 0.968419

Source DF Type I SS Mean Square F Value Pr > F

COS 1 0.00619609 0.00619609 0.01 0.9349

SIN 1 5.53252223 5.53252223 5.97 0.0154

Source DF Type III SS Mean Square F Value Pr > F

COS 1 0.00659382 0.00659382 0.01 0.9328

SIN 1 5.53252223 5.53252223 5.97 0.0154

Standard

Parameter Estimate Error t Value Pr > |t|

Intercept 0.9664592783 0.06639364 14.56 <.0001

COS 0.0074968856 0.08886780 0.08 0.9328

SIN -.2413296332 0.09875998 -2.44 0.0154

AMPLITUDE, TMAX AND TMIN FOR hsa_miR_379_5p 80

Obs AMPL TMAX TMIN

1 0.45180 18.06 6.06

hsa_miR_10a_5p 81

The MEANS Procedure

Variable Label Mean Std Dev Std Error Minimum Maximum Pr > |t|

ƒƒƒƒƒƒƒƒƒƒƒƒƒƒƒƒƒƒƒƒƒƒƒƒƒƒƒƒƒƒƒƒƒƒƒƒƒƒƒƒƒƒƒƒƒƒƒƒƒƒƒƒƒƒƒƒƒƒƒƒƒƒƒƒƒƒƒƒƒƒƒƒƒƒƒƒƒƒƒƒƒƒƒƒƒƒƒƒƒƒƒƒƒƒƒƒƒƒƒƒƒƒƒƒƒƒƒƒƒƒƒƒƒƒ

COS -0.2441178 0.6790196 0.1386043 -2.0674672 1.1291583 0.0915

SIN 0.2885511 0.8267978 0.1687694 -1.2590002 2.7540918 0.1008

Intercept Intercept -4.9135130 0.6982919 0.1425382 -7.0062501 -4.0686837 <.0001

ƒƒƒƒƒƒƒƒƒƒƒƒƒƒƒƒƒƒƒƒƒƒƒƒƒƒƒƒƒƒƒƒƒƒƒƒƒƒƒƒƒƒƒƒƒƒƒƒƒƒƒƒƒƒƒƒƒƒƒƒƒƒƒƒƒƒƒƒƒƒƒƒƒƒƒƒƒƒƒƒƒƒƒƒƒƒƒƒƒƒƒƒƒƒƒƒƒƒƒƒƒƒƒƒƒƒƒƒƒƒƒƒƒƒ

hsa_miR_10a_5p 82

model test

The GLM Procedure

Number of observations 215

NOTE: Due to missing values, only 212 observations can be used in this analysis.

hsa_miR_10a_5p 83

model test

The GLM Procedure

Dependent Variable: variabel

Sum of

Source DF Squares Mean Square F Value Pr > F

Model 2 13.0893845 6.5446923 2.92 **0.0560**

Error 209 467.8991966 2.2387521

Corrected Total 211 480.9885811

R-Square Coeff Var Root MSE variabel Mean

0.027214 -30.43485 1.496246 -4.916226

Source DF Type I SS Mean Square F Value Pr > F

COS 1 5.58746115 5.58746115 2.50 0.1157

SIN 1 7.50192339 7.50192339 3.35 0.0686

Source DF Type III SS Mean Square F Value Pr > F

COS 1 5.52930868 5.52930868 2.47 0.1176

SIN 1 7.50192339 7.50192339 3.35 0.0686

Standard

Parameter Estimate Error t Value Pr > |t|

Intercept -4.892357655 0.10384304 -47.11 <.0001

COS -0.219211219 0.13948592 -1.57 0.1176

SIN 0.281757653 0.15391906 1.83 0.0686

AMPLITUDE, TMAX AND TMIN FOR hsa_miR_10a_5p 84

Obs AMPL TMAX TMIN

1 0.75592 8.41 20.41

hsa_miR_125a_5p 85

The MEANS Procedure

Variable Label Mean Std Dev Std Error Minimum Maximum Pr > |t|

ƒƒƒƒƒƒƒƒƒƒƒƒƒƒƒƒƒƒƒƒƒƒƒƒƒƒƒƒƒƒƒƒƒƒƒƒƒƒƒƒƒƒƒƒƒƒƒƒƒƒƒƒƒƒƒƒƒƒƒƒƒƒƒƒƒƒƒƒƒƒƒƒƒƒƒƒƒƒƒƒƒƒƒƒƒƒƒƒƒƒƒƒƒƒƒƒƒƒƒƒƒƒƒƒƒƒƒƒƒƒƒƒƒƒ

COS 0.0037376 0.3277854 0.0669089 -0.4984229 0.7579941 0.9559

SIN 0.1502455 0.4503348 0.0919242 -0.9454252 1.0744130 0.1158

Intercept Intercept -3.1737587 0.8961014 0.1829159 -5.2929562 -1.8869171 <.0001

ƒƒƒƒƒƒƒƒƒƒƒƒƒƒƒƒƒƒƒƒƒƒƒƒƒƒƒƒƒƒƒƒƒƒƒƒƒƒƒƒƒƒƒƒƒƒƒƒƒƒƒƒƒƒƒƒƒƒƒƒƒƒƒƒƒƒƒƒƒƒƒƒƒƒƒƒƒƒƒƒƒƒƒƒƒƒƒƒƒƒƒƒƒƒƒƒƒƒƒƒƒƒƒƒƒƒƒƒƒƒƒƒƒƒ

hsa_miR_125a_5p 86

model test

The GLM Procedure

Number of observations 215

NOTE: Due to missing values, only 213 observations can be used in this analysis.

hsa_miR_125a_5p 87

model test

The GLM Procedure

Dependent Variable: variabel

Sum of

Source DF Squares Mean Square F Value Pr > F

Model 2 1.9358015 0.9679008 0.66 **0.5201**

Error 210 309.9364107 1.4758877

Corrected Total 212 311.8722122

R-Square Coeff Var Root MSE variabel Mean

0.006207 -38.49702 1.214861 -3.155728

Source DF Type I SS Mean Square F Value Pr > F

COS 1 0.01556397 0.01556397 0.01 0.9183

SIN 1 1.92023757 1.92023757 1.30 0.2553

Source DF Type III SS Mean Square F Value Pr > F

COS 1 0.01556398 0.01556398 0.01 0.9183

SIN 1 1.92023757 1.92023757 1.30 0.2553

Standard

Parameter Estimate Error t Value Pr > |t|

Intercept -3.156975700 0.08412339 -37.53 <.0001

COS 0.011557575 0.11254678 0.10 0.9183

SIN 0.142926761 0.12530336 1.14 0.2553

AMPLITUDE, TMAX AND TMIN FOR hsa_miR_125a_5p 88

Obs AMPL TMAX TMIN

1 0.30058 5.54 17.54

hsa_miR_127_3p 89

The MEANS Procedure

Variable Label Mean Std Dev Std Error Minimum Maximum Pr > |t|

ƒƒƒƒƒƒƒƒƒƒƒƒƒƒƒƒƒƒƒƒƒƒƒƒƒƒƒƒƒƒƒƒƒƒƒƒƒƒƒƒƒƒƒƒƒƒƒƒƒƒƒƒƒƒƒƒƒƒƒƒƒƒƒƒƒƒƒƒƒƒƒƒƒƒƒƒƒƒƒƒƒƒƒƒƒƒƒƒƒƒƒƒƒƒƒƒƒƒƒƒƒƒƒƒƒƒƒƒƒƒƒƒƒƒ

COS 0.0088658 0.4717982 0.0963054 -1.0228578 1.2295537 0.9274

SIN 0.0597191 0.3594714 0.0733768 -0.7923706 0.9084851 0.4241

Intercept Intercept -2.1791515 1.1808599 0.2410420 -5.7279932 -0.5601103 <.0001

ƒƒƒƒƒƒƒƒƒƒƒƒƒƒƒƒƒƒƒƒƒƒƒƒƒƒƒƒƒƒƒƒƒƒƒƒƒƒƒƒƒƒƒƒƒƒƒƒƒƒƒƒƒƒƒƒƒƒƒƒƒƒƒƒƒƒƒƒƒƒƒƒƒƒƒƒƒƒƒƒƒƒƒƒƒƒƒƒƒƒƒƒƒƒƒƒƒƒƒƒƒƒƒƒƒƒƒƒƒƒƒƒƒƒ

hsa_miR_127_3p 90

model test

The GLM Procedure

Number of observations 215

NOTE: Due to missing values, only 212 observations can be used in this analysis.

hsa_miR_127_3p 91

model test

The GLM Procedure

Dependent Variable: variabel

Sum of

Source DF Squares Mean Square F Value Pr > F

Model 2 1.6693819 0.8346910 0.50 **0.6061**

Error 209 347.6017936 1.6631665

Corrected Total 211 349.2711755

R-Square Coeff Var Root MSE variabel Mean

0.004780 -61.07933 1.289638 -2.111415

Source DF Type I SS Mean Square F Value Pr > F

COS 1 1.20401616 1.20401616 0.72 0.3958

SIN 1 0.46536576 0.46536576 0.28 0.5974

Source DF Type III SS Mean Square F Value Pr > F

COS 1 1.20581144 1.20581144 0.73 0.3955

SIN 1 0.46536576 0.46536576 0.28 0.5974

Standard

Parameter Estimate Error t Value Pr > |t|

Intercept -2.098308714 0.08983665 -23.36 <.0001

COS -0.102527752 0.12041195 -0.85 0.3955

SIN 0.070363023 0.13301945 0.53 0.5974

AMPLITUDE, TMAX AND TMIN FOR hsa_miR_127_3p 92

Obs AMPL TMAX TMIN

1 0.12075 5.26 17.26

hsa_miR_143_3p 93

The MEANS Procedure

Variable Label Mean Std Dev Std Error Minimum Maximum Pr > |t|

ƒƒƒƒƒƒƒƒƒƒƒƒƒƒƒƒƒƒƒƒƒƒƒƒƒƒƒƒƒƒƒƒƒƒƒƒƒƒƒƒƒƒƒƒƒƒƒƒƒƒƒƒƒƒƒƒƒƒƒƒƒƒƒƒƒƒƒƒƒƒƒƒƒƒƒƒƒƒƒƒƒƒƒƒƒƒƒƒƒƒƒƒƒƒƒƒƒƒƒƒƒƒƒƒƒƒƒƒƒƒƒƒƒƒ

COS -0.3366769 0.7704491 0.1572673 -1.7549956 1.6826006 0.0431

SIN 0.2859226 0.8146820 0.1662963 -1.1805546 2.0780761 0.0990

Intercept Intercept -4.7845123 1.2882735 0.2629677 -8.0347334 -2.6974555 <.0001

ƒƒƒƒƒƒƒƒƒƒƒƒƒƒƒƒƒƒƒƒƒƒƒƒƒƒƒƒƒƒƒƒƒƒƒƒƒƒƒƒƒƒƒƒƒƒƒƒƒƒƒƒƒƒƒƒƒƒƒƒƒƒƒƒƒƒƒƒƒƒƒƒƒƒƒƒƒƒƒƒƒƒƒƒƒƒƒƒƒƒƒƒƒƒƒƒƒƒƒƒƒƒƒƒƒƒƒƒƒƒƒƒƒƒ

hsa_miR_143_3p 94

model test

The GLM Procedure

Number of observations 215

NOTE: Due to missing values, only 212 observations can be used in this analysis.

hsa_miR_143_3p 95

model test

The GLM Procedure

Dependent Variable: variabel

Sum of

Source DF Squares Mean Square F Value Pr > F

Model 2 20.2162185 10.1081092 2.22 **0.1111**

Error 209 951.4076646 4.5521898

Corrected Total 211 971.6238830

R-Square Coeff Var Root MSE variabel Mean

0.020807 -44.40084 2.133586 -4.805283

Source DF Type I SS Mean Square F Value Pr > F

COS 1 11.74635197 11.74635197 2.58 0.1097

SIN 1 8.46986649 8.46986649 1.86 0.1740

Source DF Type III SS Mean Square F Value Pr > F

COS 1 11.62522359 11.62522359 2.55 0.1115

SIN 1 8.46986649 8.46986649 1.86 0.1740

Standard

Parameter Estimate Error t Value Pr > |t|

Intercept -4.769919705 0.14835451 -32.15 <.0001

COS -0.317096255 0.19842694 -1.60 0.1115

SIN 0.300991505 0.22066128 1.36 0.1740

AMPLITUDE, TMAX AND TMIN FOR hsa_miR_143_3p 96

Obs AMPL TMAX TMIN

1 0.88341 9.19 21.19

hsa_miR_124_3p 97

The MEANS Procedure

Variable Label Mean Std Dev Std Error Minimum Maximum Pr > |t|

ƒƒƒƒƒƒƒƒƒƒƒƒƒƒƒƒƒƒƒƒƒƒƒƒƒƒƒƒƒƒƒƒƒƒƒƒƒƒƒƒƒƒƒƒƒƒƒƒƒƒƒƒƒƒƒƒƒƒƒƒƒƒƒƒƒƒƒƒƒƒƒƒƒƒƒƒƒƒƒƒƒƒƒƒƒƒƒƒƒƒƒƒƒƒƒƒƒƒƒƒƒƒƒƒƒƒƒƒƒƒƒƒƒƒ

COS -0.0801768 1.3371957 0.2729539 -1.8204657 4.9271852 0.7716

SIN 0.5155189 1.2482818 0.2548045 -1.6250825 3.2058000 0.0548

Intercept Intercept -3.4803681 1.3137878 0.2681758 -7.0774649 -1.5857573 <.0001

ƒƒƒƒƒƒƒƒƒƒƒƒƒƒƒƒƒƒƒƒƒƒƒƒƒƒƒƒƒƒƒƒƒƒƒƒƒƒƒƒƒƒƒƒƒƒƒƒƒƒƒƒƒƒƒƒƒƒƒƒƒƒƒƒƒƒƒƒƒƒƒƒƒƒƒƒƒƒƒƒƒƒƒƒƒƒƒƒƒƒƒƒƒƒƒƒƒƒƒƒƒƒƒƒƒƒƒƒƒƒƒƒƒƒ

hsa_miR_124_3p 98

model test

The GLM Procedure

Number of observations 215

NOTE: Due to missing values, only 209 observations can be used in this analysis.

hsa_miR_124_3p 99

model test

The GLM Procedure

Dependent Variable: variabel

Sum of

Source DF Squares Mean Square F Value Pr > F

Model 2 26.237483 13.118741 1.62 **0.2008**

Error 206 1670.350827 8.108499

Corrected Total 208 1696.588310

R-Square Coeff Var Root MSE variabel Mean

0.015465 -82.16258 2.847543 -3.465742

Source DF Type I SS Mean Square F Value Pr > F

COS 1 1.23775296 1.23775296 0.15 0.6964

SIN 1 24.99972969 24.99972969 3.08 0.0806

Source DF Type III SS Mean Square F Value Pr > F

COS 1 1.31738784 1.31738784 0.16 0.6873

SIN 1 24.99972969 24.99972969 3.08 0.0806

Standard

Parameter Estimate Error t Value Pr > |t|

Intercept -3.447442264 0.20005755 -17.23 <.0001

COS -0.108466677 0.26909755 -0.40 0.6873

SIN 0.517136310 0.29451513 1.76 0.0806

AMPLITUDE, TMAX AND TMIN FOR hsa_miR_124_3p 100

Obs AMPL TMAX TMIN

1 1.04343 6.35 18.35

hsa_miR_133a_3p 101

The MEANS Procedure

Variable Label Mean Std Dev Std Error Minimum Maximum Pr > |t|

ƒƒƒƒƒƒƒƒƒƒƒƒƒƒƒƒƒƒƒƒƒƒƒƒƒƒƒƒƒƒƒƒƒƒƒƒƒƒƒƒƒƒƒƒƒƒƒƒƒƒƒƒƒƒƒƒƒƒƒƒƒƒƒƒƒƒƒƒƒƒƒƒƒƒƒƒƒƒƒƒƒƒƒƒƒƒƒƒƒƒƒƒƒƒƒƒƒƒƒƒƒƒƒƒƒƒƒƒƒƒƒƒƒƒ

COS 0.0041172 0.7982672 0.1629456 -1.2846756 1.8839835 0.9801

SIN 0.2861749 0.5714672 0.1166503 -0.7561916 1.3335065 0.0222

Intercept Intercept -4.9488115 1.3769180 0.2810622 -7.4822458 -2.4335202 <.0001

ƒƒƒƒƒƒƒƒƒƒƒƒƒƒƒƒƒƒƒƒƒƒƒƒƒƒƒƒƒƒƒƒƒƒƒƒƒƒƒƒƒƒƒƒƒƒƒƒƒƒƒƒƒƒƒƒƒƒƒƒƒƒƒƒƒƒƒƒƒƒƒƒƒƒƒƒƒƒƒƒƒƒƒƒƒƒƒƒƒƒƒƒƒƒƒƒƒƒƒƒƒƒƒƒƒƒƒƒƒƒƒƒƒƒ

hsa_miR_133a_3p 102

model test

The GLM Procedure

Number of observations 215

NOTE: Due to missing values, only 211 observations can be used in this analysis.

hsa_miR_133a_3p 103

model test

The GLM Procedure

Dependent Variable: variabel

Sum of

Source DF Squares Mean Square F Value Pr > F

Model 2 9.5725572 4.7862786 1.38 **0.2534**

Error 208 720.3734627 3.4633340

Corrected Total 210 729.9460199

R-Square Coeff Var Root MSE variabel Mean

0.013114 -37.89756 1.861003 -4.910616

Source DF Type I SS Mean Square F Value Pr > F

COS 1 0.43297528 0.43297528 0.13 0.7240

SIN 1 9.13958190 9.13958190 2.64 0.1058

Source DF Type III SS Mean Square F Value Pr > F

COS 1 0.41967515 0.41967515 0.12 0.7281

SIN 1 9.13958190 9.13958190 2.64 0.1058

Standard

Parameter Estimate Error t Value Pr > |t|

Intercept -4.901614407 0.12972850 -37.78 <.0001

COS -0.060513696 0.17383781 -0.35 0.7281

SIN 0.312664244 0.19246964 1.62 0.1058

AMPLITUDE, TMAX AND TMIN FOR hsa_miR_133a_3p 104

Obs AMPL TMAX TMIN

1 0.57241 5.57 17.57

hsa_miR_224_5p 105

The MEANS Procedure

Variable Label Mean Std Dev Std Error Minimum Maximum Pr > |t|

ƒƒƒƒƒƒƒƒƒƒƒƒƒƒƒƒƒƒƒƒƒƒƒƒƒƒƒƒƒƒƒƒƒƒƒƒƒƒƒƒƒƒƒƒƒƒƒƒƒƒƒƒƒƒƒƒƒƒƒƒƒƒƒƒƒƒƒƒƒƒƒƒƒƒƒƒƒƒƒƒƒƒƒƒƒƒƒƒƒƒƒƒƒƒƒƒƒƒƒƒƒƒƒƒƒƒƒƒƒƒƒƒƒƒ

COS -0.0409953 0.5895892 0.1203494 -1.3146136 0.9924780 0.7365

SIN 0.3800254 0.6056023 0.1236181 -0.5080940 1.5934709 0.0054

Intercept Intercept -5.6376728 1.1206411 0.2287499 -7.6485631 -3.2299674 <.0001

ƒƒƒƒƒƒƒƒƒƒƒƒƒƒƒƒƒƒƒƒƒƒƒƒƒƒƒƒƒƒƒƒƒƒƒƒƒƒƒƒƒƒƒƒƒƒƒƒƒƒƒƒƒƒƒƒƒƒƒƒƒƒƒƒƒƒƒƒƒƒƒƒƒƒƒƒƒƒƒƒƒƒƒƒƒƒƒƒƒƒƒƒƒƒƒƒƒƒƒƒƒƒƒƒƒƒƒƒƒƒƒƒƒƒ

hsa_miR_224_5p 106

model test

The GLM Procedure

Number of observations 215

NOTE: Due to missing values, only 205 observations can be used in this analysis.

hsa_miR_224_5p 107

model test

The GLM Procedure

Dependent Variable: variabel

Sum of

Source DF Squares Mean Square F Value Pr > F

Model 2 13.5107270 6.7553635 1.81 **0.1656**

Error 202 752.0699950 3.7231188

Corrected Total 204 765.5807220

R-Square Coeff Var Root MSE variabel Mean

0.017648 -34.36325 1.929538 -5.615122

Source DF Type I SS Mean Square F Value Pr > F

COS 1 0.03163710 0.03163710 0.01 0.9266

SIN 1 13.47908988 13.47908988 3.62 0.0585

Source DF Type III SS Mean Square F Value Pr > F

COS 1 0.01853591 0.01853591 0.00 0.9438

SIN 1 13.47908988 13.47908988 3.62 0.0585

Standard

Parameter Estimate Error t Value Pr > |t|

Intercept -5.616479052 0.13605283 -41.28 <.0001

COS -0.012817005 0.18164901 -0.07 0.9438

SIN 0.387044228 0.20341537 1.90 0.0585

AMPLITUDE, TMAX AND TMIN FOR hsa_miR_224_5p 108

Obs AMPL TMAX TMIN

1 0.76446 6.25 18.25

hsa_miR_10b_5p 109

The MEANS Procedure

Variable Label Mean Std Dev Std Error Minimum Maximum Pr > |t|

ƒƒƒƒƒƒƒƒƒƒƒƒƒƒƒƒƒƒƒƒƒƒƒƒƒƒƒƒƒƒƒƒƒƒƒƒƒƒƒƒƒƒƒƒƒƒƒƒƒƒƒƒƒƒƒƒƒƒƒƒƒƒƒƒƒƒƒƒƒƒƒƒƒƒƒƒƒƒƒƒƒƒƒƒƒƒƒƒƒƒƒƒƒƒƒƒƒƒƒƒƒƒƒƒƒƒƒƒƒƒƒƒƒƒ

COS -0.0351345 1.3884156 0.2834091 -3.5744044 1.9945667 0.9024

SIN 0.0667256 1.3291123 0.2713039 -2.2346031 3.0148148 0.8079

Intercept Intercept -6.3495587 0.7866830 0.1605810 -7.9621897 -4.7939038 <.0001

ƒƒƒƒƒƒƒƒƒƒƒƒƒƒƒƒƒƒƒƒƒƒƒƒƒƒƒƒƒƒƒƒƒƒƒƒƒƒƒƒƒƒƒƒƒƒƒƒƒƒƒƒƒƒƒƒƒƒƒƒƒƒƒƒƒƒƒƒƒƒƒƒƒƒƒƒƒƒƒƒƒƒƒƒƒƒƒƒƒƒƒƒƒƒƒƒƒƒƒƒƒƒƒƒƒƒƒƒƒƒƒƒƒƒ

hsa_miR_10b_5p 110

model test

The GLM Procedure

Number of observations 215

NOTE: Due to missing values, only 204 observations can be used in this analysis.

hsa_miR_10b_5p 111

model test

The GLM Procedure

Dependent Variable: variabel

Sum of

Source DF Squares Mean Square F Value Pr > F

Model 2 0.111528 0.055764 0.01 **0.9912**

Error 201 1269.853426 6.317679

Corrected Total 203 1269.964954

R-Square Coeff Var Root MSE variabel Mean

0.000088 -39.41562 2.513499 -6.376912

Source DF Type I SS Mean Square F Value Pr > F

COS 1 0.03098786 0.03098786 0.00 0.9442

SIN 1 0.08054008 0.08054008 0.01 0.9102

Source DF Type III SS Mean Square F Value Pr > F

COS 1 0.03217930 0.03217930 0.01 0.9432

SIN 1 0.08054008 0.08054008 0.01 0.9102

Standard

Parameter Estimate Error t Value Pr > |t|

Intercept -6.375337742 0.17816503 -35.78 <.0001

COS -0.016970900 0.23779094 -0.07 0.9432

SIN 0.030006284 0.26575714 0.11 0.9102

AMPLITUDE, TMAX AND TMIN FOR hsa_miR_10b_5p 112

Obs AMPL TMAX TMIN

1 0.15082 7.51 19.51

hsa_miR_99a_5p 113

The MEANS Procedure

Variable Label Mean Std Dev Std Error Minimum Maximum Pr > |t|

ƒƒƒƒƒƒƒƒƒƒƒƒƒƒƒƒƒƒƒƒƒƒƒƒƒƒƒƒƒƒƒƒƒƒƒƒƒƒƒƒƒƒƒƒƒƒƒƒƒƒƒƒƒƒƒƒƒƒƒƒƒƒƒƒƒƒƒƒƒƒƒƒƒƒƒƒƒƒƒƒƒƒƒƒƒƒƒƒƒƒƒƒƒƒƒƒƒƒƒƒƒƒƒƒƒƒƒƒƒƒƒƒƒƒ

COS -0.0050945 0.5605831 0.1144285 -1.0716582 1.1501462 0.9649

SIN 0.1860930 0.7091656 0.1447578 -1.4456386 1.8083395 0.2114

Intercept Intercept -5.3716035 0.5918316 0.1208071 -6.5621116 -4.1507858 <.0001

ƒƒƒƒƒƒƒƒƒƒƒƒƒƒƒƒƒƒƒƒƒƒƒƒƒƒƒƒƒƒƒƒƒƒƒƒƒƒƒƒƒƒƒƒƒƒƒƒƒƒƒƒƒƒƒƒƒƒƒƒƒƒƒƒƒƒƒƒƒƒƒƒƒƒƒƒƒƒƒƒƒƒƒƒƒƒƒƒƒƒƒƒƒƒƒƒƒƒƒƒƒƒƒƒƒƒƒƒƒƒƒƒƒƒ

hsa_miR_99a_5p 114

model test

The GLM Procedure

Number of observations 215

NOTE: Due to missing values, only 205 observations can be used in this analysis.

hsa_miR_99a_5p 115

model test

The GLM Procedure

Dependent Variable: variabel

Sum of

Source DF Squares Mean Square F Value Pr > F

Model 2 3.5049183 1.7524591 0.72 **0.4871**

Error 202 490.4257178 2.4278501

Corrected Total 204 493.9306361

R-Square Coeff Var Root MSE variabel Mean

0.007096 -29.13344 1.558156 -5.348341

Source DF Type I SS Mean Square F Value Pr > F

COS 1 0.01232467 0.01232467 0.01 0.9433

SIN 1 3.49259362 3.49259362 1.44 0.2318

Source DF Type III SS Mean Square F Value Pr > F

COS 1 0.01209606 0.01209606 0.00 0.9438

SIN 1 3.49259362 3.49259362 1.44 0.2318

Standard

Parameter Estimate Error t Value Pr > |t|

Intercept -5.348781473 0.10973976 -48.74 <.0001

COS 0.010432991 0.14780794 0.07 0.9438

SIN 0.194842696 0.16245049 1.20 0.2318

AMPLITUDE, TMAX AND TMIN FOR hsa_miR_99a_5p 116

Obs AMPL TMAX TMIN

1 0.37233 6.06 18.06

hsa_miR_125b_5p 117

The MEANS Procedure

Variable Label Mean Std Dev Std Error Minimum Maximum Pr > |t|

ƒƒƒƒƒƒƒƒƒƒƒƒƒƒƒƒƒƒƒƒƒƒƒƒƒƒƒƒƒƒƒƒƒƒƒƒƒƒƒƒƒƒƒƒƒƒƒƒƒƒƒƒƒƒƒƒƒƒƒƒƒƒƒƒƒƒƒƒƒƒƒƒƒƒƒƒƒƒƒƒƒƒƒƒƒƒƒƒƒƒƒƒƒƒƒƒƒƒƒƒƒƒƒƒƒƒƒƒƒƒƒƒƒƒ

COS -0.0401513 0.4554902 0.0929765 -0.8568376 0.9858442 0.6699

SIN -0.000593140 0.6070599 0.1239156 -0.8655500 1.3968213 0.9962

Intercept Intercept -4.7457314 0.3929526 0.0802111 -5.6590370 -4.0379872 <.0001

ƒƒƒƒƒƒƒƒƒƒƒƒƒƒƒƒƒƒƒƒƒƒƒƒƒƒƒƒƒƒƒƒƒƒƒƒƒƒƒƒƒƒƒƒƒƒƒƒƒƒƒƒƒƒƒƒƒƒƒƒƒƒƒƒƒƒƒƒƒƒƒƒƒƒƒƒƒƒƒƒƒƒƒƒƒƒƒƒƒƒƒƒƒƒƒƒƒƒƒƒƒƒƒƒƒƒƒƒƒƒƒƒƒƒ

hsa_miR_125b_5p 118

model test

The GLM Procedure

Number of observations 215

NOTE: Due to missing values, only 201 observations can be used in this analysis.

hsa_miR_125b_5p 119

model test

The GLM Procedure

Dependent Variable: variabel

Sum of

Source DF Squares Mean Square F Value Pr > F

Model 2 0.5254042 0.2627021 0.23 **0.7925**

Error 198 223.3727759 1.1281453

Corrected Total 200 223.8981801

R-Square Coeff Var Root MSE variabel Mean

0.002347 -22.28828 1.062142 -4.765473

Source DF Type I SS Mean Square F Value Pr > F

COS 1 0.19533178 0.19533178 0.17 0.6778

SIN 1 0.33007241 0.33007241 0.29 0.5892

Source DF Type III SS Mean Square F Value Pr > F

COS 1 0.18975469 0.18975469 0.17 0.6822

SIN 1 0.33007241 0.33007241 0.29 0.5892

Standard

Parameter Estimate Error t Value Pr > |t|

Intercept -4.763387920 0.07562069 -62.99 <.0001

COS -0.041228429 0.10052708 -0.41 0.6822

SIN 0.061487957 0.11367579 0.54 0.5892

AMPLITUDE, TMAX AND TMIN FOR hsa_miR_125b_5p 120

Obs AMPL TMAX TMIN

1 0.080311 12.03 0.03

hsa_miR_141_3p 121

The MEANS Procedure

Variable Label Mean Std Dev Std Error Minimum Maximum Pr > |t|

ƒƒƒƒƒƒƒƒƒƒƒƒƒƒƒƒƒƒƒƒƒƒƒƒƒƒƒƒƒƒƒƒƒƒƒƒƒƒƒƒƒƒƒƒƒƒƒƒƒƒƒƒƒƒƒƒƒƒƒƒƒƒƒƒƒƒƒƒƒƒƒƒƒƒƒƒƒƒƒƒƒƒƒƒƒƒƒƒƒƒƒƒƒƒƒƒƒƒƒƒƒƒƒƒƒƒƒƒƒƒƒƒƒƒ

COS -0.2004873 1.6138867 0.3294332 -6.6435642 2.1891625 0.5488

SIN -0.0777261 1.7796405 0.3632676 -6.6434327 2.9533110 0.8325

Intercept Intercept 0.4562728 2.2584480 0.4610038 -3.1769587 7.4726476 0.3326

ƒƒƒƒƒƒƒƒƒƒƒƒƒƒƒƒƒƒƒƒƒƒƒƒƒƒƒƒƒƒƒƒƒƒƒƒƒƒƒƒƒƒƒƒƒƒƒƒƒƒƒƒƒƒƒƒƒƒƒƒƒƒƒƒƒƒƒƒƒƒƒƒƒƒƒƒƒƒƒƒƒƒƒƒƒƒƒƒƒƒƒƒƒƒƒƒƒƒƒƒƒƒƒƒƒƒƒƒƒƒƒƒƒƒ

hsa_miR_141_3p 122

model test

The GLM Procedure

Number of observations 215

NOTE: Due to missing values, only 198 observations can be used in this analysis.

hsa_miR_141_3p 123

model test

The GLM Procedure

Dependent Variable: variabel

Sum of

Source DF Squares Mean Square F Value Pr > F

Model 2 0.690192 0.345096 0.03 **0.9676**

Error 195 2040.451016 10.463851

Corrected Total 197 2041.141209

R-Square Coeff Var Root MSE variabel Mean

0.000338 1284.315 3.234788 0.251869

Source DF Type I SS Mean Square F Value Pr > F

COS 1 0.59106022 0.59106022 0.06 0.8124

SIN 1 0.09913208 0.09913208 0.01 0.9226

Source DF Type III SS Mean Square F Value Pr > F

COS 1 0.58636132 0.58636132 0.06 0.8131

SIN 1 0.09913208 0.09913208 0.01 0.9226

Standard

Parameter Estimate Error t Value Pr > |t|

Intercept 0.2428581978 0.23260297 1.04 0.2977

COS 0.0736588870 0.31116323 0.24 0.8131

SIN 0.0336862081 0.34609139 0.10 0.9226

AMPLITUDE, TMAX AND TMIN FOR hsa_miR_141_3p 124

Obs AMPL TMAX TMIN

1 0.43005 13.25 1.25

hsa_miR_579_3p 125

The MEANS Procedure

Variable Label Mean Std Dev Std Error Minimum Maximum Pr > |t|

ƒƒƒƒƒƒƒƒƒƒƒƒƒƒƒƒƒƒƒƒƒƒƒƒƒƒƒƒƒƒƒƒƒƒƒƒƒƒƒƒƒƒƒƒƒƒƒƒƒƒƒƒƒƒƒƒƒƒƒƒƒƒƒƒƒƒƒƒƒƒƒƒƒƒƒƒƒƒƒƒƒƒƒƒƒƒƒƒƒƒƒƒƒƒƒƒƒƒƒƒƒƒƒƒƒƒƒƒƒƒƒƒƒƒ

COS 0.1243896 0.6871027 0.1402543 -1.1395183 1.5532992 0.3843

SIN 0.1038193 0.7832970 0.1598898 -2.0143167 1.5428195 0.5226

Intercept Intercept -5.7710745 0.5380376 0.1098265 -6.9588284 -4.7839715 <.0001

ƒƒƒƒƒƒƒƒƒƒƒƒƒƒƒƒƒƒƒƒƒƒƒƒƒƒƒƒƒƒƒƒƒƒƒƒƒƒƒƒƒƒƒƒƒƒƒƒƒƒƒƒƒƒƒƒƒƒƒƒƒƒƒƒƒƒƒƒƒƒƒƒƒƒƒƒƒƒƒƒƒƒƒƒƒƒƒƒƒƒƒƒƒƒƒƒƒƒƒƒƒƒƒƒƒƒƒƒƒƒƒƒƒƒ

hsa_miR_579_3p 126

model test

The GLM Procedure

Number of observations 215

NOTE: Due to missing values, only 190 observations can be used in this analysis.

hsa_miR_579_3p 127

model test

The GLM Procedure

Dependent Variable: variabel

Sum of

Source DF Squares Mean Square F Value Pr > F

Model 2 3.1543805 1.5771902 0.72 **0.4893**

Error 187 411.0181058 2.1979578

Corrected Total 189 414.1724863

R-Square Coeff Var Root MSE variabel Mean

0.007616 -25.75851 1.482551 -5.755579

Source DF Type I SS Mean Square F Value Pr > F

COS 1 1.82459457 1.82459457 0.83 0.3634

SIN 1 1.32978591 1.32978591 0.61 0.4377

Source DF Type III SS Mean Square F Value Pr > F

COS 1 1.85732187 1.85732187 0.85 0.3591

SIN 1 1.32978591 1.32978591 0.61 0.4377

Standard

Parameter Estimate Error t Value Pr > |t|

Intercept -5.769176601 0.10858487 -53.13 <.0001

COS 0.133018549 0.14470325 0.92 0.3591

SIN 0.126583258 0.16274029 0.78 0.4377

AMPLITUDE, TMAX AND TMIN FOR hsa_miR_579_3p 128

Obs AMPL TMAX TMIN

1 0.32404 2.39 14.39

hsa_miR_1_3p 129

The MEANS Procedure

Variable Label Mean Std Dev Std Error Minimum Maximum Pr > |t|

ƒƒƒƒƒƒƒƒƒƒƒƒƒƒƒƒƒƒƒƒƒƒƒƒƒƒƒƒƒƒƒƒƒƒƒƒƒƒƒƒƒƒƒƒƒƒƒƒƒƒƒƒƒƒƒƒƒƒƒƒƒƒƒƒƒƒƒƒƒƒƒƒƒƒƒƒƒƒƒƒƒƒƒƒƒƒƒƒƒƒƒƒƒƒƒƒƒƒƒƒƒƒƒƒƒƒƒƒƒƒƒƒƒƒ

COS 0.0555681 1.4286817 0.2916284 -4.4264885 2.5054788 0.8506

SIN -0.0746360 1.7500873 0.3572351 -5.2229203 2.4033595 0.8363

Intercept Intercept -9.0181552 1.3307297 0.2716341 -12.7299999 -6.1987731 <.0001

ƒƒƒƒƒƒƒƒƒƒƒƒƒƒƒƒƒƒƒƒƒƒƒƒƒƒƒƒƒƒƒƒƒƒƒƒƒƒƒƒƒƒƒƒƒƒƒƒƒƒƒƒƒƒƒƒƒƒƒƒƒƒƒƒƒƒƒƒƒƒƒƒƒƒƒƒƒƒƒƒƒƒƒƒƒƒƒƒƒƒƒƒƒƒƒƒƒƒƒƒƒƒƒƒƒƒƒƒƒƒƒƒƒƒ

hsa_miR_1_3p 130

model test

The GLM Procedure

Number of observations 215

NOTE: Due to missing values, only 158 observations can be used in this analysis.

hsa_miR_1_3p 131

model test

The GLM Procedure

Dependent Variable: variabel

Sum of

Source DF Squares Mean Square F Value Pr > F

Model 2 1.6777054 0.8388527 0.18 **0.8319**

Error 155 705.6108421 4.5523280

Corrected Total 157 707.2885475

R-Square Coeff Var Root MSE variabel Mean

0.002372 -24.20406 2.133619 -8.815127

Source DF Type I SS Mean Square F Value Pr > F

COS 1 0.46219964 0.46219964 0.10 0.7504

SIN 1 1.21550578 1.21550578 0.27 0.6061

Source DF Type III SS Mean Square F Value Pr > F

COS 1 0.39623459 0.39623459 0.09 0.7684

SIN 1 1.21550578 1.21550578 0.27 0.6061

Standard

Parameter Estimate Error t Value Pr > |t|

Intercept -8.814501840 0.17224826 -51.17 <.0001

COS 0.068052298 0.23066589 0.30 0.7684

SIN -0.131959785 0.25537587 -0.52 0.6061

AMPLITUDE, TMAX AND TMIN FOR hsa_miR_1_3p 132

Obs AMPL TMAX TMIN

1 0.18610 20.27 8.27

hsa_miR_107 133

The MEANS Procedure

Variable Label Mean Std Dev Std Error Minimum Maximum Pr > |t|

ƒƒƒƒƒƒƒƒƒƒƒƒƒƒƒƒƒƒƒƒƒƒƒƒƒƒƒƒƒƒƒƒƒƒƒƒƒƒƒƒƒƒƒƒƒƒƒƒƒƒƒƒƒƒƒƒƒƒƒƒƒƒƒƒƒƒƒƒƒƒƒƒƒƒƒƒƒƒƒƒƒƒƒƒƒƒƒƒƒƒƒƒƒƒƒƒƒƒƒƒƒƒƒƒƒƒƒƒƒƒƒƒƒƒ

COS 0.0507784 0.8551443 0.1745556 -0.9720853 2.9245067 0.7737

SIN -0.2701670 1.0392655 0.2121392 -3.7109229 1.6187662 0.2155

Intercept Intercept -5.6715234 0.5272617 0.1076268 -6.8239145 -4.7995064 <.0001

ƒƒƒƒƒƒƒƒƒƒƒƒƒƒƒƒƒƒƒƒƒƒƒƒƒƒƒƒƒƒƒƒƒƒƒƒƒƒƒƒƒƒƒƒƒƒƒƒƒƒƒƒƒƒƒƒƒƒƒƒƒƒƒƒƒƒƒƒƒƒƒƒƒƒƒƒƒƒƒƒƒƒƒƒƒƒƒƒƒƒƒƒƒƒƒƒƒƒƒƒƒƒƒƒƒƒƒƒƒƒƒƒƒƒ

hsa_miR_107 134

model test

The GLM Procedure

Number of observations 215

NOTE: Due to missing values, only 149 observations can be used in this analysis.

hsa_miR_107 135

model test

The GLM Procedure

Dependent Variable: variabel

Sum of

Source DF Squares Mean Square F Value Pr > F

Model 2 1.3975943 0.6987972 0.37 **0.6906**

Error 146 274.8955815 1.8828464

Corrected Total 148 276.2931758

R-Square Coeff Var Root MSE variabel Mean

0.005058 -24.46109 1.372169 -5.609597

Source DF Type I SS Mean Square F Value Pr > F

COS 1 0.11829809 0.11829809 0.06 0.8024

SIN 1 1.27929621 1.27929621 0.68 0.4111

Source DF Type III SS Mean Square F Value Pr > F

COS 1 0.09440585 0.09440585 0.05 0.8231

SIN 1 1.27929621 1.27929621 0.68 0.4111

Standard

Parameter Estimate Error t Value Pr > |t|

Intercept -5.608439093 0.11326605 -49.52 <.0001

COS 0.033560227 0.14987627 0.22 0.8231

SIN -0.141589882 0.17177269 -0.82 0.4111

AMPLITUDE, TMAX AND TMIN FOR hsa_miR_107 136

Obs AMPL TMAX TMIN

1 0.54980 18.43 6.43

hsa_miR_183_5p 137

The MEANS Procedure

Variable Label Mean Std Dev Std Error Minimum Maximum Pr > |t|

ƒƒƒƒƒƒƒƒƒƒƒƒƒƒƒƒƒƒƒƒƒƒƒƒƒƒƒƒƒƒƒƒƒƒƒƒƒƒƒƒƒƒƒƒƒƒƒƒƒƒƒƒƒƒƒƒƒƒƒƒƒƒƒƒƒƒƒƒƒƒƒƒƒƒƒƒƒƒƒƒƒƒƒƒƒƒƒƒƒƒƒƒƒƒƒƒƒƒƒƒƒƒƒƒƒƒƒƒƒƒƒƒƒƒ

COS 0.4055282 1.5050660 0.3072203 -1.4770038 5.4456696 0.1998

SIN -0.1303928 1.8203343 0.3715742 -5.7689239 3.3571456 0.7288

Intercept Intercept -9.1843516 1.6539119 0.3376033 -13.9256698 -6.2537157 <.0001

ƒƒƒƒƒƒƒƒƒƒƒƒƒƒƒƒƒƒƒƒƒƒƒƒƒƒƒƒƒƒƒƒƒƒƒƒƒƒƒƒƒƒƒƒƒƒƒƒƒƒƒƒƒƒƒƒƒƒƒƒƒƒƒƒƒƒƒƒƒƒƒƒƒƒƒƒƒƒƒƒƒƒƒƒƒƒƒƒƒƒƒƒƒƒƒƒƒƒƒƒƒƒƒƒƒƒƒƒƒƒƒƒƒƒ

hsa_miR_183_5p 138

model test

The GLM Procedure

Number of observations 215

NOTE: Due to missing values, only 130 observations can be used in this analysis.

hsa_miR_183_5p 139

model test

The GLM Procedure

Dependent Variable: variabel

Sum of

Source DF Squares Mean Square F Value Pr > F

Model 2 3.5303185 1.7651593 0.38 **0.6861**

Error 127 593.3227807 4.6718329

Corrected Total 129 596.8530992

R-Square Coeff Var Root MSE variabel Mean

0.005915 -23.32640 2.161442 -9.266077

Source DF Type I SS Mean Square F Value Pr > F

COS 1 3.40466556 3.40466556 0.73 0.3949

SIN 1 0.12565294 0.12565294 0.03 0.8700

Source DF Type III SS Mean Square F Value Pr > F

COS 1 3.43646324 3.43646324 0.74 0.3927

SIN 1 0.12565294 0.12565294 0.03 0.8700

Standard

Parameter Estimate Error t Value Pr > |t|

Intercept -9.296520036 0.19337453 -48.08 <.0001

COS 0.223889535 0.26104874 0.86 0.3927

SIN 0.046242633 0.28196803 0.16 0.8700

AMPLITUDE, TMAX AND TMIN FOR hsa_miR_183_5p 140

Obs AMPL TMAX TMIN

1 0.85195 22.49 10.49

hsa_miR_182_5p 141

The MEANS Procedure

Variable Label Mean Std Dev Std Error Minimum Maximum Pr > |t|

ƒƒƒƒƒƒƒƒƒƒƒƒƒƒƒƒƒƒƒƒƒƒƒƒƒƒƒƒƒƒƒƒƒƒƒƒƒƒƒƒƒƒƒƒƒƒƒƒƒƒƒƒƒƒƒƒƒƒƒƒƒƒƒƒƒƒƒƒƒƒƒƒƒƒƒƒƒƒƒƒƒƒƒƒƒƒƒƒƒƒƒƒƒƒƒƒƒƒƒƒƒƒƒƒƒƒƒƒƒƒƒƒƒƒ

COS 0.1367791 1.5120978 0.3086557 -2.8591275 2.5650001 0.6618

SIN 0.0570965 1.6094477 0.3285271 -2.9091370 5.5569908 0.8635

Intercept Intercept -18.1155894 0.9563514 0.1952144 -19.9477697 -16.0900000 <.0001

ƒƒƒƒƒƒƒƒƒƒƒƒƒƒƒƒƒƒƒƒƒƒƒƒƒƒƒƒƒƒƒƒƒƒƒƒƒƒƒƒƒƒƒƒƒƒƒƒƒƒƒƒƒƒƒƒƒƒƒƒƒƒƒƒƒƒƒƒƒƒƒƒƒƒƒƒƒƒƒƒƒƒƒƒƒƒƒƒƒƒƒƒƒƒƒƒƒƒƒƒƒƒƒƒƒƒƒƒƒƒƒƒƒƒ

hsa_miR_182_5p 142

model test

The GLM Procedure

Number of observations 215

NOTE: Due to missing values, only 115 observations can be used in this analysis.

hsa_miR_182_5p 143

model test

The GLM Procedure

Dependent Variable: variabel

Sum of

Source DF Squares Mean Square F Value Pr > F

Model 2 0.7957932 0.3978966 0.10 **0.9025**

Error 112 433.9848555 3.8748648

Corrected Total 114 434.7806487

R-Square Coeff Var Root MSE variabel Mean

0.001830 -10.87880 1.968468 -18.09452

Source DF Type I SS Mean Square F Value Pr > F

COS 1 0.56858857 0.56858857 0.15 0.7024

SIN 1 0.22720465 0.22720465 0.06 0.8091

Source DF Type III SS Mean Square F Value Pr > F

COS 1 0.47315785 0.47315785 0.12 0.7274

SIN 1 0.22720465 0.22720465 0.06 0.8091

Standard

Parameter Estimate Error t Value Pr > |t|

Intercept -18.09724580 0.18923538 -95.63 <.0001

COS 0.08812921 0.25220002 0.35 0.7274

SIN -0.06792482 0.28050988 -0.24 0.8091

AMPLITUDE, TMAX AND TMIN FOR hsa_miR_182_5p 144

Obs AMPL TMAX TMIN

1 0.29644 1.31 13.31
