# Supplementary material for: Diurnal Variations of Human Circulating Cell-Free Micro-RNA
Source: PLoS One. 2016 Aug 5;11(8):e0160577. doi: 10.1371/journal.pone.0160577 (PMC4975411; doi:10.1371/journal.pone.0160577)
Supplement: S2 File — The p-values are indicated in red. (DOCX) [file pone.0160577.s005.docx]

**hsa_let_7b_5p** 1

The MEANS Procedure

Variable Label Mean Std Dev Std Error Minimum Maximum Pr > |t|

ƒƒƒƒƒƒƒƒƒƒƒƒƒƒƒƒƒƒƒƒƒƒƒƒƒƒƒƒƒƒƒƒƒƒƒƒƒƒƒƒƒƒƒƒƒƒƒƒƒƒƒƒƒƒƒƒƒƒƒƒƒƒƒƒƒƒƒƒƒƒƒƒƒƒƒƒƒƒƒƒƒƒƒƒƒƒƒƒƒƒƒƒƒƒƒƒƒƒƒƒƒƒƒƒƒƒƒƒƒƒƒƒƒƒ

COS 0.0077023 0.2542529 0.0518992 -0.5587497 0.4169925 0.8833

SIN 0.0317081 0.3185398 0.0650217 -0.3858973 0.9016815 0.6304

Intercept Intercept -6.5249299 0.9616911 0.1963044 -8.7441469 -5.2095482 <.0001

ƒƒƒƒƒƒƒƒƒƒƒƒƒƒƒƒƒƒƒƒƒƒƒƒƒƒƒƒƒƒƒƒƒƒƒƒƒƒƒƒƒƒƒƒƒƒƒƒƒƒƒƒƒƒƒƒƒƒƒƒƒƒƒƒƒƒƒƒƒƒƒƒƒƒƒƒƒƒƒƒƒƒƒƒƒƒƒƒƒƒƒƒƒƒƒƒƒƒƒƒƒƒƒƒƒƒƒƒƒƒƒƒƒƒ

hsa_let_7b_5p

model test

The GLM Procedure

Number of observations 216

hsa_let_7b_5p 3

model test

The GLM Procedure

Dependent Variable: variabel

Sum of

Source DF Squares Mean Square F Value Pr > F

Model 2 0.1034796 0.0517398 0.04 **0.9566**

Error 213 248.4001352 1.1661978

Corrected Total 215 248.5036148

R-Square Coeff Var Root MSE variabel Mean

0.000416 -16.55264 1.079906 -6.524074

Source DF Type I SS Mean Square F Value Pr > F

COS 1 0.00696085 0.00696085 0.01 0.9385

SIN 1 0.09651874 0.09651874 0.08 0.7739

Source DF Type III SS Mean Square F Value Pr > F

COS 1 0.00696085 0.00696085 0.01 0.9385

SIN 1 0.09651874 0.09651874 0.08 0.7739

Standard

Parameter Estimate Error t Value Pr > |t|

Intercept -6.524929885 0.07430861 -87.81 <.0001

COS 0.007702303 0.09969547 0.08 0.9385

SIN 0.031708098 0.11021748 0.29 0.7739

AMPLITUDE, TMAX AND TMIN FOR hsa_let_7b_5p 4

Obs AMPL TMAX TMIN

1 0.065260 5.05 17.05

**hsa_miR_7_5p** 5

The MEANS Procedure

Variable Label Mean Std Dev Std Error Minimum Maximum Pr > |t|

ƒƒƒƒƒƒƒƒƒƒƒƒƒƒƒƒƒƒƒƒƒƒƒƒƒƒƒƒƒƒƒƒƒƒƒƒƒƒƒƒƒƒƒƒƒƒƒƒƒƒƒƒƒƒƒƒƒƒƒƒƒƒƒƒƒƒƒƒƒƒƒƒƒƒƒƒƒƒƒƒƒƒƒƒƒƒƒƒƒƒƒƒƒƒƒƒƒƒƒƒƒƒƒƒƒƒƒƒƒƒƒƒƒƒ

COS -0.1141932 2.2304640 0.4552916 -5.7173775 4.0076747 0.8042

SIN -0.1425035 3.1862495 0.6503904 -6.6131366 5.4444643 0.8285

Intercept Intercept 0.3510215 3.1637819 0.6458043 -7.8089769 5.6666394 0.5920

ƒƒƒƒƒƒƒƒƒƒƒƒƒƒƒƒƒƒƒƒƒƒƒƒƒƒƒƒƒƒƒƒƒƒƒƒƒƒƒƒƒƒƒƒƒƒƒƒƒƒƒƒƒƒƒƒƒƒƒƒƒƒƒƒƒƒƒƒƒƒƒƒƒƒƒƒƒƒƒƒƒƒƒƒƒƒƒƒƒƒƒƒƒƒƒƒƒƒƒƒƒƒƒƒƒƒƒƒƒƒƒƒƒƒ

hsa_miR_7_5p 6

model test

The GLM Procedure

Number of observations 216

hsa_miR_7_5p 7

model test

The GLM Procedure

Dependent Variable: variabel

Sum of

Source DF Squares Mean Square F Value Pr > F

Model 2 3.47953 1.73977 0.04 **0.9642**

Error 213 10164.38487 47.72012

Corrected Total 215 10167.86440

R-Square Coeff Var Root MSE variabel Mean

0.000342 2041.766 6.907975 0.338333

Source DF Type I SS Mean Square F Value Pr > F

COS 1 1.53003633 1.53003633 0.03 0.8581

SIN 1 1.94949610 1.94949610 0.04 0.8400

Source DF Type III SS Mean Square F Value Pr > F

COS 1 1.53003637 1.53003637 0.03 0.8581

SIN 1 1.94949610 1.94949610 0.04 0.8400

Standard

Parameter Estimate Error t Value Pr > |t|

Intercept 0.3510214622 0.47533937 0.74 0.4610

COS -.1141931818 0.63773470 -0.18 0.8581

SIN -.1425035114 0.70504223 -0.20 0.8400

AMPLITUDE, TMAX AND TMIN FOR hsa_miR_7_5p 8

Obs AMPL TMAX TMIN

1 0.36523 15.25 3.25

**hsa_miR_15b_5p** 9

The MEANS Procedure

Variable Label Mean Std Dev Std Error Minimum Maximum Pr > |t|

ƒƒƒƒƒƒƒƒƒƒƒƒƒƒƒƒƒƒƒƒƒƒƒƒƒƒƒƒƒƒƒƒƒƒƒƒƒƒƒƒƒƒƒƒƒƒƒƒƒƒƒƒƒƒƒƒƒƒƒƒƒƒƒƒƒƒƒƒƒƒƒƒƒƒƒƒƒƒƒƒƒƒƒƒƒƒƒƒƒƒƒƒƒƒƒƒƒƒƒƒƒƒƒƒƒƒƒƒƒƒƒƒƒƒ

COS -0.0323437 0.2989547 0.0610239 -0.5278509 0.6357960 0.6012

SIN 0.2903952 0.3013611 0.0615151 -0.2072360 0.9678301 <.0001

Intercept Intercept -6.2927951 0.8766233 0.1789400 -7.8974150 -4.5027131 <.0001

ƒƒƒƒƒƒƒƒƒƒƒƒƒƒƒƒƒƒƒƒƒƒƒƒƒƒƒƒƒƒƒƒƒƒƒƒƒƒƒƒƒƒƒƒƒƒƒƒƒƒƒƒƒƒƒƒƒƒƒƒƒƒƒƒƒƒƒƒƒƒƒƒƒƒƒƒƒƒƒƒƒƒƒƒƒƒƒƒƒƒƒƒƒƒƒƒƒƒƒƒƒƒƒƒƒƒƒƒƒƒƒƒƒƒ

hsa_miR_15b_5p 10

model test

The GLM Procedure

Number of observations 216

hsa_miR_15b_5p 11

model test

The GLM Procedure

Dependent Variable: variabel

Sum of

Source DF Squares Mean Square F Value Pr > F

Model 2 8.2183665 4.1091832 3.34 **0.0371**

Error 213 261.6802169 1.2285456

Corrected Total 215 269.8985833

R-Square Coeff Var Root MSE variabel Mean

0.030450 -17.60371 1.108398 -6.296389

Source DF Type I SS Mean Square F Value Pr > F

COS 1 0.12274397 0.12274397 0.10 0.7522

SIN 1 8.09562252 8.09562252 6.59 0.0109

Source DF Type III SS Mean Square F Value Pr > F

COS 1 0.12274394 0.12274394 0.10 0.7522

SIN 1 8.09562252 8.09562252 6.59 0.0109

Standard

Parameter Estimate Error t Value Pr > |t|

Intercept -6.292795146 0.07626911 -82.51 <.0001

COS -0.032343673 0.10232575 -0.32 0.7522

SIN 0.290395248 0.11312537 2.57 0.0109

AMPLITUDE, TMAX AND TMIN FOR hsa_miR_15b_5p 12

Obs AMPL TMAX TMIN

1 0.58438 6.25 18.25

**hsa_miR_17_5p** 13

The MEANS Procedure

Variable Label Mean Std Dev Std Error Minimum Maximum Pr > |t|

ƒƒƒƒƒƒƒƒƒƒƒƒƒƒƒƒƒƒƒƒƒƒƒƒƒƒƒƒƒƒƒƒƒƒƒƒƒƒƒƒƒƒƒƒƒƒƒƒƒƒƒƒƒƒƒƒƒƒƒƒƒƒƒƒƒƒƒƒƒƒƒƒƒƒƒƒƒƒƒƒƒƒƒƒƒƒƒƒƒƒƒƒƒƒƒƒƒƒƒƒƒƒƒƒƒƒƒƒƒƒƒƒƒƒ

COS -0.0439160 0.2094022 0.0427441 -0.5170937 0.2672158 0.3149

SIN 0.1194018 0.2032522 0.0414887 -0.3129074 0.4940559 0.0085

Intercept Intercept -2.5980371 0.7068647 0.1442881 -3.6670132 -1.4749153 <.0001

ƒƒƒƒƒƒƒƒƒƒƒƒƒƒƒƒƒƒƒƒƒƒƒƒƒƒƒƒƒƒƒƒƒƒƒƒƒƒƒƒƒƒƒƒƒƒƒƒƒƒƒƒƒƒƒƒƒƒƒƒƒƒƒƒƒƒƒƒƒƒƒƒƒƒƒƒƒƒƒƒƒƒƒƒƒƒƒƒƒƒƒƒƒƒƒƒƒƒƒƒƒƒƒƒƒƒƒƒƒƒƒƒƒƒ

hsa_miR_17_5p 14

model test

The GLM Procedure

Number of observations 216

hsa_miR_17_5p 15

model test

The GLM Procedure

Dependent Variable: variabel

Sum of

Source DF Squares Mean Square F Value Pr > F

Model 2 1.5949430 0.7974715 1.25 **0.2894**

Error 213 136.2031195 0.6394513

Corrected Total 215 137.7980625

R-Square Coeff Var Root MSE variabel Mean

0.011574 -30.72157 0.799657 -2.602917

Source DF Type I SS Mean Square F Value Pr > F

COS 1 0.22629047 0.22629047 0.35 0.5526

SIN 1 1.36865255 1.36865255 2.14 0.1449

Source DF Type III SS Mean Square F Value Pr > F

COS 1 0.22629045 0.22629045 0.35 0.5526

SIN 1 1.36865255 1.36865255 2.14 0.1449

Standard

Parameter Estimate Error t Value Pr > |t|

Intercept -2.598037115 0.05502458 -47.22 <.0001

COS -0.043915963 0.07382323 -0.59 0.5526

SIN 0.119401831 0.08161465 1.46 0.1449

AMPLITUDE, TMAX AND TMIN FOR hsa_miR_17_5p 16

Obs AMPL TMAX TMIN

1 0.25444 7.21 19.21

**hsa_miR_19_3p** 17

The MEANS Procedure

Variable Label Mean Std Dev Std Error Minimum Maximum Pr > |t|

ƒƒƒƒƒƒƒƒƒƒƒƒƒƒƒƒƒƒƒƒƒƒƒƒƒƒƒƒƒƒƒƒƒƒƒƒƒƒƒƒƒƒƒƒƒƒƒƒƒƒƒƒƒƒƒƒƒƒƒƒƒƒƒƒƒƒƒƒƒƒƒƒƒƒƒƒƒƒƒƒƒƒƒƒƒƒƒƒƒƒƒƒƒƒƒƒƒƒƒƒƒƒƒƒƒƒƒƒƒƒƒƒƒƒ

COS 0.0133471 0.2161657 0.0441246 -0.3074422 0.3390556 0.7650

SIN 0.2094506 0.2287981 0.0467032 -0.2571321 0.6107196 0.0002

Intercept Intercept -4.3858812 0.5240754 0.1069764 -5.1119494 -3.3801217 <.0001

ƒƒƒƒƒƒƒƒƒƒƒƒƒƒƒƒƒƒƒƒƒƒƒƒƒƒƒƒƒƒƒƒƒƒƒƒƒƒƒƒƒƒƒƒƒƒƒƒƒƒƒƒƒƒƒƒƒƒƒƒƒƒƒƒƒƒƒƒƒƒƒƒƒƒƒƒƒƒƒƒƒƒƒƒƒƒƒƒƒƒƒƒƒƒƒƒƒƒƒƒƒƒƒƒƒƒƒƒƒƒƒƒƒƒ

hsa_miR_19_3p 18

model test

The GLM Procedure

Number of observations 216

hsa_miR_19_3p 19

model test

The GLM Procedure

Dependent Variable: variabel

Sum of

Source DF Squares Mean Square F Value Pr > F

Model 2 4.23238149 2.11619075 4.87 **0.0085**

Error 213 92.52994027 0.43441287

Corrected Total 215 96.76232176

R-Square Coeff Var Root MSE variabel Mean

0.043740 -15.03285 0.659100 -4.384398

Source DF Type I SS Mean Square F Value Pr > F

COS 1 0.02090250 0.02090250 0.05 0.8266

SIN 1 4.21147899 4.21147899 9.69 0.0021

Source DF Type III SS Mean Square F Value Pr > F

COS 1 0.02090251 0.02090251 0.05 0.8266

SIN 1 4.21147899 4.21147899 9.69 0.0021

Standard

Parameter Estimate Error t Value Pr > |t|

Intercept -4.385881163 0.04535283 -96.71 <.0001

COS 0.013347149 0.06084721 0.22 0.8266

SIN 0.209450644 0.06726912 3.11 0.0021

AMPLITUDE, TMAX AND TMIN FOR hsa_miR_19_3p 20

Obs AMPL TMAX TMIN

1 0.41975 5.45 17.45

**hsa_miR_20a_5p** 21

The MEANS Procedure

Variable Label Mean Std Dev Std Error Minimum Maximum Pr > |t|

ƒƒƒƒƒƒƒƒƒƒƒƒƒƒƒƒƒƒƒƒƒƒƒƒƒƒƒƒƒƒƒƒƒƒƒƒƒƒƒƒƒƒƒƒƒƒƒƒƒƒƒƒƒƒƒƒƒƒƒƒƒƒƒƒƒƒƒƒƒƒƒƒƒƒƒƒƒƒƒƒƒƒƒƒƒƒƒƒƒƒƒƒƒƒƒƒƒƒƒƒƒƒƒƒƒƒƒƒƒƒƒƒƒƒ

COS -0.0495142 0.2240559 0.0457352 -0.4754819 0.3906064 0.2902

SIN 0.1803076 0.2355456 0.0480805 -0.2436091 0.5847666 0.0010

Intercept Intercept -3.4621836 0.8958811 0.1828710 -4.3800532 -2.0516346 <.0001

ƒƒƒƒƒƒƒƒƒƒƒƒƒƒƒƒƒƒƒƒƒƒƒƒƒƒƒƒƒƒƒƒƒƒƒƒƒƒƒƒƒƒƒƒƒƒƒƒƒƒƒƒƒƒƒƒƒƒƒƒƒƒƒƒƒƒƒƒƒƒƒƒƒƒƒƒƒƒƒƒƒƒƒƒƒƒƒƒƒƒƒƒƒƒƒƒƒƒƒƒƒƒƒƒƒƒƒƒƒƒƒƒƒƒ

hsa_miR_20a_5p 22

model test

The GLM Procedure

Number of observations 216 hsa_miR_20a_5p 23

model test

The GLM Procedure

Dependent Variable: variabel

Sum of

Source DF Squares Mean Square F Value Pr > F

Model 2 3.4087007 1.7043503 1.83 **0.1626**

Error 213 198.1409419 0.9302392

Corrected Total 215 201.5496426

R-Square Coeff Var Root MSE variabel Mean

0.016912 -27.81363 0.964489 -3.467685

Source DF Type I SS Mean Square F Value Pr > F

COS 1 0.28766048 0.28766048 0.31 0.5787

SIN 1 3.12104019 3.12104019 3.36 0.0684

Source DF Type III SS Mean Square F Value Pr > F

COS 1 0.28766046 0.28766046 0.31 0.5787

SIN 1 3.12104019 3.12104019 3.36 0.0684

Standard

Parameter Estimate Error t Value Pr > |t|

Intercept -3.462183612 0.06636672 -52.17 <.0001

COS -0.049514156 0.08904030 -0.56 0.5787

SIN 0.180307612 0.09843775 1.83 0.0684

AMPLITUDE, TMAX AND TMIN FOR hsa_miR_20a_5p 24

Obs AMPL TMAX TMIN

1 0.37397 7.01 19.01

**hsa_miR_21_5p** 25

The MEANS Procedure

Variable Label Mean Std Dev Std Error Minimum Maximum Pr > |t|

ƒƒƒƒƒƒƒƒƒƒƒƒƒƒƒƒƒƒƒƒƒƒƒƒƒƒƒƒƒƒƒƒƒƒƒƒƒƒƒƒƒƒƒƒƒƒƒƒƒƒƒƒƒƒƒƒƒƒƒƒƒƒƒƒƒƒƒƒƒƒƒƒƒƒƒƒƒƒƒƒƒƒƒƒƒƒƒƒƒƒƒƒƒƒƒƒƒƒƒƒƒƒƒƒƒƒƒƒƒƒƒƒƒƒ

COS 0.0253399 0.2368894 0.0483549 -0.3946387 0.4728238 0.6053

SIN 0.1663839 0.2330422 0.0475695 -0.1892767 0.7469633 0.0019

Intercept Intercept -4.7213804 0.7461155 0.1523002 -6.1447582 -3.1774649 <.0001

ƒƒƒƒƒƒƒƒƒƒƒƒƒƒƒƒƒƒƒƒƒƒƒƒƒƒƒƒƒƒƒƒƒƒƒƒƒƒƒƒƒƒƒƒƒƒƒƒƒƒƒƒƒƒƒƒƒƒƒƒƒƒƒƒƒƒƒƒƒƒƒƒƒƒƒƒƒƒƒƒƒƒƒƒƒƒƒƒƒƒƒƒƒƒƒƒƒƒƒƒƒƒƒƒƒƒƒƒƒƒƒƒƒƒ

hsa_miR_21_5p 26

model test

The GLM Procedure

Number of observations 216

hsa_miR_21_5p 27

model test

The GLM Procedure

Dependent Variable: variabel

Sum of

Source DF Squares Mean Square F Value Pr > F

Model 2 2.7329669 1.3664834 1.72 **0.1818**

Error 213 169.3898882 0.7952577

Corrected Total 215 172.1228551

R-Square Coeff Var Root MSE variabel Mean

0.015878 -18.89923 0.891772 -4.718565

Source DF Type I SS Mean Square F Value Pr > F

COS 1 0.07534073 0.07534073 0.09 0.7585

SIN 1 2.65762616 2.65762616 3.34 0.0689

Source DF Type III SS Mean Square F Value Pr > F

COS 1 0.07534074 0.07534074 0.09 0.7585

SIN 1 2.65762616 2.65762616 3.34 0.0689

Standard

Parameter Estimate Error t Value Pr > |t|

Intercept -4.721380354 0.06136306 -76.94 <.0001

COS 0.025339862 0.08232718 0.31 0.7585

SIN 0.166383910 0.09101612 1.83 0.0689

AMPLITUDE, TMAX AND TMIN FOR hsa_miR_21_5p 28

Obs AMPL TMAX TMIN

1 0.33660 5.25 17.25

**hsa_miR_23a_3p** 29

The MEANS Procedure

Variable Label Mean Std Dev Std Error Minimum Maximum Pr > |t|

ƒƒƒƒƒƒƒƒƒƒƒƒƒƒƒƒƒƒƒƒƒƒƒƒƒƒƒƒƒƒƒƒƒƒƒƒƒƒƒƒƒƒƒƒƒƒƒƒƒƒƒƒƒƒƒƒƒƒƒƒƒƒƒƒƒƒƒƒƒƒƒƒƒƒƒƒƒƒƒƒƒƒƒƒƒƒƒƒƒƒƒƒƒƒƒƒƒƒƒƒƒƒƒƒƒƒƒƒƒƒƒƒƒƒ

COS -0.0132446 0.4543503 0.0927439 -0.7273755 0.9456796 0.8877

SIN 0.2158066 0.4276872 0.0873013 -0.6743845 1.0337006 0.0213

Intercept Intercept -8.7339913 0.9954433 0.2031940 -11.0005881 -6.9324259 <.0001

ƒƒƒƒƒƒƒƒƒƒƒƒƒƒƒƒƒƒƒƒƒƒƒƒƒƒƒƒƒƒƒƒƒƒƒƒƒƒƒƒƒƒƒƒƒƒƒƒƒƒƒƒƒƒƒƒƒƒƒƒƒƒƒƒƒƒƒƒƒƒƒƒƒƒƒƒƒƒƒƒƒƒƒƒƒƒƒƒƒƒƒƒƒƒƒƒƒƒƒƒƒƒƒƒƒƒƒƒƒƒƒƒƒƒ

hsa_miR_23a_3p 30

model test

The GLM Procedure

Number of observations 216

hsa_miR_23a_3p 31

model test

The GLM Procedure

Dependent Variable: variabel

Sum of

Source DF Squares Mean Square F Value Pr > F

Model 2 4.4915420 2.2457710 1.41 **0.2454**

Error 213 338.2574117 1.5880630

Corrected Total 215 342.7489537

R-Square Coeff Var Root MSE variabel Mean

0.013104 -14.42607 1.260184 -8.735463

Source DF Type I SS Mean Square F Value Pr > F

COS 1 0.02058260 0.02058260 0.01 0.9095

SIN 1 4.47095938 4.47095938 2.82 0.0948

Source DF Type III SS Mean Square F Value Pr > F

COS 1 0.02058259 0.02058259 0.01 0.9095

SIN 1 4.47095938 4.47095938 2.82 0.0948

Standard

Parameter Estimate Error t Value Pr > |t|

Intercept -8.733991338 0.08671354 -100.72 <.0001

COS -0.013244614 0.11633842 -0.11 0.9095

SIN 0.215806610 0.12861696 1.68 0.0948

AMPLITUDE, TMAX AND TMIN FOR hsa_miR_23a_3p 32

Obs AMPL TMAX TMIN

1 0.43243 6.14 18.14

**hsa_miR_24_3p** 33

The MEANS Procedure

Variable Label Mean Std Dev Std Error Minimum Maximum Pr > |t|

ƒƒƒƒƒƒƒƒƒƒƒƒƒƒƒƒƒƒƒƒƒƒƒƒƒƒƒƒƒƒƒƒƒƒƒƒƒƒƒƒƒƒƒƒƒƒƒƒƒƒƒƒƒƒƒƒƒƒƒƒƒƒƒƒƒƒƒƒƒƒƒƒƒƒƒƒƒƒƒƒƒƒƒƒƒƒƒƒƒƒƒƒƒƒƒƒƒƒƒƒƒƒƒƒƒƒƒƒƒƒƒƒƒƒ

COS 0.0650644 0.2551601 0.0520843 -0.3071837 0.5625612 0.2242

SIN 0.2361475 0.2327812 0.0475163 -0.2148833 0.7382196 <.0001

Intercept Intercept -3.2614886 0.9784802 0.1997314 -5.0622830 -1.6854459 <.0001

ƒƒƒƒƒƒƒƒƒƒƒƒƒƒƒƒƒƒƒƒƒƒƒƒƒƒƒƒƒƒƒƒƒƒƒƒƒƒƒƒƒƒƒƒƒƒƒƒƒƒƒƒƒƒƒƒƒƒƒƒƒƒƒƒƒƒƒƒƒƒƒƒƒƒƒƒƒƒƒƒƒƒƒƒƒƒƒƒƒƒƒƒƒƒƒƒƒƒƒƒƒƒƒƒƒƒƒƒƒƒƒƒƒƒ

hsa_miR_24_3p 34

model test

The GLM Procedure

Number of observations 216

hsa_miR_24_3p 35

model test

The GLM Procedure

Dependent Variable: variabel

Sum of

Source DF Squares Mean Square F Value Pr > F

Model 2 5.8502179 2.9251089 2.49 **0.0855**

Error 213 250.4670636 1.1759017

Corrected Total 215 256.3172815

R-Square Coeff Var Root MSE variabel Mean

0.022824 -33.32218 1.084390 -3.254259

Source DF Type I SS Mean Square F Value Pr > F

COS 1 0.49671560 0.49671560 0.42 0.5164

SIN 1 5.35350227 5.35350227 4.55 0.0340

Source DF Type III SS Mean Square F Value Pr > F

COS 1 0.49671564 0.49671564 0.42 0.5164

SIN 1 5.35350227 5.35350227 4.55 0.0340

Standard

Parameter Estimate Error t Value Pr > |t|

Intercept -3.261488631 0.07461713 -43.71 <.0001

COS 0.065064368 0.10010939 0.65 0.5164

SIN 0.236147513 0.11067509 2.13 0.0340

AMPLITUDE, TMAX AND TMIN FOR hsa_miR_24_3p 36

Obs AMPL TMAX TMIN

1 0.48989 4.58 16.58

**hsa_miR_27a_3p** 37

The MEANS Procedure

Variable Label Mean Std Dev Std Error Minimum Maximum Pr > |t|

ƒƒƒƒƒƒƒƒƒƒƒƒƒƒƒƒƒƒƒƒƒƒƒƒƒƒƒƒƒƒƒƒƒƒƒƒƒƒƒƒƒƒƒƒƒƒƒƒƒƒƒƒƒƒƒƒƒƒƒƒƒƒƒƒƒƒƒƒƒƒƒƒƒƒƒƒƒƒƒƒƒƒƒƒƒƒƒƒƒƒƒƒƒƒƒƒƒƒƒƒƒƒƒƒƒƒƒƒƒƒƒƒƒƒ

COS 0.0915731 0.2417143 0.0493397 -0.2575314 0.5436485 0.0763

SIN 0.1277171 0.1795011 0.0366405 -0.1123223 0.7420153 0.0020

Intercept Intercept -6.7320729 0.8355278 0.1705514 -8.4278128 -5.4597337 <.0001

ƒƒƒƒƒƒƒƒƒƒƒƒƒƒƒƒƒƒƒƒƒƒƒƒƒƒƒƒƒƒƒƒƒƒƒƒƒƒƒƒƒƒƒƒƒƒƒƒƒƒƒƒƒƒƒƒƒƒƒƒƒƒƒƒƒƒƒƒƒƒƒƒƒƒƒƒƒƒƒƒƒƒƒƒƒƒƒƒƒƒƒƒƒƒƒƒƒƒƒƒƒƒƒƒƒƒƒƒƒƒƒƒƒƒ

hsa_miR_27a_3p 38

model test

The GLM Procedure

Number of observations 216

hsa_miR_27a_3p 39

model test

The GLM Procedure

Dependent Variable: variabel

Sum of

Source DF Squares Mean Square F Value Pr > F

Model 2 2.5498333 1.2749166 1.40 **0.2496**

Error 213 194.3660885 0.9125168

Corrected Total 215 196.9159218

R-Square Coeff Var Root MSE variabel Mean

0.012949 -14.21113 0.955257 -6.721898

Source DF Type I SS Mean Square F Value Pr > F

COS 1 0.98391332 0.98391332 1.08 0.3003

SIN 1 1.56591997 1.56591997 1.72 0.1916

Source DF Type III SS Mean Square F Value Pr > F

COS 1 0.98391335 0.98391335 1.08 0.3003

SIN 1 1.56591997 1.56591997 1.72 0.1916

Standard

Parameter Estimate Error t Value Pr > |t|

Intercept -6.732072930 0.06573149 -102.42 <.0001

COS 0.091573059 0.08818805 1.04 0.3003

SIN 0.127717133 0.09749556 1.31 0.1916

AMPLITUDE, TMAX AND TMIN FOR hsa_miR_27a_3p 40

Obs AMPL TMAX TMIN

1 0.31431 3.37 15.37

**hsa_miR_28_3p** 41

The MEANS Procedure

Variable Label Mean Std Dev Std Error Minimum Maximum Pr > |t|

ƒƒƒƒƒƒƒƒƒƒƒƒƒƒƒƒƒƒƒƒƒƒƒƒƒƒƒƒƒƒƒƒƒƒƒƒƒƒƒƒƒƒƒƒƒƒƒƒƒƒƒƒƒƒƒƒƒƒƒƒƒƒƒƒƒƒƒƒƒƒƒƒƒƒƒƒƒƒƒƒƒƒƒƒƒƒƒƒƒƒƒƒƒƒƒƒƒƒƒƒƒƒƒƒƒƒƒƒƒƒƒƒƒƒ

COS -0.1174601 0.4324145 0.0882662 -1.2870125 0.6769917 0.1963

SIN -0.2648781 0.4590007 0.0936931 -1.2353784 0.7890433 0.0096

Intercept Intercept -1.4900507 0.7414981 0.1513577 -2.3699192 0.4854573 <.0001

ƒƒƒƒƒƒƒƒƒƒƒƒƒƒƒƒƒƒƒƒƒƒƒƒƒƒƒƒƒƒƒƒƒƒƒƒƒƒƒƒƒƒƒƒƒƒƒƒƒƒƒƒƒƒƒƒƒƒƒƒƒƒƒƒƒƒƒƒƒƒƒƒƒƒƒƒƒƒƒƒƒƒƒƒƒƒƒƒƒƒƒƒƒƒƒƒƒƒƒƒƒƒƒƒƒƒƒƒƒƒƒƒƒƒ

hsa_miR_28_3p 42

model test

The GLM Procedure

Number of observations 216

hsa_miR_28_3p 43

model test

The GLM Procedure

Dependent Variable: variabel

Sum of

Source DF Squares Mean Square F Value Pr > F

Model 2 8.3542290 4.1771145 2.77 **0.0649**

Error 213 321.2211928 1.5080807

Corrected Total 215 329.5754218

R-Square Coeff Var Root MSE variabel Mean

0.025348 -81.70034 1.228039 -1.503102

Source DF Type I SS Mean Square F Value Pr > F

COS 1 1.61883224 1.61883224 1.07 0.3013

SIN 1 6.73539674 6.73539674 4.47 0.0357

Source DF Type III SS Mean Square F Value Pr > F

COS 1 1.61883231 1.61883231 1.07 0.3013

SIN 1 6.73539674 6.73539674 4.47 0.0357

Standard

Parameter Estimate Error t Value Pr > |t|

Intercept -1.490050737 0.08450168 -17.63 <.0001

COS -0.117460063 0.11337090 -1.04 0.3013

SIN -0.264878051 0.12533624 -2.11 0.0357

AMPLITUDE, TMAX AND TMIN FOR hsa_miR_28_3p 44

Obs AMPL TMAX TMIN

1 0.57951 16.24 4.24

**hsa_miR_29a_3p** 45

The MEANS Procedure

Variable Label Mean Std Dev Std Error Minimum Maximum Pr > |t|

ƒƒƒƒƒƒƒƒƒƒƒƒƒƒƒƒƒƒƒƒƒƒƒƒƒƒƒƒƒƒƒƒƒƒƒƒƒƒƒƒƒƒƒƒƒƒƒƒƒƒƒƒƒƒƒƒƒƒƒƒƒƒƒƒƒƒƒƒƒƒƒƒƒƒƒƒƒƒƒƒƒƒƒƒƒƒƒƒƒƒƒƒƒƒƒƒƒƒƒƒƒƒƒƒƒƒƒƒƒƒƒƒƒƒ

COS 0.0020338 0.3225135 0.0658328 -0.7681362 0.4229049 0.9756

SIN 0.2847954 0.3295513 0.0672694 -0.1952207 0.9293251 0.0003

Intercept Intercept -8.9811982 1.0978486 0.2240974 -11.2659907 -7.3612979 <.0001

ƒƒƒƒƒƒƒƒƒƒƒƒƒƒƒƒƒƒƒƒƒƒƒƒƒƒƒƒƒƒƒƒƒƒƒƒƒƒƒƒƒƒƒƒƒƒƒƒƒƒƒƒƒƒƒƒƒƒƒƒƒƒƒƒƒƒƒƒƒƒƒƒƒƒƒƒƒƒƒƒƒƒƒƒƒƒƒƒƒƒƒƒƒƒƒƒƒƒƒƒƒƒƒƒƒƒƒƒƒƒƒƒƒƒ

hsa_miR_29a_3p 46

model test

The GLM Procedure

Number of observations 216

hsa_miR_29a_3p 47

model test

The GLM Procedure

Dependent Variable: variabel

Sum of

Source DF Squares Mean Square F Value Pr > F

Model 2 7.7868918 3.8934459 2.35 **0.0982**

Error 213 353.4674040 1.6594714

Corrected Total 215 361.2542958

R-Square Coeff Var Root MSE variabel Mean

0.021555 -14.34371 1.288205 -8.980972

Source DF Type I SS Mean Square F Value Pr > F

COS 1 0.00048531 0.00048531 0.00 0.9864

SIN 1 7.78640653 7.78640653 4.69 0.0314

Source DF Type III SS Mean Square F Value Pr > F

COS 1 0.00048531 0.00048531 0.00 0.9864

SIN 1 7.78640653 7.78640653 4.69 0.0314

Standard

Parameter Estimate Error t Value Pr > |t|

Intercept -8.981198194 0.08864167 -101.32 <.0001

COS 0.002033760 0.11892528 0.02 0.9864

SIN 0.284795366 0.13147684 2.17 0.0314

AMPLITUDE, TMAX AND TMIN FOR hsa_miR_29a_3p 48

Obs AMPL TMAX TMIN

1 0.56961 5.58 17.58

**hsa_miR_34a_5p** 49

The MEANS Procedure

Variable Label Mean Std Dev Std Error Minimum Maximum Pr > |t|

ƒƒƒƒƒƒƒƒƒƒƒƒƒƒƒƒƒƒƒƒƒƒƒƒƒƒƒƒƒƒƒƒƒƒƒƒƒƒƒƒƒƒƒƒƒƒƒƒƒƒƒƒƒƒƒƒƒƒƒƒƒƒƒƒƒƒƒƒƒƒƒƒƒƒƒƒƒƒƒƒƒƒƒƒƒƒƒƒƒƒƒƒƒƒƒƒƒƒƒƒƒƒƒƒƒƒƒƒƒƒƒƒƒƒ

COS -0.4716754 1.1871675 0.2423295 -3.9946190 1.4022294 0.0639

SIN 0.7675194 1.2437473 0.2538789 -2.1507011 3.3847000 0.0061

Intercept Intercept 3.4180565 1.1144646 0.2274891 0.6361161 4.5559765 <.0001

ƒƒƒƒƒƒƒƒƒƒƒƒƒƒƒƒƒƒƒƒƒƒƒƒƒƒƒƒƒƒƒƒƒƒƒƒƒƒƒƒƒƒƒƒƒƒƒƒƒƒƒƒƒƒƒƒƒƒƒƒƒƒƒƒƒƒƒƒƒƒƒƒƒƒƒƒƒƒƒƒƒƒƒƒƒƒƒƒƒƒƒƒƒƒƒƒƒƒƒƒƒƒƒƒƒƒƒƒƒƒƒƒƒƒ

hsa_miR_34a_5p 50

model test

The GLM Procedure

Number of observations 216

hsa_miR_34a_5p 51

model test

The GLM Procedure

Dependent Variable: variabel

Sum of

Source DF Squares Mean Square F Value Pr > F

Model 2 82.656302 41.328151 5.25 **0.0060**

Error 213 1677.364007 7.874948

Corrected Total 215 1760.020309

R-Square Coeff Var Root MSE variabel Mean

0.046963 83.37871 2.806234 3.365648

Source DF Type I SS Mean Square F Value Pr > F

COS 1 26.10404652 26.10404652 3.31 0.0701

SIN 1 56.55225585 56.55225585 7.18 0.0079

Source DF Type III SS Mean Square F Value Pr > F

COS 1 26.10404570 26.10404570 3.31 0.0701

SIN 1 56.55225585 56.55225585 7.18 0.0079

Standard

Parameter Estimate Error t Value Pr > |t|

Intercept 3.418056521 0.19309761 17.70 <.0001

COS -0.471675379 0.25906763 -1.82 0.0701

SIN 0.767519375 0.28641004 2.68 0.0079

AMPLITUDE, TMAX AND TMIN FOR hsa_miR_34a_5p 52

Obs AMPL TMAX TMIN

1 1.80174 8.06 20.06

**hsa_miR_92a_5p** 53

The MEANS Procedure

Variable Label Mean Std Dev Std Error Minimum Maximum Pr > |t|

ƒƒƒƒƒƒƒƒƒƒƒƒƒƒƒƒƒƒƒƒƒƒƒƒƒƒƒƒƒƒƒƒƒƒƒƒƒƒƒƒƒƒƒƒƒƒƒƒƒƒƒƒƒƒƒƒƒƒƒƒƒƒƒƒƒƒƒƒƒƒƒƒƒƒƒƒƒƒƒƒƒƒƒƒƒƒƒƒƒƒƒƒƒƒƒƒƒƒƒƒƒƒƒƒƒƒƒƒƒƒƒƒƒƒ

COS -0.0024329 0.1139036 0.0232505 -0.1973619 0.2038701 0.9176

SIN 0.0171630 0.1533148 0.0312953 -0.2248223 0.4104899 0.5887

Intercept Intercept -3.9713963 0.6761674 0.1380221 -5.0370967 -3.0303348 <.0001

ƒƒƒƒƒƒƒƒƒƒƒƒƒƒƒƒƒƒƒƒƒƒƒƒƒƒƒƒƒƒƒƒƒƒƒƒƒƒƒƒƒƒƒƒƒƒƒƒƒƒƒƒƒƒƒƒƒƒƒƒƒƒƒƒƒƒƒƒƒƒƒƒƒƒƒƒƒƒƒƒƒƒƒƒƒƒƒƒƒƒƒƒƒƒƒƒƒƒƒƒƒƒƒƒƒƒƒƒƒƒƒƒƒƒ

hsa_miR_92a_5p 54

model test

The GLM Procedure

Number of observations 216

hsa_miR_92a_5p 55

model test

The GLM Procedure

Dependent Variable: variabel

Sum of

Source DF Squares Mean Square F Value Pr > F

Model 2 0.0289730 0.0144865 0.03 **0.9727**

Error 213 111.3516270 0.5227776

Corrected Total 215 111.3806000

R-Square Coeff Var Root MSE variabel Mean

0.000260 -18.20479 0.723034 -3.971667

Source DF Type I SS Mean Square F Value Pr > F

COS 1 0.00069451 0.00069451 0.00 0.9710

SIN 1 0.02827850 0.02827850 0.05 0.8163

Source DF Type III SS Mean Square F Value Pr > F

COS 1 0.00069451 0.00069451 0.00 0.9710

SIN 1 0.02827850 0.02827850 0.05 0.8163

Standard

Parameter Estimate Error t Value Pr > |t|

Intercept -3.971396342 0.04975211 -79.82 <.0001

COS -0.002432918 0.06674946 -0.04 0.9710

SIN 0.017162974 0.07379431 0.23 0.8163

AMPLITUDE, TMAX AND TMIN FOR hsa_miR_92a_5p 56

Obs AMPL TMAX TMIN

1 0.034669 6.32 18.32

**hsa_miR_100_5p** 57

The MEANS Procedure

Variable Label Mean Std Dev Std Error Minimum Maximum Pr > |t|

ƒƒƒƒƒƒƒƒƒƒƒƒƒƒƒƒƒƒƒƒƒƒƒƒƒƒƒƒƒƒƒƒƒƒƒƒƒƒƒƒƒƒƒƒƒƒƒƒƒƒƒƒƒƒƒƒƒƒƒƒƒƒƒƒƒƒƒƒƒƒƒƒƒƒƒƒƒƒƒƒƒƒƒƒƒƒƒƒƒƒƒƒƒƒƒƒƒƒƒƒƒƒƒƒƒƒƒƒƒƒƒƒƒƒ

COS -0.2174236 1.3830242 0.2823086 -2.3763362 2.8737692 0.4490

SIN 0.4602434 1.9690102 0.4019225 -3.8992174 5.1836286 0.2639

Intercept Intercept -4.3333418 1.4915312 0.3044575 -7.0819100 -2.1969561 <.0001

ƒƒƒƒƒƒƒƒƒƒƒƒƒƒƒƒƒƒƒƒƒƒƒƒƒƒƒƒƒƒƒƒƒƒƒƒƒƒƒƒƒƒƒƒƒƒƒƒƒƒƒƒƒƒƒƒƒƒƒƒƒƒƒƒƒƒƒƒƒƒƒƒƒƒƒƒƒƒƒƒƒƒƒƒƒƒƒƒƒƒƒƒƒƒƒƒƒƒƒƒƒƒƒƒƒƒƒƒƒƒƒƒƒƒ

hsa_miR_100_5p 58

model test

The GLM Procedure

Number of observations 216

hsa_miR_100_5p 59

model test

The GLM Procedure

Dependent Variable: variabel

Sum of

Source DF Squares Mean Square F Value Pr > F

Model 2 25.881807 12.940903 0.90 **0.4078**

Error 213 3060.102843 14.366680

Corrected Total 215 3085.984650

R-Square Coeff Var Root MSE variabel Mean

0.008387 -86.98429 3.790340 -4.357500

Source DF Type I SS Mean Square F Value Pr > F

COS 1 5.54670233 5.54670233 0.39 0.5350

SIN 1 20.33510421 20.33510421 1.42 0.2355

Source DF Type III SS Mean Square F Value Pr > F

COS 1 5.54670210 5.54670210 0.39 0.5350

SIN 1 20.33510421 20.33510421 1.42 0.2355

Standard

Parameter Estimate Error t Value Pr > |t|

Intercept -4.333341821 0.26081421 -16.61 <.0001

COS -0.217423618 0.34991899 -0.62 0.5350

SIN 0.460243413 0.38684999 1.19 0.2355

AMPLITUDE, TMAX AND TMIN FOR hsa_miR_100_5p 60

Obs AMPL TMAX TMIN

1 1.01803 7.41 19.41

**hsa_miR_106a_5p** 61

The MEANS Procedure

Variable Label Mean Std Dev Std Error Minimum Maximum Pr > |t|

ƒƒƒƒƒƒƒƒƒƒƒƒƒƒƒƒƒƒƒƒƒƒƒƒƒƒƒƒƒƒƒƒƒƒƒƒƒƒƒƒƒƒƒƒƒƒƒƒƒƒƒƒƒƒƒƒƒƒƒƒƒƒƒƒƒƒƒƒƒƒƒƒƒƒƒƒƒƒƒƒƒƒƒƒƒƒƒƒƒƒƒƒƒƒƒƒƒƒƒƒƒƒƒƒƒƒƒƒƒƒƒƒƒƒ

COS -0.0470783 0.1833647 0.0374292 -0.3388549 0.2687416 0.2211

SIN 0.1387893 0.1788857 0.0365149 -0.1985051 0.4772882 0.0009

Intercept Intercept -2.6253246 0.8093769 0.1652134 -3.8298944 -1.3089901 <.0001

ƒƒƒƒƒƒƒƒƒƒƒƒƒƒƒƒƒƒƒƒƒƒƒƒƒƒƒƒƒƒƒƒƒƒƒƒƒƒƒƒƒƒƒƒƒƒƒƒƒƒƒƒƒƒƒƒƒƒƒƒƒƒƒƒƒƒƒƒƒƒƒƒƒƒƒƒƒƒƒƒƒƒƒƒƒƒƒƒƒƒƒƒƒƒƒƒƒƒƒƒƒƒƒƒƒƒƒƒƒƒƒƒƒƒ

hsa_miR_106a_5p 62

model test

The GLM Procedure

Number of observations 216

hsa_miR_106a_5p 63

model test

The GLM Procedure

Dependent Variable: variabel

Sum of

Source DF Squares Mean Square F Value Pr > F

Model 2 2.1092516 1.0546258 1.37 **0.2573**

Error 213 164.4054817 0.7718567

Corrected Total 215 166.5147333

R-Square Coeff Var Root MSE variabel Mean

0.012667 -33.39803 0.878554 -2.630556

Source DF Type I SS Mean Square F Value Pr > F

COS 1 0.26005413 0.26005413 0.34 0.5622

SIN 1 1.84919752 1.84919752 2.40 0.1231

Source DF Type III SS Mean Square F Value Pr > F

COS 1 0.26005412 0.26005412 0.34 0.5622

SIN 1 1.84919752 1.84919752 2.40 0.1231

Standard

Parameter Estimate Error t Value Pr > |t|

Intercept -2.625324628 0.06045349 -43.43 <.0001

COS -0.047078343 0.08110687 -0.58 0.5622

SIN 0.138789315 0.08966702 1.55 0.1231

AMPLITUDE, TMAX AND TMIN FOR hsa_miR_106a_5p 64

Obs AMPL TMAX TMIN

1 0.29311 7.15 19.15

**hsa_miR_126_3p** 65

The MEANS Procedure

Variable Label Mean Std Dev Std Error Minimum Maximum Pr > |t|

ƒƒƒƒƒƒƒƒƒƒƒƒƒƒƒƒƒƒƒƒƒƒƒƒƒƒƒƒƒƒƒƒƒƒƒƒƒƒƒƒƒƒƒƒƒƒƒƒƒƒƒƒƒƒƒƒƒƒƒƒƒƒƒƒƒƒƒƒƒƒƒƒƒƒƒƒƒƒƒƒƒƒƒƒƒƒƒƒƒƒƒƒƒƒƒƒƒƒƒƒƒƒƒƒƒƒƒƒƒƒƒƒƒƒ

COS 0.0521025 0.2427051 0.0495420 -0.3692235 0.5608450 0.3039

SIN 0.1872355 0.2209280 0.0450967 -0.2294759 0.6109708 0.0004

Intercept Intercept -3.0919003 0.8438277 0.1722456 -4.5323161 -1.8067449 <.0001

ƒƒƒƒƒƒƒƒƒƒƒƒƒƒƒƒƒƒƒƒƒƒƒƒƒƒƒƒƒƒƒƒƒƒƒƒƒƒƒƒƒƒƒƒƒƒƒƒƒƒƒƒƒƒƒƒƒƒƒƒƒƒƒƒƒƒƒƒƒƒƒƒƒƒƒƒƒƒƒƒƒƒƒƒƒƒƒƒƒƒƒƒƒƒƒƒƒƒƒƒƒƒƒƒƒƒƒƒƒƒƒƒƒƒ

hsa_miR_126_3p 66

model test

The GLM Procedure

Number of observations 216

hsa_miR_126_3p 67

model test

The GLM Procedure

Dependent Variable: variabel

Sum of

Source DF Squares Mean Square F Value Pr > F

Model 2 3.6840077 1.8420038 2.11 **0.1243**

Error 213 186.3505257 0.8748851

Corrected Total 215 190.0345333

R-Square Coeff Var Root MSE variabel Mean

0.019386 -30.30847 0.935353 -3.086111

Source DF Type I SS Mean Square F Value Pr > F

COS 1 0.31852115 0.31852115 0.36 0.5469

SIN 1 3.36548651 3.36548651 3.85 0.0511

Source DF Type III SS Mean Square F Value Pr > F

COS 1 0.31852118 0.31852118 0.36 0.5469

SIN 1 3.36548651 3.36548651 3.85 0.0511

Standard

Parameter Estimate Error t Value Pr > |t|

Intercept -3.091900274 0.06436185 -48.04 <.0001

COS 0.052102487 0.08635049 0.60 0.5469

SIN 0.187235549 0.09546406 1.96 0.0511

AMPLITUDE, TMAX AND TMIN FOR hsa_miR_126_3p 68

Obs AMPL TMAX TMIN

1 0.38870 4.58 16.58

**hsa_miR_140_5p** 69

The MEANS Procedure

Variable Label Mean Std Dev Std Error Minimum Maximum Pr > |t|

ƒƒƒƒƒƒƒƒƒƒƒƒƒƒƒƒƒƒƒƒƒƒƒƒƒƒƒƒƒƒƒƒƒƒƒƒƒƒƒƒƒƒƒƒƒƒƒƒƒƒƒƒƒƒƒƒƒƒƒƒƒƒƒƒƒƒƒƒƒƒƒƒƒƒƒƒƒƒƒƒƒƒƒƒƒƒƒƒƒƒƒƒƒƒƒƒƒƒƒƒƒƒƒƒƒƒƒƒƒƒƒƒƒƒ

COS -0.0155728 0.2534383 0.0517329 -0.5104933 0.4364103 0.7661

SIN 0.2830685 0.2748630 0.0561062 -0.3415254 0.7888567 <.0001

Intercept Intercept -5.2874827 0.5324073 0.1086772 -6.0964432 -4.1694836 <.0001

ƒƒƒƒƒƒƒƒƒƒƒƒƒƒƒƒƒƒƒƒƒƒƒƒƒƒƒƒƒƒƒƒƒƒƒƒƒƒƒƒƒƒƒƒƒƒƒƒƒƒƒƒƒƒƒƒƒƒƒƒƒƒƒƒƒƒƒƒƒƒƒƒƒƒƒƒƒƒƒƒƒƒƒƒƒƒƒƒƒƒƒƒƒƒƒƒƒƒƒƒƒƒƒƒƒƒƒƒƒƒƒƒƒƒ

hsa_miR_140_5p 70

model test

The GLM Procedure

Number of observations 216

hsa_miR_140_5p 71

model test

The GLM Procedure

Dependent Variable: variabel

Sum of

Source DF Squares Mean Square F Value Pr > F

Model 2 7.7207219 3.8603609 7.33 **0.0008**

Error 213 112.2520443 0.5270049

Corrected Total 215 119.9727662

R-Square Coeff Var Root MSE variabel Mean

0.064354 -13.72512 0.725951 -5.289213

Source DF Type I SS Mean Square F Value Pr > F

COS 1 0.02845472 0.02845472 0.05 0.8165

SIN 1 7.69226717 7.69226717 14.60 0.0002

Source DF Type III SS Mean Square F Value Pr > F

COS 1 0.02845471 0.02845471 0.05 0.8165

SIN 1 7.69226717 7.69226717 14.60 0.0002

Standard

Parameter Estimate Error t Value Pr > |t|

Intercept -5.287482652 0.04995286 -105.85 <.0001

COS -0.015572788 0.06701880 -0.23 0.8165

SIN 0.283068511 0.07409207 3.82 0.0002

AMPLITUDE, TMAX AND TMIN FOR hsa_miR_140_5p 72

Obs AMPL TMAX TMIN

1 0.56699 6.13 18.13

**hsa_miR_142_3p** 73

The MEANS Procedure

Variable Label Mean Std Dev Std Error Minimum Maximum Pr > |t|

ƒƒƒƒƒƒƒƒƒƒƒƒƒƒƒƒƒƒƒƒƒƒƒƒƒƒƒƒƒƒƒƒƒƒƒƒƒƒƒƒƒƒƒƒƒƒƒƒƒƒƒƒƒƒƒƒƒƒƒƒƒƒƒƒƒƒƒƒƒƒƒƒƒƒƒƒƒƒƒƒƒƒƒƒƒƒƒƒƒƒƒƒƒƒƒƒƒƒƒƒƒƒƒƒƒƒƒƒƒƒƒƒƒƒ

COS -0.0422373 0.2771627 0.0565756 -0.6918553 0.5662272 0.4629

SIN 0.1187083 0.2521502 0.0514699 -0.4860750 0.6205940 0.0304

Intercept Intercept -5.0060477 0.8896434 0.1815977 -6.5219644 -3.4964310 <.0001

ƒƒƒƒƒƒƒƒƒƒƒƒƒƒƒƒƒƒƒƒƒƒƒƒƒƒƒƒƒƒƒƒƒƒƒƒƒƒƒƒƒƒƒƒƒƒƒƒƒƒƒƒƒƒƒƒƒƒƒƒƒƒƒƒƒƒƒƒƒƒƒƒƒƒƒƒƒƒƒƒƒƒƒƒƒƒƒƒƒƒƒƒƒƒƒƒƒƒƒƒƒƒƒƒƒƒƒƒƒƒƒƒƒƒ

hsa_miR_142_3p 74

model test

The GLM Procedure

Number of observations 216

hsa_miR_142_3p 75

model test

The GLM Procedure

Dependent Variable: variabel

Sum of

Source DF Squares Mean Square F Value Pr > F

Model 2 1.5621201 0.7810601 0.78 **0.4592**

Error 213 212.9921613 0.9999632

Corrected Total 215 214.5542815

R-Square Coeff Var Root MSE variabel Mean

0.007281 -19.95676 0.999982 -5.010741

Source DF Type I SS Mean Square F Value Pr > F

COS 1 0.20932172 0.20932172 0.21 0.6478

SIN 1 1.35279842 1.35279842 1.35 0.2461

Source DF Type III SS Mean Square F Value Pr > F

COS 1 0.20932171 0.20932171 0.21 0.6478

SIN 1 1.35279842 1.35279842 1.35 0.2461

Standard

Parameter Estimate Error t Value Pr > |t|

Intercept -5.006047704 0.06880897 -72.75 <.0001

COS -0.042237327 0.09231692 -0.46 0.6478

SIN 0.118708256 0.10206019 1.16 0.2461

AMPLITUDE, TMAX AND TMIN FOR hsa_miR_142_3p 76

Obs AMPL TMAX TMIN

1 0.25200 7.18 19.18

**hsa_miR_146a_5p** 77

The MEANS Procedure

Variable Label Mean Std Dev Std Error Minimum Maximum Pr > |t|

ƒƒƒƒƒƒƒƒƒƒƒƒƒƒƒƒƒƒƒƒƒƒƒƒƒƒƒƒƒƒƒƒƒƒƒƒƒƒƒƒƒƒƒƒƒƒƒƒƒƒƒƒƒƒƒƒƒƒƒƒƒƒƒƒƒƒƒƒƒƒƒƒƒƒƒƒƒƒƒƒƒƒƒƒƒƒƒƒƒƒƒƒƒƒƒƒƒƒƒƒƒƒƒƒƒƒƒƒƒƒƒƒƒƒ

COS 0.0546080 0.2528833 0.0516196 -0.3461060 0.5664859 0.3011

SIN 0.1222185 0.2094188 0.0427474 -0.1787957 0.6077907 0.0089

Intercept Intercept -2.5252805 0.7858757 0.1604162 -3.9474772 -1.3613408 <.0001

ƒƒƒƒƒƒƒƒƒƒƒƒƒƒƒƒƒƒƒƒƒƒƒƒƒƒƒƒƒƒƒƒƒƒƒƒƒƒƒƒƒƒƒƒƒƒƒƒƒƒƒƒƒƒƒƒƒƒƒƒƒƒƒƒƒƒƒƒƒƒƒƒƒƒƒƒƒƒƒƒƒƒƒƒƒƒƒƒƒƒƒƒƒƒƒƒƒƒƒƒƒƒƒƒƒƒƒƒƒƒƒƒƒƒ

hsa_miR_146a_5p 78

model test

The GLM Procedure

Number of observations 216

hsa_miR_146a_5p 79

model test

The GLM Procedure

Dependent Variable: variabel

Sum of

Source DF Squares Mean Square F Value Pr > F

Model 2 1.7838786 0.8919393 1.10 **0.3337**

Error 213 172.2282876 0.8085835

Corrected Total 215 174.0121662

R-Square Coeff Var Root MSE variabel Mean

0.010251 -35.69419 0.899213 -2.519213

Source DF Type I SS Mean Square F Value Pr > F

COS 1 0.34989160 0.34989160 0.43 0.5114

SIN 1 1.43398703 1.43398703 1.77 0.1844

Source DF Type III SS Mean Square F Value Pr > F

COS 1 0.34989161 0.34989161 0.43 0.5114

SIN 1 1.43398703 1.43398703 1.77 0.1844

Standard

Parameter Estimate Error t Value Pr > |t|

Intercept -2.525280514 0.06187504 -40.81 <.0001

COS 0.054607974 0.08301408 0.66 0.5114

SIN 0.122218512 0.09177551 1.33 0.1844

AMPLITUDE, TMAX AND TMIN FOR hsa_miR_146a_5p 80

Obs AMPL TMAX TMIN

1 0.26773 4.24 16.24

**hsa_miR_146b_5p** 81

The MEANS Procedure

Variable Label Mean Std Dev Std Error Minimum Maximum Pr > |t|

ƒƒƒƒƒƒƒƒƒƒƒƒƒƒƒƒƒƒƒƒƒƒƒƒƒƒƒƒƒƒƒƒƒƒƒƒƒƒƒƒƒƒƒƒƒƒƒƒƒƒƒƒƒƒƒƒƒƒƒƒƒƒƒƒƒƒƒƒƒƒƒƒƒƒƒƒƒƒƒƒƒƒƒƒƒƒƒƒƒƒƒƒƒƒƒƒƒƒƒƒƒƒƒƒƒƒƒƒƒƒƒƒƒƒ

COS 0.0490053 0.3101966 0.0633186 -0.4326084 0.6960064 0.4468

SIN 0.1670658 0.2692743 0.0549654 -0.2560786 0.7687311 0.0058

Intercept Intercept -6.5921117 0.7910277 0.1614678 -7.9364853 -5.2628328 <.0001

ƒƒƒƒƒƒƒƒƒƒƒƒƒƒƒƒƒƒƒƒƒƒƒƒƒƒƒƒƒƒƒƒƒƒƒƒƒƒƒƒƒƒƒƒƒƒƒƒƒƒƒƒƒƒƒƒƒƒƒƒƒƒƒƒƒƒƒƒƒƒƒƒƒƒƒƒƒƒƒƒƒƒƒƒƒƒƒƒƒƒƒƒƒƒƒƒƒƒƒƒƒƒƒƒƒƒƒƒƒƒƒƒƒƒ

hsa_miR_146b_5p 82

model test

The GLM Procedure

Number of observations 216

hsa_miR_146b_5p 83

model test

The GLM Procedure

Dependent Variable: variabel

Sum of

Source DF Squares Mean Square F Value Pr > F

Model 2 2.9612323 1.4806161 1.69 **0.1870**

Error 213 186.5939677 0.8760280

Corrected Total 215 189.5552000

R-Square Coeff Var Root MSE variabel Mean

0.015622 -14.20997 0.935964 -6.586667

Source DF Type I SS Mean Square F Value Pr > F

COS 1 0.28177781 0.28177781 0.32 0.5712

SIN 1 2.67945446 2.67945446 3.06 0.0817

Source DF Type III SS Mean Square F Value Pr > F

COS 1 0.28177783 0.28177783 0.32 0.5712

SIN 1 2.67945446 2.67945446 3.06 0.0817

Standard

Parameter Estimate Error t Value Pr > |t|

Intercept -6.592111694 0.06440388 -102.36 <.0001

COS 0.049005261 0.08640687 0.57 0.5712

SIN 0.167065806 0.09552639 1.75 0.0817

AMPLITUDE, TMAX AND TMIN FOR hsa_miR_146b_5p 84

Obs AMPL TMAX TMIN

1 0.34821 4.55 16.55

**hsa_miR_150_5p** 85

The MEANS Procedure

Variable Label Mean Std Dev Std Error Minimum Maximum Pr > |t|

ƒƒƒƒƒƒƒƒƒƒƒƒƒƒƒƒƒƒƒƒƒƒƒƒƒƒƒƒƒƒƒƒƒƒƒƒƒƒƒƒƒƒƒƒƒƒƒƒƒƒƒƒƒƒƒƒƒƒƒƒƒƒƒƒƒƒƒƒƒƒƒƒƒƒƒƒƒƒƒƒƒƒƒƒƒƒƒƒƒƒƒƒƒƒƒƒƒƒƒƒƒƒƒƒƒƒƒƒƒƒƒƒƒƒ

COS -0.1155852 0.2549425 0.0520399 -0.5175908 0.5087009 0.0365

SIN -0.1318230 0.2990743 0.0610483 -0.6369328 0.5018377 0.0415

Intercept Intercept -2.5709998 0.7817841 0.1595810 -4.1441421 -1.3431334 <.0001

ƒƒƒƒƒƒƒƒƒƒƒƒƒƒƒƒƒƒƒƒƒƒƒƒƒƒƒƒƒƒƒƒƒƒƒƒƒƒƒƒƒƒƒƒƒƒƒƒƒƒƒƒƒƒƒƒƒƒƒƒƒƒƒƒƒƒƒƒƒƒƒƒƒƒƒƒƒƒƒƒƒƒƒƒƒƒƒƒƒƒƒƒƒƒƒƒƒƒƒƒƒƒƒƒƒƒƒƒƒƒƒƒƒƒ

hsa_miR_150_5p 86

model test

The GLM Procedure

Number of observations 216

hsa_miR_150_5p 87

model test

The GLM Procedure

Dependent Variable: variabel

Sum of

Source DF Squares Mean Square F Value Pr > F

Model 2 3.2357860 1.6178930 1.78 **0.1703**

Error 213 193.0699246 0.9064316

Corrected Total 215 196.3057106

R-Square Coeff Var Root MSE variabel Mean

0.016483 -36.84694 0.952067 -2.583843

Source DF Type I SS Mean Square F Value Pr > F

COS 1 1.56756506 1.56756506 1.73 0.1899

SIN 1 1.66822097 1.66822097 1.84 0.1763

Source DF Type III SS Mean Square F Value Pr > F

COS 1 1.56756510 1.56756510 1.73 0.1899

SIN 1 1.66822097 1.66822097 1.84 0.1763

Standard

Parameter Estimate Error t Value Pr > |t|

Intercept -2.570999799 0.06551195 -39.24 <.0001

COS -0.115585163 0.08789351 -1.32 0.1899

SIN -0.131822993 0.09716993 -1.36 0.1763

AMPLITUDE, TMAX AND TMIN FOR hsa_miR_150_5p 88

Obs AMPL TMAX TMIN

1 0.35064 15.15 3.15

**hsa_miR_155_5p** 89

The MEANS Procedure

Variable Label Mean Std Dev Std Error Minimum Maximum Pr > |t|

ƒƒƒƒƒƒƒƒƒƒƒƒƒƒƒƒƒƒƒƒƒƒƒƒƒƒƒƒƒƒƒƒƒƒƒƒƒƒƒƒƒƒƒƒƒƒƒƒƒƒƒƒƒƒƒƒƒƒƒƒƒƒƒƒƒƒƒƒƒƒƒƒƒƒƒƒƒƒƒƒƒƒƒƒƒƒƒƒƒƒƒƒƒƒƒƒƒƒƒƒƒƒƒƒƒƒƒƒƒƒƒƒƒƒ

COS 0.0499210 0.2330254 0.0475661 -0.3344250 0.5565043 0.3048

SIN -0.1569564 0.2969566 0.0606160 -0.7618072 0.3887957 0.0164

Intercept Intercept 6.1610273 0.4696789 0.0958728 5.1426106 6.9127139 <.0001

ƒƒƒƒƒƒƒƒƒƒƒƒƒƒƒƒƒƒƒƒƒƒƒƒƒƒƒƒƒƒƒƒƒƒƒƒƒƒƒƒƒƒƒƒƒƒƒƒƒƒƒƒƒƒƒƒƒƒƒƒƒƒƒƒƒƒƒƒƒƒƒƒƒƒƒƒƒƒƒƒƒƒƒƒƒƒƒƒƒƒƒƒƒƒƒƒƒƒƒƒƒƒƒƒƒƒƒƒƒƒƒƒƒƒ

hsa_miR_155_5p 90

model test

The GLM Procedure

Number of observations 216

hsa_miR_155_5p 91

model test

The GLM Procedure

Dependent Variable: variabel

Sum of

Source DF Squares Mean Square F Value Pr > F

Model 2 2.65739720 1.32869860 2.94 **0.0550**

Error 213 96.26706762 0.45195806

Corrected Total 215 98.92446481

R-Square Coeff Var Root MSE variabel Mean

0.026863 10.90197 0.672278 6.166574

Source DF Type I SS Mean Square F Value Pr > F

COS 1 0.29240695 0.29240695 0.65 0.4221

SIN 1 2.36499025 2.36499025 5.23 0.0231

Source DF Type III SS Mean Square F Value Pr > F

COS 1 0.29240693 0.29240693 0.65 0.4221

SIN 1 2.36499025 2.36499025 5.23 0.0231

Standard

Parameter Estimate Error t Value Pr > |t|

Intercept 6.161027298 0.04625963 133.18 <.0001

COS 0.049920983 0.06206380 0.80 0.4221

SIN -0.156956410 0.06861411 -2.29 0.0231

AMPLITUDE, TMAX AND TMIN FOR hsa_miR_155_5p 92

Obs AMPL TMAX TMIN

1 0.32941 19.11 7.11

**hsa_miR_181b_5p** 93

The MEANS Procedure

Variable Label Mean Std Dev Std Error Minimum Maximum Pr > |t|

ƒƒƒƒƒƒƒƒƒƒƒƒƒƒƒƒƒƒƒƒƒƒƒƒƒƒƒƒƒƒƒƒƒƒƒƒƒƒƒƒƒƒƒƒƒƒƒƒƒƒƒƒƒƒƒƒƒƒƒƒƒƒƒƒƒƒƒƒƒƒƒƒƒƒƒƒƒƒƒƒƒƒƒƒƒƒƒƒƒƒƒƒƒƒƒƒƒƒƒƒƒƒƒƒƒƒƒƒƒƒƒƒƒƒ

COS -0.2220072 0.4288117 0.0875308 -0.8415283 1.1909288 0.0185

SIN 0.0178157 0.3104183 0.0633639 -0.5629073 0.5818072 0.7811

Intercept Intercept -4.0036659 1.0695303 0.2183170 -5.8010966 -1.6990771 <.0001

ƒƒƒƒƒƒƒƒƒƒƒƒƒƒƒƒƒƒƒƒƒƒƒƒƒƒƒƒƒƒƒƒƒƒƒƒƒƒƒƒƒƒƒƒƒƒƒƒƒƒƒƒƒƒƒƒƒƒƒƒƒƒƒƒƒƒƒƒƒƒƒƒƒƒƒƒƒƒƒƒƒƒƒƒƒƒƒƒƒƒƒƒƒƒƒƒƒƒƒƒƒƒƒƒƒƒƒƒƒƒƒƒƒƒ

hsa_miR_181b_5p 94

model test

The GLM Procedure

Number of observations 216

hsa_miR_181b_5p 95

model test

The GLM Procedure

Dependent Variable: variabel

Sum of

Source DF Squares Mean Square F Value Pr > F

Model 2 5.8135037 2.9067519 1.81 **0.1664**

Error 213 342.3638963 1.6073422

Corrected Total 215 348.1774000

R-Square Coeff Var Root MSE variabel Mean

0.016697 -31.47232 1.267810 -4.028333

Source DF Type I SS Mean Square F Value Pr > F

COS 1 5.78303328 5.78303328 3.60 0.0592

SIN 1 0.03047044 0.03047044 0.02 0.8906

Source DF Type III SS Mean Square F Value Pr > F

COS 1 5.78303327 5.78303327 3.60 0.0592

SIN 1 0.03047044 0.03047044 0.02 0.8906

Standard

Parameter Estimate Error t Value Pr > |t|

Intercept -4.003665865 0.08723831 -45.89 <.0001

COS -0.222007242 0.11704247 -1.90 0.0592

SIN 0.017815735 0.12939532 0.14 0.8906

AMPLITUDE, TMAX AND TMIN FOR hsa_miR_181b_5p 96

Obs AMPL TMAX TMIN

1 0.44544 11.42 23.42

**hsa_miR_184** 97

The MEANS Procedure

Variable Label Mean Std Dev Std Error Minimum Maximum Pr > |t|

ƒƒƒƒƒƒƒƒƒƒƒƒƒƒƒƒƒƒƒƒƒƒƒƒƒƒƒƒƒƒƒƒƒƒƒƒƒƒƒƒƒƒƒƒƒƒƒƒƒƒƒƒƒƒƒƒƒƒƒƒƒƒƒƒƒƒƒƒƒƒƒƒƒƒƒƒƒƒƒƒƒƒƒƒƒƒƒƒƒƒƒƒƒƒƒƒƒƒƒƒƒƒƒƒƒƒƒƒƒƒƒƒƒƒ

COS -0.0662342 0.3542264 0.0723062 -1.3108079 0.6529732 0.3692

SIN -0.1324562 0.5561787 0.1135295 -0.7614734 1.7053947 0.2553

Intercept Intercept 4.1713408 0.8310091 0.1696290 2.3876305 5.5733693 <.0001

ƒƒƒƒƒƒƒƒƒƒƒƒƒƒƒƒƒƒƒƒƒƒƒƒƒƒƒƒƒƒƒƒƒƒƒƒƒƒƒƒƒƒƒƒƒƒƒƒƒƒƒƒƒƒƒƒƒƒƒƒƒƒƒƒƒƒƒƒƒƒƒƒƒƒƒƒƒƒƒƒƒƒƒƒƒƒƒƒƒƒƒƒƒƒƒƒƒƒƒƒƒƒƒƒƒƒƒƒƒƒƒƒƒƒ

hsa_miR_184 98

model test

The GLM Procedure

Number of observations 216

hsa_miR_184 99

model test

The GLM Procedure

Dependent Variable: variabel

Sum of

Source DF Squares Mean Square F Value Pr > F

Model 2 2.1990240 1.0995120 0.75 **0.4720**

Error 213 310.8263519 1.4592786

Corrected Total 215 313.0253759

R-Square Coeff Var Root MSE variabel Mean

0.007025 29.01084 1.208006 4.163981

Source DF Type I SS Mean Square F Value Pr > F

COS 1 0.51473775 0.51473775 0.35 0.5532

SIN 1 1.68428624 1.68428624 1.15 0.2839

Source DF Type III SS Mean Square F Value Pr > F

COS 1 0.51473777 0.51473777 0.35 0.5532

SIN 1 1.68428624 1.68428624 1.15 0.2839

Standard

Parameter Estimate Error t Value Pr > |t|

Intercept 4.171340836 0.08312318 50.18 <.0001

COS -0.066234203 0.11152145 -0.59 0.5532

SIN -0.132456212 0.12329160 -1.07 0.2839

AMPLITUDE, TMAX AND TMIN FOR hsa_miR_184 100

Obs AMPL TMAX TMIN

1 0.29619 16.14 4.14

**hsa_miR_191_5p** 101

The MEANS Procedure

Variable Label Mean Std Dev Std Error Minimum Maximum Pr > |t|

ƒƒƒƒƒƒƒƒƒƒƒƒƒƒƒƒƒƒƒƒƒƒƒƒƒƒƒƒƒƒƒƒƒƒƒƒƒƒƒƒƒƒƒƒƒƒƒƒƒƒƒƒƒƒƒƒƒƒƒƒƒƒƒƒƒƒƒƒƒƒƒƒƒƒƒƒƒƒƒƒƒƒƒƒƒƒƒƒƒƒƒƒƒƒƒƒƒƒƒƒƒƒƒƒƒƒƒƒƒƒƒƒƒƒ

COS -0.0477212 0.2967971 0.0605835 -0.5476575 0.5331715 0.4389

SIN 0.2392673 0.3061197 0.0624864 -0.3226345 0.7917857 0.0009

Intercept Intercept -3.5140032 0.9261839 0.1890565 -5.0148342 -1.8024914 <.0001

ƒƒƒƒƒƒƒƒƒƒƒƒƒƒƒƒƒƒƒƒƒƒƒƒƒƒƒƒƒƒƒƒƒƒƒƒƒƒƒƒƒƒƒƒƒƒƒƒƒƒƒƒƒƒƒƒƒƒƒƒƒƒƒƒƒƒƒƒƒƒƒƒƒƒƒƒƒƒƒƒƒƒƒƒƒƒƒƒƒƒƒƒƒƒƒƒƒƒƒƒƒƒƒƒƒƒƒƒƒƒƒƒƒƒ

hsa_miR_191_5p 102

model test

The GLM Procedure

Number of observations 216

hsa_miR_191_5p 103

model test

The GLM Procedure

Dependent Variable: variabel

Sum of

Source DF Squares Mean Square F Value Pr > F

Model 2 5.7630926 2.8815463 2.56 **0.0798**

Error 213 239.9475033 1.1265141

Corrected Total 215 245.7105958

R-Square Coeff Var Root MSE variabel Mean

0.023455 -30.15861 1.061374 -3.519306

Source DF Type I SS Mean Square F Value Pr > F

COS 1 0.26720450 0.26720450 0.24 0.6267

SIN 1 5.49588805 5.49588805 4.88 0.0283

Source DF Type III SS Mean Square F Value Pr > F

COS 1 0.26720447 0.26720447 0.24 0.6267

SIN 1 5.49588805 5.49588805 4.88 0.0283

Standard

Parameter Estimate Error t Value Pr > |t|

Intercept -3.514003202 0.07303337 -48.12 <.0001

COS -0.047721178 0.09798455 -0.49 0.6267

SIN 0.239267284 0.10832600 2.21 0.0283

AMPLITUDE, TMAX AND TMIN FOR hsa_miR_191_5p 104

Obs AMPL TMAX TMIN

1 0.48796 6.45 18.45

**hsa_miR_192_5p** 105

The MEANS Procedure

Variable Label Mean Std Dev Std Error Minimum Maximum Pr > |t|

ƒƒƒƒƒƒƒƒƒƒƒƒƒƒƒƒƒƒƒƒƒƒƒƒƒƒƒƒƒƒƒƒƒƒƒƒƒƒƒƒƒƒƒƒƒƒƒƒƒƒƒƒƒƒƒƒƒƒƒƒƒƒƒƒƒƒƒƒƒƒƒƒƒƒƒƒƒƒƒƒƒƒƒƒƒƒƒƒƒƒƒƒƒƒƒƒƒƒƒƒƒƒƒƒƒƒƒƒƒƒƒƒƒƒ

COS -0.4765306 0.4767303 0.0973122 -1.4243247 0.2161084 <.0001

SIN 0.0445316 0.5846596 0.1193431 -1.3834062 1.1518771 0.7125

Intercept Intercept -8.4320985 0.8822964 0.1800980 -9.7401552 -6.6979443 <.0001

ƒƒƒƒƒƒƒƒƒƒƒƒƒƒƒƒƒƒƒƒƒƒƒƒƒƒƒƒƒƒƒƒƒƒƒƒƒƒƒƒƒƒƒƒƒƒƒƒƒƒƒƒƒƒƒƒƒƒƒƒƒƒƒƒƒƒƒƒƒƒƒƒƒƒƒƒƒƒƒƒƒƒƒƒƒƒƒƒƒƒƒƒƒƒƒƒƒƒƒƒƒƒƒƒƒƒƒƒƒƒƒƒƒƒ

hsa_miR_192_5p 106

model test

The GLM Procedure

Number of observations 216

hsa_miR_192_5p 107

model test

The GLM Procedure

Dependent Variable: variabel

Sum of

Source DF Squares Mean Square F Value Pr > F

Model 2 26.8345915 13.4172957 7.52 **0.0007**

Error 213 380.1662080 1.7848179

Corrected Total 215 407.0007995

R-Square Coeff Var Root MSE variabel Mean

0.065933 -15.74500 1.335971 -8.485046

Source DF Type I SS Mean Square F Value Pr > F

COS 1 26.64421727 26.64421727 14.93 0.0001

SIN 1 0.19037422 0.19037422 0.11 0.7443

Source DF Type III SS Mean Square F Value Pr > F

COS 1 26.64421723 26.64421723 14.93 0.0001

SIN 1 0.19037422 0.19037422 0.11 0.7443

Standard

Parameter Estimate Error t Value Pr > |t|

Intercept -8.432098460 0.09192846 -91.72 <.0001

COS -0.476530584 0.12333498 -3.86 0.0001

SIN 0.044531616 0.13635194 0.33 0.7443

AMPLITUDE, TMAX AND TMIN FOR hsa_miR_192_5p 108

Obs AMPL TMAX TMIN

1 0.95721 11.39 23.39

**hsa_miR_203_3p** 109

The MEANS Procedure

Variable Label Mean Std Dev Std Error Minimum Maximum Pr > |t|

ƒƒƒƒƒƒƒƒƒƒƒƒƒƒƒƒƒƒƒƒƒƒƒƒƒƒƒƒƒƒƒƒƒƒƒƒƒƒƒƒƒƒƒƒƒƒƒƒƒƒƒƒƒƒƒƒƒƒƒƒƒƒƒƒƒƒƒƒƒƒƒƒƒƒƒƒƒƒƒƒƒƒƒƒƒƒƒƒƒƒƒƒƒƒƒƒƒƒƒƒƒƒƒƒƒƒƒƒƒƒƒƒƒƒ

COS -0.1861322 0.3652338 0.0745530 -1.0107319 0.4424037 0.0201

SIN 0.3272318 0.3860169 0.0787954 -0.4277944 1.1501219 0.0004

Intercept Intercept -4.9137168 0.9218599 0.1881739 -6.6288266 -3.6701162 <.0001

ƒƒƒƒƒƒƒƒƒƒƒƒƒƒƒƒƒƒƒƒƒƒƒƒƒƒƒƒƒƒƒƒƒƒƒƒƒƒƒƒƒƒƒƒƒƒƒƒƒƒƒƒƒƒƒƒƒƒƒƒƒƒƒƒƒƒƒƒƒƒƒƒƒƒƒƒƒƒƒƒƒƒƒƒƒƒƒƒƒƒƒƒƒƒƒƒƒƒƒƒƒƒƒƒƒƒƒƒƒƒƒƒƒƒ

hsa_miR_203_3p 110

model test

The GLM Procedure

Number of observations 216

hsa_miR_203_3p 111

model test

The GLM Procedure

Dependent Variable: variabel

Sum of

Source DF Squares Mean Square F Value Pr > F

Model 2 14.3447754 7.1723877 4.92 **0.0082**

Error 213 310.6231464 1.4583246

Corrected Total 215 324.9679218

R-Square Coeff Var Root MSE variabel Mean

0.044142 -24.47332 1.207611 -4.934398

Source DF Type I SS Mean Square F Value Pr > F

COS 1 4.06503586 4.06503586 2.79 0.0965

SIN 1 10.27973953 10.27973953 7.05 0.0085

Source DF Type III SS Mean Square F Value Pr > F

COS 1 4.06503572 4.06503572 2.79 0.0965

SIN 1 10.27973953 10.27973953 7.05 0.0085

Standard

Parameter Estimate Error t Value Pr > |t|

Intercept -4.913716795 0.08309601 -59.13 <.0001

COS -0.186132187 0.11148499 -1.67 0.0965

SIN 0.327231751 0.12325129 2.65 0.0085

AMPLITUDE, TMAX AND TMIN FOR hsa_miR_203_3p 112

Obs AMPL TMAX TMIN

1 0.75293 7.59 19.59

**hsa_miR_208a_3p** 113

The MEANS Procedure

Variable Label Mean Std Dev Std Error Minimum Maximum Pr > |t|

ƒƒƒƒƒƒƒƒƒƒƒƒƒƒƒƒƒƒƒƒƒƒƒƒƒƒƒƒƒƒƒƒƒƒƒƒƒƒƒƒƒƒƒƒƒƒƒƒƒƒƒƒƒƒƒƒƒƒƒƒƒƒƒƒƒƒƒƒƒƒƒƒƒƒƒƒƒƒƒƒƒƒƒƒƒƒƒƒƒƒƒƒƒƒƒƒƒƒƒƒƒƒƒƒƒƒƒƒƒƒƒƒƒƒ

COS 0.1747223 0.3436968 0.0701568 -0.3905470 0.8490792 0.0204

SIN -0.0773344 0.3540068 0.0722613 -0.7820763 0.6436790 0.2956

Intercept Intercept 4.9812809 0.5069845 0.1034878 4.2535820 6.3266965 <.0001

ƒƒƒƒƒƒƒƒƒƒƒƒƒƒƒƒƒƒƒƒƒƒƒƒƒƒƒƒƒƒƒƒƒƒƒƒƒƒƒƒƒƒƒƒƒƒƒƒƒƒƒƒƒƒƒƒƒƒƒƒƒƒƒƒƒƒƒƒƒƒƒƒƒƒƒƒƒƒƒƒƒƒƒƒƒƒƒƒƒƒƒƒƒƒƒƒƒƒƒƒƒƒƒƒƒƒƒƒƒƒƒƒƒƒ

hsa_miR_208a_3p 114

model test

The GLM Procedure

Number of observations 216

hsa_miR_208a_3p 115

model test

The GLM Procedure

Dependent Variable: variabel

Sum of

Source DF Squares Mean Square F Value Pr > F

Model 2 4.1560770 2.0780385 3.61 **0.0288**

Error 213 122.6573188 0.5758560

Corrected Total 215 126.8133958

R-Square Coeff Var Root MSE variabel Mean

0.032773 15.17493 0.758852 5.000694

Source DF Type I SS Mean Square F Value Pr > F

COS 1 3.58193903 3.58193903 6.22 0.0134

SIN 1 0.57413796 0.57413796 1.00 0.3192

Source DF Type III SS Mean Square F Value Pr > F

COS 1 3.58193900 3.58193900 6.22 0.0134

SIN 1 0.57413796 0.57413796 1.00 0.3192

Standard

Parameter Estimate Error t Value Pr > |t|

Intercept 4.981280855 0.05221677 95.40 <.0001

COS 0.174722321 0.07005614 2.49 0.0134

SIN -0.077334363 0.07744998 -1.00 0.3192

AMPLITUDE, TMAX AND TMIN FOR hsa_miR_208a_3p 116

Obs AMPL TMAX TMIN

1 0.38214 22.25 10.25

**hsa_miR_221_3p** 117

The MEANS Procedure

Variable Label Mean Std Dev Std Error Minimum Maximum Pr > |t|

ƒƒƒƒƒƒƒƒƒƒƒƒƒƒƒƒƒƒƒƒƒƒƒƒƒƒƒƒƒƒƒƒƒƒƒƒƒƒƒƒƒƒƒƒƒƒƒƒƒƒƒƒƒƒƒƒƒƒƒƒƒƒƒƒƒƒƒƒƒƒƒƒƒƒƒƒƒƒƒƒƒƒƒƒƒƒƒƒƒƒƒƒƒƒƒƒƒƒƒƒƒƒƒƒƒƒƒƒƒƒƒƒƒƒ

COS 0.0415829 0.2892788 0.0590488 -0.4081200 0.5559807 0.4884

SIN 0.1920425 0.2113055 0.0431326 -0.2015254 0.5374138 0.0002

Intercept Intercept -5.7208240 0.7814997 0.1595230 -7.2615460 -4.2975894 <.0001

ƒƒƒƒƒƒƒƒƒƒƒƒƒƒƒƒƒƒƒƒƒƒƒƒƒƒƒƒƒƒƒƒƒƒƒƒƒƒƒƒƒƒƒƒƒƒƒƒƒƒƒƒƒƒƒƒƒƒƒƒƒƒƒƒƒƒƒƒƒƒƒƒƒƒƒƒƒƒƒƒƒƒƒƒƒƒƒƒƒƒƒƒƒƒƒƒƒƒƒƒƒƒƒƒƒƒƒƒƒƒƒƒƒƒ

hsa_miR_221_3p 118

model test

The GLM Procedure

Number of observations 216

hsa_miR_221_3p 119

model test

The GLM Procedure

Dependent Variable: variabel

Sum of

Source DF Squares Mean Square F Value Pr > F

Model 2 3.7433984 1.8716992 2.18 **0.1151**

Error 213 182.5132887 0.8568699

Corrected Total 215 186.2566870

R-Square Coeff Var Root MSE variabel Mean

0.020098 -16.19384 0.925673 -5.716204

Source DF Type I SS Mean Square F Value Pr > F

COS 1 0.20288565 0.20288565 0.24 0.6270

SIN 1 3.54051272 3.54051272 4.13 0.0433

Source DF Type III SS Mean Square F Value Pr > F

COS 1 0.20288567 0.20288567 0.24 0.6270

SIN 1 3.54051272 3.54051272 4.13 0.0433

Standard

Parameter Estimate Error t Value Pr > |t|

Intercept -5.720824026 0.06369575 -89.81 <.0001

COS 0.041582920 0.08545682 0.49 0.6270

SIN 0.192042548 0.09447607 2.03 0.0433

AMPLITUDE, TMAX AND TMIN FOR hsa_miR_221_3p 120

Obs AMPL TMAX TMIN

1 0.39299 5.11 17.11

**hsa_miR_222_3p** 121

The MEANS Procedure

Variable Label Mean Std Dev Std Error Minimum Maximum Pr > |t|

ƒƒƒƒƒƒƒƒƒƒƒƒƒƒƒƒƒƒƒƒƒƒƒƒƒƒƒƒƒƒƒƒƒƒƒƒƒƒƒƒƒƒƒƒƒƒƒƒƒƒƒƒƒƒƒƒƒƒƒƒƒƒƒƒƒƒƒƒƒƒƒƒƒƒƒƒƒƒƒƒƒƒƒƒƒƒƒƒƒƒƒƒƒƒƒƒƒƒƒƒƒƒƒƒƒƒƒƒƒƒƒƒƒƒ

COS -0.0756606 0.2670393 0.0545092 -0.7679426 0.2821669 0.1784

SIN -0.1885249 0.3083511 0.0629419 -0.7643897 0.6046930 0.0065

Intercept Intercept -0.9841396 0.6006787 0.1226130 -1.6144494 0.9551339 <.0001

ƒƒƒƒƒƒƒƒƒƒƒƒƒƒƒƒƒƒƒƒƒƒƒƒƒƒƒƒƒƒƒƒƒƒƒƒƒƒƒƒƒƒƒƒƒƒƒƒƒƒƒƒƒƒƒƒƒƒƒƒƒƒƒƒƒƒƒƒƒƒƒƒƒƒƒƒƒƒƒƒƒƒƒƒƒƒƒƒƒƒƒƒƒƒƒƒƒƒƒƒƒƒƒƒƒƒƒƒƒƒƒƒƒƒ

hsa_miR_222_3p 122

model test

The GLM Procedure

Number of observations 216

hsa_miR_222_3p 123

model test

The GLM Procedure

Dependent Variable: variabel

Sum of

Source DF Squares Mean Square F Value Pr > F

Model 2 4.0836759 2.0418379 2.87 **0.0590**

Error 213 151.6452237 0.7119494

Corrected Total 215 155.7288995

R-Square Coeff Var Root MSE variabel Mean

0.026223 -85.01074 0.843771 -0.992546

Source DF Type I SS Mean Square F Value Pr > F

COS 1 0.67167686 0.67167686 0.94 0.3325

SIN 1 3.41199900 3.41199900 4.79 0.0297

Source DF Type III SS Mean Square F Value Pr > F

COS 1 0.67167689 0.67167689 0.94 0.3325

SIN 1 3.41199900 3.41199900 4.79 0.0297

Standard

Parameter Estimate Error t Value Pr > |t|

Intercept -.9841395706 0.05806008 -16.95 <.0001

COS -.0756605512 0.07789577 -0.97 0.3325

SIN -.1885249475 0.08611701 -2.19 0.0297

AMPLITUDE, TMAX AND TMIN FOR hsa_miR_222_3p 124

Obs AMPL TMAX TMIN

1 0.40628 16.33 4.33

**hsa_miR_223_3p** 125

The MEANS Procedure

Variable Label Mean Std Dev Std Error Minimum Maximum Pr > |t|

ƒƒƒƒƒƒƒƒƒƒƒƒƒƒƒƒƒƒƒƒƒƒƒƒƒƒƒƒƒƒƒƒƒƒƒƒƒƒƒƒƒƒƒƒƒƒƒƒƒƒƒƒƒƒƒƒƒƒƒƒƒƒƒƒƒƒƒƒƒƒƒƒƒƒƒƒƒƒƒƒƒƒƒƒƒƒƒƒƒƒƒƒƒƒƒƒƒƒƒƒƒƒƒƒƒƒƒƒƒƒƒƒƒƒ

COS 0.0553610 0.2823097 0.0576262 -0.4432252 0.5565622 0.3467

SIN 0.1810619 0.2740023 0.0559305 -0.3390774 0.7032501 0.0036

Intercept Intercept 0.3580155 0.7086026 0.1446429 -0.8798877 1.5499354 0.0211

ƒƒƒƒƒƒƒƒƒƒƒƒƒƒƒƒƒƒƒƒƒƒƒƒƒƒƒƒƒƒƒƒƒƒƒƒƒƒƒƒƒƒƒƒƒƒƒƒƒƒƒƒƒƒƒƒƒƒƒƒƒƒƒƒƒƒƒƒƒƒƒƒƒƒƒƒƒƒƒƒƒƒƒƒƒƒƒƒƒƒƒƒƒƒƒƒƒƒƒƒƒƒƒƒƒƒƒƒƒƒƒƒƒƒ

hsa_miR_223_3p 126

model test

The GLM Procedure

Number of observations 216

hsa_miR_223_3p 127

model test

The GLM Procedure

Dependent Variable: variabel

Sum of

Source DF Squares Mean Square F Value Pr > F

Model 2 3.5068149 1.7534075 2.34 **0.0984**

Error 213 159.2956351 0.7478668

Corrected Total 215 162.8024500

R-Square Coeff Var Root MSE variabel Mean

0.021540 237.4717 0.864793 0.364167

Source DF Type I SS Mean Square F Value Pr > F

COS 1 0.35960747 0.35960747 0.48 0.4888

SIN 1 3.14720746 3.14720746 4.21 0.0415

Source DF Type III SS Mean Square F Value Pr > F

COS 1 0.35960749 0.35960749 0.48 0.4888

SIN 1 3.14720746 3.14720746 4.21 0.0415

Standard

Parameter Estimate Error t Value Pr > |t|

Intercept 0.3580154501 0.05950661 6.02 <.0001

COS 0.0553609662 0.07983649 0.69 0.4888

SIN 0.1810618972 0.08826256 2.05 0.0415

AMPLITUDE, TMAX AND TMIN FOR hsa_miR_223_3p 128

Obs AMPL TMAX TMIN

1 0.37867 4.52 16.52

**hsa_miR_342_3p** 129

The MEANS Procedure

Variable Label Mean Std Dev Std Error Minimum Maximum Pr > |t|

ƒƒƒƒƒƒƒƒƒƒƒƒƒƒƒƒƒƒƒƒƒƒƒƒƒƒƒƒƒƒƒƒƒƒƒƒƒƒƒƒƒƒƒƒƒƒƒƒƒƒƒƒƒƒƒƒƒƒƒƒƒƒƒƒƒƒƒƒƒƒƒƒƒƒƒƒƒƒƒƒƒƒƒƒƒƒƒƒƒƒƒƒƒƒƒƒƒƒƒƒƒƒƒƒƒƒƒƒƒƒƒƒƒƒ

COS -0.0518884 0.2622340 0.0535283 -0.4800507 0.4990123 0.3424

SIN 0.0827948 0.2752275 0.0561806 -0.3511270 0.6669418 0.1541

Intercept Intercept -9.8505772 1.0893118 0.2223548 -12.0627206 -7.9532236 <.0001

ƒƒƒƒƒƒƒƒƒƒƒƒƒƒƒƒƒƒƒƒƒƒƒƒƒƒƒƒƒƒƒƒƒƒƒƒƒƒƒƒƒƒƒƒƒƒƒƒƒƒƒƒƒƒƒƒƒƒƒƒƒƒƒƒƒƒƒƒƒƒƒƒƒƒƒƒƒƒƒƒƒƒƒƒƒƒƒƒƒƒƒƒƒƒƒƒƒƒƒƒƒƒƒƒƒƒƒƒƒƒƒƒƒƒ

hsa_miR_342_3p 130

model test

The GLM Procedure

Number of observations 216

hsa_miR_342_3p 131

model test

The GLM Procedure

Dependent Variable: variabel

Sum of

Source DF Squares Mean Square F Value Pr > F

Model 2 0.9739874 0.4869937 0.34 **0.7120**

Error 213 304.8980233 1.4314461

Corrected Total 215 305.8720106

R-Square Coeff Var Root MSE variabel Mean

0.003184 -12.13869 1.196431 -9.856343

Source DF Type I SS Mean Square F Value Pr > F

COS 1 0.31590860 0.31590860 0.22 0.6390

SIN 1 0.65807875 0.65807875 0.46 0.4985

Source DF Type III SS Mean Square F Value Pr > F

COS 1 0.31590859 0.31590859 0.22 0.6390

SIN 1 0.65807875 0.65807875 0.46 0.4985

Standard

Parameter Estimate Error t Value Pr > |t|

Intercept -9.850577219 0.08232667 -119.65 <.0001

COS -0.051888369 0.11045282 -0.47 0.6390

SIN 0.082794848 0.12211018 0.68 0.4985

AMPLITUDE, TMAX AND TMIN FOR hsa_miR_342_3p 132

Obs AMPL TMAX TMIN

1 0.19542 8.08 20.08

**hsa_miR_383_5p** 133

The MEANS Procedure

Variable Label Mean Std Dev Std Error Minimum Maximum Pr > |t|

ƒƒƒƒƒƒƒƒƒƒƒƒƒƒƒƒƒƒƒƒƒƒƒƒƒƒƒƒƒƒƒƒƒƒƒƒƒƒƒƒƒƒƒƒƒƒƒƒƒƒƒƒƒƒƒƒƒƒƒƒƒƒƒƒƒƒƒƒƒƒƒƒƒƒƒƒƒƒƒƒƒƒƒƒƒƒƒƒƒƒƒƒƒƒƒƒƒƒƒƒƒƒƒƒƒƒƒƒƒƒƒƒƒƒ

COS -0.0177869 0.2014177 0.0411142 -0.2440934 0.7652017 0.6693

SIN -0.0711824 0.3006713 0.0613743 -0.5229683 0.5761790 0.2580

Intercept Intercept -2.5831626 0.7620475 0.1555523 -4.1528002 -1.5950504 <.0001

ƒƒƒƒƒƒƒƒƒƒƒƒƒƒƒƒƒƒƒƒƒƒƒƒƒƒƒƒƒƒƒƒƒƒƒƒƒƒƒƒƒƒƒƒƒƒƒƒƒƒƒƒƒƒƒƒƒƒƒƒƒƒƒƒƒƒƒƒƒƒƒƒƒƒƒƒƒƒƒƒƒƒƒƒƒƒƒƒƒƒƒƒƒƒƒƒƒƒƒƒƒƒƒƒƒƒƒƒƒƒƒƒƒƒ

hsa_miR_383_5p 134

model test

The GLM Procedure

Number of observations 216

hsa_miR_383_5p 135

model test

The GLM Procedure

Dependent Variable: variabel

Sum of

Source DF Squares Mean Square F Value Pr > F

Model 2 0.5235474 0.2617737 0.29 **0.7465**

Error 213 190.4618485 0.8941871

Corrected Total 215 190.9853958

R-Square Coeff Var Root MSE variabel Mean

0.002741 -36.57887 0.945615 -2.585139

Source DF Type I SS Mean Square F Value Pr > F

COS 1 0.03712101 0.03712101 0.04 0.8387

SIN 1 0.48642635 0.48642635 0.54 0.4616

Source DF Type III SS Mean Square F Value Pr > F

COS 1 0.03712102 0.03712102 0.04 0.8387

SIN 1 0.48642635 0.48642635 0.54 0.4616

Standard

Parameter Estimate Error t Value Pr > |t|

Intercept -2.583162572 0.06506797 -39.70 <.0001

COS -0.017786858 0.08729784 -0.20 0.8387

SIN -0.071182449 0.09651139 -0.74 0.4616

AMPLITUDE, TMAX AND TMIN FOR hsa_miR_383_5p 136

Obs AMPL TMAX TMIN

1 0.14674 17.04 5.04

**hsa_miR_409_3p** 137

The MEANS Procedure

Variable Label Mean Std Dev Std Error Minimum Maximum Pr > |t|

ƒƒƒƒƒƒƒƒƒƒƒƒƒƒƒƒƒƒƒƒƒƒƒƒƒƒƒƒƒƒƒƒƒƒƒƒƒƒƒƒƒƒƒƒƒƒƒƒƒƒƒƒƒƒƒƒƒƒƒƒƒƒƒƒƒƒƒƒƒƒƒƒƒƒƒƒƒƒƒƒƒƒƒƒƒƒƒƒƒƒƒƒƒƒƒƒƒƒƒƒƒƒƒƒƒƒƒƒƒƒƒƒƒƒ

COS 0.0379716 0.3733797 0.0762158 -0.7281178 0.6552323 0.6231

SIN -0.1044143 0.6108065 0.1246804 -0.9297413 1.7537884 0.4110

Intercept Intercept 4.2197624 0.7970391 0.1626949 2.5616409 5.3110080 <.0001

ƒƒƒƒƒƒƒƒƒƒƒƒƒƒƒƒƒƒƒƒƒƒƒƒƒƒƒƒƒƒƒƒƒƒƒƒƒƒƒƒƒƒƒƒƒƒƒƒƒƒƒƒƒƒƒƒƒƒƒƒƒƒƒƒƒƒƒƒƒƒƒƒƒƒƒƒƒƒƒƒƒƒƒƒƒƒƒƒƒƒƒƒƒƒƒƒƒƒƒƒƒƒƒƒƒƒƒƒƒƒƒƒƒƒ

hsa_miR_409_3p 138

model test

The GLM Procedure

Number of observations 216

hsa_miR_409_3p 139

model test

The GLM Procedure

Dependent Variable: variabel

Sum of

Source DF Squares Mean Square F Value Pr > F

Model 2 1.2158016 0.6079008 0.34 **0.7105**

Error 213 378.2121744 1.7756440

Corrected Total 215 379.4279759

R-Square Coeff Var Root MSE variabel Mean

0.003204 31.54685 1.332533 4.223981

Source DF Type I SS Mean Square F Value Pr > F

COS 1 0.16917607 0.16917607 0.10 0.7579

SIN 1 1.04662549 1.04662549 0.59 0.4435

Source DF Type III SS Mean Square F Value Pr > F

COS 1 0.16917606 0.16917606 0.10 0.7579

SIN 1 1.04662549 1.04662549 0.59 0.4435

Standard

Parameter Estimate Error t Value Pr > |t|

Intercept 4.219762416 0.09169190 46.02 <.0001

COS 0.037971587 0.12301760 0.31 0.7579

SIN -0.104414313 0.13600107 -0.77 0.4435

AMPLITUDE, TMAX AND TMIN FOR hsa_miR_409_3p 140

Obs AMPL TMAX TMIN

1 0.22221 19.20 7.20

**hsa_miR_451a** 141

The MEANS Procedure

Variable Label Mean Std Dev Std Error Minimum Maximum Pr > |t|

ƒƒƒƒƒƒƒƒƒƒƒƒƒƒƒƒƒƒƒƒƒƒƒƒƒƒƒƒƒƒƒƒƒƒƒƒƒƒƒƒƒƒƒƒƒƒƒƒƒƒƒƒƒƒƒƒƒƒƒƒƒƒƒƒƒƒƒƒƒƒƒƒƒƒƒƒƒƒƒƒƒƒƒƒƒƒƒƒƒƒƒƒƒƒƒƒƒƒƒƒƒƒƒƒƒƒƒƒƒƒƒƒƒƒ

COS -0.0029738 0.1122186 0.0229065 -0.2252522 0.1980807 0.8978

SIN 0.0585349 0.1069396 0.0218290 -0.1314950 0.2500089 0.0133

Intercept Intercept -3.2999937 0.5157219 0.1052713 -4.4018391 -2.4083406 <.0001

ƒƒƒƒƒƒƒƒƒƒƒƒƒƒƒƒƒƒƒƒƒƒƒƒƒƒƒƒƒƒƒƒƒƒƒƒƒƒƒƒƒƒƒƒƒƒƒƒƒƒƒƒƒƒƒƒƒƒƒƒƒƒƒƒƒƒƒƒƒƒƒƒƒƒƒƒƒƒƒƒƒƒƒƒƒƒƒƒƒƒƒƒƒƒƒƒƒƒƒƒƒƒƒƒƒƒƒƒƒƒƒƒƒƒ

hsa_miR_451a 142

model test

The GLM Procedure

Number of observations 216

hsa_miR_451a 143

model test

The GLM Procedure

Dependent Variable: variabel

Sum of

Source DF Squares Mean Square F Value Pr > F

Model 2 0.32996528 0.16498264 0.53 **0.5907**

Error 213 66.59531203 0.31265405

Corrected Total 215 66.92527731

R-Square Coeff Var Root MSE variabel Mean

0.004930 -16.94242 0.559155 -3.300324

Source DF Type I SS Mean Square F Value Pr > F

COS 1 0.00103764 0.00103764 0.00 0.9541

SIN 1 0.32892764 0.32892764 1.05 0.3062

Source DF Type III SS Mean Square F Value Pr > F

COS 1 0.00103764 0.00103764 0.00 0.9541

SIN 1 0.32892764 0.32892764 1.05 0.3062

Standard

Parameter Estimate Error t Value Pr > |t|

Intercept -3.299993650 0.03847557 -85.77 <.0001

COS -0.002973811 0.05162040 -0.06 0.9541

SIN 0.058534857 0.05706849 1.03 0.3062

AMPLITUDE, TMAX AND TMIN FOR hsa_miR_451a 144

Obs AMPL TMAX TMIN

1 0.11722 6.12 18.12

**hsa_miR_590_5p** 145

The MEANS Procedure

Variable Label Mean Std Dev Std Error Minimum Maximum Pr > |t|

ƒƒƒƒƒƒƒƒƒƒƒƒƒƒƒƒƒƒƒƒƒƒƒƒƒƒƒƒƒƒƒƒƒƒƒƒƒƒƒƒƒƒƒƒƒƒƒƒƒƒƒƒƒƒƒƒƒƒƒƒƒƒƒƒƒƒƒƒƒƒƒƒƒƒƒƒƒƒƒƒƒƒƒƒƒƒƒƒƒƒƒƒƒƒƒƒƒƒƒƒƒƒƒƒƒƒƒƒƒƒƒƒƒƒ

COS -0.1427515 0.2903440 0.0592662 -0.6207327 0.4771539 0.0244

SIN 0.0960521 0.3005642 0.0613524 -0.5386270 0.5841295 0.1311

Intercept Intercept -4.3452035 0.8353731 0.1705198 -5.5098357 -2.6801245 <.0001

ƒƒƒƒƒƒƒƒƒƒƒƒƒƒƒƒƒƒƒƒƒƒƒƒƒƒƒƒƒƒƒƒƒƒƒƒƒƒƒƒƒƒƒƒƒƒƒƒƒƒƒƒƒƒƒƒƒƒƒƒƒƒƒƒƒƒƒƒƒƒƒƒƒƒƒƒƒƒƒƒƒƒƒƒƒƒƒƒƒƒƒƒƒƒƒƒƒƒƒƒƒƒƒƒƒƒƒƒƒƒƒƒƒƒ

hsa_miR_590_5p 146

model test

The GLM Procedure

Number of observations 216

hsa_miR_590_5p 147

model test

The GLM Procedure

Dependent Variable: variabel

Sum of

Source DF Squares Mean Square F Value Pr > F

Model 2 3.2767155 1.6383578 1.49 **0.2279**

Error 213 234.3329395 1.1001546

Corrected Total 215 237.6096551

R-Square Coeff Var Root MSE variabel Mean

0.013790 -24.05107 1.048883 -4.361065

Source DF Type I SS Mean Square F Value Pr > F

COS 1 2.39101814 2.39101814 2.17 0.1419

SIN 1 0.88569741 0.88569741 0.81 0.3706

Source DF Type III SS Mean Square F Value Pr > F

COS 1 2.39101811 2.39101811 2.17 0.1419

SIN 1 0.88569741 0.88569741 0.81 0.3706

Standard

Parameter Estimate Error t Value Pr > |t|

Intercept -4.345203537 0.07217386 -60.20 <.0001

COS -0.142751516 0.09683139 -1.47 0.1419

SIN 0.096052145 0.10705113 0.90 0.3706

AMPLITUDE, TMAX AND TMIN FOR hsa_miR_590_5p 148

Obs AMPL TMAX TMIN

1 0.34412 9.44 21.44

**hsa_miR_638** 149

The MEANS Procedure

Variable Label Mean Std Dev Std Error Minimum Maximum Pr > |t|

ƒƒƒƒƒƒƒƒƒƒƒƒƒƒƒƒƒƒƒƒƒƒƒƒƒƒƒƒƒƒƒƒƒƒƒƒƒƒƒƒƒƒƒƒƒƒƒƒƒƒƒƒƒƒƒƒƒƒƒƒƒƒƒƒƒƒƒƒƒƒƒƒƒƒƒƒƒƒƒƒƒƒƒƒƒƒƒƒƒƒƒƒƒƒƒƒƒƒƒƒƒƒƒƒƒƒƒƒƒƒƒƒƒƒ

COS 0.0069234 0.1836994 0.0374975 -0.3258996 0.3308714 0.8551

SIN -0.0173639 0.1293302 0.0263994 -0.3703858 0.1835965 0.5172

Intercept Intercept -2.9821119 0.9369861 0.1912615 -4.3972101 -1.7625134 <.0001

ƒƒƒƒƒƒƒƒƒƒƒƒƒƒƒƒƒƒƒƒƒƒƒƒƒƒƒƒƒƒƒƒƒƒƒƒƒƒƒƒƒƒƒƒƒƒƒƒƒƒƒƒƒƒƒƒƒƒƒƒƒƒƒƒƒƒƒƒƒƒƒƒƒƒƒƒƒƒƒƒƒƒƒƒƒƒƒƒƒƒƒƒƒƒƒƒƒƒƒƒƒƒƒƒƒƒƒƒƒƒƒƒƒƒ

hsa_miR_638 150

model test

The GLM Procedure

Number of observations 216

hsa_miR_638 151

model test

The GLM Procedure

Dependent Variable: variabel

Sum of

Source DF Squares Mean Square F Value Pr > F

Model 2 0.0345686 0.0172843 0.02 **0.9826**

Error 213 210.0733420 0.9862598

Corrected Total 215 210.1079106

R-Square Coeff Var Root MSE variabel Mean

0.000165 -33.31070 0.993106 -2.981343

Source DF Type I SS Mean Square F Value Pr > F

COS 1 0.00562417 0.00562417 0.01 0.9399

SIN 1 0.02894447 0.02894447 0.03 0.8641

Source DF Type III SS Mean Square F Value Pr > F

COS 1 0.00562417 0.00562417 0.01 0.9399

SIN 1 0.02894447 0.02894447 0.03 0.8641

Standard

Parameter Estimate Error t Value Pr > |t|

Intercept -2.982111858 0.06833587 -43.64 <.0001

COS 0.006923386 0.09168219 0.08 0.9399

SIN -0.017363896 0.10135847 -0.17 0.8641

AMPLITUDE, TMAX AND TMIN FOR hsa_miR_638 152

Obs AMPL TMAX TMIN

1 0.037387 19.27 7.27

**hsa_miR_708_5p** 153

The MEANS Procedure

Variable Label Mean Std Dev Std Error Minimum Maximum Pr > |t|

ƒƒƒƒƒƒƒƒƒƒƒƒƒƒƒƒƒƒƒƒƒƒƒƒƒƒƒƒƒƒƒƒƒƒƒƒƒƒƒƒƒƒƒƒƒƒƒƒƒƒƒƒƒƒƒƒƒƒƒƒƒƒƒƒƒƒƒƒƒƒƒƒƒƒƒƒƒƒƒƒƒƒƒƒƒƒƒƒƒƒƒƒƒƒƒƒƒƒƒƒƒƒƒƒƒƒƒƒƒƒƒƒƒƒ

COS -0.1073272 0.3393873 0.0692771 -0.8430983 0.5807582 0.1350

SIN -0.2341168 0.4270638 0.0871740 -1.0399317 0.8183846 0.0132

Intercept Intercept -0.0048340 0.7539583 0.1539011 -1.1018627 1.8060651 0.9752

ƒƒƒƒƒƒƒƒƒƒƒƒƒƒƒƒƒƒƒƒƒƒƒƒƒƒƒƒƒƒƒƒƒƒƒƒƒƒƒƒƒƒƒƒƒƒƒƒƒƒƒƒƒƒƒƒƒƒƒƒƒƒƒƒƒƒƒƒƒƒƒƒƒƒƒƒƒƒƒƒƒƒƒƒƒƒƒƒƒƒƒƒƒƒƒƒƒƒƒƒƒƒƒƒƒƒƒƒƒƒƒƒƒƒ

hsa_miR_708_5p 154

model test

The GLM Procedure

Number of observations 216

hsa_miR_708_5p 155

model test

The GLM Procedure

Dependent Variable: variabel

Sum of

Source DF Squares Mean Square F Value Pr > F

Model 2 6.6134027 3.3067013 2.87 **0.0587**

Error 213 245.1547288 1.1509612

Corrected Total 215 251.7681315

R-Square Coeff Var Root MSE variabel Mean

0.026268 -6401.408 1.072829 -0.016759

Source DF Type I SS Mean Square F Value Pr > F

COS 1 1.35157749 1.35157749 1.17 0.2797

SIN 1 5.26182520 5.26182520 4.57 0.0336

Source DF Type III SS Mean Square F Value Pr > F

COS 1 1.35157755 1.35157755 1.17 0.2797

SIN 1 5.26182520 5.26182520 4.57 0.0336

Standard

Parameter Estimate Error t Value Pr > |t|

Intercept -.0048340183 0.07382159 -0.07 0.9479

COS -.1073271954 0.09904205 -1.08 0.2797

SIN -.2341168052 0.10949511 -2.14 0.0336

AMPLITUDE, TMAX AND TMIN FOR hsa_miR_708_5p 156

Obs AMPL TMAX TMIN

1 0.51509 16.21 4.21

**hsa_miR_15a_5p** 157

The MEANS Procedure

Variable Label Mean Std Dev Std Error Minimum Maximum Pr > |t|

ƒƒƒƒƒƒƒƒƒƒƒƒƒƒƒƒƒƒƒƒƒƒƒƒƒƒƒƒƒƒƒƒƒƒƒƒƒƒƒƒƒƒƒƒƒƒƒƒƒƒƒƒƒƒƒƒƒƒƒƒƒƒƒƒƒƒƒƒƒƒƒƒƒƒƒƒƒƒƒƒƒƒƒƒƒƒƒƒƒƒƒƒƒƒƒƒƒƒƒƒƒƒƒƒƒƒƒƒƒƒƒƒƒƒ

COS -0.4366269 0.6704093 0.1368467 -1.8613114 0.8941417 0.0041

SIN 0.6106338 1.1885352 0.2426087 -3.1413350 3.3335873 0.0193

Intercept Intercept -4.4451474 1.1803414 0.2409362 -7.3918022 -3.0358740 <.0001

ƒƒƒƒƒƒƒƒƒƒƒƒƒƒƒƒƒƒƒƒƒƒƒƒƒƒƒƒƒƒƒƒƒƒƒƒƒƒƒƒƒƒƒƒƒƒƒƒƒƒƒƒƒƒƒƒƒƒƒƒƒƒƒƒƒƒƒƒƒƒƒƒƒƒƒƒƒƒƒƒƒƒƒƒƒƒƒƒƒƒƒƒƒƒƒƒƒƒƒƒƒƒƒƒƒƒƒƒƒƒƒƒƒƒ

hsa_miR_15a_5p 158

model test

The GLM Procedure

Number of observations 216

NOTE: Due to missing values, only 212 observations can be used in this analysis.

hsa_miR_15a_5p 159

model test

The GLM Procedure

Dependent Variable: variabel

Sum of

Source DF Squares Mean Square F Value Pr > F

Model 2 45.0987143 22.5493572 5.08 **0.0070**

Error 209 928.1113890 4.4407243

Corrected Total 211 973.2101033

R-Square Coeff Var Root MSE variabel Mean

0.046340 -47.66178 2.107303 -4.421368

Source DF Type I SS Mean Square F Value Pr > F

COS 1 13.15054996 13.15054996 2.96 0.0868

SIN 1 31.94816436 31.94816436 7.19 0.0079

Source DF Type III SS Mean Square F Value Pr > F

COS 1 13.32030970 13.32030970 3.00 0.0848

SIN 1 31.94816436 31.94816436 7.19 0.0079

Standard

Parameter Estimate Error t Value Pr > |t|

Intercept -4.390656611 0.14595343 -30.08 <.0001

COS -0.340995802 0.19688779 -1.73 0.0848

SIN 0.578402737 0.21564247 2.68 0.0079

AMPLITUDE, TMAX AND TMIN FOR hsa_miR_15a_5p 160

Obs AMPL TMAX TMIN

1 1.50136 8.22 20.22

**hsa_miR_208b_3p** 161

The MEANS Procedure

Variable Label Mean Std Dev Std Error Minimum Maximum Pr > |t|

ƒƒƒƒƒƒƒƒƒƒƒƒƒƒƒƒƒƒƒƒƒƒƒƒƒƒƒƒƒƒƒƒƒƒƒƒƒƒƒƒƒƒƒƒƒƒƒƒƒƒƒƒƒƒƒƒƒƒƒƒƒƒƒƒƒƒƒƒƒƒƒƒƒƒƒƒƒƒƒƒƒƒƒƒƒƒƒƒƒƒƒƒƒƒƒƒƒƒƒƒƒƒƒƒƒƒƒƒƒƒƒƒƒƒ

COS 0.0073129 0.8207999 0.1675451 -1.1461122 1.4394098 0.9656

SIN 0.0929329 1.2033579 0.2456344 -2.8932122 2.2553711 0.7086

Intercept Intercept -9.2600982 1.1636452 0.2375281 -10.9649122 -7.1234673 <.0001

ƒƒƒƒƒƒƒƒƒƒƒƒƒƒƒƒƒƒƒƒƒƒƒƒƒƒƒƒƒƒƒƒƒƒƒƒƒƒƒƒƒƒƒƒƒƒƒƒƒƒƒƒƒƒƒƒƒƒƒƒƒƒƒƒƒƒƒƒƒƒƒƒƒƒƒƒƒƒƒƒƒƒƒƒƒƒƒƒƒƒƒƒƒƒƒƒƒƒƒƒƒƒƒƒƒƒƒƒƒƒƒƒƒƒ

hsa_miR_208b_3p 162

model test

The GLM Procedure

Number of observations 216

NOTE: Due to missing values, only 198 observations can be used in this analysis.

hsa_miR_208b_3p 163

model test

The GLM Procedure

Dependent Variable: variabel

Sum of

Source DF Squares Mean Square F Value Pr > F

Model 2 0.1029437 0.0514719 0.01 **0.9883**

Error 195 850.7340952 4.3627389

Corrected Total 197 850.8370389

R-Square Coeff Var Root MSE variabel Mean

0.000121 -22.60652 2.088717 -9.239444

Source DF Type I SS Mean Square F Value Pr > F

COS 1 0.10087135 0.10087135 0.02 0.8793

SIN 1 0.00207239 0.00207239 0.00 0.9826

Source DF Type III SS Mean Square F Value Pr > F

COS 1 0.10039856 0.10039856 0.02 0.8796

SIN 1 0.00207239 0.00207239 0.00 0.9826

Standard

Parameter Estimate Error t Value Pr > |t|

Intercept -9.236074879 0.15009460 -61.54 <.0001

COS -0.030762177 0.20278365 -0.15 0.8796

SIN -0.004812564 0.22081097 -0.02 0.9826

AMPLITUDE, TMAX AND TMIN FOR hsa_miR_208b_3p 164

Obs AMPL TMAX TMIN

1 0.18644 5.42 17.42

**hsa_miR_375** 165

The MEANS Procedure

Variable Label Mean Std Dev Std Error Minimum Maximum Pr > |t|

ƒƒƒƒƒƒƒƒƒƒƒƒƒƒƒƒƒƒƒƒƒƒƒƒƒƒƒƒƒƒƒƒƒƒƒƒƒƒƒƒƒƒƒƒƒƒƒƒƒƒƒƒƒƒƒƒƒƒƒƒƒƒƒƒƒƒƒƒƒƒƒƒƒƒƒƒƒƒƒƒƒƒƒƒƒƒƒƒƒƒƒƒƒƒƒƒƒƒƒƒƒƒƒƒƒƒƒƒƒƒƒƒƒƒ

COS -0.4289180 0.5333000 0.1088594 -1.3440775 0.9890827 0.0007

SIN -0.3838679 0.4695582 0.0958482 -1.1988745 0.7377135 0.0006

Intercept Intercept -9.3860984 1.0564215 0.2156411 -12.0712500 -7.9888762 <.0001

ƒƒƒƒƒƒƒƒƒƒƒƒƒƒƒƒƒƒƒƒƒƒƒƒƒƒƒƒƒƒƒƒƒƒƒƒƒƒƒƒƒƒƒƒƒƒƒƒƒƒƒƒƒƒƒƒƒƒƒƒƒƒƒƒƒƒƒƒƒƒƒƒƒƒƒƒƒƒƒƒƒƒƒƒƒƒƒƒƒƒƒƒƒƒƒƒƒƒƒƒƒƒƒƒƒƒƒƒƒƒƒƒƒƒ

hsa_miR_375 166

model test

The GLM Procedure

Number of observations 216

NOTE: Due to missing values, only 209 observations can be used in this analysis.

hsa_miR_375 167

model test

The GLM Procedure

Dependent Variable: variabel

Sum of

Source DF Squares Mean Square F Value Pr > F

Model 2 32.8424366 16.4212183 8.88 **0.0002**

Error 206 380.8911156 1.8489860

Corrected Total 208 413.7335522

R-Square Coeff Var Root MSE variabel Mean

0.079381 -14.45524 1.359774 -9.406794

Source DF Type I SS Mean Square F Value Pr > F

COS 1 17.78634554 17.78634554 9.62 0.0022

SIN 1 15.05609101 15.05609101 8.14 0.0048

Source DF Type III SS Mean Square F Value Pr > F

COS 1 17.58966150 17.58966150 9.51 0.0023

SIN 1 15.05609101 15.05609101 8.14 0.0048

Standard

Parameter Estimate Error t Value Pr > |t|

Intercept -9.372971406 0.09479587 -98.88 <.0001

COS -0.393296371 0.12751404 -3.08 0.0023

SIN -0.401306984 0.14063306 -2.85 0.0048

AMPLITUDE, TMAX AND TMIN FOR hsa_miR_375 168

Obs AMPL TMAX TMIN

1 1.15122 14.47 2.47
